# Supplementary material for: Genomic surveillance of SARS-CoV-2 in Puerto Rico reveals emergence of an autochthonous lineage and early detection of variants
Source: Res Sq. 2022 Jan 20:rs.3.rs-1277781. Preprint. [Version 1] doi: 10.21203/rs.3.rs-1277781/v1 (PMC8786232; doi:10.21203/rs.3.rs-1277781/v1)
Supplement: Supplement 2 [file 0160df1a7b716913eb38bbe4.docx]

| **We gratefully acknowledge the following Authors from the Originating laboratories responsible for obtaining the specimens and the Submitting laboratories where genetic sequence data were generated and shared via the GISAID Initiative, on which this research is based.** | | | | | | |
| --- | --- | --- | --- | --- | --- | --- |
| **All submitters of data may be contacted directly via www.gisaid.org** | | | | | | |
| **Accession ID** | **Virus Name** | **Location** | **Collection Date** | **Originating Lab** | **Submitting Lab** | **Authors** |
| EPI_ISL_468145 | hCoV-19/Romania/Bucuresti-6451/2020 | Europe/Romania/Bucuresti | 2020-03-28 | [Romania, Bucharest] National Institute for Infectious Diseases] | [Romania, Bucharest] National Institute for Infectious Diseases] | Leontina Banica, Marius Cotic, Corina Casangiu, Marius Surleac, Simona Paraschiv |
| EPI_ISL_4911658 | hCoV-19/PuertoRico/PR-CDC-ASC210123672/2021 | North America/Puerto Rico | 2021-08-26 | Aegis Sciences Corporation | Centers for Disease Control and Prevention Division of Viral Diseases, Pathogen Discovery | Dakota Howard,Dhwani Batra,Peter Cook,Jason Caravas,Benjamin Rambo-Martin,Scott Sammons,Yvette Unoarumhi,Matthew Schmerer,Kristine Lacek,Tymeckia Kendall,Victoria Caban Figueroa,Shatavia Morrison,Christopher Gulvick,Erisa Sula,Cyndi Clark,Patrick Campbell,Rob Case,Vikramsinha Ghorpade,Holly Houdeshell,Ola Kvalvaag,Dillon Nall,Ethan Sanders,Alec Vest,Shaun Westlund,Matthew Hardison,Clinton Paden,Duncan MacCannell |
| EPI_ISL_5230591 | hCoV-19/PuertoRico/PR-CDC-ASC210178125/2021 | North America/Puerto Rico | 2021-08-24 | Aegis Sciences Corporation | Centers for Disease Control and Prevention Division of Viral Diseases, Pathogen Discovery | Dakota Howard,Dhwani Batra,Peter Cook,Jason Caravas,Benjamin Rambo-Martin,Scott Sammons,Yvette Unoarumhi,Matthew Schmerer,Kristine Lacek,Tymeckia Kendall,Victoria Caban Figueroa,Shatavia Morrison,Christopher Gulvick,Erisa Sula,Cyndi Clark,Patrick Campbell,Rob Case,Vikramsinha Ghorpade,Holly Houdeshell,Ola Kvalvaag,Dillon Nall,Ethan Sanders,Alec Vest,Shaun Westlund,Matthew Hardison,Clinton Paden,Duncan MacCannell |
| EPI_ISL_5985965 | hCoV-19/PuertoRico/PR-CDC-ASC210320180/2021 | North America/Puerto Rico | 2021-09-29 | Aegis Sciences Corporation | Centers for Disease Control and Prevention Division of Viral Diseases, Pathogen Discovery | Dakota Howard,Dhwani Batra,Peter Cook,Jason Caravas,Benjamin Rambo-Martin,Scott Sammons,Yvette Unoarumhi,Matthew Schmerer,Kristine Lacek,Tymeckia Kendall,Victoria Caban Figueroa,Shatavia Morrison,Christopher Gulvick,Erisa Sula,Cyndi Clark,Patrick Campbell,Rob Case,Vikramsinha Ghorpade,Holly Houdeshell,Ola Kvalvaag,Dillon Nall,Ethan Sanders,Alec Vest,Shaun Westlund,Matthew Hardison,Clinton Paden,Duncan MacCannell |
| EPI_ISL_2180568 | hCoV-19/USA/CA-CDC-ASC210061187/2021 | North America/USA/California | 2021-05-08 | Aegis Sciences Corporation | Centers for Disease Control and Prevention Division of Viral Diseases, Pathogen Discovery | Dakota Howard, Dhwani Batra, Peter W. Cook, Kara Moser, Adrian Paskey, Jason Caravas, Benjamin Rambo-Martin, Shatavia Morrison, Christopher Gulvick, Scott Sammons, Yvette Unoarumhi, Darlene Wagner, Matthew Schmerer, Cyndi Clark, Patrick Campbell, Rob Case, Vikramsinha Ghorpade, Holly Houdeshell, Ola Kvalvaag, Dillon Nall, Ethan Sanders, Alec Vest, Shaun Westlund, Matthew Hardison, Clinton R. Paden, Duncan MacCannell |
| EPI_ISL_3220355 | hCoV-19/USA/CA-CDC-ASC210155424/2021 | North America/USA/California | 2021-07-02 | Aegis Sciences Corporation | Centers for Disease Control and Prevention Division of Viral Diseases, Pathogen Discovery | Dakota Howard, Dhwani Batra, Peter W. Cook, Kara Moser, Adrian Paskey, Jason Caravas, Benjamin Rambo-Martin, Shatavia Morrison, Christopher Gulvick, Scott Sammons, Yvette Unoarumhi, Darlene Wagner, Matthew Schmerer, Cyndi Clark, Patrick Campbell, Rob Case, Vikramsinha Ghorpade, Holly Houdeshell, Ola Kvalvaag, Dillon Nall, Ethan Sanders, Alec Vest, Shaun Westlund, Matthew Hardison, Clinton R. Paden, Duncan MacCannell |
| EPI_ISL_3325136 | hCoV-19/USA/CA-CDC-ASC210159763/2021 | North America/USA/California | 2021-07-18 | Aegis Sciences Corporation | Centers for Disease Control and Prevention Division of Viral Diseases, Pathogen Discovery | Dakota Howard, Dhwani Batra, Peter W. Cook, Kara Moser, Adrian Paskey, Jason Caravas, Benjamin Rambo-Martin, Shatavia Morrison, Christopher Gulvick, Scott Sammons, Yvette Unoarumhi, Darlene Wagner, Matthew Schmerer, Cyndi Clark, Patrick Campbell, Rob Case, Vikramsinha Ghorpade, Holly Houdeshell, Ola Kvalvaag, Dillon Nall, Ethan Sanders, Alec Vest, Shaun Westlund, Matthew Hardison, Clinton R. Paden, Duncan MacCannell |
| EPI_ISL_3859009 | hCoV-19/USA/CA-CDC-ASC210185512/2021 | North America/USA/California | 2021-08-04 | Aegis Sciences Corporation | Centers for Disease Control and Prevention Division of Viral Diseases, Pathogen Discovery | Dakota Howard,Dhwani Batra,Peter Cook,Kara Moser,Adrian Paskey,Jason Caravas,Benjamin Rambo-Martin,Shatavia Morrison,Christopher Gulvick,Scott Sammons,Yvette Unoarumhi,Darlene Wagner,Matthew Schmerer,Cyndi Clark,Patrick Campbell,Rob Case,Vikramsinha Ghorpade,Holly Houdeshell,Ola Kvalvaag,Dillon Nall,Ethan Sanders,Alec Vest,Shaun Westlund,Matthew Hardison,Clinton Paden,Duncan MacCannell |
| EPI_ISL_3846475 | hCoV-19/USA/CA-CDC-ASC210204314/2021 | North America/USA/California | 2021-07-30 | Aegis Sciences Corporation | Centers for Disease Control and Prevention Division of Viral Diseases, Pathogen Discovery | Dakota Howard,Dhwani Batra,Peter Cook,Kara Moser,Adrian Paskey,Jason Caravas,Benjamin Rambo-Martin,Shatavia Morrison,Christopher Gulvick,Scott Sammons,Yvette Unoarumhi,Darlene Wagner,Matthew Schmerer,Cyndi Clark,Patrick Campbell,Rob Case,Vikramsinha Ghorpade,Holly Houdeshell,Ola Kvalvaag,Dillon Nall,Ethan Sanders,Alec Vest,Shaun Westlund,Matthew Hardison,Clinton Paden,Duncan MacCannell |
| EPI_ISL_3846476 | hCoV-19/USA/CA-CDC-ASC210204315/2021 | North America/USA/California | 2021-07-30 | Aegis Sciences Corporation | Centers for Disease Control and Prevention Division of Viral Diseases, Pathogen Discovery | Dakota Howard,Dhwani Batra,Peter Cook,Kara Moser,Adrian Paskey,Jason Caravas,Benjamin Rambo-Martin,Shatavia Morrison,Christopher Gulvick,Scott Sammons,Yvette Unoarumhi,Darlene Wagner,Matthew Schmerer,Cyndi Clark,Patrick Campbell,Rob Case,Vikramsinha Ghorpade,Holly Houdeshell,Ola Kvalvaag,Dillon Nall,Ethan Sanders,Alec Vest,Shaun Westlund,Matthew Hardison,Clinton Paden,Duncan MacCannell |
| EPI_ISL_3819064 | hCoV-19/USA/CA-CDC-ASC210208987/2021 | North America/USA/California | 2021-08-02 | Aegis Sciences Corporation | Centers for Disease Control and Prevention Division of Viral Diseases, Pathogen Discovery | Dakota Howard,Dhwani Batra,Peter Cook,Kara Moser,Adrian Paskey,Jason Caravas,Benjamin Rambo-Martin,Shatavia Morrison,Christopher Gulvick,Scott Sammons,Yvette Unoarumhi,Darlene Wagner,Matthew Schmerer,Cyndi Clark,Patrick Campbell,Rob Case,Vikramsinha Ghorpade,Holly Houdeshell,Ola Kvalvaag,Dillon Nall,Ethan Sanders,Alec Vest,Shaun Westlund,Matthew Hardison,Clinton Paden,Duncan MacCannell |
| EPI_ISL_3818867 | hCoV-19/USA/CA-CDC-ASC210209365/2021 | North America/USA/California | 2021-08-02 | Aegis Sciences Corporation | Centers for Disease Control and Prevention Division of Viral Diseases, Pathogen Discovery | Dakota Howard,Dhwani Batra,Peter Cook,Kara Moser,Adrian Paskey,Jason Caravas,Benjamin Rambo-Martin,Shatavia Morrison,Christopher Gulvick,Scott Sammons,Yvette Unoarumhi,Darlene Wagner,Matthew Schmerer,Cyndi Clark,Patrick Campbell,Rob Case,Vikramsinha Ghorpade,Holly Houdeshell,Ola Kvalvaag,Dillon Nall,Ethan Sanders,Alec Vest,Shaun Westlund,Matthew Hardison,Clinton Paden,Duncan MacCannell |
| EPI_ISL_3854041 | hCoV-19/USA/CA-CDC-ASC210239523/2021 | North America/USA/California | 2021-08-03 | Aegis Sciences Corporation | Centers for Disease Control and Prevention Division of Viral Diseases, Pathogen Discovery | Dakota Howard,Dhwani Batra,Peter Cook,Kara Moser,Adrian Paskey,Jason Caravas,Benjamin Rambo-Martin,Shatavia Morrison,Christopher Gulvick,Scott Sammons,Yvette Unoarumhi,Darlene Wagner,Matthew Schmerer,Cyndi Clark,Patrick Campbell,Rob Case,Vikramsinha Ghorpade,Holly Houdeshell,Ola Kvalvaag,Dillon Nall,Ethan Sanders,Alec Vest,Shaun Westlund,Matthew Hardison,Clinton Paden,Duncan MacCannell |
| EPI_ISL_3817977 | hCoV-19/USA/CA-CDC-ASC210245454/2021 | North America/USA/California | 2021-07-25 | Aegis Sciences Corporation | Centers for Disease Control and Prevention Division of Viral Diseases, Pathogen Discovery | Dakota Howard,Dhwani Batra,Peter Cook,Kara Moser,Adrian Paskey,Jason Caravas,Benjamin Rambo-Martin,Shatavia Morrison,Christopher Gulvick,Scott Sammons,Yvette Unoarumhi,Darlene Wagner,Matthew Schmerer,Cyndi Clark,Patrick Campbell,Rob Case,Vikramsinha Ghorpade,Holly Houdeshell,Ola Kvalvaag,Dillon Nall,Ethan Sanders,Alec Vest,Shaun Westlund,Matthew Hardison,Clinton Paden,Duncan MacCannell |
| EPI_ISL_3853603 | hCoV-19/USA/CA-CDC-ASC210270281/2021 | North America/USA/California | 2021-08-02 | Aegis Sciences Corporation | Centers for Disease Control and Prevention Division of Viral Diseases, Pathogen Discovery | Dakota Howard,Dhwani Batra,Peter Cook,Kara Moser,Adrian Paskey,Jason Caravas,Benjamin Rambo-Martin,Shatavia Morrison,Christopher Gulvick,Scott Sammons,Yvette Unoarumhi,Darlene Wagner,Matthew Schmerer,Cyndi Clark,Patrick Campbell,Rob Case,Vikramsinha Ghorpade,Holly Houdeshell,Ola Kvalvaag,Dillon Nall,Ethan Sanders,Alec Vest,Shaun Westlund,Matthew Hardison,Clinton Paden,Duncan MacCannell |
| EPI_ISL_2180670 | hCoV-19/USA/CO-CDC-ASC210061517/2021 | North America/USA/Colorado | 2021-05-10 | Aegis Sciences Corporation | Centers for Disease Control and Prevention Division of Viral Diseases, Pathogen Discovery | Dakota Howard, Dhwani Batra, Peter W. Cook, Kara Moser, Adrian Paskey, Jason Caravas, Benjamin Rambo-Martin, Shatavia Morrison, Christopher Gulvick, Scott Sammons, Yvette Unoarumhi, Darlene Wagner, Matthew Schmerer, Cyndi Clark, Patrick Campbell, Rob Case, Vikramsinha Ghorpade, Holly Houdeshell, Ola Kvalvaag, Dillon Nall, Ethan Sanders, Alec Vest, Shaun Westlund, Matthew Hardison, Clinton R. Paden, Duncan MacCannell |
| EPI_ISL_1835716 | hCoV-19/USA/CT-CDC-ASC210049167/2021 | North America/USA/Connecticut | 2021-04-06 | Aegis Sciences Corporation | Centers for Disease Control and Prevention Division of Viral Diseases, Pathogen Discovery | Dakota Howard, Dhwani Batra, Peter W. Cook, Kara Moser, Adrian Paskey, Jason Caravas, Benjamin Rambo-Martin, Shatavia Morrison, Christopher Gulvick, Scott Sammons, Yvette Unoarumhi, Darlene Wagner, Matthew Schmerer, Cyndi Clark, Patrick Campbell, Rob Case, Vikramsinha Ghorpade, Holly Houdeshell, Ola Kvalvaag, Dillon Nall, Ethan Sanders, Alec Vest, Shaun Westlund, Matthew Hardison, Clinton R. Paden, Duncan MacCannell |
| EPI_ISL_1835720 | hCoV-19/USA/CT-CDC-ASC210049172/2021 | North America/USA/Connecticut | 2021-04-06 | Aegis Sciences Corporation | Centers for Disease Control and Prevention Division of Viral Diseases, Pathogen Discovery | Dakota Howard, Dhwani Batra, Peter W. Cook, Kara Moser, Adrian Paskey, Jason Caravas, Benjamin Rambo-Martin, Shatavia Morrison, Christopher Gulvick, Scott Sammons, Yvette Unoarumhi, Darlene Wagner, Matthew Schmerer, Cyndi Clark, Patrick Campbell, Rob Case, Vikramsinha Ghorpade, Holly Houdeshell, Ola Kvalvaag, Dillon Nall, Ethan Sanders, Alec Vest, Shaun Westlund, Matthew Hardison, Clinton R. Paden, Duncan MacCannell |
| EPI_ISL_1550612 | hCoV-19/USA/FL-CDC-ASC210003523/2021 | North America/USA/Florida | 2021-03-10 | Aegis Sciences Corporation | Centers for Disease Control and Prevention Division of Viral Diseases, Pathogen Discovery | Dakota Howard, Dhwani Batra, Peter W. Cook, Kara Moser, Adrian Paskey, Jason Caravas, Benjamin Rambo-Martin, Shatavia Morrison, Christopher Gulvick, Scott Sammons, Yvette Unoarumhi, Darlene Wagner, Matthew Schmerer, Cyndi Clark, Patrick Campbell, Rob Case, Vikramsinha Ghorpade, Holly Houdeshell, Ola Kvalvaag, Dillon Nall, Ethan Sanders, Alec Vest, Shaun Westlund, Matthew Hardison, Clinton R. Paden, Duncan MacCannell |
| EPI_ISL_1562844 | hCoV-19/USA/FL-CDC-ASC210007433/2021 | North America/USA/Florida | 2021-03-16 | Aegis Sciences Corporation | Centers for Disease Control and Prevention Division of Viral Diseases, Pathogen Discovery | Dakota Howard, Dhwani Batra, Peter W. Cook, Kara Moser, Adrian Paskey, Jason Caravas, Benjamin Rambo-Martin, Shatavia Morrison, Christopher Gulvick, Scott Sammons, Yvette Unoarumhi, Darlene Wagner, Matthew Schmerer, Cyndi Clark, Patrick Campbell, Rob Case, Vikramsinha Ghorpade, Holly Houdeshell, Ola Kvalvaag, Dillon Nall, Ethan Sanders, Alec Vest, Shaun Westlund, Matthew Hardison, Clinton R. Paden, Duncan MacCannell |
| EPI_ISL_1667785 | hCoV-19/USA/FL-CDC-ASC210027664/2021 | North America/USA/Florida | 2021-03-30 | Aegis Sciences Corporation | Centers for Disease Control and Prevention Division of Viral Diseases, Pathogen Discovery | Dakota Howard, Dhwani Batra, Peter W. Cook, Kara Moser, Adrian Paskey, Jason Caravas, Benjamin Rambo-Martin, Shatavia Morrison, Christopher Gulvick, Scott Sammons, Yvette Unoarumhi, Darlene Wagner, Matthew Schmerer, Cyndi Clark, Patrick Campbell, Rob Case, Vikramsinha Ghorpade, Holly Houdeshell, Ola Kvalvaag, Dillon Nall, Ethan Sanders, Alec Vest, Shaun Westlund, Matthew Hardison, Clinton R. Paden, Duncan MacCannell |
| EPI_ISL_1688510 | hCoV-19/USA/FL-CDC-ASC210028017/2021 | North America/USA/Florida | 2021-03-28 | Aegis Sciences Corporation | Centers for Disease Control and Prevention Division of Viral Diseases, Pathogen Discovery | Dakota Howard, Dhwani Batra, Peter W. Cook, Kara Moser, Adrian Paskey, Jason Caravas, Benjamin Rambo-Martin, Shatavia Morrison, Christopher Gulvick, Scott Sammons, Yvette Unoarumhi, Darlene Wagner, Matthew Schmerer, Cyndi Clark, Patrick Campbell, Rob Case, Vikramsinha Ghorpade, Holly Houdeshell, Ola Kvalvaag, Dillon Nall, Ethan Sanders, Alec Vest, Shaun Westlund, Matthew Hardison, Clinton R. Paden, Duncan MacCannell |
| EPI_ISL_1688515 | hCoV-19/USA/FL-CDC-ASC210028021/2021 | North America/USA/Florida | 2021-03-28 | Aegis Sciences Corporation | Centers for Disease Control and Prevention Division of Viral Diseases, Pathogen Discovery | Dakota Howard, Dhwani Batra, Peter W. Cook, Kara Moser, Adrian Paskey, Jason Caravas, Benjamin Rambo-Martin, Shatavia Morrison, Christopher Gulvick, Scott Sammons, Yvette Unoarumhi, Darlene Wagner, Matthew Schmerer, Cyndi Clark, Patrick Campbell, Rob Case, Vikramsinha Ghorpade, Holly Houdeshell, Ola Kvalvaag, Dillon Nall, Ethan Sanders, Alec Vest, Shaun Westlund, Matthew Hardison, Clinton R. Paden, Duncan MacCannell |
| EPI_ISL_2150179 | hCoV-19/USA/FL-CDC-ASC210033984/2021 | North America/USA/Florida | 2021-04-26 | Aegis Sciences Corporation | Centers for Disease Control and Prevention Division of Viral Diseases, Pathogen Discovery | Dakota Howard, Dhwani Batra, Peter W. Cook, Kara Moser, Adrian Paskey, Jason Caravas, Benjamin Rambo-Martin, Shatavia Morrison, Christopher Gulvick, Scott Sammons, Yvette Unoarumhi, Darlene Wagner, Matthew Schmerer, Cyndi Clark, Patrick Campbell, Rob Case, Vikramsinha Ghorpade, Holly Houdeshell, Ola Kvalvaag, Dillon Nall, Ethan Sanders, Alec Vest, Shaun Westlund, Matthew Hardison, Clinton R. Paden, Duncan MacCannell |
| EPI_ISL_1690256 | hCoV-19/USA/FL-CDC-ASC210046529/2021 | North America/USA/Florida | 2021-04-04 | Aegis Sciences Corporation | Centers for Disease Control and Prevention Division of Viral Diseases, Pathogen Discovery | Dakota Howard, Dhwani Batra, Peter W. Cook, Kara Moser, Adrian Paskey, Jason Caravas, Benjamin Rambo-Martin, Shatavia Morrison, Christopher Gulvick, Scott Sammons, Yvette Unoarumhi, Darlene Wagner, Matthew Schmerer, Cyndi Clark, Patrick Campbell, Rob Case, Vikramsinha Ghorpade, Holly Houdeshell, Ola Kvalvaag, Dillon Nall, Ethan Sanders, Alec Vest, Shaun Westlund, Matthew Hardison, Clinton R. Paden, Duncan MacCannell |
| EPI_ISL_1836695 | hCoV-19/USA/FL-CDC-ASC210052084/2021 | North America/USA/Florida | 2021-04-08 | Aegis Sciences Corporation | Centers for Disease Control and Prevention Division of Viral Diseases, Pathogen Discovery | Dakota Howard, Dhwani Batra, Peter W. Cook, Kara Moser, Adrian Paskey, Jason Caravas, Benjamin Rambo-Martin, Shatavia Morrison, Christopher Gulvick, Scott Sammons, Yvette Unoarumhi, Darlene Wagner, Matthew Schmerer, Cyndi Clark, Patrick Campbell, Rob Case, Vikramsinha Ghorpade, Holly Houdeshell, Ola Kvalvaag, Dillon Nall, Ethan Sanders, Alec Vest, Shaun Westlund, Matthew Hardison, Clinton R. Paden, Duncan MacCannell |
| EPI_ISL_1836700 | hCoV-19/USA/FL-CDC-ASC210052090/2021 | North America/USA/Florida | 2021-04-08 | Aegis Sciences Corporation | Centers for Disease Control and Prevention Division of Viral Diseases, Pathogen Discovery | Dakota Howard, Dhwani Batra, Peter W. Cook, Kara Moser, Adrian Paskey, Jason Caravas, Benjamin Rambo-Martin, Shatavia Morrison, Christopher Gulvick, Scott Sammons, Yvette Unoarumhi, Darlene Wagner, Matthew Schmerer, Cyndi Clark, Patrick Campbell, Rob Case, Vikramsinha Ghorpade, Holly Houdeshell, Ola Kvalvaag, Dillon Nall, Ethan Sanders, Alec Vest, Shaun Westlund, Matthew Hardison, Clinton R. Paden, Duncan MacCannell |
| EPI_ISL_2035166 | hCoV-19/USA/FL-CDC-ASC210055936/2021 | North America/USA/Florida | 2021-04-10 | Aegis Sciences Corporation | Centers for Disease Control and Prevention Division of Viral Diseases, Pathogen Discovery | Dakota Howard, Dhwani Batra, Peter W. Cook, Kara Moser, Adrian Paskey, Jason Caravas, Benjamin Rambo-Martin, Shatavia Morrison, Christopher Gulvick, Scott Sammons, Yvette Unoarumhi, Darlene Wagner, Matthew Schmerer, Cyndi Clark, Patrick Campbell, Rob Case, Vikramsinha Ghorpade, Holly Houdeshell, Ola Kvalvaag, Dillon Nall, Ethan Sanders, Alec Vest, Shaun Westlund, Matthew Hardison, Clinton R. Paden, Duncan MacCannell |
| EPI_ISL_2147113 | hCoV-19/USA/FL-CDC-ASC210059357/2021 | North America/USA/Florida | 2021-05-05 | Aegis Sciences Corporation | Centers for Disease Control and Prevention Division of Viral Diseases, Pathogen Discovery | Dakota Howard, Dhwani Batra, Peter W. Cook, Kara Moser, Adrian Paskey, Jason Caravas, Benjamin Rambo-Martin, Shatavia Morrison, Christopher Gulvick, Scott Sammons, Yvette Unoarumhi, Darlene Wagner, Matthew Schmerer, Cyndi Clark, Patrick Campbell, Rob Case, Vikramsinha Ghorpade, Holly Houdeshell, Ola Kvalvaag, Dillon Nall, Ethan Sanders, Alec Vest, Shaun Westlund, Matthew Hardison, Clinton R. Paden, Duncan MacCannell |
| EPI_ISL_2321357 | hCoV-19/USA/FL-CDC-ASC210062593/2021 | North America/USA/Florida | 2021-05-12 | Aegis Sciences Corporation | Centers for Disease Control and Prevention Division of Viral Diseases, Pathogen Discovery | Dakota Howard, Dhwani Batra, Peter W. Cook, Kara Moser, Adrian Paskey, Jason Caravas, Benjamin Rambo-Martin, Shatavia Morrison, Christopher Gulvick, Scott Sammons, Yvette Unoarumhi, Darlene Wagner, Matthew Schmerer, Cyndi Clark, Patrick Campbell, Rob Case, Vikramsinha Ghorpade, Holly Houdeshell, Ola Kvalvaag, Dillon Nall, Ethan Sanders, Alec Vest, Shaun Westlund, Matthew Hardison, Clinton R. Paden, Duncan MacCannell |
| EPI_ISL_2280573 | hCoV-19/USA/FL-CDC-ASC210063922/2021 | North America/USA/Florida | 2021-05-13 | Aegis Sciences Corporation | Centers for Disease Control and Prevention Division of Viral Diseases, Pathogen Discovery | Dakota Howard, Dhwani Batra, Peter W. Cook, Kara Moser, Adrian Paskey, Jason Caravas, Benjamin Rambo-Martin, Shatavia Morrison, Christopher Gulvick, Scott Sammons, Yvette Unoarumhi, Darlene Wagner, Matthew Schmerer, Cyndi Clark, Patrick Campbell, Rob Case, Vikramsinha Ghorpade, Holly Houdeshell, Ola Kvalvaag, Dillon Nall, Ethan Sanders, Alec Vest, Shaun Westlund, Matthew Hardison, Clinton R. Paden, Duncan MacCannell |
| EPI_ISL_2280574 | hCoV-19/USA/FL-CDC-ASC210063923/2021 | North America/USA/Florida | 2021-05-15 | Aegis Sciences Corporation | Centers for Disease Control and Prevention Division of Viral Diseases, Pathogen Discovery | Dakota Howard, Dhwani Batra, Peter W. Cook, Kara Moser, Adrian Paskey, Jason Caravas, Benjamin Rambo-Martin, Shatavia Morrison, Christopher Gulvick, Scott Sammons, Yvette Unoarumhi, Darlene Wagner, Matthew Schmerer, Cyndi Clark, Patrick Campbell, Rob Case, Vikramsinha Ghorpade, Holly Houdeshell, Ola Kvalvaag, Dillon Nall, Ethan Sanders, Alec Vest, Shaun Westlund, Matthew Hardison, Clinton R. Paden, Duncan MacCannell |
| EPI_ISL_2280389 | hCoV-19/USA/FL-CDC-ASC210064169/2021 | North America/USA/Florida | 2021-05-15 | Aegis Sciences Corporation | Centers for Disease Control and Prevention Division of Viral Diseases, Pathogen Discovery | Dakota Howard, Dhwani Batra, Peter W. Cook, Kara Moser, Adrian Paskey, Jason Caravas, Benjamin Rambo-Martin, Shatavia Morrison, Christopher Gulvick, Scott Sammons, Yvette Unoarumhi, Darlene Wagner, Matthew Schmerer, Cyndi Clark, Patrick Campbell, Rob Case, Vikramsinha Ghorpade, Holly Houdeshell, Ola Kvalvaag, Dillon Nall, Ethan Sanders, Alec Vest, Shaun Westlund, Matthew Hardison, Clinton R. Paden, Duncan MacCannell |
| EPI_ISL_2528525 | hCoV-19/USA/FL-CDC-ASC210106427/2021 | North America/USA/Florida | 2021-06-01 | Aegis Sciences Corporation | Centers for Disease Control and Prevention Division of Viral Diseases, Pathogen Discovery | Dakota Howard, Dhwani Batra, Peter W. Cook, Kara Moser, Adrian Paskey, Jason Caravas, Benjamin Rambo-Martin, Shatavia Morrison, Christopher Gulvick, Scott Sammons, Yvette Unoarumhi, Darlene Wagner, Matthew Schmerer, Cyndi Clark, Patrick Campbell, Rob Case, Vikramsinha Ghorpade, Holly Houdeshell, Ola Kvalvaag, Dillon Nall, Ethan Sanders, Alec Vest, Shaun Westlund, Matthew Hardison, Clinton R. Paden, Duncan MacCannell |
| EPI_ISL_2528714 | hCoV-19/USA/FL-CDC-ASC210106545/2021 | North America/USA/Florida | 2021-06-01 | Aegis Sciences Corporation | Centers for Disease Control and Prevention Division of Viral Diseases, Pathogen Discovery | Dakota Howard, Dhwani Batra, Peter W. Cook, Kara Moser, Adrian Paskey, Jason Caravas, Benjamin Rambo-Martin, Shatavia Morrison, Christopher Gulvick, Scott Sammons, Yvette Unoarumhi, Darlene Wagner, Matthew Schmerer, Cyndi Clark, Patrick Campbell, Rob Case, Vikramsinha Ghorpade, Holly Houdeshell, Ola Kvalvaag, Dillon Nall, Ethan Sanders, Alec Vest, Shaun Westlund, Matthew Hardison, Clinton R. Paden, Duncan MacCannell |
| EPI_ISL_2528996 | hCoV-19/USA/FL-CDC-ASC210107109/2021 | North America/USA/Florida | 2021-06-02 | Aegis Sciences Corporation | Centers for Disease Control and Prevention Division of Viral Diseases, Pathogen Discovery | Dakota Howard, Dhwani Batra, Peter W. Cook, Kara Moser, Adrian Paskey, Jason Caravas, Benjamin Rambo-Martin, Shatavia Morrison, Christopher Gulvick, Scott Sammons, Yvette Unoarumhi, Darlene Wagner, Matthew Schmerer, Cyndi Clark, Patrick Campbell, Rob Case, Vikramsinha Ghorpade, Holly Houdeshell, Ola Kvalvaag, Dillon Nall, Ethan Sanders, Alec Vest, Shaun Westlund, Matthew Hardison, Clinton R. Paden, Duncan MacCannell |
| EPI_ISL_2686949 | hCoV-19/USA/FL-CDC-ASC210107683/2021 | North America/USA/Florida | 2021-06-04 | Aegis Sciences Corporation | Centers for Disease Control and Prevention Division of Viral Diseases, Pathogen Discovery | Dakota Howard, Dhwani Batra, Peter W. Cook, Kara Moser, Adrian Paskey, Jason Caravas, Benjamin Rambo-Martin, Shatavia Morrison, Christopher Gulvick, Scott Sammons, Yvette Unoarumhi, Darlene Wagner, Matthew Schmerer, Cyndi Clark, Patrick Campbell, Rob Case, Vikramsinha Ghorpade, Holly Houdeshell, Ola Kvalvaag, Dillon Nall, Ethan Sanders, Alec Vest, Shaun Westlund, Matthew Hardison, Clinton R. Paden, Duncan MacCannell |
| EPI_ISL_2709502 | hCoV-19/USA/FL-CDC-ASC210107684/2021 | North America/USA/Florida | 2021-06-04 | Aegis Sciences Corporation | Centers for Disease Control and Prevention Division of Viral Diseases, Pathogen Discovery | Dakota Howard, Dhwani Batra, Peter W. Cook, Kara Moser, Adrian Paskey, Jason Caravas, Benjamin Rambo-Martin, Shatavia Morrison, Christopher Gulvick, Scott Sammons, Yvette Unoarumhi, Darlene Wagner, Matthew Schmerer, Cyndi Clark, Patrick Campbell, Rob Case, Vikramsinha Ghorpade, Holly Houdeshell, Ola Kvalvaag, Dillon Nall, Ethan Sanders, Alec Vest, Shaun Westlund, Matthew Hardison, Clinton R. Paden, Duncan MacCannell |
| EPI_ISL_2785385 | hCoV-19/USA/FL-CDC-ASC210109441/2021 | North America/USA/Florida | 2021-06-14 | Aegis Sciences Corporation | Centers for Disease Control and Prevention Division of Viral Diseases, Pathogen Discovery | Dakota Howard, Dhwani Batra, Peter W. Cook, Kara Moser, Adrian Paskey, Jason Caravas, Benjamin Rambo-Martin, Shatavia Morrison, Christopher Gulvick, Scott Sammons, Yvette Unoarumhi, Darlene Wagner, Matthew Schmerer, Cyndi Clark, Patrick Campbell, Rob Case, Vikramsinha Ghorpade, Holly Houdeshell, Ola Kvalvaag, Dillon Nall, Ethan Sanders, Alec Vest, Shaun Westlund, Matthew Hardison, Clinton R. Paden, Duncan MacCannell |
| EPI_ISL_3860594 | hCoV-19/USA/FL-CDC-ASC210117168/2021 | North America/USA/Florida | 2021-08-08 | Aegis Sciences Corporation | Centers for Disease Control and Prevention Division of Viral Diseases, Pathogen Discovery | Dakota Howard,Dhwani Batra,Peter Cook,Kara Moser,Adrian Paskey,Jason Caravas,Benjamin Rambo-Martin,Shatavia Morrison,Christopher Gulvick,Scott Sammons,Yvette Unoarumhi,Darlene Wagner,Matthew Schmerer,Cyndi Clark,Patrick Campbell,Rob Case,Vikramsinha Ghorpade,Holly Houdeshell,Ola Kvalvaag,Dillon Nall,Ethan Sanders,Alec Vest,Shaun Westlund,Matthew Hardison,Clinton Paden,Duncan MacCannell |
| EPI_ISL_3819789 | hCoV-19/USA/FL-CDC-ASC210210663/2021 | North America/USA/Florida | 2021-08-02 | Aegis Sciences Corporation | Centers for Disease Control and Prevention Division of Viral Diseases, Pathogen Discovery | Dakota Howard,Dhwani Batra,Peter Cook,Kara Moser,Adrian Paskey,Jason Caravas,Benjamin Rambo-Martin,Shatavia Morrison,Christopher Gulvick,Scott Sammons,Yvette Unoarumhi,Darlene Wagner,Matthew Schmerer,Cyndi Clark,Patrick Campbell,Rob Case,Vikramsinha Ghorpade,Holly Houdeshell,Ola Kvalvaag,Dillon Nall,Ethan Sanders,Alec Vest,Shaun Westlund,Matthew Hardison,Clinton Paden,Duncan MacCannell |
| EPI_ISL_3846154 | hCoV-19/USA/FL-CDC-ASC210232940/2021 | North America/USA/Florida | 2021-07-27 | Aegis Sciences Corporation | Centers for Disease Control and Prevention Division of Viral Diseases, Pathogen Discovery | Dakota Howard,Dhwani Batra,Peter Cook,Kara Moser,Adrian Paskey,Jason Caravas,Benjamin Rambo-Martin,Shatavia Morrison,Christopher Gulvick,Scott Sammons,Yvette Unoarumhi,Darlene Wagner,Matthew Schmerer,Cyndi Clark,Patrick Campbell,Rob Case,Vikramsinha Ghorpade,Holly Houdeshell,Ola Kvalvaag,Dillon Nall,Ethan Sanders,Alec Vest,Shaun Westlund,Matthew Hardison,Clinton Paden,Duncan MacCannell |
| EPI_ISL_3846039 | hCoV-19/USA/FL-CDC-ASC210233090/2021 | North America/USA/Florida | 2021-07-28 | Aegis Sciences Corporation | Centers for Disease Control and Prevention Division of Viral Diseases, Pathogen Discovery | Dakota Howard,Dhwani Batra,Peter Cook,Kara Moser,Adrian Paskey,Jason Caravas,Benjamin Rambo-Martin,Shatavia Morrison,Christopher Gulvick,Scott Sammons,Yvette Unoarumhi,Darlene Wagner,Matthew Schmerer,Cyndi Clark,Patrick Campbell,Rob Case,Vikramsinha Ghorpade,Holly Houdeshell,Ola Kvalvaag,Dillon Nall,Ethan Sanders,Alec Vest,Shaun Westlund,Matthew Hardison,Clinton Paden,Duncan MacCannell |
| EPI_ISL_3848660 | hCoV-19/USA/FL-CDC-ASC210233743/2021 | North America/USA/Florida | 2021-07-29 | Aegis Sciences Corporation | Centers for Disease Control and Prevention Division of Viral Diseases, Pathogen Discovery | Dakota Howard,Dhwani Batra,Peter Cook,Kara Moser,Adrian Paskey,Jason Caravas,Benjamin Rambo-Martin,Shatavia Morrison,Christopher Gulvick,Scott Sammons,Yvette Unoarumhi,Darlene Wagner,Matthew Schmerer,Cyndi Clark,Patrick Campbell,Rob Case,Vikramsinha Ghorpade,Holly Houdeshell,Ola Kvalvaag,Dillon Nall,Ethan Sanders,Alec Vest,Shaun Westlund,Matthew Hardison,Clinton Paden,Duncan MacCannell |
| EPI_ISL_3849188 | hCoV-19/USA/FL-CDC-ASC210236631/2021 | North America/USA/Florida | 2021-07-30 | Aegis Sciences Corporation | Centers for Disease Control and Prevention Division of Viral Diseases, Pathogen Discovery | Dakota Howard,Dhwani Batra,Peter Cook,Kara Moser,Adrian Paskey,Jason Caravas,Benjamin Rambo-Martin,Shatavia Morrison,Christopher Gulvick,Scott Sammons,Yvette Unoarumhi,Darlene Wagner,Matthew Schmerer,Cyndi Clark,Patrick Campbell,Rob Case,Vikramsinha Ghorpade,Holly Houdeshell,Ola Kvalvaag,Dillon Nall,Ethan Sanders,Alec Vest,Shaun Westlund,Matthew Hardison,Clinton Paden,Duncan MacCannell |
| EPI_ISL_4058402 | hCoV-19/USA/FL-CDC-ASC210273210/2021 | North America/USA/Florida | 2021-08-04 | Aegis Sciences Corporation | Centers for Disease Control and Prevention Division of Viral Diseases, Pathogen Discovery | Dakota Howard,Dhwani Batra,Peter Cook,Kara Moser,Adrian Paskey,Jason Caravas,Benjamin Rambo-Martin,Shatavia Morrison,Christopher Gulvick,Scott Sammons,Yvette Unoarumhi,Darlene Wagner,Matthew Schmerer,Cyndi Clark,Patrick Campbell,Rob Case,Vikramsinha Ghorpade,Holly Houdeshell,Ola Kvalvaag,Dillon Nall,Ethan Sanders,Alec Vest,Shaun Westlund,Matthew Hardison,Clinton Paden,Duncan MacCannell |
| EPI_ISL_3861917 | hCoV-19/USA/FL-CDC-ASC210276607/2021 | North America/USA/Florida | 2021-08-05 | Aegis Sciences Corporation | Centers for Disease Control and Prevention Division of Viral Diseases, Pathogen Discovery | Dakota Howard,Dhwani Batra,Peter Cook,Kara Moser,Adrian Paskey,Jason Caravas,Benjamin Rambo-Martin,Shatavia Morrison,Christopher Gulvick,Scott Sammons,Yvette Unoarumhi,Darlene Wagner,Matthew Schmerer,Cyndi Clark,Patrick Campbell,Rob Case,Vikramsinha Ghorpade,Holly Houdeshell,Ola Kvalvaag,Dillon Nall,Ethan Sanders,Alec Vest,Shaun Westlund,Matthew Hardison,Clinton Paden,Duncan MacCannell |
| EPI_ISL_3853390 | hCoV-19/USA/GA-CDC-ASC210238086/2021 | North America/USA/Georgia | 2021-08-03 | Aegis Sciences Corporation | Centers for Disease Control and Prevention Division of Viral Diseases, Pathogen Discovery | Dakota Howard,Dhwani Batra,Peter Cook,Kara Moser,Adrian Paskey,Jason Caravas,Benjamin Rambo-Martin,Shatavia Morrison,Christopher Gulvick,Scott Sammons,Yvette Unoarumhi,Darlene Wagner,Matthew Schmerer,Cyndi Clark,Patrick Campbell,Rob Case,Vikramsinha Ghorpade,Holly Houdeshell,Ola Kvalvaag,Dillon Nall,Ethan Sanders,Alec Vest,Shaun Westlund,Matthew Hardison,Clinton Paden,Duncan MacCannell |
| EPI_ISL_4142353 | hCoV-19/USA/HI-CDC-ASC210189946/2021 | North America/USA/Hawaii | 2021-08-10 | Aegis Sciences Corporation | Centers for Disease Control and Prevention Division of Viral Diseases, Pathogen Discovery | Dakota Howard,Dhwani Batra,Peter Cook,Kara Moser,Adrian Paskey,Jason Caravas,Benjamin Rambo-Martin,Shatavia Morrison,Christopher Gulvick,Scott Sammons,Yvette Unoarumhi,Darlene Wagner,Matthew Schmerer,Cyndi Clark,Patrick Campbell,Rob Case,Vikramsinha Ghorpade,Holly Houdeshell,Ola Kvalvaag,Dillon Nall,Ethan Sanders,Alec Vest,Shaun Westlund,Matthew Hardison,Clinton Paden,Duncan MacCannell |
| EPI_ISL_1687243 | hCoV-19/USA/IL-CDC-ASC210025520/2021 | North America/USA/Illinois | 2021-03-29 | Aegis Sciences Corporation | Centers for Disease Control and Prevention Division of Viral Diseases, Pathogen Discovery | Dakota Howard, Dhwani Batra, Peter W. Cook, Kara Moser, Adrian Paskey, Jason Caravas, Benjamin Rambo-Martin, Shatavia Morrison, Christopher Gulvick, Scott Sammons, Yvette Unoarumhi, Darlene Wagner, Matthew Schmerer, Cyndi Clark, Patrick Campbell, Rob Case, Vikramsinha Ghorpade, Holly Houdeshell, Ola Kvalvaag, Dillon Nall, Ethan Sanders, Alec Vest, Shaun Westlund, Matthew Hardison, Clinton R. Paden, Duncan MacCannell |
| EPI_ISL_2150103 | hCoV-19/USA/IL-CDC-ASC210031317/2021 | North America/USA/Illinois | 2021-04-23 | Aegis Sciences Corporation | Centers for Disease Control and Prevention Division of Viral Diseases, Pathogen Discovery | Dakota Howard, Dhwani Batra, Peter W. Cook, Kara Moser, Adrian Paskey, Jason Caravas, Benjamin Rambo-Martin, Shatavia Morrison, Christopher Gulvick, Scott Sammons, Yvette Unoarumhi, Darlene Wagner, Matthew Schmerer, Cyndi Clark, Patrick Campbell, Rob Case, Vikramsinha Ghorpade, Holly Houdeshell, Ola Kvalvaag, Dillon Nall, Ethan Sanders, Alec Vest, Shaun Westlund, Matthew Hardison, Clinton R. Paden, Duncan MacCannell |
| EPI_ISL_1736664 | hCoV-19/USA/IL-CDC-ASC210047124/2021 | North America/USA/Illinois | 2021-04-05 | Aegis Sciences Corporation | Centers for Disease Control and Prevention Division of Viral Diseases, Pathogen Discovery | Dakota Howard, Dhwani Batra, Peter W. Cook, Kara Moser, Adrian Paskey, Jason Caravas, Benjamin Rambo-Martin, Shatavia Morrison, Christopher Gulvick, Scott Sammons, Yvette Unoarumhi, Darlene Wagner, Matthew Schmerer, Cyndi Clark, Patrick Campbell, Rob Case, Vikramsinha Ghorpade, Holly Houdeshell, Ola Kvalvaag, Dillon Nall, Ethan Sanders, Alec Vest, Shaun Westlund, Matthew Hardison, Clinton R. Paden, Duncan MacCannell |
| EPI_ISL_2181588 | hCoV-19/USA/IL-CDC-ASC210062165/2021 | North America/USA/Illinois | 2021-05-11 | Aegis Sciences Corporation | Centers for Disease Control and Prevention Division of Viral Diseases, Pathogen Discovery | Dakota Howard, Dhwani Batra, Peter W. Cook, Kara Moser, Adrian Paskey, Jason Caravas, Benjamin Rambo-Martin, Shatavia Morrison, Christopher Gulvick, Scott Sammons, Yvette Unoarumhi, Darlene Wagner, Matthew Schmerer, Cyndi Clark, Patrick Campbell, Rob Case, Vikramsinha Ghorpade, Holly Houdeshell, Ola Kvalvaag, Dillon Nall, Ethan Sanders, Alec Vest, Shaun Westlund, Matthew Hardison, Clinton R. Paden, Duncan MacCannell |
| EPI_ISL_2181592 | hCoV-19/USA/IL-CDC-ASC210062171/2021 | North America/USA/Illinois | 2021-05-11 | Aegis Sciences Corporation | Centers for Disease Control and Prevention Division of Viral Diseases, Pathogen Discovery | Dakota Howard, Dhwani Batra, Peter W. Cook, Kara Moser, Adrian Paskey, Jason Caravas, Benjamin Rambo-Martin, Shatavia Morrison, Christopher Gulvick, Scott Sammons, Yvette Unoarumhi, Darlene Wagner, Matthew Schmerer, Cyndi Clark, Patrick Campbell, Rob Case, Vikramsinha Ghorpade, Holly Houdeshell, Ola Kvalvaag, Dillon Nall, Ethan Sanders, Alec Vest, Shaun Westlund, Matthew Hardison, Clinton R. Paden, Duncan MacCannell |
| EPI_ISL_3821263 | hCoV-19/USA/KY-CDC-ASC210210434/2021 | North America/USA/Kentucky | 2021-08-02 | Aegis Sciences Corporation | Centers for Disease Control and Prevention Division of Viral Diseases, Pathogen Discovery | Dakota Howard,Dhwani Batra,Peter Cook,Kara Moser,Adrian Paskey,Jason Caravas,Benjamin Rambo-Martin,Shatavia Morrison,Christopher Gulvick,Scott Sammons,Yvette Unoarumhi,Darlene Wagner,Matthew Schmerer,Cyndi Clark,Patrick Campbell,Rob Case,Vikramsinha Ghorpade,Holly Houdeshell,Ola Kvalvaag,Dillon Nall,Ethan Sanders,Alec Vest,Shaun Westlund,Matthew Hardison,Clinton Paden,Duncan MacCannell |
| EPI_ISL_2043251 | hCoV-19/USA/MA-CDC-ASC210035821/2021 | North America/USA/Massachusetts | 2021-04-28 | Aegis Sciences Corporation | Centers for Disease Control and Prevention Division of Viral Diseases, Pathogen Discovery | Dakota Howard, Dhwani Batra, Peter W. Cook, Kara Moser, Adrian Paskey, Jason Caravas, Benjamin Rambo-Martin, Shatavia Morrison, Christopher Gulvick, Scott Sammons, Yvette Unoarumhi, Darlene Wagner, Matthew Schmerer, Cyndi Clark, Patrick Campbell, Rob Case, Vikramsinha Ghorpade, Holly Houdeshell, Ola Kvalvaag, Dillon Nall, Ethan Sanders, Alec Vest, Shaun Westlund, Matthew Hardison, Clinton R. Paden, Duncan MacCannell |
| EPI_ISL_2042264 | hCoV-19/USA/MA-CDC-ASC210036515/2021 | North America/USA/Massachusetts | 2021-04-29 | Aegis Sciences Corporation | Centers for Disease Control and Prevention Division of Viral Diseases, Pathogen Discovery | Dakota Howard, Dhwani Batra, Peter W. Cook, Kara Moser, Adrian Paskey, Jason Caravas, Benjamin Rambo-Martin, Shatavia Morrison, Christopher Gulvick, Scott Sammons, Yvette Unoarumhi, Darlene Wagner, Matthew Schmerer, Cyndi Clark, Patrick Campbell, Rob Case, Vikramsinha Ghorpade, Holly Houdeshell, Ola Kvalvaag, Dillon Nall, Ethan Sanders, Alec Vest, Shaun Westlund, Matthew Hardison, Clinton R. Paden, Duncan MacCannell |
| EPI_ISL_1835897 | hCoV-19/USA/MA-CDC-ASC210048547/2021 | North America/USA/Massachusetts | 2021-04-06 | Aegis Sciences Corporation | Centers for Disease Control and Prevention Division of Viral Diseases, Pathogen Discovery | Dakota Howard, Dhwani Batra, Peter W. Cook, Kara Moser, Adrian Paskey, Jason Caravas, Benjamin Rambo-Martin, Shatavia Morrison, Christopher Gulvick, Scott Sammons, Yvette Unoarumhi, Darlene Wagner, Matthew Schmerer, Cyndi Clark, Patrick Campbell, Rob Case, Vikramsinha Ghorpade, Holly Houdeshell, Ola Kvalvaag, Dillon Nall, Ethan Sanders, Alec Vest, Shaun Westlund, Matthew Hardison, Clinton R. Paden, Duncan MacCannell |
| EPI_ISL_2482367 | hCoV-19/USA/MD-CDC-ASC210021701/2021 | North America/USA/Maryland | 2021-03-26 | Aegis Sciences Corporation | Centers for Disease Control and Prevention Division of Viral Diseases, Pathogen Discovery | Dakota Howard, Dhwani Batra, Peter W. Cook, Kara Moser, Adrian Paskey, Jason Caravas, Benjamin Rambo-Martin, Shatavia Morrison, Christopher Gulvick, Scott Sammons, Yvette Unoarumhi, Darlene Wagner, Matthew Schmerer, Cyndi Clark, Patrick Campbell, Rob Case, Vikramsinha Ghorpade, Holly Houdeshell, Ola Kvalvaag, Dillon Nall, Ethan Sanders, Alec Vest, Shaun Westlund, Matthew Hardison, Clinton R. Paden, Duncan MacCannell |
| EPI_ISL_3860578 | hCoV-19/USA/MD-CDC-ASC210117144/2021 | North America/USA/Maryland | 2021-08-07 | Aegis Sciences Corporation | Centers for Disease Control and Prevention Division of Viral Diseases, Pathogen Discovery | Dakota Howard,Dhwani Batra,Peter Cook,Kara Moser,Adrian Paskey,Jason Caravas,Benjamin Rambo-Martin,Shatavia Morrison,Christopher Gulvick,Scott Sammons,Yvette Unoarumhi,Darlene Wagner,Matthew Schmerer,Cyndi Clark,Patrick Campbell,Rob Case,Vikramsinha Ghorpade,Holly Houdeshell,Ola Kvalvaag,Dillon Nall,Ethan Sanders,Alec Vest,Shaun Westlund,Matthew Hardison,Clinton Paden,Duncan MacCannell |
| EPI_ISL_2528089 | hCoV-19/USA/NC-CDC-ASC210106991/2021 | North America/USA/North Carolina | 2021-06-02 | Aegis Sciences Corporation | Centers for Disease Control and Prevention Division of Viral Diseases, Pathogen Discovery | Dakota Howard, Dhwani Batra, Peter W. Cook, Kara Moser, Adrian Paskey, Jason Caravas, Benjamin Rambo-Martin, Shatavia Morrison, Christopher Gulvick, Scott Sammons, Yvette Unoarumhi, Darlene Wagner, Matthew Schmerer, Cyndi Clark, Patrick Campbell, Rob Case, Vikramsinha Ghorpade, Holly Houdeshell, Ola Kvalvaag, Dillon Nall, Ethan Sanders, Alec Vest, Shaun Westlund, Matthew Hardison, Clinton R. Paden, Duncan MacCannell |
| EPI_ISL_2149537 | hCoV-19/USA/NJ-CDC-ASC210028890/2021 | North America/USA/New Jersey | 2021-04-20 | Aegis Sciences Corporation | Centers for Disease Control and Prevention Division of Viral Diseases, Pathogen Discovery | Dakota Howard, Dhwani Batra, Peter W. Cook, Kara Moser, Adrian Paskey, Jason Caravas, Benjamin Rambo-Martin, Shatavia Morrison, Christopher Gulvick, Scott Sammons, Yvette Unoarumhi, Darlene Wagner, Matthew Schmerer, Cyndi Clark, Patrick Campbell, Rob Case, Vikramsinha Ghorpade, Holly Houdeshell, Ola Kvalvaag, Dillon Nall, Ethan Sanders, Alec Vest, Shaun Westlund, Matthew Hardison, Clinton R. Paden, Duncan MacCannell |
| EPI_ISL_3849764 | hCoV-19/USA/OH-CDC-ASC210202224/2021 | North America/USA/Ohio | 2021-07-30 | Aegis Sciences Corporation | Centers for Disease Control and Prevention Division of Viral Diseases, Pathogen Discovery | Dakota Howard,Dhwani Batra,Peter Cook,Kara Moser,Adrian Paskey,Jason Caravas,Benjamin Rambo-Martin,Shatavia Morrison,Christopher Gulvick,Scott Sammons,Yvette Unoarumhi,Darlene Wagner,Matthew Schmerer,Cyndi Clark,Patrick Campbell,Rob Case,Vikramsinha Ghorpade,Holly Houdeshell,Ola Kvalvaag,Dillon Nall,Ethan Sanders,Alec Vest,Shaun Westlund,Matthew Hardison,Clinton Paden,Duncan MacCannell |
| EPI_ISL_3818751 | hCoV-19/USA/OH-CDC-ASC210205192/2021 | North America/USA/Ohio | 2021-08-01 | Aegis Sciences Corporation | Centers for Disease Control and Prevention Division of Viral Diseases, Pathogen Discovery | Dakota Howard,Dhwani Batra,Peter Cook,Kara Moser,Adrian Paskey,Jason Caravas,Benjamin Rambo-Martin,Shatavia Morrison,Christopher Gulvick,Scott Sammons,Yvette Unoarumhi,Darlene Wagner,Matthew Schmerer,Cyndi Clark,Patrick Campbell,Rob Case,Vikramsinha Ghorpade,Holly Houdeshell,Ola Kvalvaag,Dillon Nall,Ethan Sanders,Alec Vest,Shaun Westlund,Matthew Hardison,Clinton Paden,Duncan MacCannell |
| EPI_ISL_2528173 | hCoV-19/USA/OR-CDC-ASC210107523/2021 | North America/USA/Oregon | 2021-06-04 | Aegis Sciences Corporation | Centers for Disease Control and Prevention Division of Viral Diseases, Pathogen Discovery | Dakota Howard, Dhwani Batra, Peter W. Cook, Kara Moser, Adrian Paskey, Jason Caravas, Benjamin Rambo-Martin, Shatavia Morrison, Christopher Gulvick, Scott Sammons, Yvette Unoarumhi, Darlene Wagner, Matthew Schmerer, Cyndi Clark, Patrick Campbell, Rob Case, Vikramsinha Ghorpade, Holly Houdeshell, Ola Kvalvaag, Dillon Nall, Ethan Sanders, Alec Vest, Shaun Westlund, Matthew Hardison, Clinton R. Paden, Duncan MacCannell |
| EPI_ISL_2244097 | hCoV-19/USA/PA-CDC-ASC210003869/2021 | North America/USA/Pennsylvania | 2021-03-11 | Aegis Sciences Corporation | Centers for Disease Control and Prevention Division of Viral Diseases, Pathogen Discovery | Dakota Howard, Dhwani Batra, Peter W. Cook, Kara Moser, Adrian Paskey, Jason Caravas, Benjamin Rambo-Martin, Shatavia Morrison, Christopher Gulvick, Scott Sammons, Yvette Unoarumhi, Darlene Wagner, Matthew Schmerer, Cyndi Clark, Patrick Campbell, Rob Case, Vikramsinha Ghorpade, Holly Houdeshell, Ola Kvalvaag, Dillon Nall, Ethan Sanders, Alec Vest, Shaun Westlund, Matthew Hardison, Clinton R. Paden, Duncan MacCannell |
| EPI_ISL_2149818 | hCoV-19/USA/PA-CDC-ASC210030710/2021 | North America/USA/Pennsylvania | 2021-04-22 | Aegis Sciences Corporation | Centers for Disease Control and Prevention Division of Viral Diseases, Pathogen Discovery | Dakota Howard, Dhwani Batra, Peter W. Cook, Kara Moser, Adrian Paskey, Jason Caravas, Benjamin Rambo-Martin, Shatavia Morrison, Christopher Gulvick, Scott Sammons, Yvette Unoarumhi, Darlene Wagner, Matthew Schmerer, Cyndi Clark, Patrick Campbell, Rob Case, Vikramsinha Ghorpade, Holly Houdeshell, Ola Kvalvaag, Dillon Nall, Ethan Sanders, Alec Vest, Shaun Westlund, Matthew Hardison, Clinton R. Paden, Duncan MacCannell |
| EPI_ISL_1995821 | hCoV-19/USA/PA-CDC-ASC210056023/2021 | North America/USA/Pennsylvania | 2021-04-10 | Aegis Sciences Corporation | Centers for Disease Control and Prevention Division of Viral Diseases, Pathogen Discovery | Dakota Howard, Dhwani Batra, Peter W. Cook, Kara Moser, Adrian Paskey, Jason Caravas, Benjamin Rambo-Martin, Shatavia Morrison, Christopher Gulvick, Scott Sammons, Yvette Unoarumhi, Darlene Wagner, Matthew Schmerer, Cyndi Clark, Patrick Campbell, Rob Case, Vikramsinha Ghorpade, Holly Houdeshell, Ola Kvalvaag, Dillon Nall, Ethan Sanders, Alec Vest, Shaun Westlund, Matthew Hardison, Clinton R. Paden, Duncan MacCannell |
| EPI_ISL_2281066 | hCoV-19/USA/PA-CDC-ASC210064349/2021 | North America/USA/Pennsylvania | 2021-05-17 | Aegis Sciences Corporation | Centers for Disease Control and Prevention Division of Viral Diseases, Pathogen Discovery | Dakota Howard, Dhwani Batra, Peter W. Cook, Kara Moser, Adrian Paskey, Jason Caravas, Benjamin Rambo-Martin, Shatavia Morrison, Christopher Gulvick, Scott Sammons, Yvette Unoarumhi, Darlene Wagner, Matthew Schmerer, Cyndi Clark, Patrick Campbell, Rob Case, Vikramsinha Ghorpade, Holly Houdeshell, Ola Kvalvaag, Dillon Nall, Ethan Sanders, Alec Vest, Shaun Westlund, Matthew Hardison, Clinton R. Paden, Duncan MacCannell |
| EPI_ISL_4186702 | hCoV-19/USA/PA-CDC-ASC210242285/2021 | North America/USA/Pennsylvania | 2021-07-23 | Aegis Sciences Corporation | Centers for Disease Control and Prevention Division of Viral Diseases, Pathogen Discovery | Dakota Howard,Dhwani Batra,Peter Cook,Jason Caravas,Benjamin Rambo-Martin,Scott Sammons,Yvette Unoarumhi,Matthew Schmerer,Kristine Lacek,Tymeckia Kendall,Victoria Caban Figueroa,Shatavia Morrison,Christopher Gulvick,Erisa Sula,Cyndi Clark,Patrick Campbell,Rob Case,Vikramsinha Ghorpade,Holly Houdeshell,Ola Kvalvaag,Dillon Nall,Ethan Sanders,Alec Vest,Shaun Westlund,Matthew Hardison,Clinton Paden,Duncan MacCannell |
| EPI_ISL_1836347 | hCoV-19/USA/PR-CDC-ASC210053760/2021 | North America/Puerto Rico | 2021-04-09 | Aegis Sciences Corporation | Centers for Disease Control and Prevention Division of Viral Diseases, Pathogen Discovery | Dakota Howard, Dhwani Batra, Peter W. Cook, Kara Moser, Adrian Paskey, Jason Caravas, Benjamin Rambo-Martin, Shatavia Morrison, Christopher Gulvick, Scott Sammons, Yvette Unoarumhi, Darlene Wagner, Matthew Schmerer, Cyndi Clark, Patrick Campbell, Rob Case, Vikramsinha Ghorpade, Holly Houdeshell, Ola Kvalvaag, Dillon Nall, Ethan Sanders, Alec Vest, Shaun Westlund, Matthew Hardison, Clinton R. Paden, Duncan MacCannell |
| EPI_ISL_2528851 | hCoV-19/USA/PR-CDC-ASC210107208/2021 | North America/Puerto Rico | 2021-06-03 | Aegis Sciences Corporation | Centers for Disease Control and Prevention Division of Viral Diseases, Pathogen Discovery | Dakota Howard, Dhwani Batra, Peter W. Cook, Kara Moser, Adrian Paskey, Jason Caravas, Benjamin Rambo-Martin, Shatavia Morrison, Christopher Gulvick, Scott Sammons, Yvette Unoarumhi, Darlene Wagner, Matthew Schmerer, Cyndi Clark, Patrick Campbell, Rob Case, Vikramsinha Ghorpade, Holly Houdeshell, Ola Kvalvaag, Dillon Nall, Ethan Sanders, Alec Vest, Shaun Westlund, Matthew Hardison, Clinton R. Paden, Duncan MacCannell |
| EPI_ISL_3861119 | hCoV-19/USA/PR-CDC-ASC210116877/2021 | North America/Puerto Rico | 2021-08-06 | Aegis Sciences Corporation | Centers for Disease Control and Prevention Division of Viral Diseases, Pathogen Discovery | Dakota Howard,Dhwani Batra,Peter Cook,Kara Moser,Adrian Paskey,Jason Caravas,Benjamin Rambo-Martin,Shatavia Morrison,Christopher Gulvick,Scott Sammons,Yvette Unoarumhi,Darlene Wagner,Matthew Schmerer,Cyndi Clark,Patrick Campbell,Rob Case,Vikramsinha Ghorpade,Holly Houdeshell,Ola Kvalvaag,Dillon Nall,Ethan Sanders,Alec Vest,Shaun Westlund,Matthew Hardison,Clinton Paden,Duncan MacCannell |
| EPI_ISL_3220712 | hCoV-19/USA/PR-CDC-ASC210154600/2021 | North America/Puerto Rico | 2021-06-28 | Aegis Sciences Corporation | Centers for Disease Control and Prevention Division of Viral Diseases, Pathogen Discovery | Dakota Howard, Dhwani Batra, Peter W. Cook, Kara Moser, Adrian Paskey, Jason Caravas, Benjamin Rambo-Martin, Shatavia Morrison, Christopher Gulvick, Scott Sammons, Yvette Unoarumhi, Darlene Wagner, Matthew Schmerer, Cyndi Clark, Patrick Campbell, Rob Case, Vikramsinha Ghorpade, Holly Houdeshell, Ola Kvalvaag, Dillon Nall, Ethan Sanders, Alec Vest, Shaun Westlund, Matthew Hardison, Clinton R. Paden, Duncan MacCannell |
| EPI_ISL_3220571 | hCoV-19/USA/PR-CDC-ASC210154652/2021 | North America/Puerto Rico | 2021-06-28 | Aegis Sciences Corporation | Centers for Disease Control and Prevention Division of Viral Diseases, Pathogen Discovery | Dakota Howard, Dhwani Batra, Peter W. Cook, Kara Moser, Adrian Paskey, Jason Caravas, Benjamin Rambo-Martin, Shatavia Morrison, Christopher Gulvick, Scott Sammons, Yvette Unoarumhi, Darlene Wagner, Matthew Schmerer, Cyndi Clark, Patrick Campbell, Rob Case, Vikramsinha Ghorpade, Holly Houdeshell, Ola Kvalvaag, Dillon Nall, Ethan Sanders, Alec Vest, Shaun Westlund, Matthew Hardison, Clinton R. Paden, Duncan MacCannell |
| EPI_ISL_3220572 | hCoV-19/USA/PR-CDC-ASC210154653/2021 | North America/Puerto Rico | 2021-06-28 | Aegis Sciences Corporation | Centers for Disease Control and Prevention Division of Viral Diseases, Pathogen Discovery | Dakota Howard, Dhwani Batra, Peter W. Cook, Kara Moser, Adrian Paskey, Jason Caravas, Benjamin Rambo-Martin, Shatavia Morrison, Christopher Gulvick, Scott Sammons, Yvette Unoarumhi, Darlene Wagner, Matthew Schmerer, Cyndi Clark, Patrick Campbell, Rob Case, Vikramsinha Ghorpade, Holly Houdeshell, Ola Kvalvaag, Dillon Nall, Ethan Sanders, Alec Vest, Shaun Westlund, Matthew Hardison, Clinton R. Paden, Duncan MacCannell |
| EPI_ISL_3220575 | hCoV-19/USA/PR-CDC-ASC210154656/2021 | North America/Puerto Rico | 2021-06-28 | Aegis Sciences Corporation | Centers for Disease Control and Prevention Division of Viral Diseases, Pathogen Discovery | Dakota Howard, Dhwani Batra, Peter W. Cook, Kara Moser, Adrian Paskey, Jason Caravas, Benjamin Rambo-Martin, Shatavia Morrison, Christopher Gulvick, Scott Sammons, Yvette Unoarumhi, Darlene Wagner, Matthew Schmerer, Cyndi Clark, Patrick Campbell, Rob Case, Vikramsinha Ghorpade, Holly Houdeshell, Ola Kvalvaag, Dillon Nall, Ethan Sanders, Alec Vest, Shaun Westlund, Matthew Hardison, Clinton R. Paden, Duncan MacCannell |
| EPI_ISL_3324486 | hCoV-19/USA/PR-CDC-ASC210159107/2021 | North America/Puerto Rico | 2021-07-16 | Aegis Sciences Corporation | Centers for Disease Control and Prevention Division of Viral Diseases, Pathogen Discovery | Dakota Howard, Dhwani Batra, Peter W. Cook, Kara Moser, Adrian Paskey, Jason Caravas, Benjamin Rambo-Martin, Shatavia Morrison, Christopher Gulvick, Scott Sammons, Yvette Unoarumhi, Darlene Wagner, Matthew Schmerer, Cyndi Clark, Patrick Campbell, Rob Case, Vikramsinha Ghorpade, Holly Houdeshell, Ola Kvalvaag, Dillon Nall, Ethan Sanders, Alec Vest, Shaun Westlund, Matthew Hardison, Clinton R. Paden, Duncan MacCannell |
| EPI_ISL_3324489 | hCoV-19/USA/PR-CDC-ASC210159112/2021 | North America/Puerto Rico | 2021-07-16 | Aegis Sciences Corporation | Centers for Disease Control and Prevention Division of Viral Diseases, Pathogen Discovery | Dakota Howard, Dhwani Batra, Peter W. Cook, Kara Moser, Adrian Paskey, Jason Caravas, Benjamin Rambo-Martin, Shatavia Morrison, Christopher Gulvick, Scott Sammons, Yvette Unoarumhi, Darlene Wagner, Matthew Schmerer, Cyndi Clark, Patrick Campbell, Rob Case, Vikramsinha Ghorpade, Holly Houdeshell, Ola Kvalvaag, Dillon Nall, Ethan Sanders, Alec Vest, Shaun Westlund, Matthew Hardison, Clinton R. Paden, Duncan MacCannell |
| EPI_ISL_3323601 | hCoV-19/USA/PR-CDC-ASC210159537/2021 | North America/Puerto Rico | 2021-07-19 | Aegis Sciences Corporation | Centers for Disease Control and Prevention Division of Viral Diseases, Pathogen Discovery | Dakota Howard, Dhwani Batra, Peter W. Cook, Kara Moser, Adrian Paskey, Jason Caravas, Benjamin Rambo-Martin, Shatavia Morrison, Christopher Gulvick, Scott Sammons, Yvette Unoarumhi, Darlene Wagner, Matthew Schmerer, Cyndi Clark, Patrick Campbell, Rob Case, Vikramsinha Ghorpade, Holly Houdeshell, Ola Kvalvaag, Dillon Nall, Ethan Sanders, Alec Vest, Shaun Westlund, Matthew Hardison, Clinton R. Paden, Duncan MacCannell |
| EPI_ISL_3325369 | hCoV-19/USA/PR-CDC-ASC210160098/2021 | North America/Puerto Rico | 2021-07-19 | Aegis Sciences Corporation | Centers for Disease Control and Prevention Division of Viral Diseases, Pathogen Discovery | Dakota Howard, Dhwani Batra, Peter W. Cook, Kara Moser, Adrian Paskey, Jason Caravas, Benjamin Rambo-Martin, Shatavia Morrison, Christopher Gulvick, Scott Sammons, Yvette Unoarumhi, Darlene Wagner, Matthew Schmerer, Cyndi Clark, Patrick Campbell, Rob Case, Vikramsinha Ghorpade, Holly Houdeshell, Ola Kvalvaag, Dillon Nall, Ethan Sanders, Alec Vest, Shaun Westlund, Matthew Hardison, Clinton R. Paden, Duncan MacCannell |
| EPI_ISL_3325112 | hCoV-19/USA/PR-CDC-ASC210160404/2021 | North America/Puerto Rico | 2021-07-20 | Aegis Sciences Corporation | Centers for Disease Control and Prevention Division of Viral Diseases, Pathogen Discovery | Dakota Howard, Dhwani Batra, Peter W. Cook, Kara Moser, Adrian Paskey, Jason Caravas, Benjamin Rambo-Martin, Shatavia Morrison, Christopher Gulvick, Scott Sammons, Yvette Unoarumhi, Darlene Wagner, Matthew Schmerer, Cyndi Clark, Patrick Campbell, Rob Case, Vikramsinha Ghorpade, Holly Houdeshell, Ola Kvalvaag, Dillon Nall, Ethan Sanders, Alec Vest, Shaun Westlund, Matthew Hardison, Clinton R. Paden, Duncan MacCannell |
| EPI_ISL_3816758 | hCoV-19/USA/PR-CDC-ASC210162566/2021 | North America/Puerto Rico | 2021-07-21 | Aegis Sciences Corporation | Centers for Disease Control and Prevention Division of Viral Diseases, Pathogen Discovery | Dakota Howard,Dhwani Batra,Peter Cook,Kara Moser,Adrian Paskey,Jason Caravas,Benjamin Rambo-Martin,Shatavia Morrison,Christopher Gulvick,Scott Sammons,Yvette Unoarumhi,Darlene Wagner,Matthew Schmerer,Cyndi Clark,Patrick Campbell,Rob Case,Vikramsinha Ghorpade,Holly Houdeshell,Ola Kvalvaag,Dillon Nall,Ethan Sanders,Alec Vest,Shaun Westlund,Matthew Hardison,Clinton Paden,Duncan MacCannell |
| EPI_ISL_4140982 | hCoV-19/USA/PR-CDC-ASC210194561/2021 | North America/Puerto Rico | 2021-08-20 | Aegis Sciences Corporation | Centers for Disease Control and Prevention Division of Viral Diseases, Pathogen Discovery | Dakota Howard,Dhwani Batra,Peter Cook,Kara Moser,Adrian Paskey,Jason Caravas,Benjamin Rambo-Martin,Shatavia Morrison,Christopher Gulvick,Scott Sammons,Yvette Unoarumhi,Darlene Wagner,Matthew Schmerer,Cyndi Clark,Patrick Campbell,Rob Case,Vikramsinha Ghorpade,Holly Houdeshell,Ola Kvalvaag,Dillon Nall,Ethan Sanders,Alec Vest,Shaun Westlund,Matthew Hardison,Clinton Paden,Duncan MacCannell |
| EPI_ISL_3822951 | hCoV-19/USA/PR-CDC-ASC210208406/2021 | North America/Puerto Rico | 2021-08-02 | Aegis Sciences Corporation | Centers for Disease Control and Prevention Division of Viral Diseases, Pathogen Discovery | Dakota Howard,Dhwani Batra,Peter Cook,Kara Moser,Adrian Paskey,Jason Caravas,Benjamin Rambo-Martin,Shatavia Morrison,Christopher Gulvick,Scott Sammons,Yvette Unoarumhi,Darlene Wagner,Matthew Schmerer,Cyndi Clark,Patrick Campbell,Rob Case,Vikramsinha Ghorpade,Holly Houdeshell,Ola Kvalvaag,Dillon Nall,Ethan Sanders,Alec Vest,Shaun Westlund,Matthew Hardison,Clinton Paden,Duncan MacCannell |
| EPI_ISL_3818926 | hCoV-19/USA/PR-CDC-ASC210209428/2021 | North America/Puerto Rico | 2021-08-02 | Aegis Sciences Corporation | Centers for Disease Control and Prevention Division of Viral Diseases, Pathogen Discovery | Dakota Howard,Dhwani Batra,Peter Cook,Kara Moser,Adrian Paskey,Jason Caravas,Benjamin Rambo-Martin,Shatavia Morrison,Christopher Gulvick,Scott Sammons,Yvette Unoarumhi,Darlene Wagner,Matthew Schmerer,Cyndi Clark,Patrick Campbell,Rob Case,Vikramsinha Ghorpade,Holly Houdeshell,Ola Kvalvaag,Dillon Nall,Ethan Sanders,Alec Vest,Shaun Westlund,Matthew Hardison,Clinton Paden,Duncan MacCannell |
| EPI_ISL_3844062 | hCoV-19/USA/PR-CDC-ASC210231812/2021 | North America/Puerto Rico | 2021-07-27 | Aegis Sciences Corporation | Centers for Disease Control and Prevention Division of Viral Diseases, Pathogen Discovery | Dakota Howard,Dhwani Batra,Peter Cook,Kara Moser,Adrian Paskey,Jason Caravas,Benjamin Rambo-Martin,Shatavia Morrison,Christopher Gulvick,Scott Sammons,Yvette Unoarumhi,Darlene Wagner,Matthew Schmerer,Cyndi Clark,Patrick Campbell,Rob Case,Vikramsinha Ghorpade,Holly Houdeshell,Ola Kvalvaag,Dillon Nall,Ethan Sanders,Alec Vest,Shaun Westlund,Matthew Hardison,Clinton Paden,Duncan MacCannell |
| EPI_ISL_3846242 | hCoV-19/USA/PR-CDC-ASC210234725/2021 | North America/Puerto Rico | 2021-07-29 | Aegis Sciences Corporation | Centers for Disease Control and Prevention Division of Viral Diseases, Pathogen Discovery | Dakota Howard,Dhwani Batra,Peter Cook,Kara Moser,Adrian Paskey,Jason Caravas,Benjamin Rambo-Martin,Shatavia Morrison,Christopher Gulvick,Scott Sammons,Yvette Unoarumhi,Darlene Wagner,Matthew Schmerer,Cyndi Clark,Patrick Campbell,Rob Case,Vikramsinha Ghorpade,Holly Houdeshell,Ola Kvalvaag,Dillon Nall,Ethan Sanders,Alec Vest,Shaun Westlund,Matthew Hardison,Clinton Paden,Duncan MacCannell |
| EPI_ISL_3851093 | hCoV-19/USA/PR-CDC-ASC210236768/2021 | North America/Puerto Rico | 2021-07-29 | Aegis Sciences Corporation | Centers for Disease Control and Prevention Division of Viral Diseases, Pathogen Discovery | Dakota Howard,Dhwani Batra,Peter Cook,Kara Moser,Adrian Paskey,Jason Caravas,Benjamin Rambo-Martin,Shatavia Morrison,Christopher Gulvick,Scott Sammons,Yvette Unoarumhi,Darlene Wagner,Matthew Schmerer,Cyndi Clark,Patrick Campbell,Rob Case,Vikramsinha Ghorpade,Holly Houdeshell,Ola Kvalvaag,Dillon Nall,Ethan Sanders,Alec Vest,Shaun Westlund,Matthew Hardison,Clinton Paden,Duncan MacCannell |
| EPI_ISL_3816393 | hCoV-19/USA/PR-CDC-ASC210242887/2021 | North America/Puerto Rico | 2021-07-23 | Aegis Sciences Corporation | Centers for Disease Control and Prevention Division of Viral Diseases, Pathogen Discovery | Dakota Howard,Dhwani Batra,Peter Cook,Kara Moser,Adrian Paskey,Jason Caravas,Benjamin Rambo-Martin,Shatavia Morrison,Christopher Gulvick,Scott Sammons,Yvette Unoarumhi,Darlene Wagner,Matthew Schmerer,Cyndi Clark,Patrick Campbell,Rob Case,Vikramsinha Ghorpade,Holly Houdeshell,Ola Kvalvaag,Dillon Nall,Ethan Sanders,Alec Vest,Shaun Westlund,Matthew Hardison,Clinton Paden,Duncan MacCannell |
| EPI_ISL_3817970 | hCoV-19/USA/PR-CDC-ASC210245446/2021 | North America/Puerto Rico | 2021-07-26 | Aegis Sciences Corporation | Centers for Disease Control and Prevention Division of Viral Diseases, Pathogen Discovery | Dakota Howard,Dhwani Batra,Peter Cook,Kara Moser,Adrian Paskey,Jason Caravas,Benjamin Rambo-Martin,Shatavia Morrison,Christopher Gulvick,Scott Sammons,Yvette Unoarumhi,Darlene Wagner,Matthew Schmerer,Cyndi Clark,Patrick Campbell,Rob Case,Vikramsinha Ghorpade,Holly Houdeshell,Ola Kvalvaag,Dillon Nall,Ethan Sanders,Alec Vest,Shaun Westlund,Matthew Hardison,Clinton Paden,Duncan MacCannell |
| EPI_ISL_3815798 | hCoV-19/USA/PR-CDC-ASC210246201/2021 | North America/Puerto Rico | 2021-07-26 | Aegis Sciences Corporation | Centers for Disease Control and Prevention Division of Viral Diseases, Pathogen Discovery | Dakota Howard,Dhwani Batra,Peter Cook,Kara Moser,Adrian Paskey,Jason Caravas,Benjamin Rambo-Martin,Shatavia Morrison,Christopher Gulvick,Scott Sammons,Yvette Unoarumhi,Darlene Wagner,Matthew Schmerer,Cyndi Clark,Patrick Campbell,Rob Case,Vikramsinha Ghorpade,Holly Houdeshell,Ola Kvalvaag,Dillon Nall,Ethan Sanders,Alec Vest,Shaun Westlund,Matthew Hardison,Clinton Paden,Duncan MacCannell |
| EPI_ISL_2180667 | hCoV-19/USA/SC-CDC-ASC210061514/2021 | North America/USA/South Carolina | 2021-05-08 | Aegis Sciences Corporation | Centers for Disease Control and Prevention Division of Viral Diseases, Pathogen Discovery | Dakota Howard, Dhwani Batra, Peter W. Cook, Kara Moser, Adrian Paskey, Jason Caravas, Benjamin Rambo-Martin, Shatavia Morrison, Christopher Gulvick, Scott Sammons, Yvette Unoarumhi, Darlene Wagner, Matthew Schmerer, Cyndi Clark, Patrick Campbell, Rob Case, Vikramsinha Ghorpade, Holly Houdeshell, Ola Kvalvaag, Dillon Nall, Ethan Sanders, Alec Vest, Shaun Westlund, Matthew Hardison, Clinton R. Paden, Duncan MacCannell |
| EPI_ISL_2241667 | hCoV-19/USA/TN-CDC-ASC210061892/2021 | North America/USA/Tennessee | 2021-05-11 | Aegis Sciences Corporation | Centers for Disease Control and Prevention Division of Viral Diseases, Pathogen Discovery | Dakota Howard, Dhwani Batra, Peter W. Cook, Kara Moser, Adrian Paskey, Jason Caravas, Benjamin Rambo-Martin, Shatavia Morrison, Christopher Gulvick, Scott Sammons, Yvette Unoarumhi, Darlene Wagner, Matthew Schmerer, Cyndi Clark, Patrick Campbell, Rob Case, Vikramsinha Ghorpade, Holly Houdeshell, Ola Kvalvaag, Dillon Nall, Ethan Sanders, Alec Vest, Shaun Westlund, Matthew Hardison, Clinton R. Paden, Duncan MacCannell |
| EPI_ISL_4056730 | hCoV-19/USA/TX-CDC-ASC210275625/2021 | North America/USA/Texas | 2021-08-05 | Aegis Sciences Corporation | Centers for Disease Control and Prevention Division of Viral Diseases, Pathogen Discovery | Dakota Howard,Dhwani Batra,Peter Cook,Kara Moser,Adrian Paskey,Jason Caravas,Benjamin Rambo-Martin,Shatavia Morrison,Christopher Gulvick,Scott Sammons,Yvette Unoarumhi,Darlene Wagner,Matthew Schmerer,Cyndi Clark,Patrick Campbell,Rob Case,Vikramsinha Ghorpade,Holly Houdeshell,Ola Kvalvaag,Dillon Nall,Ethan Sanders,Alec Vest,Shaun Westlund,Matthew Hardison,Clinton Paden,Duncan MacCannell |
| EPI_ISL_2149968 | hCoV-19/USA/VA-CDC-ASC210029701/2021 | North America/USA/Virginia | 2021-04-20 | Aegis Sciences Corporation | Centers for Disease Control and Prevention Division of Viral Diseases, Pathogen Discovery | Dakota Howard, Dhwani Batra, Peter W. Cook, Kara Moser, Adrian Paskey, Jason Caravas, Benjamin Rambo-Martin, Shatavia Morrison, Christopher Gulvick, Scott Sammons, Yvette Unoarumhi, Darlene Wagner, Matthew Schmerer, Cyndi Clark, Patrick Campbell, Rob Case, Vikramsinha Ghorpade, Holly Houdeshell, Ola Kvalvaag, Dillon Nall, Ethan Sanders, Alec Vest, Shaun Westlund, Matthew Hardison, Clinton R. Paden, Duncan MacCannell |
| EPI_ISL_2042432 | hCoV-19/USA/WV-CDC-ASC210074083/2021 | North America/USA/West Virginia | 2021-04-18 | Aegis Sciences Corporation | Centers for Disease Control and Prevention Division of Viral Diseases, Pathogen Discovery | Dakota Howard, Dhwani Batra, Peter W. Cook, Kara Moser, Adrian Paskey, Jason Caravas, Benjamin Rambo-Martin, Shatavia Morrison, Christopher Gulvick, Scott Sammons, Yvette Unoarumhi, Darlene Wagner, Matthew Schmerer, Cyndi Clark, Patrick Campbell, Rob Case, Vikramsinha Ghorpade, Holly Houdeshell, Ola Kvalvaag, Dillon Nall, Ethan Sanders, Alec Vest, Shaun Westlund, Matthew Hardison, Clinton R. Paden, Duncan MacCannell |
| EPI_ISL_1201885 | hCoV-19/Brazil/SP-GRU-1467/2021 | South America/Brazil/Sao Paulo | 2021-01-21 | Aeroporto Internacional de Guarulhos | Instituto Adolfo Lutz, Interdiciplinary Procedures Center, Strategic Laboratory | Claudio Tavares Sacchi, Claudia Regina GonÃ§alves, Erica Valessa Ramos Gomes, Karoline Rodrigues Campos, Caio Vinicius Dias Lopes |
| EPI_ISL_4274119 | hCoV-19/Norway/18713/2021 | Europe/Norway/Viken | 2021-09-03 | Akershus University Hospital, Department for Microbiology and Infectious Disease Control | Norwegian Institute of Public Health, Department of Virology | Kathrine Stene-Johansen, Kamilla Heddeland Instefjord, Hilde Elshaug, Garcia Llorente Ignacio, Jon BrÃ¥te, Engebretsen Serina Beate,Pedersen Benedikte Nevjen, Line Victoria Moen, Debech Nadia, Atiya R Ali,Marie Paulsen Madsen, Rasmus Riis Kopperud, Hilde Vollan, Karoline Bragstad, Olav Hungnes |
| EPI_ISL_2333570 | hCoV-19/Norway/5864/2020 | Europe/Norway/Oslo | 2020-11-21 | Akershus University Hospital, Department for Microbiology and Infectious Disease Control | Norwegian Institute of Public Health, Department of Virology | 'Kathrine Stene-Johansen, Kamilla Heddeland Instefjord, Hilde Elshaug, Garcia Llorente Ignacio, Jon BrÃ¥te, Engebretsen Serina Beate,Pedersen Benedikte Nevjen, Debech Nadia, Line Victoria Moen, Atiya R Ali,Marie Paulsen Madsen, Rasmus Riis Kopperud, Hilde Vollan, Karoline Bragstad, Olav Hungnes |
| EPI_ISL_468359 | hCoV-19/USA/CA-CZB-1334/2020 | North America/USA/California | 2020-04-01 | Alameda County Public Health Lab | Chan-Zuckerberg Biohub | CZB Cliahub Consortium |
| EPI_ISL_468367 | hCoV-19/USA/CA-CZB-1353/2020 | North America/USA/California | 2020-04-11 | Alameda County Public Health Lab | Chan-Zuckerberg Biohub | CZB Cliahub Consortium |
| EPI_ISL_468369 | hCoV-19/USA/CA-CZB-1355/2020 | North America/USA/California | 2020-04-11 | Alameda County Public Health Lab | Chan-Zuckerberg Biohub | CZB Cliahub Consortium |
| EPI_ISL_1234829 | hCoV-19/USA/CA-CZB-25238/2020 | North America/USA/California | 2020-11-23 | Alameda County Public Health Lab | Chan-Zuckerberg Biohub | CZB Cliahub Consortium |
| EPI_ISL_1477569 | hCoV-19/USA/CA-CZB-27335/2020 | North America/USA/California | 2020-10-19 | Alameda County Public Health Lab | Chan-Zuckerberg Biohub | CZB Cliahub Consortium |
| EPI_ISL_3668693 | hCoV-19/USA/AK-PHL11242/2021 | North America/USA/Alaska | 2021-07-28 | Alaska State Virology Laboratory | Alaska State Virology Laboratory | Stephanie DeRonde, Elva House, Jacob Zidek, Lisa Smith, Ph.D., Jack Chen, Ph.D. |
| EPI_ISL_805861 | hCoV-19/Canada/AB-91178/2020 | North America/Canada/Alberta | 2020-03-26 | Alberta Precision Labs (APL) | Alberta Precision Labs (APL) | Gordon P, Lam LG, Pabbaraju K, Wong A, Ma R, Li V, Melin A, Tipples G, Berenger B, Zelyas N, Kellner J, Bernier F, Chui L, Croxen M |
| EPI_ISL_2164898 | hCoV-19/Canada/AB-ABPHL-05476/2020 | North America/Canada/Alberta | 2020-09-21 | Alberta Precision Labs (APL) | Public Health Agency of Canada (PHAC) National Microbiology Laboratory | Buss, E, Croxen M, Deo A, Dieu P, Gill K, Ferrato C, Khan F, Koleva P, Li V, Lloyd C, Lynch T, Ma R, Murphy S, Pabbaraju K, Shokoples S, Tipples G, Thayer J, Whitehouse M, Wong A, Yu C, Zelyas N |
| EPI_ISL_2165327 | hCoV-19/Canada/AB-ABPHL-06088/2020 | North America/Canada/Alberta | 2020-09-21 | Alberta Precision Labs (APL) | Public Health Agency of Canada (PHAC) National Microbiology Laboratory | Buss, E, Croxen M, Deo A, Dieu P, Gill K, Ferrato C, Khan F, Koleva P, Li V, Lloyd C, Lynch T, Ma R, Murphy S, Pabbaraju K, Shokoples S, Tipples G, Thayer J, Whitehouse M, Wong A, Yu C, Zelyas N |
| EPI_ISL_2162912 | hCoV-19/Canada/ABPHL-02297/2020 | North America/Canada/Alberta | 2020-12-17 | Alberta Precision Labs (APL) | Public Health Agency of Canada (PHAC) National Microbiology Laboratory | Buss, E, Croxen M, Deo A, Dieu P, Gill K, Ferrato C, Khan F, Koleva P, Li V, Lloyd C, Lynch T, Ma R, Murphy S, Pabbaraju K, Shokoples S, Tipples G, Thayer J, Whitehouse M, Wong A, Yu C, Zelyas N |
| EPI_ISL_2163153 | hCoV-19/Canada/ABPHL-02630/2021 | North America/Canada/Alberta | 2021-01-01 | Alberta Precision Labs (APL) | Public Health Agency of Canada (PHAC) National Microbiology Laboratory | Buss, E, Croxen M, Deo A, Dieu P, Gill K, Ferrato C, Khan F, Koleva P, Li V, Lloyd C, Lynch T, Ma R, Murphy S, Pabbaraju K, Shokoples S, Tipples G, Thayer J, Whitehouse M, Wong A, Yu C, Zelyas N |
| EPI_ISL_2164037 | hCoV-19/Canada/ABPHL-04042/2021 | North America/Canada/Alberta | 2021-02-03 | Alberta Precision Labs (APL) | Public Health Agency of Canada (PHAC) National Microbiology Laboratory | Buss, E, Croxen M, Deo A, Dieu P, Gill K, Ferrato C, Khan F, Koleva P, Li V, Lloyd C, Lynch T, Ma R, Murphy S, Pabbaraju K, Shokoples S, Tipples G, Thayer J, Whitehouse M, Wong A, Yu C, Zelyas N |
| EPI_ISL_2164148 | hCoV-19/Canada/ABPHL-04186/2020 | North America/Canada/Alberta | 2020-12-02 | Alberta Precision Labs (APL) | Public Health Agency of Canada (PHAC) National Microbiology Laboratory | Buss, E, Croxen M, Deo A, Dieu P, Gill K, Ferrato C, Khan F, Koleva P, Li V, Lloyd C, Lynch T, Ma R, Murphy S, Pabbaraju K, Shokoples S, Tipples G, Thayer J, Whitehouse M, Wong A, Yu C, Zelyas N |
| EPI_ISL_2166333 | hCoV-19/Canada/ABPHL-07817/2021 | North America/Canada/Alberta | 2021-03-05 | Alberta Precision Labs (APL) | Public Health Agency of Canada (PHAC) National Microbiology Laboratory | Buss, E, Croxen M, Deo A, Dieu P, Gill K, Ferrato C, Khan F, Koleva P, Li V, Lloyd C, Lynch T, Ma R, Murphy S, Pabbaraju K, Shokoples S, Tipples G, Thayer J, Whitehouse M, Wong A, Yu C, Zelyas N |
| EPI_ISL_2168161 | hCoV-19/Canada/ABPHL-10306/2021 | North America/Canada/Alberta | 2021-03-15 | Alberta Precision Labs (APL) | Public Health Agency of Canada (PHAC) National Microbiology Laboratory | Buss, E, Croxen M, Deo A, Dieu P, Gill K, Ferrato C, Khan F, Koleva P, Li V, Lloyd C, Lynch T, Ma R, Murphy S, Pabbaraju K, Shokoples S, Tipples G, Thayer J, Whitehouse M, Wong A, Yu C, Zelyas N |
| EPI_ISL_2168585 | hCoV-19/Canada/ABPHL-10780/2021 | North America/Canada/Alberta | 2021-03-22 | Alberta Precision Labs (APL) | Public Health Agency of Canada (PHAC) National Microbiology Laboratory | Buss, E, Croxen M, Deo A, Dieu P, Gill K, Ferrato C, Khan F, Koleva P, Li V, Lloyd C, Lynch T, Ma R, Murphy S, Pabbaraju K, Shokoples S, Tipples G, Thayer J, Whitehouse M, Wong A, Yu C, Zelyas N |
| EPI_ISL_5170759 | hCoV-19/USA/ND-UNDGC-G2021_103_29/2020 | North America/USA/North Dakota | 2020-12-31 | Altru Health System | Genomics Core University of North Dakota | Sara Strandquist, Sara Faraji Jalal Apostal, Antariksh Tyagi, Marijo Roiko, Bony De Kumar, Sergei Nechaev |
| EPI_ISL_5170760 | hCoV-19/USA/ND-UNDGC-G2021_103_31/2020 | North America/USA/North Dakota | 2020-12-31 | Altru Health System | Genomics Core University of North Dakota | Sara Strandquist, Sara Faraji Jalal Apostal, Antariksh Tyagi, Marijo Roiko, Bony De Kumar, Sergei Nechaev |
| EPI_ISL_3713715 | hCoV-19/Germany/BY-RKI-I-204409/2021 | Europe/Germany/Bavaria | 2021-08-11 | amedes MVZ fÃ¼r Laboratoriumsmedizin MÃ¼nchen | Robert Koch Institute | unknown |
| EPI_ISL_1510091 | hCoV-19/Lithuania/S21C1181/2021 | Europe/Lithuania/Vilnius | 2021-03-17 | Anteja laboratorija (UAB Diagnostikos laboratorija) | Vilnius University Hospital Santaros Klinikos, Center of Laboratory Medicine | Gytis Dudas, Ingrida Olendraite, Rimvydas Norvilas, Daniel Naumovas, Dovile Ezerskyte, Ligita Raugaite, Monika Katenaite, Mindaugas Stoskus, Laimonas Griskevicius |
| EPI_ISL_1067609 | hCoV-19/CostaRica/INC-0253/2021 | North America/Costa Rica/Alajuela | 2021-01-26 | AREA DE SALUD ALAJUELA NORTE - CLINICA DR. MARCIAL RODRIGUEZ | Inciensa, Instituto Costarricense de InvestigaciÃ³n y EnseÃ±anza en NutriciÃ³n y Salud | Francisco Duarte, Hebleen Porras, Claudio Soto-Garita, Estela Cordero, Adriana GodÃ­nez, Melany CalderÃ³n & Mariel LÃ³pez |
| EPI_ISL_3948499 | hCoV-19/CostaRica/INC-1073/2021 | North America/Costa Rica/San Jose | 2021-08-03 | AREA DE SALUD ALAJUELITA | Inciensa, Instituto Costarricense de InvestigaciÃ³n y EnseÃ±anza en NutriciÃ³n y Salud | Francisco Duarte, Hebleen Porras, Claudio Soto-Garita, Estela Cordero, Adriana GodÃ­nez, Melany CalderÃ³n, JosÃ© Luis Vargas, Mariela GutiÃ©rrez & Joselyn Prado |
| EPI_ISL_3037812 | hCoV-19/CostaRica/INC-0778/2021 | North America/Costa Rica/Guanacaste | 2021-06-17 | AREA DE SALUD BAGACES | Inciensa, Instituto Costarricense de InvestigaciÃ³n y EnseÃ±anza en NutriciÃ³n y Salud | Francisco Duarte, Hebleen Porras, Claudio Soto-Garita, Estela Cordero, Adriana GodÃ­nez, Melany CalderÃ³n, JosÃ© Luis Vargas, Mariela GutiÃ©rrez Joselyn Prado, Caterina GuzmÃ¡n, Nazareth Ruiz & Adriana BermÃºdez |
| EPI_ISL_3026030 | hCoV-19/CostaRica/INC-0757/2021 | North America/Costa Rica/Heredia | 2021-07-07 | AREA DE SALUD BARVA (COOPESIBA) [BARVA/Heredia] | Inciensa, Instituto Costarricense de InvestigaciÃ³n y EnseÃ±anza en NutriciÃ³n y Salud | Francisco Duarte, Hebleen Porras, Claudio Soto-Garita, Estela Cordero, Adriana GodÃ­nez, Melany CalderÃ³n, JosÃ© Luis Vargas, Mariela GutiÃ©rrez, Joselyn Prado, Caterina GuzmÃ¡n, Nazareth RuÃ­z & Valeria Peralta |
| EPI_ISL_3037817 | hCoV-19/CostaRica/INC-0781/2021 | North America/Costa Rica/Heredia | 2021-06-21 | AREA DE SALUD BELEN-FLORES - CLINICA DR. JORGE VOLIO | Inciensa, Instituto Costarricense de InvestigaciÃ³n y EnseÃ±anza en NutriciÃ³n y Salud | Francisco Duarte, Hebleen Porras, Claudio Soto-Garita, Estela Cordero, Adriana GodÃ­nez, Melany CalderÃ³n, JosÃ© Luis Vargas, Mariela GutiÃ©rrez Joselyn Prado, Caterina GuzmÃ¡n, Nazareth Ruiz & Sandra Mora LeitÃ³n |
| EPI_ISL_914794 | hCoV-19/CostaRica/INC-0187/2020 | North America/Costa Rica/Puntarenas | 2020-12-01 | AREA DE SALUD BUENOS AIRES | Inciensa, Instituto Costarricense de InvestigaciÃ³n y EnseÃ±anza en NutriciÃ³n y Salud | Francisco Duarte, Hebleen Porras, Claudio Soto-Garita, Estela Cordero, Adriana GodÃ­nez, Melany CalderÃ³n & Mariel LÃ³pez |
| EPI_ISL_1067588 | hCoV-19/CostaRica/INC-0244/2021 | North America/Costa Rica/Puntarenas | 2021-01-22 | AREA DE SALUD BUENOS AIRES | Inciensa, Instituto Costarricense de InvestigaciÃ³n y EnseÃ±anza en NutriciÃ³n y Salud | Francisco Duarte, Hebleen Porras, Claudio Soto-Garita, Estela Cordero, Adriana GodÃ­nez, Melany CalderÃ³n & Mariel LÃ³pez |
| EPI_ISL_3639052 | hCoV-19/CostaRica/INC-0983/2021 | North America/Costa Rica/Guanacaste | 2021-08-05 | AREA DE SALUD CAÃ‘AS | Inciensa, Instituto Costarricense de InvestigaciÃ³n y EnseÃ±anza en NutriciÃ³n y Salud | Francisco Duarte, Hebleen Porras, Claudio Soto-Garita, Estela Cordero, Adriana GodÃ­nez, Melany CalderÃ³n, JosÃ© Luis Vargas, Mariela GutiÃ©rrez, Joselyn Prado & Adriana BermÃºdez |
| EPI_ISL_2827995 | hCoV-19/CostaRica/INC-0725/2021 | North America/Costa Rica/Cartago | 2021-06-01 | AREA DE SALUD CARTAGO | Inciensa, Instituto Costarricense de InvestigaciÃ³n y EnseÃ±anza en NutriciÃ³n y Salud | Francisco Duarte, Hebleen Porras, Claudio Soto-Garita, Estela Cordero, Adriana GodÃ­nez, Melany CalderÃ³n, JosÃ© Luis Vargas, Mariela GutiÃ©rrez, Joselyn Prado & Monserrat Segura |
| EPI_ISL_3037814 | hCoV-19/CostaRica/INC-0779/2021 | North America/Costa Rica/Puntarenas | 2021-06-16 | AREA DE SALUD CHACARITA - CLINICA DR. FRANCISCO QUINTANA | Inciensa, Instituto Costarricense de InvestigaciÃ³n y EnseÃ±anza en NutriciÃ³n y Salud | Francisco Duarte, Hebleen Porras, Claudio Soto-Garita, Estela Cordero, Adriana GodÃ­nez, Melany CalderÃ³n, JosÃ© Luis Vargas, Mariela GutiÃ©rrez Joselyn Prado, Caterina GuzmÃ¡n, Nazareth Ruiz & MarÃ­a JosÃ© GÃ³mez |
| EPI_ISL_2658271 | hCoV-19/CostaRica/INC-0674/2021 | North America/Costa Rica/Puntarenas | 2021-04-27 | AREA DE SALUD CHOMES - MONTEVERDE | Inciensa, Instituto Costarricense de InvestigaciÃ³n y EnseÃ±anza en NutriciÃ³n y Salud | Francisco Duarte, Hebleen Porras, Claudio Soto-Garita, Estela Cordero, Adriana GodÃ­nez, Melany CalderÃ³n, JosÃ© Luis Vargas, Mariela GutiÃ©rrez, Joselyn Prado & Javier ZÃ¡rate-LeÃ³n |
| EPI_ISL_2827982 | hCoV-19/CostaRica/INC-0712/2021 | North America/Costa Rica/Puntarenas | 2021-05-25 | AREA DE SALUD COBANO | Inciensa, Instituto Costarricense de InvestigaciÃ³n y EnseÃ±anza en NutriciÃ³n y Salud | Francisco Duarte, Hebleen Porras, Claudio Soto-Garita, Estela Cordero, Adriana GodÃ­nez, Melany CalderÃ³n, JosÃ© Luis Vargas, Mariela GutiÃ©rrez, Joselyn Prado & MarÃ­a JosÃ© GÃ³mez |
| EPI_ISL_1067618 | hCoV-19/CostaRica/INC-0254/2021 | North America/Costa Rica/Cartago | 2021-01-25 | AREA DE SALUD CORRALILLO | Inciensa, Instituto Costarricense de InvestigaciÃ³n y EnseÃ±anza en NutriciÃ³n y Salud | Francisco Duarte, Hebleen Porras, Claudio Soto-Garita, Estela Cordero, Adriana GodÃ­nez, Melany CalderÃ³n, Caterina GuzmÃ¡n, Nazareth Ruiz & MÃ³nica Charpentier-Artavia |
| EPI_ISL_512668 | hCoV-19/CostaRica/INC-0062/2020 | North America/Costa Rica/Puntarenas | 2020-06-19 | Area De Salud Corredores | Inciensa, Instituto Costarricense de InvestigaciÃ³n y EnseÃ±anza en NutriciÃ³n y Salud | Francisco Duarte, Hebleen Porras, Claudio Soto-Garita, Estela Cordero, Adriana Godinez & Melany Calderon |
| EPI_ISL_1196421 | hCoV-19/CostaRica/INC-0278/2021 | North America/Costa Rica/Puntarenas | 2021-02-09 | AREA DE SALUD CORREDORES | Inciensa, Instituto Costarricense de InvestigaciÃ³n y EnseÃ±anza en NutriciÃ³n y Salud | Francisco Duarte, Hebleen Porras, Claudio Soto-Garita, Estela Cordero, Adriana GodÃ­nez, Melany CalderÃ³n & Mariel LÃ³pez |
| EPI_ISL_3026026 | hCoV-19/CostaRica/INC-0751/2021 | North America/Costa Rica/Puntarenas | 2021-06-30 | AREA DE SALUD CORREDORES [CORREDORES/PUNTARENAS] | Inciensa, Instituto Costarricense de InvestigaciÃ³n y EnseÃ±anza en NutriciÃ³n y Salud | Francisco Duarte, Hebleen Porras, Claudio Soto-Garita, Estela Cordero, Adriana GodÃ­nez, Melany CalderÃ³n, JosÃ© Luis Vargas, Mariela GutiÃ©rrez, Joselyn Prado, Caterina GuzmÃ¡n, Nazareth RuÃ­z & MarÃ­a Victoria |
| EPI_ISL_1201437 | hCoV-19/CostaRica/INC-0246/2021 | North America/Costa Rica/Puntarenas | 2021-01-22 | AREA DE SALUD COTO BRUS | Inciensa, Instituto Costarricense de InvestigaciÃ³n y EnseÃ±anza en NutriciÃ³n y Salud | Francisco Duarte, Hebleen Porras, Claudio Soto-Garita, Estela Cordero, Adriana GodÃ­nez, Melany CalderÃ³n & MÃ³nica Charpentier-Artavia |
| EPI_ISL_1827536 | hCoV-19/CostaRica/INC-0507/2021 | North America/Costa Rica/Puntarenas | 2021-03-15 | AREA DE SALUD COTO BRUS | Inciensa, Instituto Costarricense de InvestigaciÃ³n y EnseÃ±anza en NutriciÃ³n y Salud | Francisco Duarte, Hebleen Porras, Claudio Soto-Garita, Estela Cordero, Adriana GodÃ­nez, Melany CalderÃ³n, JosÃ© Luis Vargas, Mariela GutiÃ©rrez, Joselyn Prado & Fabricio Aguilar |
| EPI_ISL_3464520 | hCoV-19/CostaRica/INC-0894/2021 | North America/Costa Rica/Puntarenas | 2021-07-09 | AREA DE SALUD COTO BRUS | Inciensa, Instituto Costarricense de InvestigaciÃ³n y EnseÃ±anza en NutriciÃ³n y Salud | Francisco Duarte, Hebleen Porras, Claudio Soto-Garita, Estela Cordero, Adriana GodÃ­nez, Melany CalderÃ³n, JosÃ© Luis Vargas, Mariela GutiÃ©rrez, Joselyn Prado & Yendri RamÃ­rez AlpÃ­zar |
| EPI_ISL_3274354 | hCoV-19/CostaRica/INC-0783/2021 | North America/Costa Rica/San Jose | 2021-06-23 | AREA DE SALUD DESAMPARADOS 1 - CLINICA DR. MARCIAL FALLAS | Inciensa, Instituto Costarricense de InvestigaciÃ³n y EnseÃ±anza en NutriciÃ³n y Salud | Francisco Duarte, Hebleen Porras, Claudio Soto-Garita, Estela Cordero, Adriana GodÃ­nez, Melany CalderÃ³n, JosÃ© Luis Vargas, Mariela GutiÃ©rrez Joselyn Prado, Caterina GuzmÃ¡n, & Nazareth Ruiz |
| EPI_ISL_3026022 | hCoV-19/CostaRica/INC-0747/2021 | North America/Costa Rica/ | 2021-06-24 | AREA DE SALUD EL GUARCO [EL GUARCO/CARTAGO] | Inciensa, Instituto Costarricense de InvestigaciÃ³n y EnseÃ±anza en NutriciÃ³n y Salud | Francisco Duarte, Hebleen Porras, Claudio Soto-Garita, Estela Cordero, Adriana GodÃ­nez, Melany CalderÃ³n, JosÃ© Luis Vargas, Mariela GutiÃ©rrez, Joselyn Prado, Caterina GuzmÃ¡n, Nazareth RuÃ­z & MÃ³nica Charpentier |
| EPI_ISL_3464528 | hCoV-19/CostaRica/INC-0905/2021 | North America/Costa Rica/Alajuela | 2021-07-28 | AREA DE SALUD FORTUNA | Inciensa, Instituto Costarricense de InvestigaciÃ³n y EnseÃ±anza en NutriciÃ³n y Salud | Francisco Duarte, Hebleen Porras, Claudio Soto-Garita, Estela Cordero, Adriana GodÃ­nez, Melany CalderÃ³n, JosÃ© Luis Vargas, Mariela GutiÃ©rrez, Joselyn Prado & Juan Carlos Villalobos Ugalde |
| EPI_ISL_682240 | hCoV-19/CostaRica/INC-0099/2020 | North America/Costa Rica/San Jose | 2020-07-10 | AREA DE SALUD GOICOCHEA 1 | Inciensa, Instituto Costarricense de InvestigaciÃ³n y EnseÃ±anza en NutriciÃ³n y Salud | Francisco Duarte, Hebleen Porras, Claudio Soto-Garita, Estela Cordero, Adriana Godinez & Melany Calderon |
| EPI_ISL_527757 | hCoV-19/CostaRica/INC-0089/2020 | North America/Costa Rica/San Jose | 2020-07-10 | Area De Salud Goicoechea 1 | Inciensa, Instituto Costarricense de InvestigaciÃ³n y EnseÃ±anza en NutriciÃ³n y Salud | Francisco Duarte, Hebleen Porras, Claudio Soto-Garita, Estela Cordero, Adriana Godinez & Melany Calderon |
| EPI_ISL_2502755 | hCoV-19/CostaRica/INC-0659/2021 | North America/Costa Rica/Cartago | 2021-05-22 | AREA DE SALUD GOICOECHEA 2 - CLINICA DR. JIMENEZ NUÃ‘EZ | Inciensa, Instituto Costarricense de InvestigaciOn y EnseÃ±anza en NutriciOn y Salud | Francisco Duarte, Hebleen Porras, Claudio Soto-Garita, Estela Cordero, Adriana GodÃ­nez, Melany CalderOn, Jose Luis Vargas, Mariela Gutierrez, Joselyn Prado & Alberto Madrigal-Vega |
| EPI_ISL_3037801 | hCoV-19/CostaRica/INC-0767/2021 | North America/Costa Rica/San Jose | 2021-06-15 | AREA DE SALUD GOICOECHEA 2 - CLINICA DR. JIMENEZ NUÃ‘EZ | Inciensa, Instituto Costarricense de InvestigaciÃ³n y EnseÃ±anza en NutriciÃ³n y Salud | Francisco Duarte, Hebleen Porras, Claudio Soto-Garita, Estela Cordero, Adriana GodÃ­nez, Melany CalderÃ³n, JosÃ© Luis Vargas, Mariela GutiÃ©rrez Joselyn Prado, Caterina GuzmÃ¡n, & Nazareth Ruiz |
| EPI_ISL_770016 | hCoV-19/CostaRica/INC-0166/2020 | North America/Costa Rica/San Jose | 2020-10-19 | Area De Salud Goicoechea 2 - Clinica Dr. Jimenez NuÃ±ez | Inciensa, Instituto Costarricense de InvestigaciÃ³n y EnseÃ±anza en NutriciÃ³n y Salud | Francisco Duarte, Hebleen Porras, Claudio Soto-Garita, Estela Cordero, Adriana GodÃ­nez, Melany CalderÃ³n & Mariel LÃ³pez |
| EPI_ISL_3298372 | hCoV-19/CostaRica/INC-0886/2021 | North America/Costa Rica/Alajuela | 2021-07-21 | AREA DE SALUD GUATUSO | Inciensa, Instituto Costarricense de InvestigaciÃ³n y EnseÃ±anza en NutriciÃ³n y Salud | Francisco Duarte, Hebleen Porras, Claudio Soto-Garita, Estela Cordero, Adriana GodÃ­nez, Melany CalderÃ³n, JosÃ© Luis Vargas, Mariela GutiÃ©rrez, Joselyn Prado & Francisco ChacÃ³n |
| EPI_ISL_4258628 | hCoV-19/CostaRica/INC-1157/2021 | North America/Costa Rica/Alajuela | 2021-08-17 | AREA DE SALUD GUATUSO | Inciensa, Instituto Costarricense de InvestigaciÃ³n y EnseÃ±anza en NutriciÃ³n y Salud | Francisco Duarte, Hebleen Porras, Claudio Soto-Garita, Estela Cordero, Adriana GodÃ­nez, Melany CalderÃ³n, JosÃ© Luis Vargas, Mariela GutiÃ©rrez, Joselyn Prado & Juan Carlos Villalobos Ugalde |
| EPI_ISL_3026025 | hCoV-19/CostaRica/INC-0750/2021 | North America/Costa Rica/Heredia | 2021-06-22 | AREA DE SALUD Heredia-VIRILLA [Heredia/Heredia] | Inciensa, Instituto Costarricense de InvestigaciÃ³n y EnseÃ±anza en NutriciÃ³n y Salud | Francisco Duarte, Hebleen Porras, Claudio Soto-Garita, Estela Cordero, Adriana GodÃ­nez, Melany CalderÃ³n, JosÃ© Luis Vargas, Mariela GutiÃ©rrez, Joselyn Prado, Caterina GuzmÃ¡n, Nazareth RuÃ­z & Laura Garro |
| EPI_ISL_527747 | hCoV-19/CostaRica/INC-0079/2020 | North America/Costa Rica/Guanacaste | 2020-07-03 | Area De Salud La Cruz | Inciensa, Instituto Costarricense de InvestigaciÃ³n y EnseÃ±anza en NutriciÃ³n y Salud | Francisco Duarte, Hebleen Porras, Claudio Soto-Garita, Estela Cordero, Adriana Godinez & Melany Calderon |
| EPI_ISL_527760 | hCoV-19/CostaRica/INC-0092/2020 | North America/Costa Rica/San Jose | 2020-06-11 | Area De Salud La Cruz | Inciensa, Instituto Costarricense de InvestigaciÃ³n y EnseÃ±anza en NutriciÃ³n y Salud | Francisco Duarte, Hebleen Porras, Claudio Soto-Garita, Estela Cordero, Adriana Godinez & Melany Calderon |
| EPI_ISL_770000 | hCoV-19/CostaRica/INC-0158/2020 | North America/Costa Rica/Guanacaste | 2020-10-05 | Area De Salud La Cruz | Inciensa, Instituto Costarricense de InvestigaciÃ³n y EnseÃ±anza en NutriciÃ³n y Salud | Francisco Duarte, Hebleen Porras, Claudio Soto-Garita, Estela Cordero, Adriana GodÃ­nez, Melany CalderÃ³n & Mariel LÃ³pez |
| EPI_ISL_770002 | hCoV-19/CostaRica/INC-0161/2020 | North America/Costa Rica/Guanacaste | 2020-10-09 | Area De Salud La Cruz | Inciensa, Instituto Costarricense de InvestigaciÃ³n y EnseÃ±anza en NutriciÃ³n y Salud | Francisco Duarte, Hebleen Porras, Claudio Soto-Garita, Estela Cordero, Adriana GodÃ­nez, Melany CalderÃ³n & Mariel LÃ³pez |
| EPI_ISL_770003 | hCoV-19/CostaRica/INC-0173/2020 | North America/Costa Rica/Guanacaste | 2020-11-04 | Area De Salud La Cruz | Inciensa, Instituto Costarricense de InvestigaciÃ³n y EnseÃ±anza en NutriciÃ³n y Salud | Francisco Duarte, Hebleen Porras, Claudio Soto-Garita, Estela Cordero, Adriana GodÃ­nez, Melany CalderÃ³n & Mariel LÃ³pez |
| EPI_ISL_3464538 | hCoV-19/CostaRica/INC-0919/2021 | North America/Costa Rica/Guanacaste | 2021-07-20 | AREA DE SALUD LA CRUZ | Inciensa, Instituto Costarricense de InvestigaciÃ³n y EnseÃ±anza en NutriciÃ³n y Salud | Francisco Duarte, Hebleen Porras, Claudio Soto-Garita, Estela Cordero, Adriana GodÃ­nez, Melany CalderÃ³n, JosÃ© Luis Vargas, Mariela GutiÃ©rrez, Joselyn Prado & Adriana BermÃºdez Espinoza |
| EPI_ISL_2502756 | hCoV-19/CostaRica/INC-0660/2021 | North America/Costa Rica/Cartago | 2021-05-24 | AREA DE SALUD LA UNION | Inciensa, Instituto Costarricense de InvestigaciOn y EnseÃ±anza en NutriciOn y Salud | Francisco Duarte, Hebleen Porras, Claudio Soto-Garita, Estela Cordero, Adriana GodÃ­nez, Melany CalderOn, Jose Luis Vargas, Mariela Gutierrez, Joselyn Prado & MOnica Charpentier |
| EPI_ISL_3639156 | hCoV-19/CostaRica/INC-1001/2021 | North America/Costa Rica/LimÃ³n | 2021-08-04 | AREA DE SALUD LIMON | Inciensa, Instituto Costarricense de InvestigaciÃ³n y EnseÃ±anza en NutriciÃ³n y Salud | Francisco Duarte, Hebleen Porras, Claudio Soto-Garita, Estela Cordero, Adriana GodÃ­nez, Melany CalderÃ³n, JosÃ© Luis Vargas, Mariela GutiÃ©rrez, Joselyn Prado & Jose ZÃºÃ±iga |
| EPI_ISL_3026032 | hCoV-19/CostaRica/INC-0759/2021 | North America/Costa Rica/Limon | 2021-07-07 | AREA DE SALUD LIMON [LIMON/LIMON] | Inciensa, Instituto Costarricense de InvestigaciÃ³n y EnseÃ±anza en NutriciÃ³n y Salud | Francisco Duarte, Hebleen Porras, Claudio Soto-Garita, Estela Cordero, Adriana GodÃ­nez, Melany CalderÃ³n, JosÃ© Luis Vargas, Mariela GutiÃ©rrez, Joselyn Prado, Caterina GuzmÃ¡n, Nazareth RuÃ­z & Karolina Hall |
| EPI_ISL_3274367 | hCoV-19/CostaRica/INC-0814/2021 | North America/Costa Rica/San Jose | 2021-07-01 | AREA DE SALUD MATA REDONDA-HOSPITAL - CLINICA DR. MORENO CAÃ‘AS [SAN JOSE/SAN JOSE] | Inciensa, Instituto Costarricense de InvestigaciÃ³n y EnseÃ±anza en NutriciÃ³n y Salud | Francisco Duarte, Hebleen Porras, Claudio Soto-Garita, Estela Cordero, Adriana GodÃ­nez, Melany CalderÃ³n, JosÃ© Luis Vargas, Mariela GutiÃ©rrez Joselyn Prado, Caterina GuzmÃ¡n, Nazareth Ruiz & Ricardo GonzÃ¡lez |
| EPI_ISL_4258634 | hCoV-19/CostaRica/INC-1130/2021 | North America/Costa Rica/Limon | 2021-08-12 | AREA DE SALUD MATINA | Inciensa, Instituto Costarricense de InvestigaciÃ³n y EnseÃ±anza en NutriciÃ³n y Salud | Francisco Duarte, Hebleen Porras, Claudio Soto-Garita, Estela Cordero, Adriana GodÃ­nez, Melany CalderÃ³n, JosÃ© Luis Vargas, Mariela GutiÃ©rrez, Joselyn Prado & Tashana Anglin Williams |
| EPI_ISL_1712418 | hCoV-19/CostaRica/INC-0450/2021 | North America/Costa Rica/Alajuela | 2021-03-10 | AREA DE SALUD NARANJO | Inciensa, Instituto Costarricense de InvestigaciÃ³n y EnseÃ±anza en NutriciÃ³n y Salud | Francisco Duarte, Hebleen Porras, Claudio Soto-Garita, Estela Cordero, Adriana GodÃ­nez, Melany CalderÃ³n, JosÃ© Luis Vargas, Mariela GutiÃ©rrez, Joselyn Prado & Teresita Somogyi |
| EPI_ISL_512658 | hCoV-19/CostaRica/INC-0052/2020 | North America/Costa Rica/Puntarenas | 2020-03-21 | Area De Salud Orotina-San Mateo [Orotina/Alajuela] | Inciensa, Instituto Costarricense de InvestigaciÃ³n y EnseÃ±anza en NutriciÃ³n y Salud | Francisco Duarte, Hebleen Porras, Claudio Soto-Garita, Estela Cordero, Adriana Godinez & Melany Calderon |
| EPI_ISL_3037804 | hCoV-19/CostaRica/INC-0769/2021 | North America/Costa Rica/Puntarenas | 2021-06-14 | AREA DE SALUD OSA | Inciensa, Instituto Costarricense de InvestigaciÃ³n y EnseÃ±anza en NutriciÃ³n y Salud | Francisco Duarte, Hebleen Porras, Claudio Soto-Garita, Estela Cordero, Adriana GodÃ­nez, Melany CalderÃ³n, JosÃ© Luis Vargas, Mariela GutiÃ©rrez Joselyn Prado, Caterina GuzmÃ¡n, Nazareth Ruiz & Mariamilia Cob |
| EPI_ISL_3464514 | hCoV-19/CostaRica/INC-0917/2021 | North America/Costa Rica/Puntarenas | 2021-07-18 | AREA DE SALUD PAQUERA | Inciensa, Instituto Costarricense de InvestigaciÃ³n y EnseÃ±anza en NutriciÃ³n y Salud | Francisco Duarte, Hebleen Porras, Claudio Soto-Garita, Estela Cordero, Adriana GodÃ­nez, Melany CalderÃ³n, JosÃ© Luis Vargas, Mariela GutiÃ©rrez, Joselyn Prado & Ariela Rojas Herrera |
| EPI_ISL_2502757 | hCoV-19/CostaRica/INC-0661/2021 | North America/Costa Rica/Cartago | 2021-05-24 | AREA DE SALUD PARAISO-CERVANTES | Inciensa, Instituto Costarricense de InvestigaciOn y EnseÃ±anza en NutriciOn y Salud | Francisco Duarte, Hebleen Porras, Claudio Soto-Garita, Estela Cordero, Adriana GodÃ­nez, Melany CalderOn, Jose Luis Vargas, Mariela Gutierrez, Joselyn Prado & MOnica Charpentier |
| EPI_ISL_3037802 | hCoV-19/CostaRica/INC-0768/2021 | North America/Costa Rica/Cartago | 2021-06-17 | AREA DE SALUD PARAISO-CERVANTES | Inciensa, Instituto Costarricense de InvestigaciÃ³n y EnseÃ±anza en NutriciÃ³n y Salud | Francisco Duarte, Hebleen Porras, Claudio Soto-Garita, Estela Cordero, Adriana GodÃ­nez, Melany CalderÃ³n, JosÃ© Luis Vargas, Mariela GutiÃ©rrez Joselyn Prado, Caterina GuzmÃ¡n, Nazareth Ruiz & MÃ³nica Charpentier |
| EPI_ISL_3037822 | hCoV-19/CostaRica/INC-0789/2021 | North America/Costa Rica/Cartago | 2021-06-28 | AREA DE SALUD PARAISO-CERVANTES | Inciensa, Instituto Costarricense de InvestigaciÃ³n y EnseÃ±anza en NutriciÃ³n y Salud | Francisco Duarte, Hebleen Porras, Claudio Soto-Garita, Estela Cordero, Adriana GodÃ­nez, Melany CalderÃ³n, JosÃ© Luis Vargas, Mariela GutiÃ©rrez Joselyn Prado, Caterina GuzmÃ¡n, Nazareth Ruiz & MÃ³nica Charpentier |
| EPI_ISL_3948518 | hCoV-19/CostaRica/INC-1092/2021 | North America/Costa Rica/Cartago | 2021-08-10 | AREA DE SALUD PARAISO-CERVANTES | Inciensa, Instituto Costarricense de InvestigaciÃ³n y EnseÃ±anza en NutriciÃ³n y Salud | Francisco Duarte, Hebleen Porras, Claudio Soto-Garita, Estela Cordero, Adriana GodÃ­nez, Melany CalderÃ³n, JosÃ© Luis Vargas, Mariela GutiÃ©rrez, Joselyn Prado & MÃ³nica Charpentier |
| EPI_ISL_4258646 | hCoV-19/CostaRica/INC-1181/2021 | North America/Costa Rica/Cartago | 2021-08-20 | AREA DE SALUD PARAISO-CERVANTES | Inciensa, Instituto Costarricense de InvestigaciÃ³n y EnseÃ±anza en NutriciÃ³n y Salud | Francisco Duarte, Hebleen Porras, Claudio Soto-Garita, Estela Cordero, Adriana GodÃ­nez, Melany CalderÃ³n, JosÃ© Luis Vargas, Mariela GutiÃ©rrez, Joselyn Prado & MÃ³nica Chapentier Artavia |
| EPI_ISL_3464525 | hCoV-19/CostaRica/INC-0901/2021 | North America/Costa Rica/San Jose | 2021-07-28 | AREA DE SALUD PAVAS (COOPESALUD) | Inciensa, Instituto Costarricense de InvestigaciÃ³n y EnseÃ±anza en NutriciÃ³n y Salud | Francisco Duarte, Hebleen Porras, Claudio Soto-Garita, Estela Cordero, Adriana GodÃ­nez, Melany CalderÃ³n, JosÃ© Luis Vargas, Mariela GutiÃ©rrez, Joselyn Prado & Paola Sanchez TraÃ±a |
| EPI_ISL_3464543 | hCoV-19/CostaRica/INC-0926/2021 | North America/Costa Rica/San Jose | 2021-07-25 | AREA DE SALUD PAVAS (COOPESALUD) | Inciensa, Instituto Costarricense de InvestigaciÃ³n y EnseÃ±anza en NutriciÃ³n y Salud | Francisco Duarte, Hebleen Porras, Claudio Soto-Garita, Estela Cordero, Adriana GodÃ­nez, Melany CalderÃ³n, JosÃ© Luis Vargas, Mariela GutiÃ©rrez & Joselyn Prado |
| EPI_ISL_4258662 | hCoV-19/CostaRica/INC-1167/2021 | North America/Costa Rica/San Jose | 2021-08-17 | AREA DE SALUD PAVAS (COOPESALUD) | Inciensa, Instituto Costarricense de InvestigaciÃ³n y EnseÃ±anza en NutriciÃ³n y Salud | Francisco Duarte, Hebleen Porras, Claudio Soto-Garita, Estela Cordero, Adriana GodÃ­nez, Melany CalderÃ³n, JosÃ© Luis Vargas, Mariela GutiÃ©rrez & Joselyn Prado |
| EPI_ISL_3037828 | hCoV-19/CostaRica/INC-0796/2021 | North America/Costa Rica/Heredia | 2021-06-28 | AREA DE SALUD SAN ISIDRO | Inciensa, Instituto Costarricense de InvestigaciÃ³n y EnseÃ±anza en NutriciÃ³n y Salud | Francisco Duarte, Hebleen Porras, Claudio Soto-Garita, Estela Cordero, Adriana GodÃ­nez, Melany CalderÃ³n, JosÃ© Luis Vargas, Mariela GutiÃ©rrez Joselyn Prado, Caterina GuzmÃ¡n, Nazareth Ruiz & Cristian PÃ©rez |
| EPI_ISL_770025 | hCoV-19/CostaRica/INC-0165/2020 | North America/Costa Rica/Cartago | 2020-10-19 | Area De Salud San Juan-San Diego-Concepcion 2 | Inciensa, Instituto Costarricense de InvestigaciÃ³n y EnseÃ±anza en NutriciÃ³n y Salud | Francisco Duarte, Hebleen Porras, Claudio Soto-Garita, Estela Cordero, Adriana GodÃ­nez, Melany CalderÃ³n & Mariel LÃ³pez |
| EPI_ISL_2502727 | hCoV-19/CostaRica/INC-0629/2021 | North America/Costa Rica/Cartago | 2021-05-04 | AREA DE SALUD SAN JUAN-SAN DIEGO-CONCEPCION 2 | Inciensa, Instituto Costarricense de InvestigaciOn y EnseÃ±anza en NutriciOn y Salud | Francisco Duarte, Hebleen Porras, Claudio Soto-Garita, Estela Cordero, Adriana GodÃ­nez, Melany CalderOn, Jose Luis Vargas, Mariela Gutierrez, Joselyn Prado & Mariel LOpez |
| EPI_ISL_3638816 | hCoV-19/CostaRica/INC-0945/2021 | North America/Costa Rica/Cartago | 2021-07-22 | AREA DE SALUD SAN JUAN-SAN DIEGO-CONCEPCION 2 | Inciensa, Instituto Costarricense de InvestigaciÃ³n y EnseÃ±anza en NutriciÃ³n y Salud | Francisco Duarte, Hebleen Porras, Claudio Soto-Garita, Estela Cordero, Adriana GodÃ­nez, Melany CalderÃ³n, JosÃ© Luis Vargas, Mariela GutiÃ©rrez & Joselyn Prado |
| EPI_ISL_770007 | hCoV-19/CostaRica/INC-0157/2020 | North America/Costa Rica/Puntarenas | 2020-09-30 | Area De Salud San Rafael | Inciensa, Instituto Costarricense de InvestigaciÃ³n y EnseÃ±anza en NutriciÃ³n y Salud | Francisco Duarte, Hebleen Porras, Claudio Soto-Garita, Estela Cordero, Adriana GodÃ­nez, Melany CalderÃ³n & Mariel LÃ³pez |
| EPI_ISL_2828007 | hCoV-19/CostaRica/INC-0737/2021 | North America/Costa Rica/Alajuela | 2021-06-14 | AREA DE SALUD SAN RAMON | Inciensa, Instituto Costarricense de InvestigaciÃ³n y EnseÃ±anza en NutriciÃ³n y Salud | Francisco Duarte, Hebleen Porras, Claudio Soto-Garita, Estela Cordero, Adriana GodÃ­nez, Melany CalderÃ³n, JosÃ© Luis Vargas, Mariela GutiÃ©rrez & Joselyn Prado |
| EPI_ISL_3298362 | hCoV-19/CostaRica/INC-0874/2021 | North America/Costa Rica/Guanacaste | 2021-07-19 | AREA DE SALUD SANTA CRUZ | Inciensa, Instituto Costarricense de InvestigaciÃ³n y EnseÃ±anza en NutriciÃ³n y Salud | Francisco Duarte, Hebleen Porras, Claudio Soto-Garita, Estela Cordero, Adriana GodÃ­nez, Melany CalderÃ³n, JosÃ© Luis Vargas, Mariela GutiÃ©rrez, Joselyn Prado & Fabiola GutiÃ©rrez |
| EPI_ISL_3948515 | hCoV-19/CostaRica/INC-1089/2021 | North America/Costa Rica/Guanacaste | 2021-08-09 | AREA DE SALUD SANTA CRUZ | Inciensa, Instituto Costarricense de InvestigaciÃ³n y EnseÃ±anza en NutriciÃ³n y Salud | Francisco Duarte, Hebleen Porras, Claudio Soto-Garita, Estela Cordero, Adriana GodÃ­nez, Melany CalderÃ³n, JosÃ© Luis Vargas, Mariela GutiÃ©rrez, Joselyn Prado & Fabiola GutiÃ©rrez |
| EPI_ISL_2827969 | hCoV-19/CostaRica/INC-0699/2021 | North America/Costa Rica/Limon | 2021-05-28 | AREA DE SALUD SIQUIRRES | Inciensa, Instituto Costarricense de InvestigaciÃ³n y EnseÃ±anza en NutriciÃ³n y Salud | Francisco Duarte, Hebleen Porras, Claudio Soto-Garita, Estela Cordero, Adriana GodÃ­nez, Melany CalderÃ³n, JosÃ© Luis Vargas, Mariela GutiÃ©rrez, Joselyn Prado & Ileana Chavez |
| EPI_ISL_3638878 | hCoV-19/CostaRica/INC-0955/2021 | North America/Costa Rica/LimÃ³n | 2021-07-24 | AREA DE SALUD SIQUIRRES | Inciensa, Instituto Costarricense de InvestigaciÃ³n y EnseÃ±anza en NutriciÃ³n y Salud | Francisco Duarte, Hebleen Porras, Claudio Soto-Garita, Estela Cordero, Adriana GodÃ­nez, Melany CalderÃ³n, JosÃ© Luis Vargas, Mariela GutiÃ©rrez & Joselyn Prado |
| EPI_ISL_3037839 | hCoV-19/CostaRica/INC-0809/2021 | North America/Costa Rica/Limon | 2021-06-29 | AREA DE SALUD SIQUIRRES [SIQUIRRES/LIMON] | Inciensa, Instituto Costarricense de InvestigaciÃ³n y EnseÃ±anza en NutriciÃ³n y Salud | Francisco Duarte, Hebleen Porras, Claudio Soto-Garita, Estela Cordero, Adriana GodÃ­nez, Melany CalderÃ³n, JosÃ© Luis Vargas, Mariela GutiÃ©rrez Joselyn Prado, Caterina GuzmÃ¡n, Nazareth Ruiz & Ileana Chavez Peraza |
| EPI_ISL_3464545 | hCoV-19/CostaRica/INC-0928/2021 | North America/Costa Rica/Alajuela | 2021-07-23 | AREA DE SALUD UPALA | Inciensa, Instituto Costarricense de InvestigaciÃ³n y EnseÃ±anza en NutriciÃ³n y Salud | Francisco Duarte, Hebleen Porras, Claudio Soto-Garita, Estela Cordero, Adriana GodÃ­nez, Melany CalderÃ³n, JosÃ© Luis Vargas, Mariela GutiÃ©rrez, Joselyn Prado & Juan Carlos Villalobos Ugalde |
| EPI_ISL_2502758 | hCoV-19/CostaRica/INC-0662/2021 | North America/Costa Rica/San Jose | 2021-05-22 | AREA DE SALUD ZAPOTE-CATEDRAL - CLINICA DR. CARLOS DURAN | Inciensa, Instituto Costarricense de InvestigaciOn y EnseÃ±anza en NutriciOn y Salud | Francisco Duarte, Hebleen Porras, Claudio Soto-Garita, Estela Cordero, Adriana GodÃ­nez, Melany CalderOn, Jose Luis Vargas, Mariela Gutierrez, Joselyn Prado & Marco ChÃ¡ves-OtÃ¡rola |
| EPI_ISL_2502759 | hCoV-19/CostaRica/INC-0663/2021 | North America/Costa Rica/San Jose | 2021-05-23 | AREA DE SALUD ZAPOTE-CATEDRAL - CLINICA DR. CARLOS DURAN | Inciensa, Instituto Costarricense de InvestigaciOn y EnseÃ±anza en NutriciOn y Salud | Francisco Duarte, Hebleen Porras, Claudio Soto-Garita, Estela Cordero, Adriana GodÃ­nez, Melany CalderOn, Jose Luis Vargas, Mariela Gutierrez, Joselyn Prado & Marco ChÃ¡ves-OtÃ¡rola |
| EPI_ISL_406223 | hCoV-19/USA/AZ-CDC-02993465-001/2020 | North America/USA/Arizona | 2020-01-22 | Arizona Department of Health Services | Pathogen Discovery, Respiratory Viruses Branch, Division of Viral Diseases, Centers for Disease Control and Prevention | Ying Tao, Clinton R. Paden, Krista Queen, Anna Uehara, Yan Li, Jing Zhang, Xiaoyan Lu, Brian Lynch, Senthil Kumar K. Sakthivel, Brett L. Whitaker, Shifaq Kamili, Lijuan Wang, Janna' R. Murray, Susan I. Gerber, Stephen Lindstrom, Suxiang Tong |
| EPI_ISL_4257534 | hCoV-19/USA/AZ-ASU14434/2021 | North America/USA/Arizona | 2021-09-06 | Arizona State University | Arizona State University | Ajeet Bains, LaRinda A. Holland, Matthew F. Smith, Regan A. Sullins, Nicholas J. Mellor, Nathaniel Johnson, Joshua LaBaer, Vel Murugan, Efrem S. Lim |
| EPI_ISL_3474087 | hCoV-19/USA/AR-UMGC-7706/2021 | North America/USA/Arkansas | 2021-07-30 | Arkansas Public Health Laboratory, Arkansas Department of Health | University of Minnesota Genomics Center | Daryl M. Gohl, John Garbe, Jaquelyn Kuriger-Laber, Corbin Dirkx |
| EPI_ISL_3721184 | hCoV-19/USA/AR-UMGC-9394/2021 | North America/USA/Arkansas | 2021-07-27 | Arkansas Public Health Laboratory, Arkansas Department of Health | University of Minnesota Genomics Center | Daryl M. Gohl, John Garbe, Jaquelyn Kuriger-Laber, Corbin Dirkx |
| EPI_ISL_1444125 | hCoV-19/Portugal/PT5332/2021 | Europe/Portugal | 2021-03-01 | ARS Algarve - Laboratorio Laura Ayres | Instituto Nacional de Saude (INSA) and i3S - Instituto de InvestigaÃ§Ã£o e InovaÃ§Ã£o em SaÃºde | Borges et al |
| EPI_ISL_1811225 | hCoV-19/CostaRica/HNN-0455/2021 | North America/Costa Rica/Heredia | 2021-04-04 | AS Puerto Viejo | Inciensa, Instituto Costarricense de InvestigaciÃ³n y EnseÃ±anza en NutriciÃ³n y Salud | PÃ©rez-Corrales C & Murillo-Bustos H |
| EPI_ISL_1533526 | hCoV-19/USA/GA-CDC-4049192-001/2020 | North America/USA/Georgia | 2020-10-18 | Atlanta VA Medical Center | Genomics and Discovery, Respiratory Viruses Branch, Division of Viral Diseases, Centers for Disease Control and Prevention | Yan Li, Ying Tao, Anna Kelleher, Jing Zhang, Anna Montmayeur, Brian Lynch, Krista Queen, Anna Uehara, Peter Cook, Han Jia Justin Ng, Rachel Marine, Clinton R. Paden, Haibin Wang, Mark Burroughs, Justin Lee, Adam Retchless, Suxiang Tong |
| EPI_ISL_2975142 | hCoV-19/Italy/VEN-IZSVe-21RS1729-1_PD/2021 | Europe/Italy/Veneto | 2021-06-28 | AULSS 6 Euganea | Istituto Zooprofilattico Sperimentale delle Venezie | Adelaide Milani, Alessia Schivo, Annalisa Salviato, Elisa Palumbo, Erika Giorgia Quaranta, Luca Tassoni, Ambra Pastori, Edoardo Giussani, Alice Fusaro, Isabella Monne, Calogero Terregino, Antonia Ricci |
| EPI_ISL_4080678 | hCoV-19/Chile/LL-UACH-00088/2021 | South America/Chile/Los Lagos | 2021-08-31 | AUSTRAL-omics, UACh | AUSTRAL-omics, UACh | Cristian Molina, Suany Quesada,Andrea Silva, Luis GuzmÃ¡n, Carolina Encina, Daniela Plaza |
| EPI_ISL_4261449 | hCoV-19/Chile/LL-UACH-00155/2021 | South America/Chile/Los Lagos | 2021-09-07 | AUSTRAL-omics, UACh | AUSTRAL-omics, UACh | Cristian Molina, Suany Quesada,Andrea Silva, Luis GuzmÃ¡n, Carolina Encina, Daniela Plaza |
| EPI_ISL_4080688 | hCoV-19/Chile/LR-UACH-00119/2021 | South America/Chile/Los RÃ­os | 2021-08-22 | AUSTRAL-omics, UACh | AUSTRAL-omics, UACh | Cristian Molina, Suany Quesada,Andrea Silva, Luis GuzmÃ¡n, Carolina Encina, Daniela Plaza |
| EPI_ISL_2617643 | hCoV-19/Austria/CeMM11086/2021 | Europe/Austria/Vorarlberg | 2021-05-13 | Austrian Agency for Health and Food Safety (AGES) | Bergthaler laboratory, CeMM Research Center for Molecular Medicine of the Austrian Academy of Sciences | Lukas Endler, Anna Schedl, Fabian Amman, Petr Triska, Matthew Thornton, Thomas Penz, Benedikt Agerer, Maelle Le Moing, Michael Schuster, Bekir Erguner, Jan Laine, Martin Senekowitsch, Christoph Bock, Andreas Bergthaler |
| EPI_ISL_2617650 | hCoV-19/Austria/CeMM11093/2021 | Europe/Austria/Vorarlberg | 2021-05-13 | Austrian Agency for Health and Food Safety (AGES) | Bergthaler laboratory, CeMM Research Center for Molecular Medicine of the Austrian Academy of Sciences | Lukas Endler, Anna Schedl, Fabian Amman, Petr Triska, Matthew Thornton, Thomas Penz, Benedikt Agerer, Maelle Le Moing, Michael Schuster, Bekir Erguner, Jan Laine, Martin Senekowitsch, Christoph Bock, Andreas Bergthaler |
| EPI_ISL_694115 | hCoV-19/USA/AZ-TG276586/2020 | North America/USA/Arizona | 2020-04-16 | AZ SPHL, Arizona Department of Health Services | TGen North | Jolene Bowers, Megan Folkerts, Chris French, Hayley Yaglom, Ashlyn Pfeiffer, Darrin Lemmer, Dave Engelthaler, The Arizona COVID Genomics Union (ACGU) |
| EPI_ISL_694149 | hCoV-19/USA/AZ-TG280790/2020 | North America/USA/Arizona | 2020-04-17 | AZ SPHL, Arizona Department of Health Services | TGen North | Jolene Bowers, Megan Folkerts, Chris French, Hayley Yaglom, Ashlyn Pfeiffer, Darrin Lemmer, Dave Engelthaler, The Arizona COVID Genomics Union (ACGU) |
| EPI_ISL_882637 | hCoV-19/Azerbaijan/Aghayev-01/2021 | Europe/Azerbaijan | 2021-01-02 | Azerbaijan National Hematology Center Division of Medical Genetics | Azerbaijan National Hematology Center Division of Medical Genetics | Aghayev Agha Rza, BayramlÄ± Ramin |
| EPI_ISL_539886 | hCoV-19/Sweden/20-52480/2020 | Europe/Sweden/Halland | 2020-08-03 | Barnakuten | The Public Health Agency of Sweden | Anna-Malin Linde, Maria Lind Karlberg, Oskar Karlsson Lindsjo, Olov Svartstrom, Mattias Haukland, Reza Advani, Sandra Broddesson, Anna Risberg, Theresa Enkirch, Mia Brytting, Karin Tegmark-Wisell |
| EPI_ISL_2774269 | hCoV-19/USA/TX-BSWTemple-ILL-R12-0042/2021 | North America/USA/Texas | 2021-06-14 | Baylor Scott & White-Temple | Baylor Scott & White-Temple | Ari Rao, Linden Morales, Kimberly Walker, Marcus Volz, Shelby Johnson |
| EPI_ISL_415578 | hCoV-19/Canada/BC_13297/2020 | North America/Canada/British Columbia | 2020-02-27 | BCCDC Public Health Laboratory | BCCDC Public Health Laboratory | Harrigan, Prystajecky, Krajden, Lee, Kamelian, Lapointe, Choi, Hoang, Sekirov, Levett, Tyson, Snutch, Loman, Quick, Li, Gilmour |
| EPI_ISL_415588 | hCoV-19/Canada/BC_83109/2020 | North America/Canada/British Columbia | 2020-03-05 | BCCDC Public Health Laboratory | BCCDC Public Health Laboratory | Harrigan, Prystajecky, Krajden, Lee, Kamelian, Lapointe, Choi, Hoang, Sekirov, Levett, Tyson, Snutch, Loman, Quick, Li, Gilmour |
| EPI_ISL_968563 | hCoV-19/Canada/BC-BCCDC-3005/2020 | North America/Canada/British Columbia | 2020-08-10 | BCCDC Public Health Laboratory | BCCDC Public Health Laboratory | Prystajecky Natalie, Linda Hoang, Dan Fornika, John Tyson, Shannon Russell, Kim Macdonald, Kimia Kamelian, Ana Pacagnella, Corrinne Ng, Loretta Janz, Robert Azana Terry Snutch, Mel Krajden |
| EPI_ISL_2498326 | hCoV-19/Canada/BC-BCCDC-35569/2020 | North America/Canada/British Columbia | 2020-12-22 | BCCDC Public Health Laboratory | BCCDC Public Health Laboratory | Prystajecky Natalie, Linda Hoang, Dan Fornika, John Tyson, Shannon Russell, Kim Macdonald, Kimia Kamelian, Ana Pacagnella, Corrinne Ng, Loretta Janz, Robert Azana, Mel Krajden |
| EPI_ISL_968277 | hCoV-19/Canada/BC-BCCDC-3587/2020 | North America/Canada/British Columbia | 2020-06-29 | BCCDC Public Health Laboratory | BCCDC Public Health Laboratory | Prystajecky Natalie, Linda Hoang, Dan Fornika, John Tyson, Shannon Russell, Kim Macdonald, Kimia Kamelian, Ana Pacagnella, Corrinne Ng, Loretta Janz, Robert Azana Terry Snutch, Mel Krajden |
| EPI_ISL_2528966 | hCoV-19/Canada/BC-BCCDC-53384/2021 | North America/Canada/British Columbia | 2021-01-27 | BCCDC Public Health Laboratory | BCCDC Public Health Laboratory | Prystajecky Natalie, Linda Hoang, Dan Fornika, John Tyson, Shannon Russell, Kim Macdonald, Kimia Kamelian, Ana Pacagnella, Corrinne Ng, Loretta Janz, Robert Azana, Mel Krajden |
| EPI_ISL_2529012 | hCoV-19/Canada/BC-BCCDC-53421/2021 | North America/Canada/British Columbia | 2021-02-09 | BCCDC Public Health Laboratory | BCCDC Public Health Laboratory | Prystajecky Natalie, Linda Hoang, Dan Fornika, John Tyson, Shannon Russell, Kim Macdonald, Kimia Kamelian, Ana Pacagnella, Corrinne Ng, Loretta Janz, Robert Azana, Mel Krajden |
| EPI_ISL_2718924 | hCoV-19/Canada/BC-BCCDC-58339/2021 | North America/Canada/British Columbia | 2021-04-04 | BCCDC Public Health Laboratory | BCCDC Public Health Laboratory | Prystajecky Natalie, Linda Hoang, Dan Fornika, John Tyson, Shannon Russell, Kim Macdonald, Kimia Kamelian, Ana Pacagnella, Corrinne Ng, Loretta Janz, Robert Azana, Mel Krajden |
| EPI_ISL_2721129 | hCoV-19/Canada/BC-BCCDC-79218/2021 | North America/Canada/British Columbia | 2021-04-13 | BCCDC Public Health Laboratory | BCCDC Public Health Laboratory | Prystajecky Natalie, Linda Hoang, Dan Fornika, John Tyson, Shannon Russell, Kim Macdonald, Kimia Kamelian, Ana Pacagnella, Corrinne Ng, Loretta Janz, Robert Azana, Mel Krajden |
| EPI_ISL_3185949 | hCoV-19/USA/CA-NR-52509/2020 | North America/USA/California | 2020-01-22 | BEI Resources | Access DX Laboratory | Ekaterina Sermyagina |
| EPI_ISL_509713 | hCoV-19/Belize/un-CDC-3595771-001/2020 | North America/Belize/Belmopan | 2020-03-27 | Belize Ministry of Health | Pathogen Discovery, Respiratory Viruses Branch, Division of Viral Diseases, Centers for Disease Control and Prevention | Jing Zhang, Ying Tao, Krista Queen, Anna Uehara, Yan Li, Clinton Paden, Haibin Wang, Suxiang Tong |
| EPI_ISL_509714 | hCoV-19/Belize/un-CDC-3595772-001/2020 | North America/Belize/Belmopan | 2020-04-09 | Belize Ministry of Health | Pathogen Discovery, Respiratory Viruses Branch, Division of Viral Diseases, Centers for Disease Control and Prevention | Jing Zhang, Ying Tao, Krista Queen, Anna Uehara, Yan Li, Clinton Paden, Haibin Wang, Suxiang Tong |
| EPI_ISL_780384 | hCoV-19/Bermuda/204990926/2020 | North America/Bermuda | 2020-11-10 | Bermuda Government Molecular Diagnostics Laboratory (MDL) | Respiratory Virus Unit, National Infection Service, Public Health England | PHE Covid Sequencing Team, Dr Carika Weldon (Bermuda), Dr Ayoola Oyinloye (Bermuda) |
| EPI_ISL_780396 | hCoV-19/Bermuda/204990928/2020 | North America/Bermuda | 2020-11-13 | Bermuda Government Molecular Diagnostics Laboratory (MDL) | Respiratory Virus Unit, National Infection Service, Public Health England | PHE Covid Sequencing Team, Dr Carika Weldon (Bermuda), Dr Ayoola Oyinloye (Bermuda) |
| EPI_ISL_780408 | hCoV-19/Bermuda/205081299/2020 | North America/Bermuda | 2020-11-04 | Bermuda Government Molecular Diagnostics Laboratory (MDL) | Respiratory Virus Unit, National Infection Service, Public Health England | PHE Covid Sequencing Team, Dr Carika Weldon (Bermuda), Dr Ayoola Oyinloye (Bermuda) |
| EPI_ISL_924805 | hCoV-19/England/TFCI-2702001/2021 | Europe/United Kingdom/England | 2021-01-10 | Bioinformatics and Biostatistics Lab, Advanced Sequencing Facility | COVID-19 Genomics UK (COG-UK) Consortium | Aengus Stewart,Jerome Nicod,Chelsea Sawyer,Laura Cubitt,Harshil Patel,Margaret Crawford |
| EPI_ISL_1297375 | hCoV-19/France/ARA-HCL021023163701/2021 | Europe/France/Auvergne-Rhone-Alpes | 2021-01-29 | BIOMNIS LYON | CNR Virus des Infections Respiratoires - France SUD | Antonin Bal, Gregory Destras, Gwendolyne Burfin, Hadrien Regue, Quentin Semanas, Martine Valette, Bruno Lina, Laurence Josset |
| EPI_ISL_3145700 | hCoV-19/Luxembourg/LNS6279788/2021 | Europe/Luxembourg | 2021-06-01 | BioneXt Lab | Laboratoire national de sante, Microbiology, Microbial Genomics Platform | Anke Wienecke-Baldacchino, Catherine Ragimbeau,Jessica Tapp, Fatu Djabi, Lise Pignon, Raoul Salmon,Virginie Jover, Elodie Solarino, Thibault Ferrandon, Tamir Abdelrahman |
| EPI_ISL_2464486 | hCoV-19/France/GP-HMN-21052200197/2021 | North America/Guadeloupe | 2021-05-10 | Biopole Antilles | Department of Virology, Henri Mondor University Hospital, Assistance Publique HÃ´pitaux de Paris, UniversitÃ© Paris-Est CrÃ©teil, INSERM U955 | Christophe Rodriguez, Slim Fourati, Vanessa Demontant, Guillaume Gricourt, Melissa N'Debi, Alexandre Soulier, Elisabeth Trawinski, Jean-Michel Pawlotsky |
| EPI_ISL_2983867 | hCoV-19/France/GP-HMN-21072050394/2021 | North America/Guadeloupe | 2021-06-29 | Biopole Antilles | Department of Virology, Henri Mondor University Hospital, Assistance Publique HÃ´pitaux de Paris, UniversitÃ© Paris-Est CrÃ©teil, INSERM U955 | Christophe Rodriguez, Slim Fourati, Vanessa Demontant, Guillaume Gricourt, Melissa N'Debi, Alexandre Soulier, Elisabeth Trawinski, Jean-Michel Pawlotsky |
| EPI_ISL_2983948 | hCoV-19/France/GP-HMN-21072050408/2021 | North America/Guadeloupe | 2021-06-29 | Biopole Antilles | Department of Virology, Henri Mondor University Hospital, Assistance Publique HÃ´pitaux de Paris, UniversitÃ© Paris-Est CrÃ©teil, INSERM U955 | Christophe Rodriguez, Slim Fourati, Vanessa Demontant, Guillaume Gricourt, Melissa N'Debi, Alexandre Soulier, Elisabeth Trawinski, Jean-Michel Pawlotsky |
| EPI_ISL_2983853 | hCoV-19/France/GP-HMN-21072050409/2021 | North America/Guadeloupe | 2021-06-29 | Biopole Antilles | Department of Virology, Henri Mondor University Hospital, Assistance Publique HÃ´pitaux de Paris, UniversitÃ© Paris-Est CrÃ©teil, INSERM U955 | Christophe Rodriguez, Slim Fourati, Vanessa Demontant, Guillaume Gricourt, Melissa N'Debi, Alexandre Soulier, Elisabeth Trawinski, Jean-Michel Pawlotsky |
| EPI_ISL_3390947 | hCoV-19/Guadeloupe/GP-HMN-21072130434/2021 | North America/Guadeloupe | 2021-07-06 | Biopole Antilles | Department of Virology, Henri Mondor University Hospital, Assistance Publique HÃ´pitaux de Paris, UniversitÃ© Paris-Est CrÃ©teil, INSERM U955 | Christophe Rodriguez, Slim Fourati, Vanessa Demontant, Guillaume Gricourt, Melissa N'Debi, Alexandre Soulier, Elisabeth Trawinski, Jean-Michel Pawlotsky |
| EPI_ISL_955258 | hCoV-19/USA/NJ-PHEL-4553854/2021 | North America/USA/New Jersey | 2021-01-03 | BioReference Lab | NJ Public Health and Environmental Laboratories | Lindsey Bodnar, Shiv Verma, Dana Woell, Byeong Jeong |
| EPI_ISL_804946 | hCoV-19/USA/NY-Wadsworth-21001194-01/2020 | North America/USA/New York | 2020-12-27 | BIO-REFERENCE LABORATORIES | Wadsworth Center, New York State Department.of Health | Kirsten St. George, Daryl M. Lamson, Alexis Russel, Matthew Shudt, Melissa A Leisner, Jonathan Plitnick, Navjot Singh, John Kelly, Sara Griesemer, Erasmus Schneider, Erica Lasek-Nesselquist |
| EPI_ISL_804982 | hCoV-19/USA/NY-Wadsworth-21002419-01/2020 | North America/USA/New York | 2020-12-31 | BIO-REFERENCE LABORATORIES | Wadsworth Center, New York State Department of Health | Kirsten St. George, Daryl M. Lamson, Alexis Russel, Matthew Shudt, Melissa A Leisner, Jonathan Plitnick, Navjot Singh, John Kelly, Erasmus Schneider, Erica Lasek-Nesselquist |
| EPI_ISL_804983 | hCoV-19/USA/NY-Wadsworth-21002420-01/2020 | North America/USA/New York | 2020-12-31 | BIO-REFERENCE LABORATORIES | Wadsworth Center, New York State Department of Health | Kirsten St. George, Daryl M. Lamson, Alexis Russel, Matthew Shudt, Melissa A Leisner, Jonathan Plitnick, Navjot Singh, John Kelly, Erasmus Schneider, Erica Lasek-Nesselquist |
| EPI_ISL_861178 | hCoV-19/USA/NY-Wadsworth-21006732-01/2021 | North America/USA/New York | 2021-01-03 | BIO-REFERENCE LABORATORIES | Wadsworth Center, New York State Department of Health | Kirsten St. George, Daryl M. Lamson, Alexis Russel, Matthew Shudt, Melissa A Leisner, Jonathan Plitnick, Navjot Singh, John Kelly, Erasmus Schneider, Erica Lasek-Nesselquist |
| EPI_ISL_1226577 | hCoV-19/USA/NY-Wadsworth-21025870-01/2021 | North America/USA/New York | 2021-02-23 | BIO-REFERENCE LABORATORIES | Wadsworth Center, New York State Department of Health | Kirsten St. George, Daryl M. Lamson, Alexis Russel, Matthew Shudt, Melissa A Leisner, Jonathan Plitnick, Navjot Singh, John Kelly, Erasmus Schneider, Erica Lasek-Nesselquist |
| EPI_ISL_1397698 | hCoV-19/USA/NY-Wadsworth-21028900-01/2021 | North America/USA/New York | 2021-03-01 | BIO-REFERENCE LABORATORIES | Wadsworth Center, New York State Department of Health | Kirsten St. George, Daryl M. Lamson, Alexis Russel, Matthew Shudt, Melissa A Leisner, Jonathan Plitnick, Navjot Singh, John Kelly, Erasmus Schneider, Erica Lasek-Nesselquist |
| EPI_ISL_3364255 | hCoV-19/USA/NY-GEO-0079/2021 | North America/USA/New York | 2021-02-25 | Biotia | Biotia | Dorottya Nagy-Szakal, Mara Couto-Rodriguez, Xavier Jirau Serrano, Marilyne Debieu, David Danko, ChristopherÂ Mason, NiamhÂ Oâ€™Hara |
| EPI_ISL_3364462 | hCoV-19/USA/NY-GEO-0207/2021 | North America/USA/New York | 2021-01-04 | Biotia | Biotia | Dorottya Nagy-Szakal, Mara Couto-Rodriguez, Xavier Jirau Serrano, Marilyne Debieu, David Danko, ChristopherÂ Mason, NiamhÂ Oâ€™Hara |
| EPI_ISL_3364539 | hCoV-19/USA/NY-GEO-0231/2020 | North America/USA/New York | 2020-01-28 | Biotia | Biotia | Dorottya Nagy-Szakal, Mara Couto-Rodriguez, Xavier Jirau Serrano, Marilyne Debieu, David Danko, ChristopherÂ Mason, NiamhÂ Oâ€™Hara |
| EPI_ISL_3494198 | hCoV-19/USA/MA-NEIDL-00678/2021 | North America/USA/Massachusetts | 2021-01-14 | Boston University CTL | Boston University/National Emerging Infectious Disease Laboratories | Jacquelyn Turcinovic, John H. Connor, Lena Landeverde, Catherine Klapperich, Lynn Doucette-Stamm |
| EPI_ISL_3494199 | hCoV-19/USA/MA-NEIDL-00679/2021 | North America/USA/Massachusetts | 2021-01-14 | Boston University CTL | Boston University/National Emerging Infectious Disease Laboratories | Jacquelyn Turcinovic, John H. Connor, Lena Landeverde, Catherine Klapperich, Lynn Doucette-Stamm |
| EPI_ISL_3494364 | hCoV-19/USA/MA-NEIDL-00871/2020 | North America/USA/Massachusetts | 2020-10-31 | Boston University CTL | Boston University/National Emerging Infectious Disease Laboratories | Jacquelyn Turcinovic, John H. Connor, Lena Landeverde, Catherine Klapperich, Lynn Doucette-Stamm |
| EPI_ISL_4299816 | hCoV-19/Botswana/R31B90_BHP_000741037/2021 | Africa/Botswana/Gaborone | 2021-09-01 | Botswana Harvard AIDS Institute Partnership | Botswana Harvard HIV Reference Laboratory | Sikhulile Moyo, Wonderful T. Choga, Dorcas Maruapula, Keoratile Ntshambiwa, Sefetogi Ramaologa, Thongbotho Mphoyakgosi, Boitumelo Zuze, Botshelo Radibe, Legodile Kooepile, Ontlametse T. Bareng, Patrick T. Mokgethi, Pamela Smith-Lawrence, Kgomotso Moruisi, Roger Shapiro, Shahin Lockman, Joseph Makhema, Mphaphi B. Mbulawa, Mosepele Mosepele, Simani Gaseitsiwe |
| EPI_ISL_900726 | hCoV-19/USA/MT-BHDH-588/2020 | North America/USA/Montana | 2020-05-08 | Bozeman Health Deaconess Hospital | Wiedenheft lab, Montana State University | Artem Nemudryi, Anna Nemudraia, Tanner Wiegand, Joseph Nichols, Deann T. Snyder, Jodi F. Hedges, Calvin Cicha, Helen Lee, Karl K. Vanderwood, Diane Bimczok, Mark A. Jutila and Blake Wiedenheft |
| EPI_ISL_765589 | hCoV-19/USA/MA-MGH-02696/2020 | North America/USA/Massachusetts | 2020-09-27 | Brigham and Womens Hospital | Infectious Disease Program, Broad Institute of Harvard and MIT | Lemieux,J.E., Siddle,K.J., Shaw,B., Adams,G., Pierce,V., Turbett,S., Anahtar,M., Branda,J., Slater,D., Harris,J., Lin,A.E., Gladden-Young,A., Lagerborg,K., Rudy,M., DeRuff,K., Carter,A., Normandin,E., Bauer,M., Reilly,S., Tomkins-Tinch,C., Loreth,C., Chaluvadi,S., Neumann,A., Cusick,C., Chapman,S.B., Gnirke,A., Flowers,K., Cerrato,F., Birren,B.W., Gallagher,G., Smole,S., Park,D.J., MacInnis,B.L., Ryan,E., LaRocque,R., Rosenberg,E. and Sabeti,P.C. |
| EPI_ISL_765605 | hCoV-19/USA/MA-MGH-02732/2020 | North America/USA/Massachusetts | 2020-09-30 | Brigham and Womens Hospital | Infectious Disease Program, Broad Institute of Harvard and MIT | Lemieux,J.E., Siddle,K.J., Shaw,B., Adams,G., Pierce,V., Turbett,S., Anahtar,M., Branda,J., Slater,D., Harris,J., Lin,A.E., Gladden-Young,A., Lagerborg,K., Rudy,M., DeRuff,K., Carter,A., Normandin,E., Bauer,M., Reilly,S., Tomkins-Tinch,C., Loreth,C., Chaluvadi,S., Neumann,A., Cusick,C., Chapman,S.B., Gnirke,A., Flowers,K., Cerrato,F., Birren,B.W., Gallagher,G., Smole,S., Park,D.J., MacInnis,B.L., Ryan,E., LaRocque,R., Rosenberg,E. and Sabeti,P.C. |
| EPI_ISL_3479145 | hCoV-19/Canada/BC-BCCDC-103855/2021 | North America/Canada/British Columbia | 2021-06-01 | British Columbia Centre For Disease Control | BCCDC Public Health Laboratory | Prystajecky Natalie, Linda Hoang, Dan Fornika, John Tyson, Shannon Russell, Kim Macdonald, Kimia Kamelian, Ana Pacagnella, Corrinne Ng, Loretta Janz, Robert Azana, Mel Krajden |
| EPI_ISL_3602747 | hCoV-19/Canada/BC-BCCDC-134128/2021 | North America/Canada/British Columbia | 2021-07-04 | British Columbia Centre For Disease Control | BCCDC Public Health Laboratory | Prystajecky Natalie, Linda Hoang, Dan Fornika, John Tyson, Shannon Russell, Kim Macdonald, Kimia Kamelian, Ana Pacagnella, Corrinne Ng, Loretta Janz, Robert Azana, Mel Krajden |
| EPI_ISL_4277029 | hCoV-19/Canada/BC-BCCDC-180989/2021 | North America/Canada/British Columbia | 2021-08-19 | British Columbia Centre For Disease Control | BCCDC Public Health Laboratory | Prystajecky Natalie, Linda Hoang, Dan Fornika, John Tyson, Shannon Russell, Kim Macdonald, Kimia Kamelian, Ana Pacagnella, Corrinne Ng, Loretta Janz, Robert Azana, Mel Krajden |
| EPI_ISL_4278587 | hCoV-19/Canada/BC-BCCDC-183164/2021 | North America/Canada/British Columbia | 2021-08-23 | British Columbia Centre For Disease Control | BCCDC Public Health Laboratory | Prystajecky Natalie, Linda Hoang, Dan Fornika, John Tyson, Shannon Russell, Kim Macdonald, Kimia Kamelian, Ana Pacagnella, Corrinne Ng, Loretta Janz, Robert Azana, Mel Krajden |
| EPI_ISL_4278596 | hCoV-19/Canada/BC-BCCDC-183188/2021 | North America/Canada/British Columbia | 2021-08-23 | British Columbia Centre For Disease Control | BCCDC Public Health Laboratory | Prystajecky Natalie, Linda Hoang, Dan Fornika, John Tyson, Shannon Russell, Kim Macdonald, Kimia Kamelian, Ana Pacagnella, Corrinne Ng, Loretta Janz, Robert Azana, Mel Krajden |
| EPI_ISL_4093690 | hCoV-19/USA/CT-CDCBI-CRSP_DZQUNJFOE7SPRMJY/2021 | North America/USA/Connecticut | 2021-09-02 | Broad Institute Clinical Research Sequencing Platform | Infectious Disease Program, Broad Institute of Harvard and MIT | Siddle,K.J., Adams,G., Pearlman,L., Gladden-Young,A., Vicente,G., Blumenstiel,B., DeFelice,M., Lee,M., McGovern,S., Lagerborg,K., Rudy,M., DeRuff,K., Carter,A., Normandin,E., Bauer,M., Reilly,S., Tomkins-Tinch,C., Loreth,C., Chaluvadi,S., Meldrim,J., Granger,B., Lemieux,J.E., Birren,B.W., Sabeti,P.C., Larkin,K., Dodge,S., Lennon,N., Madoff,L., Brown,C., Gallagher,G., Smole,S., Park,D.J., Gabriel,S., and MacInnis,B.L. |
| EPI_ISL_1253858 | hCoV-19/USA/MA-Broad_CRSP-01307/2021 | North America/USA/Massachusetts | 2021-02-15 | Broad Institute Clinical Research Sequencing Platform | Infectious Disease Program, Broad Institute of Harvard and MIT | Lemieux,J.E., Siddle,K.J., Adams,G., Gladden-Young,A., Lagerborg,K., Rudy,M., DeRuff,K., Carter,A., Normandin,E., Bauer,M., Reilly,S., Tomkins-Tinch,C., Loreth,C., Chaluvadi,S., Birren,B.W., Gallagher,G., Smole,S., Park,D.J., MacInnis,B.L., and Sabeti,P.C. |
| EPI_ISL_1253876 | hCoV-19/USA/MA-Broad_CRSP-01329/2021 | North America/USA/Massachusetts | 2021-02-05 | Broad Institute Clinical Research Sequencing Platform | Infectious Disease Program, Broad Institute of Harvard and MIT | Lemieux,J.E., Siddle,K.J., Adams,G., Gladden-Young,A., Lagerborg,K., Rudy,M., DeRuff,K., Carter,A., Normandin,E., Bauer,M., Reilly,S., Tomkins-Tinch,C., Loreth,C., Chaluvadi,S., Birren,B.W., Gallagher,G., Smole,S., Park,D.J., MacInnis,B.L., and Sabeti,P.C. |
| EPI_ISL_1710155 | hCoV-19/USA/MA-CDCBI-CRSP_02256/2021 | North America/USA/Massachusetts | 2021-04-01 | Broad Institute Clinical Research Sequencing Platform | Infectious Disease Program, Broad Institute of Harvard and MIT | Siddle,K.J., Adams,G., Pearlman,L., Gladden-Young,A., Vicente,G., Blumenstiel,B., DeFelice,M., Lee,M., McGovern,S., Lagerborg,K., Rudy,M., DeRuff,K., Carter,A., Normandin,E., Bauer,M., Reilly,S., Tomkins-Tinch,C., Loreth,C., Chaluvadi,S., Meldrim,J., Granger,B., Lemieux,J.E., Birren,B.W., Sabeti,P.C., Larkin,K., Dodge,S., Lennon,N., Madoff,L., Brown,C., Gallagher,G., Smole,S., Park,D.J., Gabriel,S., and MacInnis,B.L. |
| EPI_ISL_1757079 | hCoV-19/USA/MA-CDCBI-CRSP_02423/2021 | North America/USA/Massachusetts | 2021-04-09 | Broad Institute Clinical Research Sequencing Platform | Infectious Disease Program, Broad Institute of Harvard and MIT | Siddle,K.J., Adams,G., Pearlman,L., Gladden-Young,A., Vicente,G., Blumenstiel,B., DeFelice,M., Lee,M., McGovern,S., Lagerborg,K., Rudy,M., DeRuff,K., Carter,A., Normandin,E., Bauer,M., Reilly,S., Tomkins-Tinch,C., Loreth,C., Chaluvadi,S., Meldrim,J., Granger,B., Lemieux,J.E., Birren,B.W., Sabeti,P.C., Larkin,K., Dodge,S., Lennon,N., Madoff,L., Brown,C., Gallagher,G., Smole,S., Park,D.J., Gabriel,S., and MacInnis,B.L. |
| EPI_ISL_4303904 | hCoV-19/USA/MA-CDCBI-CRSP_32F7XGQWIS5BA4IJ/2021 | North America/USA/Massachusetts | 2021-09-08 | Broad Institute Clinical Research Sequencing Platform | Infectious Disease Program, Broad Institute of Harvard and MIT | Siddle,K.J., Adams,G., Pearlman,L., Gladden-Young,A., Vicente,G., Blumenstiel,B., DeFelice,M., Lee,M., McGovern,S., Lagerborg,K., Rudy,M., DeRuff,K., Carter,A., Normandin,E., Bauer,M., Reilly,S., Tomkins-Tinch,C., Loreth,C., Chaluvadi,S., Meldrim,J., Granger,B., Lemieux,J.E., Birren,B.W., Sabeti,P.C., Larkin,K., Dodge,S., Lennon,N., Madoff,L., Brown,C., Gallagher,G., Smole,S., Park,D.J., Gabriel,S., and MacInnis,B.L. |
| EPI_ISL_1743752 | hCoV-19/USA/MA-CDCBI-CRSP_3FRTBTPVRMK7LJKI/2021 | North America/USA/Massachusetts | 2021-04-14 | Broad Institute Clinical Research Sequencing Platform | Infectious Disease Program, Broad Institute of Harvard and MIT | Siddle,K.J., Adams,G., Pearlman,L., Gladden-Young,A., Vicente,G., Blumenstiel,B., DeFelice,M., Lee,M., McGovern,S., Lagerborg,K., Rudy,M., DeRuff,K., Carter,A., Normandin,E., Bauer,M., Reilly,S., Tomkins-Tinch,C., Loreth,C., Chaluvadi,S., Meldrim,J., Granger,B., Lemieux,J.E., Birren,B.W., Sabeti,P.C., Larkin,K., Dodge,S., Lennon,N., Madoff,L., Brown,C., Gallagher,G., Smole,S., Park,D.J., Gabriel,S., and MacInnis,B.L. |
| EPI_ISL_4303963 | hCoV-19/USA/MA-CDCBI-CRSP_3GH6WD2P72LAKLV3/2021 | North America/USA/Massachusetts | 2021-09-10 | Broad Institute Clinical Research Sequencing Platform | Infectious Disease Program, Broad Institute of Harvard and MIT | Siddle,K.J., Adams,G., Pearlman,L., Gladden-Young,A., Vicente,G., Blumenstiel,B., DeFelice,M., Lee,M., McGovern,S., Lagerborg,K., Rudy,M., DeRuff,K., Carter,A., Normandin,E., Bauer,M., Reilly,S., Tomkins-Tinch,C., Loreth,C., Chaluvadi,S., Meldrim,J., Granger,B., Lemieux,J.E., Birren,B.W., Sabeti,P.C., Larkin,K., Dodge,S., Lennon,N., Madoff,L., Brown,C., Gallagher,G., Smole,S., Park,D.J., Gabriel,S., and MacInnis,B.L. |
| EPI_ISL_4093808 | hCoV-19/USA/MA-CDCBI-CRSP_3WDFXJLTJPJDWBFH/2021 | North America/USA/Massachusetts | 2021-09-03 | Broad Institute Clinical Research Sequencing Platform | Infectious Disease Program, Broad Institute of Harvard and MIT | Siddle,K.J., Adams,G., Pearlman,L., Gladden-Young,A., Vicente,G., Blumenstiel,B., DeFelice,M., Lee,M., McGovern,S., Lagerborg,K., Rudy,M., DeRuff,K., Carter,A., Normandin,E., Bauer,M., Reilly,S., Tomkins-Tinch,C., Loreth,C., Chaluvadi,S., Meldrim,J., Granger,B., Lemieux,J.E., Birren,B.W., Sabeti,P.C., Larkin,K., Dodge,S., Lennon,N., Madoff,L., Brown,C., Gallagher,G., Smole,S., Park,D.J., Gabriel,S., and MacInnis,B.L. |
| EPI_ISL_3007321 | hCoV-19/USA/MA-CDCBI-CRSP_4DGT7O5IKNWOOZRD/2021 | North America/USA/Massachusetts | 2021-06-29 | Broad Institute Clinical Research Sequencing Platform | Infectious Disease Program, Broad Institute of Harvard and MIT | Siddle,K.J., Adams,G., Pearlman,L., Gladden-Young,A., Vicente,G., Blumenstiel,B., DeFelice,M., Lee,M., McGovern,S., Lagerborg,K., Rudy,M., DeRuff,K., Carter,A., Normandin,E., Bauer,M., Reilly,S., Tomkins-Tinch,C., Loreth,C., Chaluvadi,S., Meldrim,J., Granger,B., Lemieux,J.E., Birren,B.W., Sabeti,P.C., Larkin,K., Dodge,S., Lennon,N., Madoff,L., Brown,C., Gallagher,G., Smole,S., Park,D.J., Gabriel,S., and MacInnis,B.L. |
| EPI_ISL_3407233 | hCoV-19/USA/MA-CDCBI-CRSP_526OFEGODCSJQCSP/2021 | North America/USA/Massachusetts | 2021-07-27 | Broad Institute Clinical Research Sequencing Platform | Infectious Disease Program, Broad Institute of Harvard and MIT | Siddle,K.J., Adams,G., Pearlman,L., Gladden-Young,A., Vicente,G., Blumenstiel,B., DeFelice,M., Lee,M., McGovern,S., Lagerborg,K., Rudy,M., DeRuff,K., Carter,A., Normandin,E., Bauer,M., Reilly,S., Tomkins-Tinch,C., Loreth,C., Chaluvadi,S., Meldrim,J., Granger,B., Lemieux,J.E., Birren,B.W., Sabeti,P.C., Larkin,K., Dodge,S., Lennon,N., Madoff,L., Brown,C., Gallagher,G., Smole,S., Park,D.J., Gabriel,S., and MacInnis,B.L. |
| EPI_ISL_4285254 | hCoV-19/USA/MA-CDCBI-CRSP_5Y2NWQXCFKC7MR7W/2021 | North America/USA/Massachusetts | 2021-09-08 | Broad Institute Clinical Research Sequencing Platform | Infectious Disease Program, Broad Institute of Harvard and MIT | Siddle,K.J., Adams,G., Pearlman,L., Gladden-Young,A., Vicente,G., Blumenstiel,B., DeFelice,M., Lee,M., McGovern,S., Lagerborg,K., Rudy,M., DeRuff,K., Carter,A., Normandin,E., Bauer,M., Reilly,S., Tomkins-Tinch,C., Loreth,C., Chaluvadi,S., Meldrim,J., Granger,B., Lemieux,J.E., Birren,B.W., Sabeti,P.C., Larkin,K., Dodge,S., Lennon,N., Madoff,L., Brown,C., Gallagher,G., Smole,S., Park,D.J., Gabriel,S., and MacInnis,B.L. |
| EPI_ISL_4304084 | hCoV-19/USA/MA-CDCBI-CRSP_AGBET24SKQDYDU63/2021 | North America/USA/Massachusetts | 2021-09-10 | Broad Institute Clinical Research Sequencing Platform | Infectious Disease Program, Broad Institute of Harvard and MIT | Siddle,K.J., Adams,G., Pearlman,L., Gladden-Young,A., Vicente,G., Blumenstiel,B., DeFelice,M., Lee,M., McGovern,S., Lagerborg,K., Rudy,M., DeRuff,K., Carter,A., Normandin,E., Bauer,M., Reilly,S., Tomkins-Tinch,C., Loreth,C., Chaluvadi,S., Meldrim,J., Granger,B., Lemieux,J.E., Birren,B.W., Sabeti,P.C., Larkin,K., Dodge,S., Lennon,N., Madoff,L., Brown,C., Gallagher,G., Smole,S., Park,D.J., Gabriel,S., and MacInnis,B.L. |
| EPI_ISL_4094271 | hCoV-19/USA/MA-CDCBI-CRSP_DZ6FUYONW4JMFYPE/2021 | North America/USA/Massachusetts | 2021-09-01 | Broad Institute Clinical Research Sequencing Platform | Infectious Disease Program, Broad Institute of Harvard and MIT | Siddle,K.J., Adams,G., Pearlman,L., Gladden-Young,A., Vicente,G., Blumenstiel,B., DeFelice,M., Lee,M., McGovern,S., Lagerborg,K., Rudy,M., DeRuff,K., Carter,A., Normandin,E., Bauer,M., Reilly,S., Tomkins-Tinch,C., Loreth,C., Chaluvadi,S., Meldrim,J., Granger,B., Lemieux,J.E., Birren,B.W., Sabeti,P.C., Larkin,K., Dodge,S., Lennon,N., Madoff,L., Brown,C., Gallagher,G., Smole,S., Park,D.J., Gabriel,S., and MacInnis,B.L. |
| EPI_ISL_4094398 | hCoV-19/USA/MA-CDCBI-CRSP_FJPI5JNAYI2OXOGM/2021 | North America/USA/Massachusetts | 2021-09-02 | Broad Institute Clinical Research Sequencing Platform | Infectious Disease Program, Broad Institute of Harvard and MIT | Siddle,K.J., Adams,G., Pearlman,L., Gladden-Young,A., Vicente,G., Blumenstiel,B., DeFelice,M., Lee,M., McGovern,S., Lagerborg,K., Rudy,M., DeRuff,K., Carter,A., Normandin,E., Bauer,M., Reilly,S., Tomkins-Tinch,C., Loreth,C., Chaluvadi,S., Meldrim,J., Granger,B., Lemieux,J.E., Birren,B.W., Sabeti,P.C., Larkin,K., Dodge,S., Lennon,N., Madoff,L., Brown,C., Gallagher,G., Smole,S., Park,D.J., Gabriel,S., and MacInnis,B.L. |
| EPI_ISL_4304222 | hCoV-19/USA/MA-CDCBI-CRSP_FLS4TM5YJ7QYI7ST/2021 | North America/USA/Massachusetts | 2021-09-10 | Broad Institute Clinical Research Sequencing Platform | Infectious Disease Program, Broad Institute of Harvard and MIT | Siddle,K.J., Adams,G., Pearlman,L., Gladden-Young,A., Vicente,G., Blumenstiel,B., DeFelice,M., Lee,M., McGovern,S., Lagerborg,K., Rudy,M., DeRuff,K., Carter,A., Normandin,E., Bauer,M., Reilly,S., Tomkins-Tinch,C., Loreth,C., Chaluvadi,S., Meldrim,J., Granger,B., Lemieux,J.E., Birren,B.W., Sabeti,P.C., Larkin,K., Dodge,S., Lennon,N., Madoff,L., Brown,C., Gallagher,G., Smole,S., Park,D.J., Gabriel,S., and MacInnis,B.L. |
| EPI_ISL_3007360 | hCoV-19/USA/MA-CDCBI-CRSP_FOYGWNMXCLFB4R4M/2021 | North America/USA/Massachusetts | 2021-06-29 | Broad Institute Clinical Research Sequencing Platform | Infectious Disease Program, Broad Institute of Harvard and MIT | Siddle,K.J., Adams,G., Pearlman,L., Gladden-Young,A., Vicente,G., Blumenstiel,B., DeFelice,M., Lee,M., McGovern,S., Lagerborg,K., Rudy,M., DeRuff,K., Carter,A., Normandin,E., Bauer,M., Reilly,S., Tomkins-Tinch,C., Loreth,C., Chaluvadi,S., Meldrim,J., Granger,B., Lemieux,J.E., Birren,B.W., Sabeti,P.C., Larkin,K., Dodge,S., Lennon,N., Madoff,L., Brown,C., Gallagher,G., Smole,S., Park,D.J., Gabriel,S., and MacInnis,B.L. |
| EPI_ISL_4285811 | hCoV-19/USA/MA-CDCBI-CRSP_HDODDN2QD6FAE453/2021 | North America/USA/Massachusetts | 2021-09-08 | Broad Institute Clinical Research Sequencing Platform | Infectious Disease Program, Broad Institute of Harvard and MIT | Siddle,K.J., Adams,G., Pearlman,L., Gladden-Young,A., Vicente,G., Blumenstiel,B., DeFelice,M., Lee,M., McGovern,S., Lagerborg,K., Rudy,M., DeRuff,K., Carter,A., Normandin,E., Bauer,M., Reilly,S., Tomkins-Tinch,C., Loreth,C., Chaluvadi,S., Meldrim,J., Granger,B., Lemieux,J.E., Birren,B.W., Sabeti,P.C., Larkin,K., Dodge,S., Lennon,N., Madoff,L., Brown,C., Gallagher,G., Smole,S., Park,D.J., Gabriel,S., and MacInnis,B.L. |
| EPI_ISL_4304298 | hCoV-19/USA/MA-CDCBI-CRSP_I37CQTT453PDZCRL/2021 | North America/USA/Massachusetts | 2021-09-10 | Broad Institute Clinical Research Sequencing Platform | Infectious Disease Program, Broad Institute of Harvard and MIT | Siddle,K.J., Adams,G., Pearlman,L., Gladden-Young,A., Vicente,G., Blumenstiel,B., DeFelice,M., Lee,M., McGovern,S., Lagerborg,K., Rudy,M., DeRuff,K., Carter,A., Normandin,E., Bauer,M., Reilly,S., Tomkins-Tinch,C., Loreth,C., Chaluvadi,S., Meldrim,J., Granger,B., Lemieux,J.E., Birren,B.W., Sabeti,P.C., Larkin,K., Dodge,S., Lennon,N., Madoff,L., Brown,C., Gallagher,G., Smole,S., Park,D.J., Gabriel,S., and MacInnis,B.L. |
| EPI_ISL_4286033 | hCoV-19/USA/MA-CDCBI-CRSP_JRW3GK2EC755452K/2021 | North America/USA/Massachusetts | 2021-09-05 | Broad Institute Clinical Research Sequencing Platform | Infectious Disease Program, Broad Institute of Harvard and MIT | Siddle,K.J., Adams,G., Pearlman,L., Gladden-Young,A., Vicente,G., Blumenstiel,B., DeFelice,M., Lee,M., McGovern,S., Lagerborg,K., Rudy,M., DeRuff,K., Carter,A., Normandin,E., Bauer,M., Reilly,S., Tomkins-Tinch,C., Loreth,C., Chaluvadi,S., Meldrim,J., Granger,B., Lemieux,J.E., Birren,B.W., Sabeti,P.C., Larkin,K., Dodge,S., Lennon,N., Madoff,L., Brown,C., Gallagher,G., Smole,S., Park,D.J., Gabriel,S., and MacInnis,B.L. |
| EPI_ISL_4304376 | hCoV-19/USA/MA-CDCBI-CRSP_JWWON3EFSC65XPL2/2021 | North America/USA/Massachusetts | 2021-09-10 | Broad Institute Clinical Research Sequencing Platform | Infectious Disease Program, Broad Institute of Harvard and MIT | Siddle,K.J., Adams,G., Pearlman,L., Gladden-Young,A., Vicente,G., Blumenstiel,B., DeFelice,M., Lee,M., McGovern,S., Lagerborg,K., Rudy,M., DeRuff,K., Carter,A., Normandin,E., Bauer,M., Reilly,S., Tomkins-Tinch,C., Loreth,C., Chaluvadi,S., Meldrim,J., Granger,B., Lemieux,J.E., Birren,B.W., Sabeti,P.C., Larkin,K., Dodge,S., Lennon,N., Madoff,L., Brown,C., Gallagher,G., Smole,S., Park,D.J., Gabriel,S., and MacInnis,B.L. |
| EPI_ISL_4285689 | hCoV-19/USA/MA-CDCBI-CRSP_LDXZQVJGNRHE26SL/2021 | North America/USA/Massachusetts | 2021-09-09 | Broad Institute Clinical Research Sequencing Platform | Infectious Disease Program, Broad Institute of Harvard and MIT | Siddle,K.J., Adams,G., Pearlman,L., Gladden-Young,A., Vicente,G., Blumenstiel,B., DeFelice,M., Lee,M., McGovern,S., Lagerborg,K., Rudy,M., DeRuff,K., Carter,A., Normandin,E., Bauer,M., Reilly,S., Tomkins-Tinch,C., Loreth,C., Chaluvadi,S., Meldrim,J., Granger,B., Lemieux,J.E., Birren,B.W., Sabeti,P.C., Larkin,K., Dodge,S., Lennon,N., Madoff,L., Brown,C., Gallagher,G., Smole,S., Park,D.J., Gabriel,S., and MacInnis,B.L. |
| EPI_ISL_4095059 | hCoV-19/USA/MA-CDCBI-CRSP_MCTWET7REVGCIRHI/2021 | North America/USA/Massachusetts | 2021-09-03 | Broad Institute Clinical Research Sequencing Platform | Infectious Disease Program, Broad Institute of Harvard and MIT | Siddle,K.J., Adams,G., Pearlman,L., Gladden-Young,A., Vicente,G., Blumenstiel,B., DeFelice,M., Lee,M., McGovern,S., Lagerborg,K., Rudy,M., DeRuff,K., Carter,A., Normandin,E., Bauer,M., Reilly,S., Tomkins-Tinch,C., Loreth,C., Chaluvadi,S., Meldrim,J., Granger,B., Lemieux,J.E., Birren,B.W., Sabeti,P.C., Larkin,K., Dodge,S., Lennon,N., Madoff,L., Brown,C., Gallagher,G., Smole,S., Park,D.J., Gabriel,S., and MacInnis,B.L. |
| EPI_ISL_4094922 | hCoV-19/USA/MA-CDCBI-CRSP_NLTW3MX3BCJ7J5P3/2021 | North America/USA/Massachusetts | 2021-09-02 | Broad Institute Clinical Research Sequencing Platform | Infectious Disease Program, Broad Institute of Harvard and MIT | Siddle,K.J., Adams,G., Pearlman,L., Gladden-Young,A., Vicente,G., Blumenstiel,B., DeFelice,M., Lee,M., McGovern,S., Lagerborg,K., Rudy,M., DeRuff,K., Carter,A., Normandin,E., Bauer,M., Reilly,S., Tomkins-Tinch,C., Loreth,C., Chaluvadi,S., Meldrim,J., Granger,B., Lemieux,J.E., Birren,B.W., Sabeti,P.C., Larkin,K., Dodge,S., Lennon,N., Madoff,L., Brown,C., Gallagher,G., Smole,S., Park,D.J., Gabriel,S., and MacInnis,B.L. |
| EPI_ISL_4095057 | hCoV-19/USA/MA-CDCBI-CRSP_O2XTLZUCWG3LKEIU/2021 | North America/USA/Massachusetts | 2021-09-02 | Broad Institute Clinical Research Sequencing Platform | Infectious Disease Program, Broad Institute of Harvard and MIT | Siddle,K.J., Adams,G., Pearlman,L., Gladden-Young,A., Vicente,G., Blumenstiel,B., DeFelice,M., Lee,M., McGovern,S., Lagerborg,K., Rudy,M., DeRuff,K., Carter,A., Normandin,E., Bauer,M., Reilly,S., Tomkins-Tinch,C., Loreth,C., Chaluvadi,S., Meldrim,J., Granger,B., Lemieux,J.E., Birren,B.W., Sabeti,P.C., Larkin,K., Dodge,S., Lennon,N., Madoff,L., Brown,C., Gallagher,G., Smole,S., Park,D.J., Gabriel,S., and MacInnis,B.L. |
| EPI_ISL_4095250 | hCoV-19/USA/MA-CDCBI-CRSP_OCE3R4RW44Z22A5O/2021 | North America/USA/Massachusetts | 2021-09-03 | Broad Institute Clinical Research Sequencing Platform | Infectious Disease Program, Broad Institute of Harvard and MIT | Siddle,K.J., Adams,G., Pearlman,L., Gladden-Young,A., Vicente,G., Blumenstiel,B., DeFelice,M., Lee,M., McGovern,S., Lagerborg,K., Rudy,M., DeRuff,K., Carter,A., Normandin,E., Bauer,M., Reilly,S., Tomkins-Tinch,C., Loreth,C., Chaluvadi,S., Meldrim,J., Granger,B., Lemieux,J.E., Birren,B.W., Sabeti,P.C., Larkin,K., Dodge,S., Lennon,N., Madoff,L., Brown,C., Gallagher,G., Smole,S., Park,D.J., Gabriel,S., and MacInnis,B.L. |
| EPI_ISL_3407138 | hCoV-19/USA/MA-CDCBI-CRSP_RWXOLYDZC3EX4QOO/2021 | North America/USA/Massachusetts | 2021-07-27 | Broad Institute Clinical Research Sequencing Platform | Infectious Disease Program, Broad Institute of Harvard and MIT | Siddle,K.J., Adams,G., Pearlman,L., Gladden-Young,A., Vicente,G., Blumenstiel,B., DeFelice,M., Lee,M., McGovern,S., Lagerborg,K., Rudy,M., DeRuff,K., Carter,A., Normandin,E., Bauer,M., Reilly,S., Tomkins-Tinch,C., Loreth,C., Chaluvadi,S., Meldrim,J., Granger,B., Lemieux,J.E., Birren,B.W., Sabeti,P.C., Larkin,K., Dodge,S., Lennon,N., Madoff,L., Brown,C., Gallagher,G., Smole,S., Park,D.J., Gabriel,S., and MacInnis,B.L. |
| EPI_ISL_4095328 | hCoV-19/USA/MA-CDCBI-CRSP_S6WBHNJA56TGKSQZ/2021 | North America/USA/Massachusetts | 2021-09-03 | Broad Institute Clinical Research Sequencing Platform | Infectious Disease Program, Broad Institute of Harvard and MIT | Siddle,K.J., Adams,G., Pearlman,L., Gladden-Young,A., Vicente,G., Blumenstiel,B., DeFelice,M., Lee,M., McGovern,S., Lagerborg,K., Rudy,M., DeRuff,K., Carter,A., Normandin,E., Bauer,M., Reilly,S., Tomkins-Tinch,C., Loreth,C., Chaluvadi,S., Meldrim,J., Granger,B., Lemieux,J.E., Birren,B.W., Sabeti,P.C., Larkin,K., Dodge,S., Lennon,N., Madoff,L., Brown,C., Gallagher,G., Smole,S., Park,D.J., Gabriel,S., and MacInnis,B.L. |
| EPI_ISL_4304728 | hCoV-19/USA/MA-CDCBI-CRSP_UG26NMOZPUJUHBZM/2021 | North America/USA/Massachusetts | 2021-09-10 | Broad Institute Clinical Research Sequencing Platform | Infectious Disease Program, Broad Institute of Harvard and MIT | Siddle,K.J., Adams,G., Pearlman,L., Gladden-Young,A., Vicente,G., Blumenstiel,B., DeFelice,M., Lee,M., McGovern,S., Lagerborg,K., Rudy,M., DeRuff,K., Carter,A., Normandin,E., Bauer,M., Reilly,S., Tomkins-Tinch,C., Loreth,C., Chaluvadi,S., Meldrim,J., Granger,B., Lemieux,J.E., Birren,B.W., Sabeti,P.C., Larkin,K., Dodge,S., Lennon,N., Madoff,L., Brown,C., Gallagher,G., Smole,S., Park,D.J., Gabriel,S., and MacInnis,B.L. |
| EPI_ISL_4095433 | hCoV-19/USA/MA-CDCBI-CRSP_UNEADGAXRRLIDZK6/2021 | North America/USA/Massachusetts | 2021-09-02 | Broad Institute Clinical Research Sequencing Platform | Infectious Disease Program, Broad Institute of Harvard and MIT | Siddle,K.J., Adams,G., Pearlman,L., Gladden-Young,A., Vicente,G., Blumenstiel,B., DeFelice,M., Lee,M., McGovern,S., Lagerborg,K., Rudy,M., DeRuff,K., Carter,A., Normandin,E., Bauer,M., Reilly,S., Tomkins-Tinch,C., Loreth,C., Chaluvadi,S., Meldrim,J., Granger,B., Lemieux,J.E., Birren,B.W., Sabeti,P.C., Larkin,K., Dodge,S., Lennon,N., Madoff,L., Brown,C., Gallagher,G., Smole,S., Park,D.J., Gabriel,S., and MacInnis,B.L. |
| EPI_ISL_4095583 | hCoV-19/USA/MA-CDCBI-CRSP_WHZYEDE4AOFZK4F6/2021 | North America/USA/Massachusetts | 2021-09-03 | Broad Institute Clinical Research Sequencing Platform | Infectious Disease Program, Broad Institute of Harvard and MIT | Siddle,K.J., Adams,G., Pearlman,L., Gladden-Young,A., Vicente,G., Blumenstiel,B., DeFelice,M., Lee,M., McGovern,S., Lagerborg,K., Rudy,M., DeRuff,K., Carter,A., Normandin,E., Bauer,M., Reilly,S., Tomkins-Tinch,C., Loreth,C., Chaluvadi,S., Meldrim,J., Granger,B., Lemieux,J.E., Birren,B.W., Sabeti,P.C., Larkin,K., Dodge,S., Lennon,N., Madoff,L., Brown,C., Gallagher,G., Smole,S., Park,D.J., Gabriel,S., and MacInnis,B.L. |
| EPI_ISL_4304814 | hCoV-19/USA/MA-CDCBI-CRSP_XVUX66MAPS5UEUH6/2021 | North America/USA/Massachusetts | 2021-09-11 | Broad Institute Clinical Research Sequencing Platform | Infectious Disease Program, Broad Institute of Harvard and MIT | Siddle,K.J., Adams,G., Pearlman,L., Gladden-Young,A., Vicente,G., Blumenstiel,B., DeFelice,M., Lee,M., McGovern,S., Lagerborg,K., Rudy,M., DeRuff,K., Carter,A., Normandin,E., Bauer,M., Reilly,S., Tomkins-Tinch,C., Loreth,C., Chaluvadi,S., Meldrim,J., Granger,B., Lemieux,J.E., Birren,B.W., Sabeti,P.C., Larkin,K., Dodge,S., Lennon,N., Madoff,L., Brown,C., Gallagher,G., Smole,S., Park,D.J., Gabriel,S., and MacInnis,B.L. |
| EPI_ISL_4286119 | hCoV-19/USA/MA-CDCBI-CRSP_XYFBQIPWDXB5EULY/2021 | North America/USA/Massachusetts | 2021-09-09 | Broad Institute Clinical Research Sequencing Platform | Infectious Disease Program, Broad Institute of Harvard and MIT | Siddle,K.J., Adams,G., Pearlman,L., Gladden-Young,A., Vicente,G., Blumenstiel,B., DeFelice,M., Lee,M., McGovern,S., Lagerborg,K., Rudy,M., DeRuff,K., Carter,A., Normandin,E., Bauer,M., Reilly,S., Tomkins-Tinch,C., Loreth,C., Chaluvadi,S., Meldrim,J., Granger,B., Lemieux,J.E., Birren,B.W., Sabeti,P.C., Larkin,K., Dodge,S., Lennon,N., Madoff,L., Brown,C., Gallagher,G., Smole,S., Park,D.J., Gabriel,S., and MacInnis,B.L. |
| EPI_ISL_4095829 | hCoV-19/USA/MA-CDCBI-CRSP_Y4HVXMYD3D2XX3VY/2021 | North America/USA/Massachusetts | 2021-09-01 | Broad Institute Clinical Research Sequencing Platform | Infectious Disease Program, Broad Institute of Harvard and MIT | Siddle,K.J., Adams,G., Pearlman,L., Gladden-Young,A., Vicente,G., Blumenstiel,B., DeFelice,M., Lee,M., McGovern,S., Lagerborg,K., Rudy,M., DeRuff,K., Carter,A., Normandin,E., Bauer,M., Reilly,S., Tomkins-Tinch,C., Loreth,C., Chaluvadi,S., Meldrim,J., Granger,B., Lemieux,J.E., Birren,B.W., Sabeti,P.C., Larkin,K., Dodge,S., Lennon,N., Madoff,L., Brown,C., Gallagher,G., Smole,S., Park,D.J., Gabriel,S., and MacInnis,B.L. |
| EPI_ISL_4286617 | hCoV-19/USA/MA-CDCBI-CRSP_Y5ZTRD6AHF467Z2V/2021 | North America/USA/Massachusetts | 2021-09-05 | Broad Institute Clinical Research Sequencing Platform | Infectious Disease Program, Broad Institute of Harvard and MIT | Siddle,K.J., Adams,G., Pearlman,L., Gladden-Young,A., Vicente,G., Blumenstiel,B., DeFelice,M., Lee,M., McGovern,S., Lagerborg,K., Rudy,M., DeRuff,K., Carter,A., Normandin,E., Bauer,M., Reilly,S., Tomkins-Tinch,C., Loreth,C., Chaluvadi,S., Meldrim,J., Granger,B., Lemieux,J.E., Birren,B.W., Sabeti,P.C., Larkin,K., Dodge,S., Lennon,N., Madoff,L., Brown,C., Gallagher,G., Smole,S., Park,D.J., Gabriel,S., and MacInnis,B.L. |
| EPI_ISL_4095983 | hCoV-19/USA/RI-CDCBI-CRSP_4F4IACAUW6S7N3AY/2021 | North America/USA/Rhode Island | 2021-09-02 | Broad Institute Clinical Research Sequencing Platform | Infectious Disease Program, Broad Institute of Harvard and MIT | Siddle,K.J., Adams,G., Pearlman,L., Gladden-Young,A., Vicente,G., Blumenstiel,B., DeFelice,M., Lee,M., McGovern,S., Lagerborg,K., Rudy,M., DeRuff,K., Carter,A., Normandin,E., Bauer,M., Reilly,S., Tomkins-Tinch,C., Loreth,C., Chaluvadi,S., Meldrim,J., Granger,B., Lemieux,J.E., Birren,B.W., Sabeti,P.C., Larkin,K., Dodge,S., Lennon,N., Madoff,L., Brown,C., Gallagher,G., Smole,S., Park,D.J., Gabriel,S., and MacInnis,B.L. |
| EPI_ISL_4096397 | hCoV-19/USA/VT-CDCBI-CRSP_WT7TP35XOXM6O5SZ/2021 | North America/USA/Vermont | 2021-09-01 | Broad Institute Clinical Research Sequencing Platform | Infectious Disease Program, Broad Institute of Harvard and MIT | Siddle,K.J., Adams,G., Pearlman,L., Gladden-Young,A., Vicente,G., Blumenstiel,B., DeFelice,M., Lee,M., McGovern,S., Lagerborg,K., Rudy,M., DeRuff,K., Carter,A., Normandin,E., Bauer,M., Reilly,S., Tomkins-Tinch,C., Loreth,C., Chaluvadi,S., Meldrim,J., Granger,B., Lemieux,J.E., Birren,B.W., Sabeti,P.C., Larkin,K., Dodge,S., Lennon,N., Madoff,L., Brown,C., Gallagher,G., Smole,S., Park,D.J., Gabriel,S., and MacInnis,B.L. |
| EPI_ISL_732541 | hCoV-19/Germany/BY-IMB00234/2020 | Europe/Germany/Bavaria | 2020-02-01 | Bundeswehr Institute of Microbiology | Bundeswehr Institute of Microbiology | Markus Antwerpen, Alexandra Rehn, Mathias Walter, Malena Bestehorn-Willmann, Sabine Zange, Enrico Georgi, Roman WÃ¶lfel |
| EPI_ISL_979286 | hCoV-19/Canada/MB-NML-1013/2020 | North America/Canada/Manitoba | 2020-03-29 | Cadham Provincial laboratory | National Microbiology Laboratory (NML) | Anna Majer, Shari Tyson, Grace Seo, Philip Mabon, Elsie Grudeski, Rhiannon Huzarewich, Russell Mandes, Anneliese Landgraff, Jennifer Tanner, Natalie Knox, Morag Graham, Gary Van Domselaar, Paul Van Caeseele, Jared Bullard, David Alexander, Kerry Dust, Nathalie Bastien, Yan Li, Timothy Booth, Darian Hole, Madison Chapel, Kirsten Biggar, CanCOGeN's metadata curation team, Public Health Agency of Canada CanCOGeN team |
| EPI_ISL_408009 | hCoV-19/USA/CA-CDC-03039595-001/2020 | North America/USA/California | 2020-01-29 | California Department of Health | Pathogen Discovery, Respiratory Viruses Branch, Division of Viral Diseases, Centers for Dieases Control and Prevention | Krista Queen, Jing Zhang, Yan Li, Ying Tao, Anna Uehara, Clinton Paden, Xiaoyan Lu, Brian Lynch, Senthil Kumar K. Sakthivel, Brett L. Whitaker, Shifaq Kamili, Lijuan Wang, Janna' R. Murray, Susan I. Gerber, Stephen Lindstrom, Suxiang Tong |
| EPI_ISL_408008 | hCoV-19/USA/CA-CDC-03039618-001/2020 | North America/USA/California | 2020-01-29 | California Department of Health | Pathogen Discovery, Respiratory Viruses Branch, Division of Viral Diseases, Centers for Disease Control and Prevention | Krista Queen, Jing Zhang, Yan Li, Ying Tao, Anna Uehara, Clinton Paden, Xiaoyan Lu, Brian Lynch, Senthil Kumar K. Sakthivel, Brett L. Whitaker, Shifaq Kamili, Lijuan Wang, Janna' R. Murray, Susan I. Gerber, Stephen Lindstrom, Suxiang Tong |
| EPI_ISL_408010 | hCoV-19/USA/CA-CDC-03040142-001/2020 | North America/USA/California | 2020-01-29 | California Department of Health | Pathogen Discovery, Respiratory Viruses Branch, Division of Viral Diseases, Centers for Dieases Control and Prevention | Ying Tao, Krista Queen, Jing Zhang, Yan Li, Anna Uehara, Clinton Paden, Xiaoyan Lu, Brian Lynch, Senthil Kumar K. Sakthivel, Brett L. Whitaker, Shifaq Kamili, Lijuan Wang, Janna' R. Murray, Susan I. Gerber, Stephen Lindstrom, Suxiang Tong |
| EPI_ISL_406036 | hCoV-19/USA/CA-CDC-02993506-001/2020 | North America/USA/California | 2020-01-22 | California Department of Public Health | Pathogen Discovery, Respiratory Viruses Branch, Division of Viral Diseases, Centers for Dieases Control and Prevention | Anna Uehara, Krista Queen, Ying Tao, Yan Li, Clinton R. Paden, Jing Zhang, Xiaoyan Lu, Brian Lynch, Senthil Kumar K. Sakthivel, Brett L. Whitaker, Shifaq Kamili, Lijuan Wang, Janna' R. Murray, Susan I. Gerber, Stephen Lindstrom, Suxiang Tong |
| EPI_ISL_410044 | hCoV-19/USA/CA-CDC-6/2020 | North America/USA/California | 2020-01-27 | California Department of Public Health | Pathogen Discovery, Respiratory Viruses Branch, Division of Viral Diseases, Centers for Dieases Control and Prevention | Jing Zhang, Krista Queen, Yan Li, Ying Tao, Anna Uehara, Clinton R. Paden, Xiaoyan Lu, Brian Lynch, Senthil Kumar K. Sakthivel, Brett L. Whitaker, Shifaq Kamili, Lijuan Wang, Janna' R. Murray, Susan I. Gerber, Stephen Lindstrom, Suxiang Tong |
| EPI_ISL_755022 | hCoV-19/USA/CA-CDPH063/2020 | North America/USA/California | 2020-03-27 | California Department of Public Health | California Department of Public Health | CDPH IDLB COVIDNet |
| EPI_ISL_3537059 | hCoV-19/USA/CA-CDPH1180/2020 | North America/USA/California | 2020-01-03 | California Department of Public Health | California Department of Public Health | CDPH IDLB COVIDNet et al |
| EPI_ISL_3537061 | hCoV-19/USA/CA-CDPH1182/2020 | North America/USA/California | 2020-01-03 | California Department of Public Health | California Department of Public Health | CDPH IDLB COVIDNet et al |
| EPI_ISL_3537065 | hCoV-19/USA/CA-CDPH1186/2020 | North America/USA/California | 2020-01-03 | California Department of Public Health | California Department of Public Health | CDPH IDLB COVIDNet et al |
| EPI_ISL_3537066 | hCoV-19/USA/CA-CDPH1187/2020 | North America/USA/California | 2020-01-03 | California Department of Public Health | California Department of Public Health | CDPH IDLB COVIDNet et al |
| EPI_ISL_3537067 | hCoV-19/USA/CA-CDPH1188/2020 | North America/USA/California | 2020-01-03 | California Department of Public Health | California Department of Public Health | CDPH IDLB COVIDNet et al |
| EPI_ISL_413926 | hCoV-19/USA/CA-CDPH-UC8/2020 | North America/USA/California | 2020-03-05 | California Department of Public Health | Chiu Laboratory, University of California, San Francisco | Xianding Deng, Scot Federman, Chao-Yang Pan, Hugo Guevara,Wei Gu, Debra A. Wadford, and Charles Y. Chiu |
| EPI_ISL_2778233 | hCoV-19/USA/CA-CDPH-2000035454/2021 | North America/USA/California | 2021-02-03 | California Department of Public Health Valencia Branch Laboratory (CDPH VBL) | California Department of Public Health | CDPH-COVIDNet,UCLA Technology Center for Genomics & Bioinformatics |
| EPI_ISL_977658 | hCoV-19/Barbados/BRB50400/2020 | North America/Barbados | 2020-12-31 | Caribbean Public Health Agency | Carrington Lab, Department of PreClinical Sciences, Building 36, First Floor Biochemistry Unit, Faculty of Medical Sciences, The University of the West Indies | Nikita S. D. Sahadeo, Arianne Brown-Jordan, Vernie Ramkissoon, Sarah Hill, Naresh Nandram, Avery Hinds, Kenneth George, Jerome Foster, Stanley Giddings, Karla Georges, Marsha Ivey, Rahul Naidu, Risha Singh, SueMin Nathaniel, Rajini Haraksingh, Jaya Jayaraman, Chinna Chinnadurai, Adesh Ramsubhag, Nuno Faria, Oliver Pybus, Christopher Oura, Gabriel Escobar, Christine V. F. Carrington |
| EPI_ISL_977659 | hCoV-19/Barbados/BRB50403/2021 | North America/Barbados | 2021-01-03 | Caribbean Public Health Agency | Carrington Lab, Department of PreClinical Sciences, Building 36, First Floor Biochemistry Unit, Faculty of Medical Sciences, The University of the West Indies | Nikita S. D. Sahadeo, Arianne Brown-Jordan, Vernie Ramkissoon, Sarah Hill, Naresh Nandram, Avery Hinds, Kenneth George, Jerome Foster, Stanley Giddings, Karla Georges, Marsha Ivey, Rahul Naidu, Risha Singh, SueMin Nathaniel, Rajini Haraksingh, Jaya Jayaraman, Chinna Chinnadurai, Adesh Ramsubhag, Nuno Faria, Oliver Pybus, Christopher Oura, Gabriel Escobar, Christine V. F. Carrington |
| EPI_ISL_872195 | hCoV-19/SaintLucia/LCA47897/2020 | North America/Saint Lucia/ | 2020-12-23 | Caribbean Public Health Agency | Carrington Lab, Department of PreClinical Sciences, Faculty of Medical Sciences, The University of the West Indies | Nikita S. D. Sahadeo, Arianne Brown-Jordan, Vernie Ramkissoon, Sarah Hill, Naresh Nandram, Avery Hinds, Jerome Foster, Stanley Giddings, Karla Georges, Marsha Ivey, Rahul Naidu, Risha Singh, SueMin Nathaniel, Rajini Haraksingh, Jaya Jayaraman, Chinna Chinnadurai, Adesh Ramsubhag, Nuno Faria, Oliver Pybus, Christopher Oura, Gabriel Escobar, Christine V. F. Carrington |
| EPI_ISL_872196 | hCoV-19/SaintLucia/LCA47987/2020 | North America/Saint Lucia/ | 2020-12-17 | Caribbean Public Health Agency | Carrington Lab, Department of PreClinical Sciences, Faculty of Medical Sciences, The University of the West Indies | Nikita S. D. Sahadeo, Arianne Brown-Jordan, Vernie Ramkissoon, Sarah Hill, Naresh Nandram, Avery Hinds, Jerome Foster, Stanley Giddings, Karla Georges, Marsha Ivey, Rahul Naidu, Risha Singh, SueMin Nathaniel, Rajini Haraksingh, Jaya Jayaraman, Chinna Chinnadurai, Adesh Ramsubhag, Nuno Faria, Oliver Pybus, Christopher Oura, Gabriel Escobar, Christine V. F. Carrington |
| EPI_ISL_977538 | hCoV-19/SaintLucia/SLU51389/2021 | North America/Saint Lucia/ | 2021-01-18 | Caribbean Public Health Agency | Carrington Lab, Department of PreClinical Sciences | Nikita S. D. Sahadeo, Arianne Brown-Jordan, Vernie Ramkissoon, Sarah Hill, Naresh Nandram, Dr. Sharon Belmar-George Avery Hinds, Jerome Foster, Stanley Giddings, Karla Georges, Marsha Ivey, Rahul Naidu, Risha Singh, SueMin Nathaniel, Rajini Haraksingh, Jaya Jayaraman, Chinna Chinnadurai, Adesh Ramsubhag, Nuno Faria, Oliver Pybus, Christopher Oura, Gabriel Escobar, Christine V. F. Carrington |
| EPI_ISL_1588899 | hCoV-19/TrinidadandTobago/60336/2021 | South America/Trinidad and Tobago/ | 2021-03-23 | Caribbean Public Health Agency | Carrington Lab, Department of PreClinical Sciences, Faculty of Medical Sciences, The University of the West Indies | Nikita S. D. Sahadeo, Arianne Brown-Jordan, Sarah Hill, Vernie Ramkissoon, Roshan Parasram, Naresh Nandram, Avery Hinds, Jerome Foster, Stanley Giddings, Karla Georges, Marsha Ivey, Rahul Naidu, Risha Singh, SueMin Nathaniel, Rajini Haraksingh, Jaya Jayaraman, Chinna Chinnadurai, Adesh Ramsubhag, Nuno Faria, Oliver Pybus, Christopher Oura, Gabriel Escobar, Christine V. F. Carrington |
| EPI_ISL_3047303 | hCoV-19/USA/GA-CDC-4192716-001/2021 | North America/USA/Georgia | 2021-02-08 | CDC Dengue Branch | Genomics and Discovery, Respiratory Viruses Branch, Division of Viral Diseases, Centers for Disease Control and Prevention | Jing Zhang, Anna Kelleher, Ying Tao, Yan Li, Brian Lynch, Anna Uehara, Peter Cook, Han Jia Justin Ng, Rachel Marine, Clinton R. Paden, Dhwani Batra, Haibin Wang, Tara Coalter, Jasmine Padilla, Morgan Davis, Mili Sheth, Sarah Nobles, Mark Burroughs, Justin Lee, Adam Retchless, Suxiang Tong |
| EPI_ISL_3637250 | hCoV-19/USA/CA-CDPH-2000007562/2020 | North America/USA/California | 2020-12-10 | CDPH VBL | California Department of Public Health | Emily Smith on behalf of CDPH-COVIDNet and UCI Genome Sciences Center/GHTF |
| EPI_ISL_3671677 | hCoV-19/USA/CA-CDPH-2000020295/2021 | North America/USA/California | 2021-01-05 | CDPH VBL | California Department of Public Health | Emily Smith on behalf of CDPH-COVIDNet and UCSD EXCITE lab |
| EPI_ISL_3244212 | hCoV-19/USA/CA-CDPH-2000048380/2021 | North America/USA/California | 2021-05-18 | CDPH VBL | California Department of Public Health | Emily Smith on behalf of CDPH-COVIDNet and UCSD EXCITE lab |
| EPI_ISL_3244467 | hCoV-19/USA/CA-CDPH-2000048985/2021 | North America/USA/California | 2021-05-27 | CDPH VBL | California Department of Public Health | Emily Smith on behalf of CDPH-COVIDNet and UCSD EXCITE lab |
| EPI_ISL_475645 | hCoV-19/USA/CA-CSMC34/2020 | North America/USA/California | 2020-04-03 | Cedars-Sinai Medical Center, Department of Pathology & Laboratory Medicine, Molecular Pathology Laboratory | Cedars-Sinai Medical Center, Molecular Pathology Laboratory of Department of Pathology & Laboratory Medicine and Genomic Core | Wenjuan Zhang, John Paul Govindavari, Brian Davis, Stephanie Chen, Jong Taek Kim, Jianbo Song, Jean Lopategui, Jasmine T Plummer, Eric Vail |
| EPI_ISL_4256793 | hCoV-19/Ecuador/USFQ-2075-PUCE-82779/2021 | South America/Ecuador/Pichincha | 2021-09-06 | Center for Research on Health in Latin America | Institute of Microbiology, Universidad San Francisco de Quito | Erika B. MuÃ±oz, Fernanda Zurita, Mateo Carvajal, Monica Becerra-Wong, Juan JosÃ© Guadalupe, Sully MÃ¡rquez, BelÃ©n Prado-Vivar, Bernardo GutiÃ©rrez, Hugo Navarrete, Jaime Costales, VerÃ³nica BarragÃ¡n, Patricio Rojas-Silva, Gabriel Trueba, Michelle Grunauer, PaÃºl CÃ¡rdenas |
| EPI_ISL_913080 | hCoV-19/Austria/MUW_1323920/2020 | Europe/Austria/ | 2020-10-12 | Center for Virology | Center for Virology | Jeremy V. Camp, Irene Goerzer, Monika Redlberger-Fritz, Stephan W. Aberle |
| EPI_ISL_853900 | hCoV-19/Austria/CeMM1353/2020 | Europe/Austria/Vienna | 2020-08-10 | Center for Virology, Medical University of Vienna | Bergthaler laboratory, CeMM Research Center for Molecular Medicine of the Austrian Academy of Sciences | Lukas Endler, Alexandra Popa, Benedikt Agerer, Â Jakob-Wendelin Genger, Alexander Lercher, Anna Schedl, Thomas Penz, MichaelÂ Schuster, Jan Laine, MartinÂ Senekowitsch, ChristophÂ Bock, Andreas Bergthaler |
| EPI_ISL_815265 | hCoV-19/Germany/BW-Cento-30739418/2020 | Europe/Germany/Baden-Wurttemberg | 2020-07-04 | Centogene | Centogene | Peter Bauer, Krishna Kumar Kandaswamy, Vivi Hue-Trang Lieu |
| EPI_ISL_492988 | hCoV-19/Latvia/043/2020 | Europe/Latvia/ | 2020-06-30 | CentrÄlÄ laboratorija | Latvian Biomedical Research and Study Centre | Ivars SilamiÄ·elis, Kaspars Megnis, Monta Ustinova, Å…ikita Zrelovs, Vita RovÄ«te, Stella LapiÅ†a, Jana OsÄ«te, Marta PriedÄ«te, Uga Dumpis, JÄnis KloviÅ†Å¡ |
| EPI_ISL_2502545 | hCoV-19/Suriname/SR-100/2020 | South America/Suriname/ | 2020-12-16 | Central Laboratory, Bureau of Public Health (BOG) and Academic Hospital Paramaribo | Erasmus Medical Center | Bas B Oude Munnink, Cherise Beek, Consuella Partowidjojo, Dion Gajadin, Ed PF IJzerman, Emmanuelle Munger, Gary Gummels, Ingrid SK Krishnadath, Lycke Woittiez, Marion PG Koopmans, Mireille Van de Veer, Phyllis Pinas, Princes Wongsowidjojo, Radjesh Ori, Ranisha Doerbalie, Rohma Banwari, Soeradj Harkisoen, Stephen Vreden, Tilotmadebie Ramlal, Verne Nanhoe |
| EPI_ISL_2502548 | hCoV-19/Suriname/SR-105/2020 | South America/Suriname/ | 2020-12-19 | Central Laboratory, Bureau of Public Health (BOG) and Academic Hospital Paramaribo | Erasmus Medical Center | Bas B Oude Munnink, Cherise Beek, Consuella Partowidjojo, Dion Gajadin, Ed PF IJzerman, Emmanuelle Munger, Gary Gummels, Ingrid SK Krishnadath, Lycke Woittiez, Marion PG Koopmans, Mireille Van de Veer, Phyllis Pinas, Princes Wongsowidjojo, Radjesh Ori, Ranisha Doerbalie, Rohma Banwari, Soeradj Harkisoen, Stephen Vreden, Tilotmadebie Ramlal, Verne Nanhoe |
| EPI_ISL_2502448 | hCoV-19/Suriname/SR-154/2021 | South America/Suriname/ | 2021-03-30 | Central Laboratory, Bureau of Public Health (BOG) and Academic Hospital Paramaribo | Erasmus Medical Center | Bas B Oude Munnink, Cherise Beek, Consuella Partowidjojo, Dion Gajadin, Ed PF IJzerman, Emmanuelle Munger, Gary Gummels, Ingrid SK Krishnadath, Lycke Woittiez, Marion PG Koopmans, Mireille Van de Veer, Phyllis Pinas, Princes Wongsowidjojo, Radjesh Ori, Ranisha Doerbalie, Rohma Banwari, Soeradj Harkisoen, Stephen Vreden, Tilotmadebie Ramlal, Verne Nanhoe |
| EPI_ISL_3462737 | hCoV-19/Suriname/SR-318/2021 | South America/Suriname/ | 2021-05-24 | Central Laboratory, Bureau of Public Health (BOG) and Academic Hospital Paramaribo | Erasmus Medical Center | Bas B Oude Munnink, Cherise Beek, Consuella Partowidjojo, Dion Gajadin, Ed PF IJzerman, Emmanuelle Munger, Gary Gummels, Ingrid SK Krishnadath, Lycke Woittiez, Marion PG Koopmans, Mireille Van de Veer, Phyllis Pinas, Princes Wongsowidjojo, Radjesh Ori, Ranisha Doerbalie, Rohma Banwari, Soeradj Harkisoen, Stephen Vreden, Tilotmadebie Ramlal, Verne Nanhoe |
| EPI_ISL_4003124 | hCoV-19/Suriname/SR-541/2021 | South America/Suriname/ | 2021-08-05 | Central Laboratory, Bureau of Public Health (BOG) and Academic Hospital Paramaribo | Erasmus Medical Center | Bas B Oude Munnink, Cherise Beek, Consuella Partowidjojo, Dion Gajadin, Ed PF IJzerman, Emmanuelle Munger, Gary Gummels, Ingrid SK Krishnadath, Lycke Woittiez, Marion PG Koopmans, Mireille Van de Veer, Phyllis Pinas, Princes Wongsowidjojo, Radjesh Ori, Ranisha Doerbalie, Rohma Banwari, Soeradj Harkisoen, Stephen Vreden, Tilotmadebie Ramlal, Verne Nanhoe |
| EPI_ISL_4003128 | hCoV-19/Suriname/SR-545/2021 | South America/Suriname/ | 2021-08-05 | Central Laboratory, Bureau of Public Health (BOG) and Academic Hospital Paramaribo | Erasmus Medical Center | Bas B Oude Munnink, Cherise Beek, Consuella Partowidjojo, Dion Gajadin, Ed PF IJzerman, Emmanuelle Munger, Gary Gummels, Ingrid SK Krishnadath, Lycke Woittiez, Marion PG Koopmans, Mireille Van de Veer, Phyllis Pinas, Princes Wongsowidjojo, Radjesh Ori, Ranisha Doerbalie, Rohma Banwari, Soeradj Harkisoen, Stephen Vreden, Tilotmadebie Ramlal, Verne Nanhoe |
| EPI_ISL_4003136 | hCoV-19/Suriname/SR-553/2021 | South America/Suriname/ | 2021-08-08 | Central Laboratory, Bureau of Public Health (BOG) and Academic Hospital Paramaribo | Erasmus Medical Center | Bas B Oude Munnink, Cherise Beek, Consuella Partowidjojo, Dion Gajadin, Ed PF IJzerman, Emmanuelle Munger, Gary Gummels, Ingrid SK Krishnadath, Lycke Woittiez, Marion PG Koopmans, Mireille Van de Veer, Phyllis Pinas, Princes Wongsowidjojo, Radjesh Ori, Ranisha Doerbalie, Rohma Banwari, Soeradj Harkisoen, Stephen Vreden, Tilotmadebie Ramlal, Verne Nanhoe |
| EPI_ISL_4003138 | hCoV-19/Suriname/SR-555/2021 | South America/Suriname/ | 2021-08-09 | Central Laboratory, Bureau of Public Health (BOG) and Academic Hospital Paramaribo | Erasmus Medical Center | Bas B Oude Munnink, Cherise Beek, Consuella Partowidjojo, Dion Gajadin, Ed PF IJzerman, Emmanuelle Munger, Gary Gummels, Ingrid SK Krishnadath, Lycke Woittiez, Marion PG Koopmans, Mireille Van de Veer, Phyllis Pinas, Princes Wongsowidjojo, Radjesh Ori, Ranisha Doerbalie, Rohma Banwari, Soeradj Harkisoen, Stephen Vreden, Tilotmadebie Ramlal, Verne Nanhoe |
| EPI_ISL_1822739 | hCoV-19/Belize/BZ-CML-TCMC-BZ001-0820/2020 | North America/Belize/Belize | 2020-08-12 | Central Medical Laboratory | Texas Children's Microbiome Center | Ruth Ann Luna, Jennifer K. Spinler, Miguel Saldana, Sarah M. Gunter, Shannon E. Ronca, Adrianna Maliga, Kristy O. Murray, Gerhaldine Morazan, Melissa Diaz-Musa, Francis Morey, Russell I Manzanero |
| EPI_ISL_1822760 | hCoV-19/Belize/BZ-CML-TCMC-BZ002-0820/2020 | North America/Belize/Belize | 2020-08-12 | Central Medical Laboratory | Texas Children's Microbiome Center | Ruth Ann Luna, Jennifer K. Spinler, Miguel Saldana, Sarah M. Gunter, Shannon E. Ronca, Adrianna Maliga, Kristy O. Murray, Gerhaldine Morazan, Melissa Diaz-Musa, Francis Morey, Russell I Manzanero |
| EPI_ISL_1822764 | hCoV-19/Belize/BZ-CML-TCMC-BZ007-0920/2020 | North America/Belize/Belize | 2020-09-23 | Central Medical Laboratory | Texas Children's Microbiome Center | Ruth Ann Luna, Jennifer K. Spinler, Miguel Saldana, Sarah M. Gunter, Shannon E. Ronca, Adrianna Maliga, Kristy O. Murray, Gerhaldine Morazan, Melissa Diaz-Musa, Francis Morey, Russell I Manzanero |
| EPI_ISL_1822743 | hCoV-19/Belize/BZ-CML-TCMC-BZ026-1220/2020 | North America/Belize/Belize | 2020-12-22 | Central Medical Laboratory | Texas Children's Microbiome Center | Ruth Ann Luna, Jennifer K. Spinler, Miguel Saldana, Sarah M. Gunter, Shannon E. Ronca, Adrianna Maliga, Kristy O. Murray, Gerhaldine Morazan, Melissa Diaz-Musa, Francis Morey, Russell I Manzanero |
| EPI_ISL_1822779 | hCoV-19/Belize/BZ-CML-TCMC-BZ030-1220/2020 | North America/Belize/Belize | 2020-12-26 | Central Medical Laboratory | Texas Children's Microbiome Center | Ruth Ann Luna, Jennifer K. Spinler, Miguel Saldana, Sarah M. Gunter, Shannon E. Ronca, Adrianna Maliga, Kristy O. Murray, Gerhaldine Morazan, Melissa Diaz-Musa, Francis Morey, Russell I Manzanero |
| EPI_ISL_1822754 | hCoV-19/Belize/BZ-CML-TCMC-BZ040-0121/2021 | North America/Belize/Belize | 2021-01-04 | Central Medical Laboratory | Texas Children's Microbiome Center | Ruth Ann Luna, Jennifer K. Spinler, Miguel Saldana, Sarah M. Gunter, Shannon E. Ronca, Adrianna Maliga, Kristy O. Murray, Gerhaldine Morazan, Melissa Diaz-Musa, Francis Morey, Russell I Manzanero |
| EPI_ISL_4296404 | hCoV-19/Belize/CML-100/2021 | North America/Belize/Belize | 2021-06-29 | Central Medical Laboratory | Baylor College of Medicine | Alexander Kneubehl, Sarah Gunter, Shannon Ronca, Allison Lino, Adrianna Maliga, Sarah Strobel, Russell Manzanero, Gerhaldine Morazan, Kristy Murray |
| EPI_ISL_4296406 | hCoV-19/Belize/CML-102/2021 | North America/Belize/Belize | 2021-01-07 | Central Medical Laboratory | Baylor College of Medicine | Alexander Kneubehl, Sarah Gunter, Shannon Ronca, Allison Lino, Adrianna Maliga, Sarah Strobel, Russell Manzanero, Gerhaldine Morazan, Kristy Murray |
| EPI_ISL_4296408 | hCoV-19/Belize/CML-104/2021 | North America/Belize/Cayo | 2021-06-30 | Central Medical Laboratory | Baylor College of Medicine | Alexander Kneubehl, Sarah Gunter, Shannon Ronca, Allison Lino, Adrianna Maliga, Sarah Strobel, Russell Manzanero, Gerhaldine Morazan, Kristy Murray |
| EPI_ISL_4296411 | hCoV-19/Belize/CML-107/2021 | North America/Belize/Corozal | 2021-06-30 | Central Medical Laboratory | Baylor College of Medicine | Alexander Kneubehl, Sarah Gunter, Shannon Ronca, Allison Lino, Adrianna Maliga, Sarah Strobel, Russell Manzanero, Gerhaldine Morazan, Kristy Murray |
| EPI_ISL_4296320 | hCoV-19/Belize/CML-11/2021 | North America/Belize/Toledo | 2021-01-18 | Central Medical Laboratory | Baylor College of Medicine | Alexander Kneubehl, Sarah Gunter, Shannon Ronca, Allison Lino, Adrianna Maliga, Sarah Strobel, Russell Manzanero, Gerhaldine Morazan, Kristy Murray |
| EPI_ISL_4296414 | hCoV-19/Belize/CML-110/2021 | North America/Belize/Belize | 2021-06-29 | Central Medical Laboratory | Baylor College of Medicine | Alexander Kneubehl, Sarah Gunter, Shannon Ronca, Allison Lino, Adrianna Maliga, Sarah Strobel, Russell Manzanero, Gerhaldine Morazan, Kristy Murray |
| EPI_ISL_4296418 | hCoV-19/Belize/CML-114/2021 | North America/Belize/Toledo | 2021-06-27 | Central Medical Laboratory | Baylor College of Medicine | Alexander Kneubehl, Sarah Gunter, Shannon Ronca, Allison Lino, Adrianna Maliga, Sarah Strobel, Russell Manzanero, Gerhaldine Morazan, Kristy Murray |
| EPI_ISL_4296419 | hCoV-19/Belize/CML-115/2021 | North America/Belize/Toledo | 2021-06-27 | Central Medical Laboratory | Baylor College of Medicine | Alexander Kneubehl, Sarah Gunter, Shannon Ronca, Allison Lino, Adrianna Maliga, Sarah Strobel, Russell Manzanero, Gerhaldine Morazan, Kristy Murray |
| EPI_ISL_4296421 | hCoV-19/Belize/CML-117/2021 | North America/Belize/Belize | 2021-06-28 | Central Medical Laboratory | Baylor College of Medicine | Alexander Kneubehl, Sarah Gunter, Shannon Ronca, Allison Lino, Adrianna Maliga, Sarah Strobel, Russell Manzanero, Gerhaldine Morazan, Kristy Murray |
| EPI_ISL_4296429 | hCoV-19/Belize/CML-125/2021 | North America/Belize/Stann Creek | 2021-06-21 | Central Medical Laboratory | Baylor College of Medicine | Alexander Kneubehl, Sarah Gunter, Shannon Ronca, Allison Lino, Adrianna Maliga, Sarah Strobel, Russell Manzanero, Gerhaldine Morazan, Kristy Murray |
| EPI_ISL_4296430 | hCoV-19/Belize/CML-126/2021 | North America/Belize/Cayo | 2021-06-21 | Central Medical Laboratory | Baylor College of Medicine | Alexander Kneubehl, Sarah Gunter, Shannon Ronca, Allison Lino, Adrianna Maliga, Sarah Strobel, Russell Manzanero, Gerhaldine Morazan, Kristy Murray |
| EPI_ISL_4296321 | hCoV-19/Belize/CML-13/2021 | North America/Belize/Toledo | 2021-01-18 | Central Medical Laboratory | Baylor College of Medicine | Alexander Kneubehl, Sarah Gunter, Shannon Ronca, Allison Lino, Adrianna Maliga, Sarah Strobel, Russell Manzanero, Gerhaldine Morazan, Kristy Murray |
| EPI_ISL_4296342 | hCoV-19/Belize/CML-34/2021 | North America/Belize/Cayo | 2021-01-29 | Central Medical Laboratory | Baylor College of Medicine | Alexander Kneubehl, Sarah Gunter, Shannon Ronca, Allison Lino, Adrianna Maliga, Sarah Strobel, Russell Manzanero, Gerhaldine Morazan, Kristy Murray |
| EPI_ISL_4296343 | hCoV-19/Belize/CML-35/2021 | North America/Belize/Corozal | 2021-02-01 | Central Medical Laboratory | Baylor College of Medicine | Alexander Kneubehl, Sarah Gunter, Shannon Ronca, Allison Lino, Adrianna Maliga, Sarah Strobel, Russell Manzanero, Gerhaldine Morazan, Kristy Murray |
| EPI_ISL_4296345 | hCoV-19/Belize/CML-37/2021 | North America/Belize/Stann Creek | 2021-02-04 | Central Medical Laboratory | Baylor College of Medicine | Alexander Kneubehl, Sarah Gunter, Shannon Ronca, Allison Lino, Adrianna Maliga, Sarah Strobel, Russell Manzanero, Gerhaldine Morazan, Kristy Murray |
| EPI_ISL_4296349 | hCoV-19/Belize/CML-41/2021 | North America/Belize/Cayo | 2021-02-05 | Central Medical Laboratory | Baylor College of Medicine | Alexander Kneubehl, Sarah Gunter, Shannon Ronca, Allison Lino, Adrianna Maliga, Sarah Strobel, Russell Manzanero, Gerhaldine Morazan, Kristy Murray |
| EPI_ISL_4296357 | hCoV-19/Belize/CML-49/2021 | North America/Belize/Cayo | 2021-02-17 | Central Medical Laboratory | Baylor College of Medicine | Alexander Kneubehl, Sarah Gunter, Shannon Ronca, Allison Lino, Adrianna Maliga, Sarah Strobel, Russell Manzanero, Gerhaldine Morazan, Kristy Murray |
| EPI_ISL_4296314 | hCoV-19/Belize/CML-5/2021 | North America/Belize/Toledo | 2021-01-18 | Central Medical Laboratory | Baylor College of Medicine | Alexander Kneubehl, Sarah Gunter, Shannon Ronca, Allison Lino, Adrianna Maliga, Sarah Strobel, Russell Manzanero, Gerhaldine Morazan, Kristy Murray |
| EPI_ISL_4296363 | hCoV-19/Belize/CML-55/2021 | North America/Belize/Belize | 2021-03-09 | Central Medical Laboratory | Baylor College of Medicine | Alexander Kneubehl, Sarah Gunter, Shannon Ronca, Allison Lino, Adrianna Maliga, Sarah Strobel, Russell Manzanero, Gerhaldine Morazan, Kristy Murray |
| EPI_ISL_4296364 | hCoV-19/Belize/CML-56/2021 | North America/Belize/Cayo | 2021-03-15 | Central Medical Laboratory | Baylor College of Medicine | Alexander Kneubehl, Sarah Gunter, Shannon Ronca, Allison Lino, Adrianna Maliga, Sarah Strobel, Russell Manzanero, Gerhaldine Morazan, Kristy Murray |
| EPI_ISL_4296367 | hCoV-19/Belize/CML-62/2021 | North America/Belize/Stann Creek | 2021-05-11 | Central Medical Laboratory | Baylor College of Medicine | Alexander Kneubehl, Sarah Gunter, Shannon Ronca, Allison Lino, Adrianna Maliga, Sarah Strobel, Russell Manzanero, Gerhaldine Morazan, Kristy Murray |
| EPI_ISL_4296368 | hCoV-19/Belize/CML-63/2021 | North America/Belize/Orange Walk | 2021-05-25 | Central Medical Laboratory | Baylor College of Medicine | Alexander Kneubehl, Sarah Gunter, Shannon Ronca, Allison Lino, Adrianna Maliga, Sarah Strobel, Russell Manzanero, Gerhaldine Morazan, Kristy Murray |
| EPI_ISL_4296369 | hCoV-19/Belize/CML-64/2021 | North America/Belize/Orange Walk | 2021-05-25 | Central Medical Laboratory | Baylor College of Medicine | Alexander Kneubehl, Sarah Gunter, Shannon Ronca, Allison Lino, Adrianna Maliga, Sarah Strobel, Russell Manzanero, Gerhaldine Morazan, Kristy Murray |
| EPI_ISL_4296370 | hCoV-19/Belize/CML-66/2021 | North America/Belize/Cayo | 2021-05-30 | Central Medical Laboratory | Baylor College of Medicine | Alexander Kneubehl, Sarah Gunter, Shannon Ronca, Allison Lino, Adrianna Maliga, Sarah Strobel, Russell Manzanero, Gerhaldine Morazan, Kristy Murray |
| EPI_ISL_4296372 | hCoV-19/Belize/CML-68/2021 | North America/Belize/Orange Walk | 2021-06-18 | Central Medical Laboratory | Baylor College of Medicine | Alexander Kneubehl, Sarah Gunter, Shannon Ronca, Allison Lino, Adrianna Maliga, Sarah Strobel, Russell Manzanero, Gerhaldine Morazan, Kristy Murray |
| EPI_ISL_4296316 | hCoV-19/Belize/CML-7/2021 | North America/Belize/Corozal | 2021-01-19 | Central Medical Laboratory | Baylor College of Medicine | Alexander Kneubehl, Sarah Gunter, Shannon Ronca, Allison Lino, Adrianna Maliga, Sarah Strobel, Russell Manzanero, Gerhaldine Morazan, Kristy Murray |
| EPI_ISL_4296375 | hCoV-19/Belize/CML-71/2021 | North America/Belize/Orange Walk | 2021-06-19 | Central Medical Laboratory | Baylor College of Medicine | Alexander Kneubehl, Sarah Gunter, Shannon Ronca, Allison Lino, Adrianna Maliga, Sarah Strobel, Russell Manzanero, Gerhaldine Morazan, Kristy Murray |
| EPI_ISL_4296376 | hCoV-19/Belize/CML-72/2021 | North America/Belize/Belize | 2021-06-23 | Central Medical Laboratory | Baylor College of Medicine | Alexander Kneubehl, Sarah Gunter, Shannon Ronca, Allison Lino, Adrianna Maliga, Sarah Strobel, Russell Manzanero, Gerhaldine Morazan, Kristy Murray |
| EPI_ISL_4296377 | hCoV-19/Belize/CML-73/2021 | North America/Belize/Orange Walk | 2021-06-23 | Central Medical Laboratory | Baylor College of Medicine | Alexander Kneubehl, Sarah Gunter, Shannon Ronca, Allison Lino, Adrianna Maliga, Sarah Strobel, Russell Manzanero, Gerhaldine Morazan, Kristy Murray |
| EPI_ISL_4296379 | hCoV-19/Belize/CML-75/2021 | North America/Belize/Belize | 2021-06-23 | Central Medical Laboratory | Baylor College of Medicine | Alexander Kneubehl, Sarah Gunter, Shannon Ronca, Allison Lino, Adrianna Maliga, Sarah Strobel, Russell Manzanero, Gerhaldine Morazan, Kristy Murray |
| EPI_ISL_4296380 | hCoV-19/Belize/CML-76/2021 | North America/Belize/Stann Creek | 2021-06-23 | Central Medical Laboratory | Baylor College of Medicine | Alexander Kneubehl, Sarah Gunter, Shannon Ronca, Allison Lino, Adrianna Maliga, Sarah Strobel, Russell Manzanero, Gerhaldine Morazan, Kristy Murray |
| EPI_ISL_4296386 | hCoV-19/Belize/CML-82/2021 | North America/Belize/Corozal | 2021-06-23 | Central Medical Laboratory | Baylor College of Medicine | Alexander Kneubehl, Sarah Gunter, Shannon Ronca, Allison Lino, Adrianna Maliga, Sarah Strobel, Russell Manzanero, Gerhaldine Morazan, Kristy Murray |
| EPI_ISL_4296394 | hCoV-19/Belize/CML-90/2021 | North America/Belize/Belize | 2021-05-07 | Central Medical Laboratory | Baylor College of Medicine | Alexander Kneubehl, Sarah Gunter, Shannon Ronca, Allison Lino, Adrianna Maliga, Sarah Strobel, Russell Manzanero, Gerhaldine Morazan, Kristy Murray |
| EPI_ISL_4296396 | hCoV-19/Belize/CML-92/2021 | North America/Belize/Toledo | 2021-02-07 | Central Medical Laboratory | Baylor College of Medicine | Alexander Kneubehl, Sarah Gunter, Shannon Ronca, Allison Lino, Adrianna Maliga, Sarah Strobel, Russell Manzanero, Gerhaldine Morazan, Kristy Murray |
| EPI_ISL_2463942 | hCoV-19/Greece/KEDY_11782/2021 | Europe/Greece/Attica | 2021-05-21 | Central Public Health Lab, National Public Health Organization | Central Public Health Lab, National Public Health Organization | Kyriaki Tryfinopoulou, Gregory Spanakos et al |
| EPI_ISL_693471 | hCoV-19/PapuaNewGuinea/1/2020 | Oceania/Papua New Guinea/ | 2020-07-21 | Central Public Health Laboratory | National Public Health Laboratory, National Centre for Infectious Diseases | Tze Minn Mak, Sophie Octavia, Zhenyang Zhou, Esorom Daoni, Theresa Palou, Lin Cui, Raymond Tzer Pin Lin |
| EPI_ISL_978512 | hCoV-19/Brazil/BA-27/2020 | South America/Brazil/Bahia | 2020-02-28 | Central Public Health Laboratory - LACEN -Bahia, Salvador, Brazil | Central Public Health Laboratory - LACEN -Bahia, Salvador, Brazil | Stephane Tosta, Luciana Oliveira, Vanessa Nardy,PatrÃ­cia Cajado,Marcela GÃ³mez, Breno Dominguez, Jaqueline Gomes, Vagner Fonseca,Marta Giovanetti,Luiz Alcantara, Felicidade Pereira, Arabela Leal |
| EPI_ISL_1583668 | hCoV-19/Brazil/BA-LACEN-140/2020 | South America/Brazil/Bahia | 2020-06-15 | Central Public Health Laboratory - LACEN -Bahia, Salvador, Brazil | Central Public Health Laboratory - LACEN -Bahia, Salvador, Brazil | Stephane Tosta, Luciana Oliveira, Vanessa Nardy,PatrÃ­cia Cajado,Marcela GÃ³mez, Breno Dominguez, Jaqueline Gomes, Vagner Fonseca,Marta Giovanetti,Luiz Alcantara, Felicidade Pereira, Arabela Leal |
| EPI_ISL_4417769 | hCoV-19/Brazil/BA-LACEN-BA588-291653340/2021 | South America/Brazil/Bahia | 2021-09-02 | Central Public Health Laboratory - LACEN -Bahia, Salvador, Brazil | Central Public Health Laboratory - LACEN -Bahia, Salvador, Brazil | Stephane Tosta, Luciana Oliveira, Vanessa Nardy, Gabriela Menezes,Marcela GÃ³mez, Lenisa Dandara, Jaqueline Gomes, Vagner Fonseca,Marta Giovanetti,Luiz Alcantara, Felicidade Pereira, Arabela Leal |
| EPI_ISL_3045198 | hCoV-19/Greece/16386/2021 | Europe/Greece/ | 2021-06-12 | Central Public Health Laboratory, National Public Health Organization | Central Public Health Laboratory, National Public Health Organization | Kyriaki Tryfinopoulou, G.Spanakos, A.Katsoulidou et al |
| EPI_ISL_1717098 | hCoV-19/SriLanka/NR5/2021 | Asia/Sri Lanka/Quarantine | 2021-01-22 | Centre for Dengue Research and AICBU, Department of Immunology and Molecular Medicine | Centre for Dengue Research and AICBU, Department of Immunology and Molecular Medicine | Chandima Jeewandara, Deshni Jayathilaka, Dinuka Ariyaratne, Tibutius Thanesh Pramanayagam, Diyanath Ranasinghe, Laksiri Gomes, Gathsaurie Neelika Malavige |
| EPI_ISL_413550 | hCoV-19/Nigeria/Lagos01/2020 | Africa/Nigeria/Lagos | 2020-02-27 | Centre for Human and Zoonotic Virology (CHAZVY), College of Medicine University of Lagos/Lagos University Teaching Hospital (LUTH), part of the Laboratory Network of the Nigeria Centre for Disease Control (NCDC) | African Centre of Excellence for Genomics of Infectious Diseases (ACEGID), Redeemer's University, Ede, Osun State, Nigeria | Oluniyi P.E., Ajogbasile F.V., Kayode A., Oguzie J., Folarin O.A., Ihekweazu C. Happi C.T. |
| EPI_ISL_1760556 | hCoV-19/Gabon/JE08/2021 | Africa/Gabon/Libreville | 2021-01-12 | Centre Hospitalier Universitaire MÃ¨re-Enfant, Fondation Jeanne Ebori (CHUMEFJE) | Centre de recherches mÃ©dicales de LambarÃ©nÃ© (CERMEL) | Haruka Abe, Yuri Ushijima, Rodrigue Bikangui, Samira Zoa-Assoumou, Georgelin Nguema Ondo, GÃ©dÃ©on P. Manouana, Ayong Moure, Emilio Skarwan, BÃ©nÃ©dicte Ndeboko, Rotimi Myrabelle Avome Houechenou, Joel Fleury Djoba Siawaya, Bertrand Lell, Ayola A. Adegnika, Jiro Yasuda |
| EPI_ISL_629018 | hCoV-19/Chile/RM-CMM-0069M/2020 | South America/Chile/Region Metropolitana de Santiago | 2020-05-01 | Centro de BiotecnologÃ­a Vegetal, Universidad AndrÃ©s Bello, Center for Genome Regulation | Center for Mathematical Modeling and Center for Genome Regulation. Santiago, Chile | Bastias M, Sanhueza D, Travisany D, Allende ML, Maass A, GonzÃ¡lez M, Bustos F, Arriagada G, Montecino, M, Orellana A, Castro E, Meneses C. |
| EPI_ISL_635485 | hCoV-19/Mexico/BCN-ALSR-2457/2020 | North America/Mexico/Baja California | 2020-05-16 | Centro de Diagnâˆšâ‰¥stico COVID-19 UABC Tijuana | Andersen lab at Scripps Research | SEARCH Alliance San Diego with Idanya RubÃ­ SerafÃ­n Higuera, Manuel SÃ¡nchez Alavez, Jorge Luis JimÃ©nez Niebla, GermÃ¡n Ibarra, Jonathan Vincent Baena, Oscar EfrÃ©n Zazueta Fierro |
| EPI_ISL_635517 | hCoV-19/Mexico/BCN-ALSR-2512/2020 | North America/Mexico/Baja California | 2020-06-12 | Centro de Diagnâˆšâ‰¥stico COVID-19 UABC Tijuana | Andersen lab at Scripps Research | SEARCH Alliance San Diego with Idanya RubÃ­ SerafÃ­n Higuera, Manuel SÃ¡nchez Alavez, Jorge Luis JimÃ©nez Niebla, GermÃ¡n Ibarra, Jonathan Vincent Baena, Oscar EfrÃ©n Zazueta Fierro |
| EPI_ISL_635559 | hCoV-19/Mexico/BCN-ALSR-2556/2020 | North America/Mexico/Baja California | 2020-07-04 | Centro de Diagnâˆšâ‰¥stico COVID-19 UABC Tijuana | Andersen lab at Scripps Research | SEARCH Alliance San Diego with Idanya RubÃ­ SerafÃ­n Higuera, Manuel SÃ¡nchez Alavez, Jorge Luis JimÃ©nez Niebla, GermÃ¡n Ibarra, Jonathan Vincent Baena, Oscar EfrÃ©n Zazueta Fierro |
| EPI_ISL_1531872 | hCoV-19/Mexico/BCN-ALSR-8408/2020 | North America/Mexico/Baja California | 2020-08-26 | Centro de Diagnostico COVID-19 UABC Tijuana | Andersen lab at Scripps Research | SEARCH Alliance San Diego with Idanya Rubi Serafin Higuera, Manuel Sanchez Alavez, Jorge Luis Jimenez Niebla, German Ibarra, Jonathan Vincent Baena, Oscar Efren Zazueta Fierro |
| EPI_ISL_1531890 | hCoV-19/Mexico/BCN-ALSR-8426/2020 | North America/Mexico/Baja California | 2020-09-04 | Centro de Diagnostico COVID-19 UABC Tijuana | Andersen lab at Scripps Research | SEARCH Alliance San Diego with Idanya Rubi Serafin Higuera, Manuel Sanchez Alavez, Jorge Luis Jimenez Niebla, German Ibarra, Jonathan Vincent Baena, Oscar Efren Zazueta Fierro |
| EPI_ISL_2339903 | hCoV-19/Colombia/ANT-C73/2020 | South America/Colombia/Antioquia | 2020-09-09 | Centro de Estudio de Enfermedades Autoinmunes (CREA), Universidad del Rosario, Bogota, Colombia | Centro de Investigaciones en MicrobiologÃ­a y BiotecnologÃ­a-UR (CIMBIUR), Facultad de Ciencias Naturales, Universidad del Rosario, BogotÃ¡, Colombia | Juan-Manuel Anaya, Gustavo Salguero, Juan Esteban Gallo, Carolina RamÃ­rez-Santana, Sergio CastaÃ±eda, Nathalia Ballesteros, Marina MuÃ±oz, Luz H. PatiÃ±o, Juan David RamÃ­rez |
| EPI_ISL_2339897 | hCoV-19/Colombia/DC-C47/2020 | South America/Colombia/Bogota | 2020-07-07 | Centro de Estudio de Enfermedades Autoinmunes (CREA), Universidad del Rosario, Bogota, Colombia | Centro de Investigaciones en MicrobiologÃ­a y BiotecnologÃ­a-UR (CIMBIUR), Facultad de Ciencias Naturales, Universidad del Rosario, BogotÃ¡, Colombia | Juan-Manuel Anaya, Gustavo Salguero, Juan Esteban Gallo, Carolina RamÃ­rez-Santana, Sergio CastaÃ±eda, Nathalia Ballesteros, Marina MuÃ±oz, Luz H. PatiÃ±o, Juan David RamÃ­rez |
| EPI_ISL_2612378 | hCoV-19/Brazil/AC-MB-09646/2021 | South America/Brazil/Acre | 2021-03-19 | Centro de Infectologia Charles MÃ©rieux/ LaboratÃ³rio Rodolphe MÃ©rieux, FUNDHACRE | Bioinformatics Laboratory / LNCC | Alessandra P Lamarca, Luiz G P de Almeida, Ronaldo da Silva F Jr,Douglas Terra Machado, Alexandra L Gerber, Ana Paula de C GuimarÃ£es, Cirley Maria de Oliveira Lobato, Andreas Stocker, Luiz Fellype Alves de Souza, Ana Tereza R Vasconcelos |
| EPI_ISL_732772 | hCoV-19/Spain/RI-IBV-99010934/2020 | Europe/Spain/La_Rioja | 2020-11-11 | Centro de InvestigaciÃ³n BiomÃ©dica de La Rioja - Hospital San Pedro LogroÃ±o | SeqCOVID-SPAIN consortium/IBV(CSIC) | MarÃ­a de Toro, JosÃ© Manuel Azcona GutiÃ©rrez, MarÃ­a Pilar Bea Escudero, Miriam Blasco Alberdi and SeqCOVID-SPAIN consortium |
| EPI_ISL_1585522 | hCoV-19/Mexico/COL_INER_IMSS_00519/2021 | North America/Mexico/Colima | 2021-03-15 | Centro de InvestigaciÃ³n BiomÃ©dica de Occidente (CIBO) | Instituto Nacional de Enfermedades Respiratorias (INER): Centro de InvestigaciÃ³n en Enfermedades Infecciosas (CIENI) | Consorcio Mexicano de Vigilancia GenÃ³mica (CoViGen-Mex). Authors (in alphabetical order): Julio Elias Alvarado-Yaah, Carlos F. Arias, Santiago Ãvila-RÃ­os, VÃ­ctor Hugo Borja-Aburto, Celia Boukadida, Juan Bautista Chale-Dzul, CÃ©lida Duque Molina, JosÃ© Antonio Enciso-Moreno, Gloria Elena Espinosa-Ayala, Fernando Fontove-Herrera, Victor Eduardo Garcia-Arias, ConcepciÃ³n Grajales-MuÃ±iz, Ricardo Grande, Alfredo Herrera-Estrella, Carla IvÃ³n Herrera-Najera, Pavel Isa, Brenda Irasema Maldonado-Meza, Bernardo MartÃ­nez-Miguel, Margarita MatÃ­as-Florentino, MarÃ­a Guadalupe de JesÃºs Mireles-Rivera, Gloria MarÃ­a Molina-Salinas, Hector Montoya-Fuentes, JosÃ© Esteban MuÃ±oz-Medina, JosÃ© de JesÃºs NuÃ±ez-Contreras, Alicia OcaÃ±a-MondragÃ³n, Luis Alberto Ochoa-Carrera, Hector Esteban Paz-JuÃ¡rez, Francisco Pulido, Helen Haydee Fernanda RamÃ­rez-Plascencia, Angel Gustavo Salas-Lais, Alejandro Sanchez-Flores, Clara Esperanza Santacruz-Tinoco, MarÃ­a Guadalupe Santiago-Mauricio, Nelly SÃ©lem-Mojica, Blanca Taboada, Gloria Vazquez |
| EPI_ISL_4006594 | hCoV-19/Mexico/GUA_IBT_IMSS_2695/2021 | North America/Mexico/Guanajuato | 2021-08-13 | Centro de InvestigaciÃ³n BiomÃ©dica de Occidente (CIBO) | Instituto de BiotecnologÃ­a de la UNAM | Consorcio Mexicano de Vigilancia GenÃ³mica (CoViGen-Mex). Authors (in alphabetical order): Julio Elias Alvarado-Yaah, Carlos F. Arias, Santiago Ãvila-RÃ­os, Eduardo Becerril-Vargas, VÃ­ctor Hugo Borja-Aburto, Celia Boukadida, CristÃ³bal ChÃ¡idez-QuirÃ³z, Juan Bautista Chale-Dzul, Ricardo Ciria Merce, Andreu Comas-GarcÃ­a, CÃ©lida Duque Molina, Julissa Enciso-Ibarra, JosÃ© Antonio Enciso-Moreno, Gloria Elena Espinosa-Ayala, Fernando Fontove-Herrera, Daniel Fregoso-Rueda, Victor Eduardo GarcÃ­a-Arias, Alejandra GarcÃ­a-Gasca, Bruno GÃ³mez-Gil, Jean Pierre GonzÃ¡lez, Irvin GonzÃ¡lez-LÃ³pez, ConcepciÃ³n Grajales-MuÃ±iz, Ricardo Grande, Rosa MarÃ­a GutiÃ©rrez Rios, JesÃºs HernÃ¡ndez, Alejandra HernÃ¡ndez-TerÃ¡n, Alfredo Herrera-Estrella, Carla IvÃ³n Herrera-Najera, Pavel Isa, VerÃ³nica Mata-Haro, Daniel Lira Morales, Susana Lopez, Antonio Loza RomÃ¡n, Brenda Irasema Maldonado-Meza, Bernardo MartÃ­nez-Miguel, JosÃ© Arturo MartÃ­nez-Orozco, CÃ©lida MartÃ­nez- RodrÃ­guez, Margarita MatÃ­as-Florentino, Fidencio MejÃ­a-Nepomuceno, MarÃ­a Guadalupe de JesÃºs Mireles-Rivera, Gloria MarÃ­a Molina-Salinas, Hector Montoya-Fuentes, , Mario MÃºjica-SÃ¡nchez, JosÃ© Esteban MuÃ±oz-Medina, JosÃ© de JesÃºs NuÃ±ez-Contreras, Alicia OcaÃ±a-MondragÃ³n, Luis Alberto Ochoa-Carrera, Hector Esteban Paz-JuÃ¡rez, Marissa Perez-Garcia, Francisco Pulido, Helen Haydee Fernanda RamÃ­rez-Plascencia, Jorge Salas-HernÃ¡ndez, Angel Gustavo Salas-Lais, Alejandro SÃ¡nchez-Flores, Clara Esperanza Santacruz-Tinoco, MarÃ­a Guadalupe Santiago-Mauricio, Selene ZÃ¡rate, Nelly SÃ©lem-Mojica, Blanca Taboada, Kathia Elizabeth Tapia-Diaz, Gloria Vazquez, Joel Armando VÃ¡zquez-PÃ©rez). |
| EPI_ISL_3557188 | hCoV-19/Mexico/GUA_INER_IMSS_1917/2021 | North America/Mexico/Guanajuato | 2021-07-27 | Centro de InvestigaciÃ³n BiomÃ©dica de Occidente (CIBO) | Centro de InvestigaciÃ³n en Enfermedades Infecciosas (CIENI), Instituto Nacional de Enfermedades Respiratorias (INER) | Consorcio Mexicano de Vigilancia GenÃ³mica (CoViGen-Mex). Authors (in alphabetical order): Julio Elias Alvarado-Yaah, Carlos F. Arias, Santiago Ãvila-RÃ­os, Eduardo Becerril-Vargas, VÃ­ctor Hugo Borja-Aburto, Celia Boukadida, CristÃ³bal ChÃ¡idez-QuirÃ³z, Juan Bautista Chale-Dzul, Ricardo Ciria Merce, Andreu Comas-GarcÃ­a, CÃ©lida Duque Molina, Julissa Enciso-Ibarra, JosÃ© Antonio Enciso-Moreno, Gloria Elena Espinosa-Ayala, Fernando Fontove-Herrera, Daniel Fregoso-Rueda, Victor Eduardo GarcÃ­a-Arias, Alejandra GarcÃ­a-Gasca, Bruno GÃ³mez-Gil, Jean Pierre GonzÃ¡lez, Irvin GonzÃ¡lez-LÃ³pez, ConcepciÃ³n Grajales-MuÃ±iz, Ricardo Grande, Rosa MarÃ­a GutiÃ©rrez Rios, JesÃºs HernÃ¡ndez, Alejandra HernÃ¡ndez-TerÃ¡n, Alfredo Herrera-Estrella, Carla IvÃ³n Herrera-Najera, Pavel Isa, VerÃ³nica Mata-Haro, Daniel Lira Morales, Susana Lopez, Antonio Loza RomÃ¡n, Brenda Irasema Maldonado-Meza, Bernardo MartÃ­nez-Miguel, JosÃ© Arturo MartÃ­nez-Orozco, CÃ©lida MartÃ­nez- RodrÃ­guez, Margarita MatÃ­as-Florentino, Fidencio MejÃ­a-Nepomuceno, MarÃ­a Guadalupe de JesÃºs Mireles-Rivera, Gloria MarÃ­a Molina-Salinas, Hector Montoya-Fuentes, Mario MÃºjica-SÃ¡nchez, JosÃ© Esteban MuÃ±oz-Medina, JosÃ© de JesÃºs NuÃ±ez-Contreras, Alicia OcaÃ±a-MondragÃ³n, Luis Alberto Ochoa-Carrera, Hector Esteban Paz-JuÃ¡rez, Marissa Perez-Garcia, Francisco Pulido, Helen Haydee Fernanda RamÃ­rez-Plascencia, Jorge Salas-HernÃ¡ndez, Angel Gustavo Salas-Lais, Alejandro SÃ¡nchez-Flores, Clara Esperanza Santacruz-Tinoco, MarÃ­a Guadalupe Santiago-Mauricio, Selene ZÃ¡rate, Nelly SÃ©lem-Mojica, Blanca Taboada, Kathia Elizabeth Tapia-Diaz, Gloria Vazquez, Joel Armando VÃ¡zquez-PÃ©rez. |
| EPI_ISL_3347825 | hCoV-19/Mexico/JAL_IBT_IMSS_2202/2021 | North America/Mexico/Jalisco | 2021-07-15 | Centro de InvestigaciÃ³n BiomÃ©dica de Occidente (CIBO) | Instituto de BiotecnologÃ­a de la UNAM | Consorcio Mexicano de Vigilancia GenÃ³mica (CoViGen-Mex). Authors (in alphabetical order): Julio Elias Alvarado-Yaah, Carlos F. Arias, Santiago Ãvila-RÃ­os, Eduardo Becerril-Vargas, VÃ­ctor Hugo Borja-Aburto, Celia Boukadida, CristÃ³bal ChÃ¡idez-QuirÃ³z, Juan Bautista Chale-Dzul, Ricardo Ciria Merce, Andreu Comas-GarcÃ­a, CÃ©lida Duque Molina, Julissa Enciso-Ibarra, JosÃ© Antonio Enciso-Moreno, Gloria Elena Espinosa-Ayala, Fernando Fontove-Herrera, Daniel Fregoso-Rueda, Victor Eduardo GarcÃ­a-Arias, Alejandra GarcÃ­a-Gasca, Bruno GÃ³mez-Gil, Jean Pierre GonzÃ¡lez, Irvin GonzÃ¡lez-LÃ³pez, ConcepciÃ³n Grajales-MuÃ±iz, Ricardo Grande, Rosa MarÃ­a GutiÃ©rrez Rios, JesÃºs HernÃ¡ndez, Alejandra HernÃ¡ndez-TerÃ¡n, Alfredo Herrera-Estrella, Carla IvÃ³n Herrera-Najera, Pavel Isa, VerÃ³nica Mata-Haro, Daniel Lira Morales, Susana Lopez, Antonio Loza RomÃ¡n, Brenda Irasema Maldonado-Meza, Bernardo MartÃ­nez-Miguel, JosÃ© Arturo MartÃ­nez-Orozco, CÃ©lida MartÃ­nez- RodrÃ­guez, Margarita MatÃ­as-Florentino, Fidencio MejÃ­a-Nepomuceno, MarÃ­a Guadalupe de JesÃºs Mireles-Rivera, Gloria MarÃ­a Molina-Salinas, Hector Montoya-Fuentes, , Mario MÃºjica-SÃ¡nchez, JosÃ© Esteban MuÃ±oz-Medina, JosÃ© de JesÃºs NuÃ±ez-Contreras, Alicia OcaÃ±a-MondragÃ³n, Luis Alberto Ochoa-Carrera, Hector Esteban Paz-JuÃ¡rez, Marissa Perez-Garcia, Francisco Pulido, Helen Haydee Fernanda RamÃ­rez-Plascencia, Jorge Salas-HernÃ¡ndez, Angel Gustavo Salas-Lais, Alejandro SÃ¡nchez-Flores, Clara Esperanza Santacruz-Tinoco, MarÃ­a Guadalupe Santiago-Mauricio, Selene ZÃ¡rate, Nelly SÃ©lem-Mojica, Blanca Taboada, Kathia Elizabeth Tapia-Diaz, Gloria Vazquez, Joel Armando VÃ¡zquez-PÃ©rez). |
| EPI_ISL_2490498 | hCoV-19/Mexico/JAL_INER_IMSS_1181/2021 | North America/Mexico/Jalisco | 2021-05-12 | Centro de InvestigaciÃ³n BiomÃ©dica de Occidente (CIBO) | Centro de InvestigaciÃ³n en Enfermedades Infecciosas (CIENI), Instituto Nacional de Enfermedades Respiratorias (INER) | Consorcio Mexicano de Vigilancia GenÃ³mica (CoViGen-Mex). Authors (in alphabetical order): Julio Elias Alvarado-Yaah, Carlos F. Arias, Santiago Ãvila-RÃ­os, Eduardo Becerril-Vargas, VÃ­ctor Hugo Borja-Aburto, Celia Boukadida, CristÃ³bal ChÃ¡idez-QuirÃ³z, Juan Bautista Chale-Dzul, Ricardo Ciria Merce, Andreu Comas-GarcÃ­a, CÃ©lida Duque Molina, Julissa Enciso-Ibarra, JosÃ© Antonio Enciso-Moreno, Gloria Elena Espinosa-Ayala, Fernando Fontove-Herrera, Daniel Fregoso-Rueda, Victor Eduardo Garcia-Arias, Alejandra Garcia-Gasca, Bruno Gomez-Gil, Jean Pierre GonzÃ¡lez, Irvin GonzÃ¡lez-LÃ³pez, ConcepciÃ³n Grajales-MuÃ±iz, Ricardo Grande, Rosa MarÃ­a Gutierrez Rios, Alejandra HernÃ¡ndez-TerÃ¡n, Alfredo Herrera-Estrella, Carla IvÃ³n Herrera-Najera, Pavel Isa, Daniel Lira Morales, Susana Lopez, Antonio Loza RomÃ¡n, Brenda Irasema Maldonado-Meza, Bernardo MartÃ­nez-Miguel, JosÃ© Arturo MartÃ­nez-Orozco, CÃ©lida MartÃ­nez- RodrÃ­guez, Margarita MatÃ­as-Florentino, Fidencio MejÃ­a-Nepomuceno, MarÃ­a Guadalupe de JesÃºs Mireles-Rivera, Gloria MarÃ­a Molina-Salinas, Hector Montoya-Fuentes, , Mario MÃºjica-SÃ¡nchez, JosÃ© Esteban MuÃ±oz-Medina, JosÃ© de JesÃºs NuÃ±ez-Contreras, Alicia OcaÃ±a-MondragÃ³n, Luis Alberto Ochoa-Carrera, Hector Esteban Paz-JuÃ¡rez, Marissa Perez-Garcia, Francisco Pulido, Helen Haydee Fernanda RamÃ­rez-Plascencia, Jorge Salas-HernÃ¡ndez, Angel Gustavo Salas-Lais, Alejandro Sanchez-Flores, Clara Esperanza Santacruz-Tinoco, MarÃ­a Guadalupe Santiago-Mauricio, Selene ZÃ¡rate, Nelly SÃ©lem-Mojica, Blanca Taboada, Gloria Vazquez, Joel Armando VÃ¡zquez-PÃ©rez. |
| EPI_ISL_2402173 | hCoV-19/Mexico/JAL_LANGEBIO_IMSS_0762/2021 | North America/Mexico/Jalisco | 2021-04-26 | Centro de InvestigaciÃ³n BiomÃ©dica de Occidente (CIBO) | Unidad de Genomica Avanzada | Consorcio Mexicano de Vigilancia Genomica (CoViGen-Mex). Authors (in alphabetical order): Julio Elias Alvarado-Yaah, Carlos F. Arias, Santiago Avila-Rios, Victor Hugo Borja-Aburto, Celia Boukadida, Juan Bautista Chale-Dzul , Jose Antonio Enciso-Moreno, Gloria Elena Espinoza-Ayala, Fernando Fontove-Herrera, Concepcion Grajales-Muniz, Ricardo Grande, Alfredo Herrera-Estrella, Carla Ivon Herrera-Najera, Pavel Isa, Brenda Irasema Maldonado-Meza, Bernardo Martinez-Miguel, Margarita Matias-Florentino, Maria Guadalupe de Jesus Mireles-Rivera, Gloria Maria Molina-Salinas, Hector Montoya-Fuentes, Jose Esteban Munoz-Medina, Jose de Jesus Nunez-Contreras, Alicia Ocana-Mondragon, Luis Alberto Ochoa-Carrera, Hector Esteban Paz-Juarez, Francisco Pulido, Helen Haydee Fernanda Ramirez-Plascencia, Angel Gustavo Salas-Lais, Jorge Ivan Salinal-Nevarez, Alejandro Sanchez-Flores, Clara Esperanza Santacruz-Tinoco, Maria Guadalupe Santiago-Mauricio, Nelly Selem-Mojica, Blanca Taboada, Gloria Vazquez |
| EPI_ISL_2801742 | hCoV-19/Mexico/JAL-IBT_IMSS_1875/2021 | North America/Mexico/Jalisco | 2021-06-06 | Centro de InvestigaciÃ³n BiomÃ©dica de Occidente (CIBO) | Instituto de BiotecnologÃ­a de la UNAM | Consorcio Mexicano de Vigilancia GenÃ³mica (CoViGen-Mex). Authors (in alphabetical order): Julio Elias Alvarado-Yaah, Carlos F. Arias, Santiago Ãvila-RÃ­os, Eduardo Becerril-Vargas, VÃ­ctor Hugo Borja-Aburto, Celia Boukadida, CristÃ³bal ChÃ¡idez-QuirÃ³z, Juan Bautista Chale-Dzul, Ricardo Ciria Merce, Andreu Comas-GarcÃ­a, CÃ©lida Duque Molina, Julissa Enciso-Ibarra, JosÃ© Antonio Enciso-Moreno, Gloria Elena Espinosa-Ayala, Fernando Fontove-Herrera, Daniel Fregoso-Rueda, Victor Eduardo GarcÃ­a-Arias, Alejandra GarcÃ­a-Gasca, Bruno GÃ³mez-Gil, Jean Pierre GonzÃ¡lez, Irvin GonzÃ¡lez-LÃ³pez, ConcepciÃ³n Grajales-MuÃ±iz, Ricardo Grande, Rosa MarÃ­a GutiÃ©rrez Rios, JesÃºs HernÃ¡ndez, Alejandra HernÃ¡ndez-TerÃ¡n, Alfredo Herrera-Estrella, Carla IvÃ³n Herrera-Najera, Pavel Isa, VerÃ³nica Mata-Haro, Daniel Lira Morales, Susana Lopez, Antonio Loza RomÃ¡n, Brenda Irasema Maldonado-Meza, Bernardo MartÃ­nez-Miguel, JosÃ© Arturo MartÃ­nez-Orozco, CÃ©lida MartÃ­nez- RodrÃ­guez, Margarita MatÃ­as-Florentino, Fidencio MejÃ­a-Nepomuceno, MarÃ­a Guadalupe de JesÃºs Mireles-Rivera, Gloria MarÃ­a Molina-Salinas, Hector Montoya-Fuentes, , Mario MÃºjica-SÃ¡nchez, JosÃ© Esteban MuÃ±oz-Medina, JosÃ© de JesÃºs NuÃ±ez-Contreras, Alicia OcaÃ±a-MondragÃ³n, Luis Alberto Ochoa-Carrera, Hector Esteban Paz-JuÃ¡rez, Marissa Perez-Garcia, Francisco Pulido, Helen Haydee Fernanda RamÃ­rez-Plascencia, Jorge Salas-HernÃ¡ndez, Angel Gustavo Salas-Lais, Alejandro SÃ¡nchez-Flores, Clara Esperanza Santacruz-Tinoco, MarÃ­a Guadalupe Santiago-Mauricio, Selene ZÃ¡rate, Nelly SÃ©lem-Mojica, Blanca Taboada, Gloria Vazquez, Joel Armando VÃ¡zquez-PÃ©rez. |
| EPI_ISL_2091145 | hCoV-19/Mexico/MIC_INER_IMSS_00689/2021 | North America/Mexico/Michoacan | 2021-03-16 | Centro de InvestigaciÃ³n BiomÃ©dica de Occidente (CIBO) | Centro de InvestigaciÃ³n en Enfermedades Infecciosas (CIENI), Instituto Nacional de Enfermedades Respiratorias (INER) | Consorcio Mexicano de Vigilancia GenÃ³mica (CoViGen-Mex). Authors (in alphabetical order): Julio Elias Alvarado-Yaah, Carlos F. Arias, Santiago Ãvila-RÃ­os, VÃ­ctor Hugo Borja-Aburto, Celia Boukadida, Juan Bautista Chale-Dzul, CÃ©lida Duque Molina, JosÃ© Antonio Enciso-Moreno, Gloria Elena Espinosa-Ayala, Fernando Fontove-Herrera, Victor Eduardo Garcia-Arias, ConcepciÃ³n Grajales-MuÃ±iz, Ricardo Grande, Alfredo Herrera-Estrella, Carla IvÃ³n Herrera-Najera, Pavel Isa, Brenda Irasema Maldonado-Meza, Bernardo MartÃ­nez-Miguel, Margarita MatÃ­as-Florentino, MarÃ­a Guadalupe de JesÃºs Mireles-Rivera, Gloria MarÃ­a Molina-Salinas, Hector Montoya-Fuentes, JosÃ© Esteban MuÃ±oz-Medina, JosÃ© de JesÃºs NuÃ±ez-Contreras, Alicia OcaÃ±a-MondragÃ³n, Luis Alberto Ochoa-Carrera, Hector Esteban Paz-JuÃ¡rez, Francisco Pulido, Helen Haydee Fernanda RamÃ­rez-Plascencia, Angel Gustavo Salas-Lais, Alejandro Sanchez-Flores, Clara Esperanza Santacruz-Tinoco, MarÃ­a Guadalupe Santiago-Mauricio, Nelly SÃ©lem-Mojica, Blanca Taboada, Gloria Vazquez |
| EPI_ISL_3347830 | hCoV-19/Mexico/SON_IBT_IMSS_2211/2021 | North America/Mexico/Sonora | 2021-07-13 | Centro de InvestigaciÃ³n BiomÃ©dica de Occidente (CIBO) | Instituto de BiotecnologÃ­a de la UNAM | Consorcio Mexicano de Vigilancia GenÃ³mica (CoViGen-Mex). Authors (in alphabetical order): Julio Elias Alvarado-Yaah, Carlos F. Arias, Santiago Ãvila-RÃ­os, Eduardo Becerril-Vargas, VÃ­ctor Hugo Borja-Aburto, Celia Boukadida, CristÃ³bal ChÃ¡idez-QuirÃ³z, Juan Bautista Chale-Dzul, Ricardo Ciria Merce, Andreu Comas-GarcÃ­a, CÃ©lida Duque Molina, Julissa Enciso-Ibarra, JosÃ© Antonio Enciso-Moreno, Gloria Elena Espinosa-Ayala, Fernando Fontove-Herrera, Daniel Fregoso-Rueda, Victor Eduardo GarcÃ­a-Arias, Alejandra GarcÃ­a-Gasca, Bruno GÃ³mez-Gil, Jean Pierre GonzÃ¡lez, Irvin GonzÃ¡lez-LÃ³pez, ConcepciÃ³n Grajales-MuÃ±iz, Ricardo Grande, Rosa MarÃ­a GutiÃ©rrez Rios, JesÃºs HernÃ¡ndez, Alejandra HernÃ¡ndez-TerÃ¡n, Alfredo Herrera-Estrella, Carla IvÃ³n Herrera-Najera, Pavel Isa, VerÃ³nica Mata-Haro, Daniel Lira Morales, Susana Lopez, Antonio Loza RomÃ¡n, Brenda Irasema Maldonado-Meza, Bernardo MartÃ­nez-Miguel, JosÃ© Arturo MartÃ­nez-Orozco, CÃ©lida MartÃ­nez- RodrÃ­guez, Margarita MatÃ­as-Florentino, Fidencio MejÃ­a-Nepomuceno, MarÃ­a Guadalupe de JesÃºs Mireles-Rivera, Gloria MarÃ­a Molina-Salinas, Hector Montoya-Fuentes, , Mario MÃºjica-SÃ¡nchez, JosÃ© Esteban MuÃ±oz-Medina, JosÃ© de JesÃºs NuÃ±ez-Contreras, Alicia OcaÃ±a-MondragÃ³n, Luis Alberto Ochoa-Carrera, Hector Esteban Paz-JuÃ¡rez, Marissa Perez-Garcia, Francisco Pulido, Helen Haydee Fernanda RamÃ­rez-Plascencia, Jorge Salas-HernÃ¡ndez, Angel Gustavo Salas-Lais, Alejandro SÃ¡nchez-Flores, Clara Esperanza Santacruz-Tinoco, MarÃ­a Guadalupe Santiago-Mauricio, Selene ZÃ¡rate, Nelly SÃ©lem-Mojica, Blanca Taboada, Kathia Elizabeth Tapia-Diaz, Gloria Vazquez, Joel Armando VÃ¡zquez-PÃ©rez). |
| EPI_ISL_1585534 | hCoV-19/Mexico/SON_INER_IMSS_00531/2021 | North America/Mexico/Sonora | 2021-03-05 | Centro de InvestigaciÃ³n BiomÃ©dica de Occidente (CIBO) | Instituto Nacional de Enfermedades Respiratorias (INER): Centro de InvestigaciÃ³n en Enfermedades Infecciosas (CIENI) | Consorcio Mexicano de Vigilancia GenÃ³mica (CoViGen-Mex). Authors (in alphabetical order): Julio Elias Alvarado-Yaah, Carlos F. Arias, Santiago Ãvila-RÃ­os, VÃ­ctor Hugo Borja-Aburto, Celia Boukadida, Juan Bautista Chale-Dzul, CÃ©lida Duque Molina, JosÃ© Antonio Enciso-Moreno, Gloria Elena Espinosa-Ayala, Fernando Fontove-Herrera, Victor Eduardo Garcia-Arias, ConcepciÃ³n Grajales-MuÃ±iz, Ricardo Grande, Alfredo Herrera-Estrella, Carla IvÃ³n Herrera-Najera, Pavel Isa, Brenda Irasema Maldonado-Meza, Bernardo MartÃ­nez-Miguel, Margarita MatÃ­as-Florentino, MarÃ­a Guadalupe de JesÃºs Mireles-Rivera, Gloria MarÃ­a Molina-Salinas, Hector Montoya-Fuentes, JosÃ© Esteban MuÃ±oz-Medina, JosÃ© de JesÃºs NuÃ±ez-Contreras, Alicia OcaÃ±a-MondragÃ³n, Luis Alberto Ochoa-Carrera, Hector Esteban Paz-JuÃ¡rez, Francisco Pulido, Helen Haydee Fernanda RamÃ­rez-Plascencia, Angel Gustavo Salas-Lais, Alejandro Sanchez-Flores, Clara Esperanza Santacruz-Tinoco, MarÃ­a Guadalupe Santiago-Mauricio, Nelly SÃ©lem-Mojica, Blanca Taboada, Gloria Vazquez |
| EPI_ISL_2681248 | hCoV-19/Mexico/CHH_IBT_IMSS_1442/2021 | North America/Mexico/Chihuahua | 2021-05-31 | Centro de InvestigaciÃ³n BiomÃ©dica del Noreste (CIBIN) | Instituto de BiotecnologÃ­a de la UNAM | Consorcio Mexicano de Vigilancia GenÃ³mica (CoViGen-Mex). Authors (in alphabetical order): Julio Elias Alvarado-Yaah, Carlos F. Arias, Santiago Ãvila-RÃ­os, Eduardo Becerril-Vargas, VÃ­ctor Hugo Borja-Aburto, Celia Boukadida, CristÃ³bal ChÃ¡idez-QuirÃ³z, Juan Bautista Chale-Dzul, Ricardo Ciria Merce, Andreu Comas-GarcÃ­a, CÃ©lida Duque Molina, Julissa Enciso-Ibarra, JosÃ© Antonio Enciso-Moreno, Gloria Elena Espinosa-Ayala, Fernando Fontove-Herrera, Daniel Fregoso-Rueda, Victor Eduardo GarcÃ­a-Arias, Alejandra GarcÃ­a-Gasca, Bruno GÃ³mez-Gil, Jean Pierre GonzÃ¡lez, Irvin GonzÃ¡lez-LÃ³pez, ConcepciÃ³n Grajales-MuÃ±iz, Ricardo Grande, Rosa MarÃ­a GutiÃ©rrez Rios, JesÃºs HernÃ¡ndez, Alejandra HernÃ¡ndez-TerÃ¡n, Alfredo Herrera-Estrella, Carla IvÃ³n Herrera-Najera, Pavel Isa, VerÃ³nica Mata-Haro, Daniel Lira Morales, Susana Lopez, Antonio Loza RomÃ¡n, Brenda Irasema Maldonado-Meza, Bernardo MartÃ­nez-Miguel, JosÃ© Arturo MartÃ­nez-Orozco, CÃ©lida MartÃ­nez- RodrÃ­guez, Margarita MatÃ­as-Florentino, Fidencio MejÃ­a-Nepomuceno, MarÃ­a Guadalupe de JesÃºs Mireles-Rivera, Gloria MarÃ­a Molina-Salinas, Hector Montoya-Fuentes, , Mario MÃºjica-SÃ¡nchez, JosÃ© Esteban MuÃ±oz-Medina, JosÃ© de JesÃºs NuÃ±ez-Contreras, Alicia OcaÃ±a-MondragÃ³n, Luis Alberto Ochoa-Carrera, Hector Esteban Paz-JuÃ¡rez, Marissa Perez-Garcia, Francisco Pulido, Helen Haydee Fernanda RamÃ­rez-Plascencia, Jorge Salas-HernÃ¡ndez, Angel Gustavo Salas-Lais, Alejandro SÃ¡nchez-Flores, Clara Esperanza Santacruz-Tinoco, MarÃ­a Guadalupe Santiago-Mauricio, Selene ZÃ¡rate, Nelly SÃ©lem-Mojica, Blanca Taboada, Gloria Vazquez, Joel Armando VÃ¡zquez-PÃ©rez. |
| EPI_ISL_2490324 | hCoV-19/Mexico/CHH_INER_IMSS_1094/2021 | North America/Mexico/Chihuahua | 2021-05-04 | Centro de InvestigaciÃ³n BiomÃ©dica del Noreste (CIBIN) | Centro de InvestigaciÃ³n en Enfermedades Infecciosas (CIENI), Instituto Nacional de Enfermedades Respiratorias (INER) | Consorcio Mexicano de Vigilancia GenÃ³mica (CoViGen-Mex). Authors (in alphabetical order): Julio Elias Alvarado-Yaah, Carlos F. Arias, Santiago Ãvila-RÃ­os, Eduardo Becerril-Vargas, VÃ­ctor Hugo Borja-Aburto, Celia Boukadida, CristÃ³bal ChÃ¡idez-QuirÃ³z, Juan Bautista Chale-Dzul, Ricardo Ciria Merce, Andreu Comas-GarcÃ­a, CÃ©lida Duque Molina, Julissa Enciso-Ibarra, JosÃ© Antonio Enciso-Moreno, Gloria Elena Espinosa-Ayala, Fernando Fontove-Herrera, Daniel Fregoso-Rueda, Victor Eduardo Garcia-Arias, Alejandra Garcia-Gasca, Bruno Gomez-Gil, Jean Pierre GonzÃ¡lez, Irvin GonzÃ¡lez-LÃ³pez, ConcepciÃ³n Grajales-MuÃ±iz, Ricardo Grande, Rosa MarÃ­a Gutierrez Rios, Alejandra HernÃ¡ndez-TerÃ¡n, Alfredo Herrera-Estrella, Carla IvÃ³n Herrera-Najera, Pavel Isa, Daniel Lira Morales, Susana Lopez, Antonio Loza RomÃ¡n, Brenda Irasema Maldonado-Meza, Bernardo MartÃ­nez-Miguel, JosÃ© Arturo MartÃ­nez-Orozco, CÃ©lida MartÃ­nez- RodrÃ­guez, Margarita MatÃ­as-Florentino, Fidencio MejÃ­a-Nepomuceno, MarÃ­a Guadalupe de JesÃºs Mireles-Rivera, Gloria MarÃ­a Molina-Salinas, Hector Montoya-Fuentes, , Mario MÃºjica-SÃ¡nchez, JosÃ© Esteban MuÃ±oz-Medina, JosÃ© de JesÃºs NuÃ±ez-Contreras, Alicia OcaÃ±a-MondragÃ³n, Luis Alberto Ochoa-Carrera, Hector Esteban Paz-JuÃ¡rez, Marissa Perez-Garcia, Francisco Pulido, Helen Haydee Fernanda RamÃ­rez-Plascencia, Jorge Salas-HernÃ¡ndez, Angel Gustavo Salas-Lais, Alejandro Sanchez-Flores, Clara Esperanza Santacruz-Tinoco, MarÃ­a Guadalupe Santiago-Mauricio, Selene ZÃ¡rate, Nelly SÃ©lem-Mojica, Blanca Taboada, Gloria Vazquez, Joel Armando VÃ¡zquez-PÃ©rez. |
| EPI_ISL_1279266 | hCoV-19/Mexico/CHH-INER-IMSS-00220/2021 | North America/Mexico/Chihuahua | 2021-02-10 | Centro de InvestigaciÃ³n BiomÃ©dica del Noreste (CIBIN) | Instituto Nacional de Enfermedades Respiratorias (INER): Centro de InvestigaciÃ³n en Enfermedades Infecciosas (CIENI) | Consorcio Mexicano de Vigilancia GenÃ³mica (CoViGen-Mex). Authors (in alphabetical order): |
| EPI_ISL_2681095 | hCoV-19/Mexico/COA_IBT_IMSS_1400/2021 | North America/Mexico/Coahuila | 2021-05-29 | Centro de InvestigaciÃ³n BiomÃ©dica del Noreste (CIBIN) | Instituto de BiotecnologÃ­a de la UNAM | Consorcio Mexicano de Vigilancia GenÃ³mica (CoViGen-Mex). Authors (in alphabetical order): Julio Elias Alvarado-Yaah, Carlos F. Arias, Santiago Ãvila-RÃ­os, Eduardo Becerril-Vargas, VÃ­ctor Hugo Borja-Aburto, Celia Boukadida, CristÃ³bal ChÃ¡idez-QuirÃ³z, Juan Bautista Chale-Dzul, Ricardo Ciria Merce, Andreu Comas-GarcÃ­a, CÃ©lida Duque Molina, Julissa Enciso-Ibarra, JosÃ© Antonio Enciso-Moreno, Gloria Elena Espinosa-Ayala, Fernando Fontove-Herrera, Daniel Fregoso-Rueda, Victor Eduardo GarcÃ­a-Arias, Alejandra GarcÃ­a-Gasca, Bruno GÃ³mez-Gil, Jean Pierre GonzÃ¡lez, Irvin GonzÃ¡lez-LÃ³pez, ConcepciÃ³n Grajales-MuÃ±iz, Ricardo Grande, Rosa MarÃ­a GutiÃ©rrez Rios, JesÃºs HernÃ¡ndez, Alejandra HernÃ¡ndez-TerÃ¡n, Alfredo Herrera-Estrella, Carla IvÃ³n Herrera-Najera, Pavel Isa, VerÃ³nica Mata-Haro, Daniel Lira Morales, Susana Lopez, Antonio Loza RomÃ¡n, Brenda Irasema Maldonado-Meza, Bernardo MartÃ­nez-Miguel, JosÃ© Arturo MartÃ­nez-Orozco, CÃ©lida MartÃ­nez- RodrÃ­guez, Margarita MatÃ­as-Florentino, Fidencio MejÃ­a-Nepomuceno, MarÃ­a Guadalupe de JesÃºs Mireles-Rivera, Gloria MarÃ­a Molina-Salinas, Hector Montoya-Fuentes, , Mario MÃºjica-SÃ¡nchez, JosÃ© Esteban MuÃ±oz-Medina, JosÃ© de JesÃºs NuÃ±ez-Contreras, Alicia OcaÃ±a-MondragÃ³n, Luis Alberto Ochoa-Carrera, Hector Esteban Paz-JuÃ¡rez, Marissa Perez-Garcia, Francisco Pulido, Helen Haydee Fernanda RamÃ­rez-Plascencia, Jorge Salas-HernÃ¡ndez, Angel Gustavo Salas-Lais, Alejandro SÃ¡nchez-Flores, Clara Esperanza Santacruz-Tinoco, MarÃ­a Guadalupe Santiago-Mauricio, Selene ZÃ¡rate, Nelly SÃ©lem-Mojica, Blanca Taboada, Gloria Vazquez, Joel Armando VÃ¡zquez-PÃ©rez. |
| EPI_ISL_3347610 | hCoV-19/Mexico/DUR_IBT_IMSS_2363/2021 | North America/Mexico/Durango | 2021-07-20 | Centro de InvestigaciÃ³n BiomÃ©dica del Noreste (CIBIN) | Instituto de BiotecnologÃ­a de la UNAM | Consorcio Mexicano de Vigilancia GenÃ³mica (CoViGen-Mex). Authors (in alphabetical order): Julio Elias Alvarado-Yaah, Carlos F. Arias, Santiago Ãvila-RÃ­os, Eduardo Becerril-Vargas, VÃ­ctor Hugo Borja-Aburto, Celia Boukadida, CristÃ³bal ChÃ¡idez-QuirÃ³z, Juan Bautista Chale-Dzul, Ricardo Ciria Merce, Andreu Comas-GarcÃ­a, CÃ©lida Duque Molina, Julissa Enciso-Ibarra, JosÃ© Antonio Enciso-Moreno, Gloria Elena Espinosa-Ayala, Fernando Fontove-Herrera, Daniel Fregoso-Rueda, Victor Eduardo GarcÃ­a-Arias, Alejandra GarcÃ­a-Gasca, Bruno GÃ³mez-Gil, Jean Pierre GonzÃ¡lez, Irvin GonzÃ¡lez-LÃ³pez, ConcepciÃ³n Grajales-MuÃ±iz, Ricardo Grande, Rosa MarÃ­a GutiÃ©rrez Rios, JesÃºs HernÃ¡ndez, Alejandra HernÃ¡ndez-TerÃ¡n, Alfredo Herrera-Estrella, Carla IvÃ³n Herrera-Najera, Pavel Isa, VerÃ³nica Mata-Haro, Daniel Lira Morales, Susana Lopez, Antonio Loza RomÃ¡n, Brenda Irasema Maldonado-Meza, Bernardo MartÃ­nez-Miguel, JosÃ© Arturo MartÃ­nez-Orozco, CÃ©lida MartÃ­nez- RodrÃ­guez, Margarita MatÃ­as-Florentino, Fidencio MejÃ­a-Nepomuceno, MarÃ­a Guadalupe de JesÃºs Mireles-Rivera, Gloria MarÃ­a Molina-Salinas, Hector Montoya-Fuentes, , Mario MÃºjica-SÃ¡nchez, JosÃ© Esteban MuÃ±oz-Medina, JosÃ© de JesÃºs NuÃ±ez-Contreras, Alicia OcaÃ±a-MondragÃ³n, Luis Alberto Ochoa-Carrera, Hector Esteban Paz-JuÃ¡rez, Marissa Perez-Garcia, Francisco Pulido, Helen Haydee Fernanda RamÃ­rez-Plascencia, Jorge Salas-HernÃ¡ndez, Angel Gustavo Salas-Lais, Alejandro SÃ¡nchez-Flores, Clara Esperanza Santacruz-Tinoco, MarÃ­a Guadalupe Santiago-Mauricio, Selene ZÃ¡rate, Nelly SÃ©lem-Mojica, Blanca Taboada, Kathia Elizabeth Tapia-Diaz, Gloria Vazquez, Joel Armando VÃ¡zquez-PÃ©rez). |
| EPI_ISL_1279465 | hCoV-19/Mexico/DUR-INER-IMSS-00194/2021 | North America/Mexico/Durango | 2021-02-08 | Centro de InvestigaciÃ³n BiomÃ©dica del Noreste (CIBIN) | Instituto Nacional de Enfermedades Respiratorias (INER): Centro de InvestigaciÃ³n en Enfermedades Infecciosas (CIENI) | Consorcio Mexicano de Vigilancia GenÃ³mica (CoViGen-Mex). Authors (in alphabetical order): |
| EPI_ISL_1287772 | hCoV-19/Mexico/DUR-INER-IMSS-00273/2021 | North America/Mexico/Durango | 2021-02-16 | Centro de InvestigaciÃ³n BiomÃ©dica del Noreste (CIBIN) | Instituto Nacional de Enfermedades Respiratorias (INER): Centro de InvestigaciÃ³n en Enfermedades Infecciosas (CIENI) | Consorcio Mexicano de Vigilancia GenÃ³mica (CoViGen-Mex). Authors (in alphabetical order): |
| EPI_ISL_3347666 | hCoV-19/Mexico/NLE_IBT_IMSS_2361/2021 | North America/Mexico/Nuevo Leon | 2021-07-18 | Centro de InvestigaciÃ³n BiomÃ©dica del Noreste (CIBIN) | Instituto de BiotecnologÃ­a de la UNAM | Consorcio Mexicano de Vigilancia GenÃ³mica (CoViGen-Mex). Authors (in alphabetical order): Julio Elias Alvarado-Yaah, Carlos F. Arias, Santiago Ãvila-RÃ­os, Eduardo Becerril-Vargas, VÃ­ctor Hugo Borja-Aburto, Celia Boukadida, CristÃ³bal ChÃ¡idez-QuirÃ³z, Juan Bautista Chale-Dzul, Ricardo Ciria Merce, Andreu Comas-GarcÃ­a, CÃ©lida Duque Molina, Julissa Enciso-Ibarra, JosÃ© Antonio Enciso-Moreno, Gloria Elena Espinosa-Ayala, Fernando Fontove-Herrera, Daniel Fregoso-Rueda, Victor Eduardo GarcÃ­a-Arias, Alejandra GarcÃ­a-Gasca, Bruno GÃ³mez-Gil, Jean Pierre GonzÃ¡lez, Irvin GonzÃ¡lez-LÃ³pez, ConcepciÃ³n Grajales-MuÃ±iz, Ricardo Grande, Rosa MarÃ­a GutiÃ©rrez Rios, JesÃºs HernÃ¡ndez, Alejandra HernÃ¡ndez-TerÃ¡n, Alfredo Herrera-Estrella, Carla IvÃ³n Herrera-Najera, Pavel Isa, VerÃ³nica Mata-Haro, Daniel Lira Morales, Susana Lopez, Antonio Loza RomÃ¡n, Brenda Irasema Maldonado-Meza, Bernardo MartÃ­nez-Miguel, JosÃ© Arturo MartÃ­nez-Orozco, CÃ©lida MartÃ­nez- RodrÃ­guez, Margarita MatÃ­as-Florentino, Fidencio MejÃ­a-Nepomuceno, MarÃ­a Guadalupe de JesÃºs Mireles-Rivera, Gloria MarÃ­a Molina-Salinas, Hector Montoya-Fuentes, , Mario MÃºjica-SÃ¡nchez, JosÃ© Esteban MuÃ±oz-Medina, JosÃ© de JesÃºs NuÃ±ez-Contreras, Alicia OcaÃ±a-MondragÃ³n, Luis Alberto Ochoa-Carrera, Hector Esteban Paz-JuÃ¡rez, Marissa Perez-Garcia, Francisco Pulido, Helen Haydee Fernanda RamÃ­rez-Plascencia, Jorge Salas-HernÃ¡ndez, Angel Gustavo Salas-Lais, Alejandro SÃ¡nchez-Flores, Clara Esperanza Santacruz-Tinoco, MarÃ­a Guadalupe Santiago-Mauricio, Selene ZÃ¡rate, Nelly SÃ©lem-Mojica, Blanca Taboada, Kathia Elizabeth Tapia-Diaz, Gloria Vazquez, Joel Armando VÃ¡zquez-PÃ©rez). |
| EPI_ISL_4299472 | hCoV-19/Mexico/NLE_INER_IMSS_2049/2021 | North America/Mexico/Nuevo Leon | 2021-08-23 | Centro de InvestigaciÃ³n BiomÃ©dica del Noreste (CIBIN) | Centro de InvestigaciÃ³n en Enfermedades Infecciosas (CIENI), Instituto Nacional de Enfermedades Respiratorias (INER) | Consorcio Mexicano de Vigilancia GenÃ³mica (CoViGen-Mex). Authors (in alphabetical order): Julio Elias Alvarado-Yaah, Carlos F. Arias, Santiago Ãvila-RÃ­os, Eduardo Becerril-Vargas, VÃ­ctor Hugo Borja-Aburto, Celia Boukadida, CristÃ³bal ChÃ¡idez-QuirÃ³z, Juan Bautista Chale-Dzul, Gabriel Chavira-Trujillo, Ricardo Ciria Merce, Andreu Comas-GarcÃ­a, CÃ©lida Duque Molina, Julissa Enciso-Ibarra, JosÃ© Antonio Enciso-Moreno, Gloria Elena Espinosa-Ayala, Fernando Fontove-Herrera, Daniel Fregoso-Rueda, Victor Eduardo GarcÃ­a-Arias, Alejandra GarcÃ­a-Gasca, Bruno GÃ³mez-Gil, Jean Pierre GonzÃ¡lez, Irvin GonzÃ¡lez-LÃ³pez, ConcepciÃ³n Grajales-MuÃ±iz, Ricardo Grande, Rosa MarÃ­a GutiÃ©rrez Rios, JesÃºs HernÃ¡ndez, Alejandra HernÃ¡ndez-TerÃ¡n, Alfredo Herrera-Estrella, Carla IvÃ³n Herrera-Najera, Pavel Isa, MarÃ­a Eugenia JimÃ©nez-Corona, Daniel Lira Morales, Susana Lopez, Antonio Loza RomÃ¡n, Brenda Irasema Maldonado-Meza, Bernardo MartÃ­nez-Miguel, JosÃ© Arturo MartÃ­nez-Orozco, CÃ©lida MartÃ­nez-RodrÃ­guez, VerÃ³nica Mata-Haro, Margarita MatÃ­as-Florentino, Fidencio MejÃ­a-Nepomuceno, MarÃ­a Guadalupe de JesÃºs Mireles-Rivera, Gloria MarÃ­a Molina-Salinas, Hector Montoya-Fuentes, Mario MÃºjica-SÃ¡nchez, JosÃ© Esteban MuÃ±oz-Medina, JosÃ© de JesÃºs NuÃ±ez-Contreras, Alicia OcaÃ±a-MondragÃ³n, Luis Alberto Ochoa-Carrera, Marissa Perez-Garcia, Francisco Pulido, Helen Haydee Fernanda RamÃ­rez-Plascencia, Eduardo Rivera-MartÃ­nez, Jorge Salas-HernÃ¡ndez, Angel Gustavo Salas-Lais, Alejandro SÃ¡nchez-Flores, Clara Esperanza Santacruz-Tinoco, MarÃ­a Guadalupe Santiago-Mauricio, Selene ZÃ¡rate, Nelly SÃ©lem-Mojica, Blanca Taboada, Kathia Elizabeth Tapia-Diaz, Gloria Vazquez, Rosario Vazquez-Larios, Joel Armando VÃ¡zquez-PÃ©rez. |
| EPI_ISL_3155403 | hCoV-19/Mexico/NLE-INER_IMSS_1393/2021 | North America/Mexico/Nuevo Leon | 2021-07-05 | Centro de InvestigaciÃ³n BiomÃ©dica del Noreste (CIBIN) | Centro de InvestigaciÃ³n en Enfermedades Infecciosas (CIENI), Instituto Nacional de Enfermedades Respiratorias (INER) | Consorcio Mexicano de Vigilancia GenÃ³mica (CoViGen-Mex). Authors (in alphabetical order): Julio Elias Alvarado-Yaah, Carlos F. Arias, Santiago Ãvila-RÃ­os, Eduardo Becerril-Vargas, VÃ­ctor Hugo Borja-Aburto, Celia Boukadida, CristÃ³bal ChÃ¡idez-QuirÃ³z, Juan Bautista Chale-Dzul, Ricardo Ciria Merce, Andreu Comas-GarcÃ­a, CÃ©lida Duque Molina, Julissa Enciso-Ibarra, JosÃ© Antonio Enciso-Moreno, Gloria Elena Espinosa-Ayala, Fernando Fontove-Herrera, Daniel Fregoso-Rueda, Victor Eduardo GarcÃ­a-Arias, Alejandra GarcÃ­a-Gasca, Bruno GÃ³mez-Gil, Jean Pierre GonzÃ¡lez, Irvin GonzÃ¡lez-LÃ³pez, ConcepciÃ³n Grajales-MuÃ±iz, Ricardo Grande, Rosa MarÃ­a GutiÃ©rrez Rios, JesÃºs HernÃ¡ndez, Alejandra HernÃ¡ndez-TerÃ¡n, Alfredo Herrera-Estrella, Carla IvÃ³n Herrera-Najera, Pavel Isa, VerÃ³nica Mata-Haro, Daniel Lira Morales, Susana Lopez, Antonio Loza RomÃ¡n, Brenda Irasema Maldonado-Meza, Bernardo MartÃ­nez-Miguel, JosÃ© Arturo MartÃ­nez-Orozco, CÃ©lida MartÃ­nez- RodrÃ­guez, Margarita MatÃ­as-Florentino, Fidencio MejÃ­a-Nepomuceno, MarÃ­a Guadalupe de JesÃºs Mireles-Rivera, Gloria MarÃ­a Molina-Salinas, Hector Montoya-Fuentes, , Mario MÃºjica-SÃ¡nchez, JosÃ© Esteban MuÃ±oz-Medina, JosÃ© de JesÃºs NuÃ±ez-Contreras, Alicia OcaÃ±a-MondragÃ³n, Luis Alberto Ochoa-Carrera, Hector Esteban Paz-JuÃ¡rez, Marissa Perez-Garcia, Francisco Pulido, Helen Haydee Fernanda RamÃ­rez-Plascencia, Jorge Salas-HernÃ¡ndez, Angel Gustavo Salas-Lais, Alejandro SÃ¡nchez-Flores, Clara Esperanza Santacruz-Tinoco, MarÃ­a Guadalupe Santiago-Mauricio, Selene ZÃ¡rate, Nelly SÃ©lem-Mojica, Blanca Taboada, Kathia Elizabeth Tapia-Diaz, Gloria Vazquez, Joel Armando VÃ¡zquez-PÃ©rez. |
| EPI_ISL_2401973 | hCoV-19/Mexico/TAM_LANGEBIO_IMSS_0550/2021 | North America/Mexico/Tamaulipas | 2021-04-25 | Centro de InvestigaciÃ³n BiomÃ©dica del Noreste (CIBIN) | Unidad de Genomica Avanzada | Consorcio Mexicano de Vigilancia Genomica (CoViGen-Mex). Authors (in alphabetical order): Julio Elias Alvarado-Yaah, Carlos F. Arias, Santiago Avila-Rios, Victor Hugo Borja-Aburto, Celia Boukadida, Juan Bautista Chale-Dzul , Jose Antonio Enciso-Moreno, Gloria Elena Espinoza-Ayala, Fernando Fontove-Herrera, Concepcion Grajales-Muniz, Ricardo Grande, Alfredo Herrera-Estrella, Carla Ivon Herrera-Najera, Pavel Isa, Brenda Irasema Maldonado-Meza, Bernardo Martinez-Miguel, Margarita Matias-Florentino, Maria Guadalupe de Jesus Mireles-Rivera, Gloria Maria Molina-Salinas, Hector Montoya-Fuentes, Jose Esteban Munoz-Medina, Jose de Jesus Nunez-Contreras, Alicia Ocana-Mondragon, Luis Alberto Ochoa-Carrera, Hector Esteban Paz-Juarez, Francisco Pulido, Helen Haydee Fernanda Ramirez-Plascencia, Angel Gustavo Salas-Lais, Jorge Ivan Salinal-Nevarez, Alejandro Sanchez-Flores, Clara Esperanza Santacruz-Tinoco, Maria Guadalupe Santiago-Mauricio, Nelly Selem-Mojica, Blanca Taboada, Gloria Vazquez |
| EPI_ISL_1500866 | hCoV-19/Mexico/SLP-AH1COV2SS035_S24SLP-UASLP/2020 | North America/Mexico/San Luis Potosi | 2020-07-27 | Centro de InvestigaciÃ³n en Ciencias de la Salud y Biomedicina, U.A.S.L.P. | Centro de InvestigaciÃ³n en Ciencias de la Salud y Biomedicina, U.A.S.L.P. | MD PhD SofÃ­a Bernal Silva |
| EPI_ISL_1494727 | hCoV-19/Mexico/SLP-UASLP-AH1COV2SS029_S18/2020 | North America/Mexico/San Luis Potosi | 2020-05-26 | Centro de InvestigaciÃ³n en Ciencias de la Salud y Biomedicina, U.A.S.LP. | Centro de InvestigaciÃ³n en Ciencias de la Salud y Biomedicina, U.A.S.LP. | MD PhD SofÃ­a Bernal Silva |
| EPI_ISL_1351552 | hCoV-19/Mexico/TAM_LANGEBIO_IMSS_0053/2021 | North America/Mexico/Sonora | 2021-02-27 | Centro de InvestigaciÃƒÂ³n BiomÃƒÂ©dica de Occidente (CIBO) | Unidad de Genomica Avanzada | Consorcio Mexicano de Vigilancia Genomica (CoViGen-Mex). Authors (in alphabetical order): Julio Elias Alvarado-Yaah, Carlos F. Arias, Santiago Avila-Rios, Victor Hugo Borja-Aburto, Celia Boukadida, Juan Bautista Chale-Dzul , Jose Antonio Enciso-Moreno, Gloria Elena Espinoza-Ayala, Fernando Fontove-Herrera, Concepcion Grajales-Muniz, Ricardo Grande, Alfredo Herrera-Estrella, Carla Ivon Herrera-Najera, Pavel Isa, Brenda Irasema Maldonado-Meza, Bernardo Martinez-Miguel, Margarita Matias-Florentino, Maria Guadalupe de Jesus Mireles-Rivera, Gloria Maria Molina-Salinas, Hector Montoya-Fuentes, Jose Esteban Munoz-Medina, Jose de Jesus Nunez-Contreras, Alicia Ocana-Mondragon, Luis Alberto Ochoa-Carrera, Hector Esteban Paz-Juarez, Francisco Pulido, Helen Haydee Fernanda Ramirez-Plascencia, Angel Gustavo Salas-Lais, Jorge Ivan Salinal-Nevarez, Alejandro Sanchez-Flores, Clara Esperanza Santacruz-Tinoco, Maria Guadalupe Santiago-Mauricio, Nelly Selem-Mojica, Blanca Taboada, Gloria Vazquez |
| EPI_ISL_1351608 | hCoV-19/Mexico/TAM_LANGEBIO_IMSS_0071/2021 | North America/Mexico/Jalisco | 2021-03-05 | Centro de InvestigaciÃƒÂ³n BiomÃƒÂ©dica de Occidente (CIBO) | Unidad de Genomica Avanzada | Consorcio Mexicano de Vigilancia Genomica (CoViGen-Mex). Authors (in alphabetical order): Julio Elias Alvarado-Yaah, Carlos F. Arias, Santiago Avila-Rios, Victor Hugo Borja-Aburto, Celia Boukadida, Juan Bautista Chale-Dzul , Jose Antonio Enciso-Moreno, Gloria Elena Espinoza-Ayala, Fernando Fontove-Herrera, Concepcion Grajales-Muniz, Ricardo Grande, Alfredo Herrera-Estrella, Carla Ivon Herrera-Najera, Pavel Isa, Brenda Irasema Maldonado-Meza, Bernardo Martinez-Miguel, Margarita Matias-Florentino, Maria Guadalupe de Jesus Mireles-Rivera, Gloria Maria Molina-Salinas, Hector Montoya-Fuentes, Jose Esteban Munoz-Medina, Jose de Jesus Nunez-Contreras, Alicia Ocana-Mondragon, Luis Alberto Ochoa-Carrera, Hector Esteban Paz-Juarez, Francisco Pulido, Helen Haydee Fernanda Ramirez-Plascencia, Angel Gustavo Salas-Lais, Jorge Ivan Salinal-Nevarez, Alejandro Sanchez-Flores, Clara Esperanza Santacruz-Tinoco, Maria Guadalupe Santiago-Mauricio, Nelly Selem-Mojica, Blanca Taboada, Gloria Vazquez |
| EPI_ISL_3805400 | hCoV-19/Mexico/COL_LANGEBIO_IMSS_2318/2021 | North America/Mexico/Colima | 2021-08-05 | Centro de Investigacion Biomedica de Occidente (CIBO) | Unidad de Genomica Avanzada | Consorcio Mexicano de Vigilancia Genomica (CoViGen-Mex). Authors (in alphabetical order): Julio Elias Alvarado-Yaah, Carlos F. Arias, Santiago avila-Rios, Eduardo Becerril-Vargas, Victor Hugo Borja-Aburto, Celia Boukadida, Cristobal Chaidez-Quiroz, Juan Bautista Chale-Dzul, Ricardo Ciria Merce, Andreu Comas-Garcia, Celida Duque Molina, Julissa Enciso-Ibarra, Jose Antonio Enciso-Moreno, Gloria Elena Espinosa-Ayala, Fernando Fontove-Herrera, Daniel Fregoso-Rueda, Victor Eduardo Garcia-Arias, Alejandra Garcia-Gasca, Bruno Gomez-Gil, Jean Pierre Gonzalez, Irvin Gonzalez-Lopez, Concepcion Grajales-MuÃ±iz, Ricardo Grande, Rosa Maria Gutierrez Rios, Jesus Hernandez, Alejandra Hernandez-Teran, Alfredo Herrera-Estrella, Carla Ivon Herrera-Najera, Pavel Isa, Veronica Mata-Haro, Daniel Lira Morales, Susana Lopez, Antonio Loza Roman, Brenda Irasema Maldonado-Meza, Bernardo Martinez-Miguel, Jose Arturo Martinez-Orozco, Celida Martinez- Rodriguez, Margarita Matias-Florentino, Fidencio Mejia-Nepomuceno, Maria Guadalupe de Jesus Mireles-Rivera, Gloria Maria Molina-Salinas, Hector Montoya-Fuentes, , Mario Mujica-Sanchez, Jose Esteban MuÃ±oz-Medina, Jose de Jesus NuÃ±ez-Contreras, Alicia OcaÃ±a-Mondragon, Luis Alberto Ochoa-Carrera, Hector Esteban Paz-Juarez, Marissa Perez-Garcia, Francisco Pulido, Helen Haydee Fernanda Ramirez-Plascencia, Jorge Salas-Hernandez, Angel Gustavo Salas-Lais, Alejandro Sanchez-Flores, Clara Esperanza Santacruz-Tinoco, Maria Guadalupe Santiago-Mauricio, Selene Zarate, Nelly Selem-Mojica, Blanca Taboada, Gloria Vazquez, Joel Armando Vazquez-Perez. |
| EPI_ISL_2671554 | hCoV-19/Mexico/COL-LANGEBIO_IMSS_1033/2021 | North America/Mexico/Colima | 2021-05-24 | Centro de Investigacion Biomedica de Occidente (CIBO) | Unidad de Genomica Avanzada | Consorcio Mexicano de Vigilancia Genomica (CoViGen-Mex). Authors (in alphabetical order): Julio Elias Alvarado-Yaah, Carlos F. Arias, Santiago avila-Rios, Eduardo Becerril-Vargas, Victor Hugo Borja-Aburto, Celia Boukadida, Cristobal Chaidez-Quiroz, Juan Bautista Chale-Dzul, Ricardo Ciria Merce, Andreu Comas-Garcia, Celida Duque Molina, Julissa Enciso-Ibarra, Jose Antonio Enciso-Moreno, Gloria Elena Espinosa-Ayala, Fernando Fontove-Herrera, Daniel Fregoso-Rueda, Victor Eduardo Garcia-Arias, Alejandra Garcia-Gasca, Bruno Gomez-Gil, Jean Pierre Gonzalez, Irvin Gonzalez-Lopez, Concepcion Grajales-MuÃ±iz, Ricardo Grande, Rosa Maria Gutierrez Rios, Jesus Hernandez, Alejandra Hernandez-Teran, Alfredo Herrera-Estrella, Carla Ivon Herrera-Najera, Pavel Isa, Veronica Mata-Haro, Daniel Lira Morales, Susana Lopez, Antonio Loza Roman, Brenda Irasema Maldonado-Meza, Bernardo Martinez-Miguel, Jose Arturo Martinez-Orozco, Celida Martinez- Rodriguez, Margarita Matias-Florentino, Fidencio Mejia-Nepomuceno, Maria Guadalupe de Jesus Mireles-Rivera, Gloria Maria Molina-Salinas, Hector Montoya-Fuentes, , Mario Mujica-Sanchez, Jose Esteban MuÃ±oz-Medina, Jose de Jesus NuÃ±ez-Contreras, Alicia OcaÃ±a-Mondragon, Luis Alberto Ochoa-Carrera, Hector Esteban Paz-Juarez, Marissa Perez-Garcia, Francisco Pulido, Helen Haydee Fernanda Ramirez-Plascencia, Jorge Salas-Hernandez, Angel Gustavo Salas-Lais, Alejandro Sanchez-Flores, Clara Esperanza Santacruz-Tinoco, Maria Guadalupe Santiago-Mauricio, Selene Zarate, Nelly Selem-Mojica, Blanca Taboada, Gloria Vazquez, Joel Armando Vazquez-Perez. |
| EPI_ISL_2671567 | hCoV-19/Mexico/GUA-LANGEBIO_IMSS_1013/2021 | North America/Mexico/Guanajuato | 2021-05-21 | Centro de Investigacion Biomedica de Occidente (CIBO) | Unidad de Genomica Avanzada | Consorcio Mexicano de Vigilancia Genomica (CoViGen-Mex). Authors (in alphabetical order): Julio Elias Alvarado-Yaah, Carlos F. Arias, Santiago avila-Rios, Eduardo Becerril-Vargas, Victor Hugo Borja-Aburto, Celia Boukadida, Cristobal Chaidez-Quiroz, Juan Bautista Chale-Dzul, Ricardo Ciria Merce, Andreu Comas-Garcia, Celida Duque Molina, Julissa Enciso-Ibarra, Jose Antonio Enciso-Moreno, Gloria Elena Espinosa-Ayala, Fernando Fontove-Herrera, Daniel Fregoso-Rueda, Victor Eduardo Garcia-Arias, Alejandra Garcia-Gasca, Bruno Gomez-Gil, Jean Pierre Gonzalez, Irvin Gonzalez-Lopez, Concepcion Grajales-MuÃ±iz, Ricardo Grande, Rosa Maria Gutierrez Rios, Jesus Hernandez, Alejandra Hernandez-Teran, Alfredo Herrera-Estrella, Carla Ivon Herrera-Najera, Pavel Isa, Veronica Mata-Haro, Daniel Lira Morales, Susana Lopez, Antonio Loza Roman, Brenda Irasema Maldonado-Meza, Bernardo Martinez-Miguel, Jose Arturo Martinez-Orozco, Celida Martinez- Rodriguez, Margarita Matias-Florentino, Fidencio Mejia-Nepomuceno, Maria Guadalupe de Jesus Mireles-Rivera, Gloria Maria Molina-Salinas, Hector Montoya-Fuentes, , Mario Mujica-Sanchez, Jose Esteban MuÃ±oz-Medina, Jose de Jesus NuÃ±ez-Contreras, Alicia OcaÃ±a-Mondragon, Luis Alberto Ochoa-Carrera, Hector Esteban Paz-Juarez, Marissa Perez-Garcia, Francisco Pulido, Helen Haydee Fernanda Ramirez-Plascencia, Jorge Salas-Hernandez, Angel Gustavo Salas-Lais, Alejandro Sanchez-Flores, Clara Esperanza Santacruz-Tinoco, Maria Guadalupe Santiago-Mauricio, Selene Zarate, Nelly Selem-Mojica, Blanca Taboada, Gloria Vazquez, Joel Armando Vazquez-Perez. |
| EPI_ISL_3805367 | hCoV-19/Mexico/JAL_LANGEBIO_IMSS_2213/2021 | North America/Mexico/Jalisco | 2021-08-03 | Centro de Investigacion Biomedica de Occidente (CIBO) | Unidad de Genomica Avanzada | Consorcio Mexicano de Vigilancia Genomica (CoViGen-Mex). Authors (in alphabetical order): Julio Elias Alvarado-Yaah, Carlos F. Arias, Santiago avila-Rios, Eduardo Becerril-Vargas, Victor Hugo Borja-Aburto, Celia Boukadida, Cristobal Chaidez-Quiroz, Juan Bautista Chale-Dzul, Ricardo Ciria Merce, Andreu Comas-Garcia, Celida Duque Molina, Julissa Enciso-Ibarra, Jose Antonio Enciso-Moreno, Gloria Elena Espinosa-Ayala, Fernando Fontove-Herrera, Daniel Fregoso-Rueda, Victor Eduardo Garcia-Arias, Alejandra Garcia-Gasca, Bruno Gomez-Gil, Jean Pierre Gonzalez, Irvin Gonzalez-Lopez, Concepcion Grajales-MuÃ±iz, Ricardo Grande, Rosa Maria Gutierrez Rios, Jesus Hernandez, Alejandra Hernandez-Teran, Alfredo Herrera-Estrella, Carla Ivon Herrera-Najera, Pavel Isa, Veronica Mata-Haro, Daniel Lira Morales, Susana Lopez, Antonio Loza Roman, Brenda Irasema Maldonado-Meza, Bernardo Martinez-Miguel, Jose Arturo Martinez-Orozco, Celida Martinez- Rodriguez, Margarita Matias-Florentino, Fidencio Mejia-Nepomuceno, Maria Guadalupe de Jesus Mireles-Rivera, Gloria Maria Molina-Salinas, Hector Montoya-Fuentes, , Mario Mujica-Sanchez, Jose Esteban MuÃ±oz-Medina, Jose de Jesus NuÃ±ez-Contreras, Alicia OcaÃ±a-Mondragon, Luis Alberto Ochoa-Carrera, Hector Esteban Paz-Juarez, Marissa Perez-Garcia, Francisco Pulido, Helen Haydee Fernanda Ramirez-Plascencia, Jorge Salas-Hernandez, Angel Gustavo Salas-Lais, Alejandro Sanchez-Flores, Clara Esperanza Santacruz-Tinoco, Maria Guadalupe Santiago-Mauricio, Selene Zarate, Nelly Selem-Mojica, Blanca Taboada, Gloria Vazquez, Joel Armando Vazquez-Perez. |
| EPI_ISL_1662126 | hCoV-19/Mexico/MIC_LANGEBIO_IMSS_0352/2021 | North America/Mexico/Michoacan | 2021-03-25 | Centro de Investigacion Biomedica de Occidente (CIBO) | Unidad de Genomica Avanzada | Consorcio Mexicano de Vigilancia Genomica (CoViGen-Mex). Authors (in alphabetical order): Julio Elias Alvarado-Yaah, Carlos F. Arias, Santiago Ãvila-Rios, Victor Hugo Borja-Aburto, Celia Boukadida, Juan Bautista Chale-Dzul, Celida Duque Molina, Jose Antonio Enciso-Moreno, Gloria Elena Espinosa-Ayala, Fernando Fontove-Herrera, Victor Eduardo Garcia-Arias,Concepcion Grajales-Muniz, Ricardo Grande, Alfredo Herrera-Estrella, Carla Ivon Herrera-Najera, Pavel Isa, Brenda Irasema Maldonado-Meza, Bernardo Martinez-Miguel, Margarita Matias-Florentino, Maria Guadalupe de Jesus Mireles-Rivera, Gloria Maria Molina-Salinas, Hector Montoya-Fuentes, Jose Esteban Munoz-Medina, Jose de Jesus Nunez-Contreras, Alicia Ocana-Mondragon, Luis Alberto Ochoa-Carrera, Hector Esteban Paz-Juarez, Francisco Pulido, Helen Haydee Fernanda Ramirez-Plascencia, Angel Gustavo Salas-Lais, Alejandro Sanchez-Flores, Clara Esperanza Santacruz-Tinoco, Maria Guadalupe Santiago-Mauricio, Nelly Selem-Mojica, Blanca Taboada, Gloria Vazquez |
| EPI_ISL_3805368 | hCoV-19/Mexico/MIC_LANGEBIO_IMSS_2285/2021 | North America/Mexico/Michoacan | 2021-08-03 | Centro de Investigacion Biomedica de Occidente (CIBO) | Unidad de Genomica Avanzada | Consorcio Mexicano de Vigilancia Genomica (CoViGen-Mex). Authors (in alphabetical order): Julio Elias Alvarado-Yaah, Carlos F. Arias, Santiago avila-Rios, Eduardo Becerril-Vargas, Victor Hugo Borja-Aburto, Celia Boukadida, Cristobal Chaidez-Quiroz, Juan Bautista Chale-Dzul, Ricardo Ciria Merce, Andreu Comas-Garcia, Celida Duque Molina, Julissa Enciso-Ibarra, Jose Antonio Enciso-Moreno, Gloria Elena Espinosa-Ayala, Fernando Fontove-Herrera, Daniel Fregoso-Rueda, Victor Eduardo Garcia-Arias, Alejandra Garcia-Gasca, Bruno Gomez-Gil, Jean Pierre Gonzalez, Irvin Gonzalez-Lopez, Concepcion Grajales-MuÃ±iz, Ricardo Grande, Rosa Maria Gutierrez Rios, Jesus Hernandez, Alejandra Hernandez-Teran, Alfredo Herrera-Estrella, Carla Ivon Herrera-Najera, Pavel Isa, Veronica Mata-Haro, Daniel Lira Morales, Susana Lopez, Antonio Loza Roman, Brenda Irasema Maldonado-Meza, Bernardo Martinez-Miguel, Jose Arturo Martinez-Orozco, Celida Martinez- Rodriguez, Margarita Matias-Florentino, Fidencio Mejia-Nepomuceno, Maria Guadalupe de Jesus Mireles-Rivera, Gloria Maria Molina-Salinas, Hector Montoya-Fuentes, , Mario Mujica-Sanchez, Jose Esteban MuÃ±oz-Medina, Jose de Jesus NuÃ±ez-Contreras, Alicia OcaÃ±a-Mondragon, Luis Alberto Ochoa-Carrera, Hector Esteban Paz-Juarez, Marissa Perez-Garcia, Francisco Pulido, Helen Haydee Fernanda Ramirez-Plascencia, Jorge Salas-Hernandez, Angel Gustavo Salas-Lais, Alejandro Sanchez-Flores, Clara Esperanza Santacruz-Tinoco, Maria Guadalupe Santiago-Mauricio, Selene Zarate, Nelly Selem-Mojica, Blanca Taboada, Gloria Vazquez, Joel Armando Vazquez-Perez. |
| EPI_ISL_2671618 | hCoV-19/Mexico/NAY-LANGEBIO_IMSS_1041/2021 | North America/Mexico/Nayarit | 2021-05-28 | Centro de Investigacion Biomedica de Occidente (CIBO) | Unidad de Genomica Avanzada | Consorcio Mexicano de Vigilancia Genomica (CoViGen-Mex). Authors (in alphabetical order): Julio Elias Alvarado-Yaah, Carlos F. Arias, Santiago avila-Rios, Eduardo Becerril-Vargas, Victor Hugo Borja-Aburto, Celia Boukadida, Cristobal Chaidez-Quiroz, Juan Bautista Chale-Dzul, Ricardo Ciria Merce, Andreu Comas-Garcia, Celida Duque Molina, Julissa Enciso-Ibarra, Jose Antonio Enciso-Moreno, Gloria Elena Espinosa-Ayala, Fernando Fontove-Herrera, Daniel Fregoso-Rueda, Victor Eduardo Garcia-Arias, Alejandra Garcia-Gasca, Bruno Gomez-Gil, Jean Pierre Gonzalez, Irvin Gonzalez-Lopez, Concepcion Grajales-MuÃ±iz, Ricardo Grande, Rosa Maria Gutierrez Rios, Jesus Hernandez, Alejandra Hernandez-Teran, Alfredo Herrera-Estrella, Carla Ivon Herrera-Najera, Pavel Isa, Veronica Mata-Haro, Daniel Lira Morales, Susana Lopez, Antonio Loza Roman, Brenda Irasema Maldonado-Meza, Bernardo Martinez-Miguel, Jose Arturo Martinez-Orozco, Celida Martinez- Rodriguez, Margarita Matias-Florentino, Fidencio Mejia-Nepomuceno, Maria Guadalupe de Jesus Mireles-Rivera, Gloria Maria Molina-Salinas, Hector Montoya-Fuentes, , Mario Mujica-Sanchez, Jose Esteban MuÃ±oz-Medina, Jose de Jesus NuÃ±ez-Contreras, Alicia OcaÃ±a-Mondragon, Luis Alberto Ochoa-Carrera, Hector Esteban Paz-Juarez, Marissa Perez-Garcia, Francisco Pulido, Helen Haydee Fernanda Ramirez-Plascencia, Jorge Salas-Hernandez, Angel Gustavo Salas-Lais, Alejandro Sanchez-Flores, Clara Esperanza Santacruz-Tinoco, Maria Guadalupe Santiago-Mauricio, Selene Zarate, Nelly Selem-Mojica, Blanca Taboada, Gloria Vazquez, Joel Armando Vazquez-Perez. |
| EPI_ISL_2942503 | hCoV-19/Mexico/CHH-LANGEBIO_IMSS_1374/2021 | North America/Mexico/Chihuahua | 2021-06-20 | Centro de Investigacion Biomedica del Noreste (CIBIN) | Unidad de Genomica Avanzada | Consorcio Mexicano de Vigilancia Genomica (CoViGen-Mex). Authors (in alphabetical order): Julio Elias Alvarado-Yaah, Carlos F. Arias, Santiago avila-Rios, Eduardo Becerril-Vargas, Victor Hugo Borja-Aburto, Celia Boukadida, Cristobal Chaidez-Quiroz, Juan Bautista Chale-Dzul, Ricardo Ciria Merce, Andreu Comas-Garcia, Celida Duque Molina, Julissa Enciso-Ibarra, Jose Antonio Enciso-Moreno, Gloria Elena Espinosa-Ayala, Fernando Fontove-Herrera, Daniel Fregoso-Rueda, Victor Eduardo Garcia-Arias, Alejandra Garcia-Gasca, Bruno Gomez-Gil, Jean Pierre Gonzalez, Irvin Gonzalez-Lopez, Concepcion Grajales-MuÃ±iz, Ricardo Grande, Rosa Maria Gutierrez Rios, Jesus Hernandez, Alejandra Hernandez-Teran, Alfredo Herrera-Estrella, Carla Ivon Herrera-Najera, Pavel Isa, Veronica Mata-Haro, Daniel Lira Morales, Susana Lopez, Antonio Loza Roman, Brenda Irasema Maldonado-Meza, Bernardo Martinez-Miguel, Jose Arturo Martinez-Orozco, Celida Martinez- Rodriguez, Margarita Matias-Florentino, Fidencio Mejia-Nepomuceno, Maria Guadalupe de Jesus Mireles-Rivera, Gloria Maria Molina-Salinas, Hector Montoya-Fuentes, , Mario Mujica-Sanchez, Jose Esteban MuÃ±oz-Medina, Jose de Jesus NuÃ±ez-Contreras, Alicia OcaÃ±a-Mondragon, Luis Alberto Ochoa-Carrera, Hector Esteban Paz-Juarez, Marissa Perez-Garcia, Francisco Pulido, Helen Haydee Fernanda Ramirez-Plascencia, Jorge Salas-Hernandez, Angel Gustavo Salas-Lais, Alejandro Sanchez-Flores, Clara Esperanza Santacruz-Tinoco, Maria Guadalupe Santiago-Mauricio, Selene Zarate, Nelly Selem-Mojica, Blanca Taboada, Gloria Vazquez, Joel Armando Vazquez-Perez. |
| EPI_ISL_2942784 | hCoV-19/Mexico/CHH-LANGEBIO_IMSS_1695/2021 | North America/Mexico/Chihuahua | 2021-06-30 | Centro de Investigacion Biomedica del Noreste (CIBIN) | Unidad de Genomica Avanzada | Consorcio Mexicano de Vigilancia Genomica (CoViGen-Mex). Authors (in alphabetical order): Julio Elias Alvarado-Yaah, Carlos F. Arias, Santiago avila-Rios, Eduardo Becerril-Vargas, Victor Hugo Borja-Aburto, Celia Boukadida, Cristobal Chaidez-Quiroz, Juan Bautista Chale-Dzul, Ricardo Ciria Merce, Andreu Comas-Garcia, Celida Duque Molina, Julissa Enciso-Ibarra, Jose Antonio Enciso-Moreno, Gloria Elena Espinosa-Ayala, Fernando Fontove-Herrera, Daniel Fregoso-Rueda, Victor Eduardo Garcia-Arias, Alejandra Garcia-Gasca, Bruno Gomez-Gil, Jean Pierre Gonzalez, Irvin Gonzalez-Lopez, Concepcion Grajales-MuÃ±iz, Ricardo Grande, Rosa Maria Gutierrez Rios, Jesus Hernandez, Alejandra Hernandez-Teran, Alfredo Herrera-Estrella, Carla Ivon Herrera-Najera, Pavel Isa, Veronica Mata-Haro, Daniel Lira Morales, Susana Lopez, Antonio Loza Roman, Brenda Irasema Maldonado-Meza, Bernardo Martinez-Miguel, Jose Arturo Martinez-Orozco, Celida Martinez- Rodriguez, Margarita Matias-Florentino, Fidencio Mejia-Nepomuceno, Maria Guadalupe de Jesus Mireles-Rivera, Gloria Maria Molina-Salinas, Hector Montoya-Fuentes, , Mario Mujica-Sanchez, Jose Esteban MuÃ±oz-Medina, Jose de Jesus NuÃ±ez-Contreras, Alicia OcaÃ±a-Mondragon, Luis Alberto Ochoa-Carrera, Hector Esteban Paz-Juarez, Marissa Perez-Garcia, Francisco Pulido, Helen Haydee Fernanda Ramirez-Plascencia, Jorge Salas-Hernandez, Angel Gustavo Salas-Lais, Alejandro Sanchez-Flores, Clara Esperanza Santacruz-Tinoco, Maria Guadalupe Santiago-Mauricio, Selene Zarate, Nelly Selem-Mojica, Blanca Taboada, Gloria Vazquez, Joel Armando Vazquez-Perez. |
| EPI_ISL_3805431 | hCoV-19/Mexico/COA_LANGEBIO_IMSS_1908/2021 | North America/Mexico/Coahuila | 2021-08-01 | Centro de Investigacion Biomedica del Noreste (CIBIN) | Unidad de Genomica Avanzada | Consorcio Mexicano de Vigilancia Genomica (CoViGen-Mex). Authors (in alphabetical order): Julio Elias Alvarado-Yaah, Carlos F. Arias, Santiago avila-Rios, Eduardo Becerril-Vargas, Victor Hugo Borja-Aburto, Celia Boukadida, Cristobal Chaidez-Quiroz, Juan Bautista Chale-Dzul, Ricardo Ciria Merce, Andreu Comas-Garcia, Celida Duque Molina, Julissa Enciso-Ibarra, Jose Antonio Enciso-Moreno, Gloria Elena Espinosa-Ayala, Fernando Fontove-Herrera, Daniel Fregoso-Rueda, Victor Eduardo Garcia-Arias, Alejandra Garcia-Gasca, Bruno Gomez-Gil, Jean Pierre Gonzalez, Irvin Gonzalez-Lopez, Concepcion Grajales-MuÃ±iz, Ricardo Grande, Rosa Maria Gutierrez Rios, Jesus Hernandez, Alejandra Hernandez-Teran, Alfredo Herrera-Estrella, Carla Ivon Herrera-Najera, Pavel Isa, Veronica Mata-Haro, Daniel Lira Morales, Susana Lopez, Antonio Loza Roman, Brenda Irasema Maldonado-Meza, Bernardo Martinez-Miguel, Jose Arturo Martinez-Orozco, Celida Martinez- Rodriguez, Margarita Matias-Florentino, Fidencio Mejia-Nepomuceno, Maria Guadalupe de Jesus Mireles-Rivera, Gloria Maria Molina-Salinas, Hector Montoya-Fuentes, , Mario Mujica-Sanchez, Jose Esteban MuÃ±oz-Medina, Jose de Jesus NuÃ±ez-Contreras, Alicia OcaÃ±a-Mondragon, Luis Alberto Ochoa-Carrera, Hector Esteban Paz-Juarez, Marissa Perez-Garcia, Francisco Pulido, Helen Haydee Fernanda Ramirez-Plascencia, Jorge Salas-Hernandez, Angel Gustavo Salas-Lais, Alejandro Sanchez-Flores, Clara Esperanza Santacruz-Tinoco, Maria Guadalupe Santiago-Mauricio, Selene Zarate, Nelly Selem-Mojica, Blanca Taboada, Gloria Vazquez, Joel Armando Vazquez-Perez. |
| EPI_ISL_2970002 | hCoV-19/Mexico/NLE_LANGEBIO_IMSS_47239-NC/2021 | North America/Mexico/Nuevo Leon | 2021-03-30 | Centro de Investigacion Biomedica del Noreste (CIBIN) | Unidad de Genomica Avanzada | Consorcio Mexicano de Vigilancia Genomica (CoViGen-Mex). Authors (in alphabetical order): Julio Elias Alvarado-Yaah, Carlos F. Arias, Santiago avila-Rios, Eduardo Becerril-Vargas, Victor Hugo Borja-Aburto, Celia Boukadida, Cristobal Chaidez-Quiroz, Juan Bautista Chale-Dzul, Ricardo Ciria Merce, Andreu Comas-Garcia, Celida Duque Molina, Julissa Enciso-Ibarra, Jose Antonio Enciso-Moreno, Gloria Elena Espinosa-Ayala, Fernando Fontove-Herrera, Daniel Fregoso-Rueda, Victor Eduardo Garcia-Arias, Alejandra Garcia-Gasca, Bruno Gomez-Gil, Jean Pierre Gonzalez, Irvin Gonzalez-Lopez, Concepcion Grajales-MuÃ±iz, Ricardo Grande, Rosa Maria Gutierrez Rios, Jesus Hernandez, Alejandra Hernandez-Teran, Alfredo Herrera-Estrella, Carla Ivon Herrera-Najera, Pavel Isa, Veronica Mata-Haro, Daniel Lira Morales, Susana Lopez, Antonio Loza Roman, Brenda Irasema Maldonado-Meza, Bernardo Martinez-Miguel, Jose Arturo Martinez-Orozco, Celida Martinez- Rodriguez, Margarita Matias-Florentino, Fidencio Mejia-Nepomuceno, Maria Guadalupe de Jesus Mireles-Rivera, Gloria Maria Molina-Salinas, Hector Montoya-Fuentes, , Mario Mujica-Sanchez, Jose Esteban MuÃ±oz-Medina, Jose de Jesus NuÃ±ez-Contreras, Alicia OcaÃ±a-Mondragon, Luis Alberto Ochoa-Carrera, Hector Esteban Paz-Juarez, Marissa Perez-Garcia, Francisco Pulido, Helen Haydee Fernanda Ramirez-Plascencia, Jorge Salas-Hernandez, Angel Gustavo Salas-Lais, Alejandro Sanchez-Flores, Clara Esperanza Santacruz-Tinoco, Maria Guadalupe Santiago-Mauricio, Selene Zarate, Nelly Selem-Mojica, Blanca Taboada, Gloria Vazquez, Joel Armando Vazquez-Perez. |
| EPI_ISL_2942486 | hCoV-19/Mexico/NLE-LANGEBIO_IMSS_1355/2021 | North America/Mexico/Nuevo Leon | 2021-06-17 | Centro de Investigacion Biomedica del Noreste (CIBIN) | Unidad de Genomica Avanzada | Consorcio Mexicano de Vigilancia Genomica (CoViGen-Mex). Authors (in alphabetical order): Julio Elias Alvarado-Yaah, Carlos F. Arias, Santiago avila-Rios, Eduardo Becerril-Vargas, Victor Hugo Borja-Aburto, Celia Boukadida, Cristobal Chaidez-Quiroz, Juan Bautista Chale-Dzul, Ricardo Ciria Merce, Andreu Comas-Garcia, Celida Duque Molina, Julissa Enciso-Ibarra, Jose Antonio Enciso-Moreno, Gloria Elena Espinosa-Ayala, Fernando Fontove-Herrera, Daniel Fregoso-Rueda, Victor Eduardo Garcia-Arias, Alejandra Garcia-Gasca, Bruno Gomez-Gil, Jean Pierre Gonzalez, Irvin Gonzalez-Lopez, Concepcion Grajales-MuÃ±iz, Ricardo Grande, Rosa Maria Gutierrez Rios, Jesus Hernandez, Alejandra Hernandez-Teran, Alfredo Herrera-Estrella, Carla Ivon Herrera-Najera, Pavel Isa, Veronica Mata-Haro, Daniel Lira Morales, Susana Lopez, Antonio Loza Roman, Brenda Irasema Maldonado-Meza, Bernardo Martinez-Miguel, Jose Arturo Martinez-Orozco, Celida Martinez- Rodriguez, Margarita Matias-Florentino, Fidencio Mejia-Nepomuceno, Maria Guadalupe de Jesus Mireles-Rivera, Gloria Maria Molina-Salinas, Hector Montoya-Fuentes, , Mario Mujica-Sanchez, Jose Esteban MuÃ±oz-Medina, Jose de Jesus NuÃ±ez-Contreras, Alicia OcaÃ±a-Mondragon, Luis Alberto Ochoa-Carrera, Hector Esteban Paz-Juarez, Marissa Perez-Garcia, Francisco Pulido, Helen Haydee Fernanda Ramirez-Plascencia, Jorge Salas-Hernandez, Angel Gustavo Salas-Lais, Alejandro Sanchez-Flores, Clara Esperanza Santacruz-Tinoco, Maria Guadalupe Santiago-Mauricio, Selene Zarate, Nelly Selem-Mojica, Blanca Taboada, Gloria Vazquez, Joel Armando Vazquez-Perez. |
| EPI_ISL_1469111 | hCoV-19/Mexico/SLP_UASLP_A020/2021 | North America/Mexico/San Luis Potosi | 2021-01-21 | Centro de Investigacion en Ciencias de la Salud y Biomedicina | CINVESTAV | Sofia Bernal, Andreu Comas |
| EPI_ISL_4404947 | hCoV-19/Argentina/PAIS-A0765/2020 | South America/Argentina/Buenos Aires | 2020-11-05 | Centro de Investigaciones BÃ¡sicas y Aplicadas, UNNOBA | Ãrea de SecuenciaciÃ³n del Laboratorio de VirologÃ­a del Hospital de NiÃ±os Dr. Ricardo Gutierrez on behalf of 'Proyecto Argentino Interinstitucional de genomica de SARS-CoV-2' (PAIS Consortium) | Nabaes Jodar, MS; Goya, S; AcuÃ±a, D; Natale, MI; Lusso, S; Cristina, C; Alaniz, L; Pasquinelli, V; Palumbo, L; Sevic, I; HernÃ¡ndez del Pin, R; Spinelli, F; Demarchi, G; Vitale, D; Icardi, A; Cassarini,C; Rosales, P; Perrone, S; Bonadeo, N; Valla, S; Chimento, A; Moroni, A; Castro, M; FernÃ¡ndez, A; Barbero, A; EspaÃ±ol, L; Morro, L; Menite, N; VillafaÃ±e, G; Romano, L; Gracia Balbi, L; Brandone, A; Bagnis, N; Valinotto, LE; Viegas, M. |
| EPI_ISL_941143 | hCoV-19/Colombia/AMA-GUR-06792/2020 | South America/Colombia/Amazonas | 2020-05-04 | Centro de Investigaciones en MicrobiologÃ­a y BiotecnologÃ­a-UR (CIMBIUR), Facultad de Ciencias Naturales, Universidad del Rosario, BogotÃ¡, Colombia | Centro de Investigaciones en MicrobiologÃ­a y BiotecnologÃ­a-UR (CIMBIUR), Facultad de Ciencias Naturales, Universidad del Rosario, BogotÃ¡, Colombia |  |
| EPI_ISL_941976 | hCoV-19/Colombia/GUR-04814/2020 | South America/Colombia/Choco | 2020-04-26 | Centro de Investigaciones en MicrobiologÃ­a y BiotecnologÃ­a-UR (CIMBIUR), Facultad de Ciencias Naturales, Universidad del Rosario, BogotÃ¡, Colombia | Centro de Investigaciones en MicrobiologÃ­a y BiotecnologÃ­a-UR (CIMBIUR), Facultad de Ciencias Naturales, Universidad del Rosario, BogotÃ¡, Colombia |  |
| EPI_ISL_941950 | hCoV-19/Colombia/GUR-04816/2020 | South America/Colombia/Choco | 2020-04-26 | Centro de Investigaciones en MicrobiologÃ­a y BiotecnologÃ­a-UR (CIMBIUR), Facultad de Ciencias Naturales, Universidad del Rosario, BogotÃ¡, Colombia | Centro de Investigaciones en MicrobiologÃ­a y BiotecnologÃ­a-UR (CIMBIUR), Facultad de Ciencias Naturales, Universidad del Rosario, BogotÃ¡, Colombia |  |
| EPI_ISL_941977 | hCoV-19/Colombia/GUR-05010/2020 | South America/Colombia/San Andres y Providencia | 2020-04-27 | Centro de Investigaciones en MicrobiologÃ­a y BiotecnologÃ­a-UR (CIMBIUR), Facultad de Ciencias Naturales, Universidad del Rosario, BogotÃ¡, Colombia | Centro de Investigaciones en MicrobiologÃ­a y BiotecnologÃ­a-UR (CIMBIUR), Facultad de Ciencias Naturales, Universidad del Rosario, BogotÃ¡, Colombia |  |
| EPI_ISL_942000 | hCoV-19/Colombia/GUR-06494/2020 | South America/Colombia/Magdalena | 2020-05-03 | Centro de Investigaciones en MicrobiologÃ­a y BiotecnologÃ­a-UR (CIMBIUR), Facultad de Ciencias Naturales, Universidad del Rosario, BogotÃ¡, Colombia | Centro de Investigaciones en MicrobiologÃ­a y BiotecnologÃ­a-UR (CIMBIUR), Facultad de Ciencias Naturales, Universidad del Rosario, BogotÃ¡, Colombia |  |
| EPI_ISL_2491693 | hCoV-19/Brazil/BA-FIOCRUZ-21776/2020 | South America/Brazil/Bahia | 2020-07-05 | Centro de Pesquisa GonÃ§alo Moniz (CPqGM - FIOCRUZ/BA) | Laboratory of Respiratory Viruses and Measles, Oswaldo Cruz Institute, FIOCRUZ | Paola Resende, Luciana Appolinario, Fernando Motta, Anna Carolina Paixao, Ana Carolina Mendonca, Alice Sampaio Rocha, Taina Venas, Elisa Cavalcante Pereira, Renata Serrano Lopes, Ricardo Khouri, Camila I. de Oliveira, Marilda Siqueira on behalf of the Fiocruz COVID-19 Genomic Surveillance Network |
| EPI_ISL_3277552 | hCoV-19/Mexico/CMX-INMEGEN-19-12/2021 | North America/Mexico/Mexico City | 2021-07-23 | Centro Medico ABC | Instituto Nacional de Medicina Genomica | Cedro-Tanda A, Escobar-Arrazola MA, Ramirez-Vega O, Roxana Trejo-GonzÃ¡lez, Cesar Lara, Jacomine Reyes-Carrasco, Daniel Aguirre M, Rangel-DeLeon D, Munguia-Garza P, Hidalgo-Miranda A, Mendoza-Vargas A, Reyes-Grajeda JP, Herrera-Montalvo LA. |
| EPI_ISL_837554 | hCoV-19/Bolivia/26937/2020 | South America/Bolivia/Santa Cruz | 2020-08-03 | Centro Nacional de Enfermedades Tropicales (CENETROP) | Laboratory of Respiratory Viruses and Measles, Oswaldo Cruz Institute, FIOCRUZ | Paola Resende, Roxana Loayza, Cinthia Avila, Luciana Appolinario, Fernando Motta, Anna Carolina Paixao, Ana Carolina Mendonca, Marilda Siqueira on behalf of the Fiocruz COVID-19 Genomic Surveillance Network |
| EPI_ISL_837559 | hCoV-19/Bolivia/26938/2020 | South America/Bolivia/Santa Cruz | 2020-08-11 | Centro Nacional de Enfermedades Tropicales (CENETROP) | Laboratory of Respiratory Viruses and Measles, Oswaldo Cruz Institute, FIOCRUZ | Paola Resende, Roxana Loayza, Cinthia Avila, Luciana Appolinario, Fernando Motta, Anna Carolina Paixao, Ana Carolina Mendonca, Marilda Siqueira on behalf of the Fiocruz COVID-19 Genomic Surveillance Network |
| EPI_ISL_837560 | hCoV-19/Bolivia/26939/2020 | South America/Bolivia/Pando | 2020-07-03 | Centro Nacional de Enfermedades Tropicales (CENETROP) | Laboratory of Respiratory Viruses and Measles, Oswaldo Cruz Institute, FIOCRUZ | Paola Resende, Roxana Loayza, Cinthia Avila, Luciana Appolinario, Fernando Motta, Anna Carolina Paixao, Ana Carolina Mendonca, Marilda Siqueira on behalf of the Fiocruz COVID-19 Genomic Surveillance Network |
| EPI_ISL_837563 | hCoV-19/Bolivia/26942/2020 | South America/Bolivia/Santa Cruz | 2020-05-10 | Centro Nacional de Enfermedades Tropicales (CENETROP) | Laboratory of Respiratory Viruses and Measles, Oswaldo Cruz Institute, FIOCRUZ | Paola Resende, Roxana Loayza, Cinthia Avila, Luciana Appolinario, Fernando Motta, Anna Carolina Paixao, Ana Carolina Mendonca, Marilda Siqueira on behalf of the Fiocruz COVID-19 Genomic Surveillance Network |
| EPI_ISL_837573 | hCoV-19/Bolivia/26955/2020 | South America/Bolivia/Tarija | 2020-04-20 | Centro Nacional de Enfermedades Tropicales (CENETROP) | Laboratory of Respiratory Viruses and Measles, Oswaldo Cruz Institute, FIOCRUZ | Paola Resende, Roxana Loayza, Cinthia Avila, Luciana Appolinario, Fernando Motta, Anna Carolina Paixao, Ana Carolina Mendonca, Marilda Siqueira on behalf of the Fiocruz COVID-19 Genomic Surveillance Network |
| EPI_ISL_837578 | hCoV-19/Bolivia/26960/2020 | South America/Bolivia/Santa Cruz | 2020-04-25 | Centro Nacional de Enfermedades Tropicales (CENETROP) | Laboratory of Respiratory Viruses and Measles, Oswaldo Cruz Institute, FIOCRUZ | Paola Resende, Roxana Loayza, Cinthia Avila, Luciana Appolinario, Fernando Motta, Anna Carolina Paixao, Ana Carolina Mendonca, Marilda Siqueira on behalf of the Fiocruz COVID-19 Genomic Surveillance Network |
| EPI_ISL_2274039 | hCoV-19/Bolivia/21742/2021 | South America/Bolivia/ | 2021-03-23 | Centro Nacional de Enfermidades Tropicales (CENETROP) | Laboratory of Respiratory Viruses and Measles, Oswaldo Cruz Institute, FIOCRUZ | Paola Resende, Roxana Loayza, Cinthia Avila, Luciana Appolinario, Fernando Motta, Anna Carolina Paixao, Ana Carolina Mendonca, Alice Sampaio Rocha, Taina Venas, Elisa Cavalcante Pereira, Renata Serrano Lopes, Marilda Siqueira on behalf of the Fiocruz COVID-19 Genomic Surveillance Network |
| EPI_ISL_753785 | hCoV-19/Germany/BE-ChVir-D1936-8209/2020 | Europe/Germany/Berlin | 2020-09-24 | CharitÃ© UniversitÃ¤tsmedizin Berlin, Institut fÃ¼r Virologie/Labor Berlin | CharitÃ© UniversitÃ¤tsmedizin Berlin, Institut fÃ¼r Virologie | Victor M Corman, JÃ¶rn Beheim-Schwarzbach, Barbara MÃ¼hlemann, Julia Schneider, Talitha Veith, Terry Jones, Christian Drosten |
| EPI_ISL_2278336 | hCoV-19/France/OCC-CHU-TLS-210571395301/2021 | Europe/France/Haute-Garonne | 2021-02-26 | CHU Purpan - Laboratoire de Virologie - Institut FÃ©dÃ©ratif de Biologie | CHU Purpan - Laboratoire de Virologie - Institut FÃ©dÃ©ratif de Biologie | Latour J., Milhes M., Bulach T., Ranger N., Salin G., Nicot F., Tremeaux P., Donnadieu C., Izopet J. |
| EPI_ISL_1652173 | hCoV-19/USA/WI-MHDL-0641/2020 | North America/USA/Wisconsin | 2020-12-23 | City of Milwaukee Health Department Laboratory | City of Milwaukee Health Department Laboratory | Sanjib Bhattacharyya |
| EPI_ISL_2150975 | hCoV-19/Colombia/MET-249/2020 | South America/Colombia/Meta | 2020-06-04 | ClÃ­nica Meta | Instituto de VirologÃ­a-Universidad El Bosque | Eliana P. Calvo, L. Johana MadroÃ±ero, Maria Fernanda MontaÃ±ez-Suarez, Adriana Franco, Andres F. Cardona-Rios, Maria Stella LÃ³pez, Jhann AndrÃ©s Arturo, Lilia Bernal-Cepeda, Sonia P. BohÃ³rquez, MarÃ­a AngÃ©lica CalderÃ³n-PelÃ¡ez, Sigrid J. Camacho-Ortega, Carolina Coronel-Ruiz, Felix G. Delgado, Lady Lopez, â€ªMyriam Lucia Velandia-Romero, Laura Silvana PÃ©rez, Karl A Ciuoderis, Jorge E. Osorio, Juan P. Hernandez-Ortiz, Jaime E. Castellanos |
| EPI_ISL_3948487 | hCoV-19/CostaRica/INC-1061/2021 | North America/Costa Rica/ | 2021-08-03 | CLINICA BIBLICA | Inciensa, Instituto Costarricense de InvestigaciÃ³n y EnseÃ±anza en NutriciÃ³n y Salud | Francisco Duarte, Hebleen Porras, Claudio Soto-Garita, Estela Cordero, Adriana GodÃ­nez, Melany CalderÃ³n, JosÃ© Luis Vargas, Mariela GutiÃ©rrez & Joselyn Prado |
| EPI_ISL_3638856 | hCoV-19/CostaRica/INC-0950/2021 | North America/Costa Rica/Cartago | 2021-07-28 | CLINICA CATOLICA | Inciensa, Instituto Costarricense de InvestigaciÃ³n y EnseÃ±anza en NutriciÃ³n y Salud | Francisco Duarte, Hebleen Porras, Claudio Soto-Garita, Estela Cordero, Adriana GodÃ­nez, Melany CalderÃ³n, JosÃ© Luis Vargas, Mariela GutiÃ©rrez & Joselyn Prado |
| EPI_ISL_445297 | hCoV-19/Chile/LI-ISPCH-7/2020 | South America/Chile/O'Higgins | 2020-03-21 | CLINICA INTEGRAL S.A. | Instituto de Salud Publica de Chile | AndrÃ©s E Castillo, BÃ¡rbara Parra,Paz Tapia, Jaime Lagos, Loredana Arata, Alejandra Acevedo, Winston Andrade, Gabriel Leal, Carolina Tambley, Patricia Bustos, Rodrigo Fasce, Jorge Fernandez |
| EPI_ISL_3459408 | hCoV-19/Colombia/MET-INS-VG-5022/2021 | South America/Colombia/Meta | 2021-07-23 | CLINICA PRIMAVERA DE VILLAVICENCIO | Instituto Nacional de Salud | Katherine Laiton-Donato, Diego A. Ãlvarez-DÃ­az, Carlos Franco-MuÃ±oz, Hector Alejandro Ruiz-Moreno, Paola Rojas, Maria T. Herrera-SepÃºlveda, Diego AndrÃ©s Prada, Jhonnatan Reales-GonzÃ¡lez, Sheryll Corchuelo, Julian Naizaque, Jorge Rivera, Gerardo SantamarÃ­a, Sergio Gomez, Lisseth Pardo, Juan Camilo Martinez, Marta Lopez Blanco, Ãngela Alarcon Cruz, Diana Malo, Carmen Osorio, Magdalena Wiesner, Martha Lucia Ospina Martinez, Marcela Mercado-Reyes |
| EPI_ISL_3721575 | hCoV-19/Colombia/ANT-CWOHC-VG-SEC01493K/2021 | South America/Colombia/Antioquia | 2021-02-10 | Clinica SOMER Rionegro | Universidad Nacional de Colombia - Laboratorio GenÃ³mico One Health | Maria Stella LÃ³pez, Andres F. Cardona-Rios, Daniel O. Maldonado-Perez, SimÃ³n Villegas VelÃ¡squez, Lina M. Hurtado, Laura Silvana Perez, Karl A Ciuoderis, Maria AngÃ©lica Maya, Carolina MuÃ±oz-Arango, Celeny Ortiz, Rita Almanza Payares, Idabely Betancur Ortiz, Sandra Ines Cano, Diego A. Ãlvarez-DÃ­az, Carlos Franco-MuÃ±oz, Katherine Laiton-Donato, Hector Alejandro Ruiz-Moreno, Marcela Mercado-Reyes, Jorge E. Osorio, Juan P. Hernandez-Ortiz |
| EPI_ISL_462464 | hCoV-19/BosniaandHerzegovina/ChVir7361/2020 | Europe/Bosnia and Herzegovina/Ilidza | 2020-03-25 | Clinical Center, University of Sarajevo | Charite Universitatsmedizin Berlin, Institute of Virology | Victor M Corman, Jorn Beheim-Schwarzbach, Barbara Muehlemann, Talitha Veith, Julia Schneider, Terry Jones, Amela Dedeic-Ljubovic, Irma Salimovic-Besic, Suzana Arapcic, Almedina Hadzihasanovic-Moro, Selma Mutevelic, Christian Drosten |
| EPI_ISL_2500551 | hCoV-19/USA/NY-CUIMC-NP-3378/2020 | North America/USA/New York | 2020-11-08 | Clinical Microbiology Laboratory, NewYork Presbyterian Hospital/Columbia University Irving Medical Center | Uhlemann Laboratory, Columbia University Irving Medical Center | Medini K. Annavajhala, Anne Kelley, Maya Tagliavia, Anne-Catrin Uhlemann |
| EPI_ISL_2500582 | hCoV-19/USA/NY-CUIMC-NP-3638/2020 | North America/USA/New York | 2020-11-19 | Clinical Microbiology Laboratory, NewYork Presbyterian Hospital/Columbia University Irving Medical Center | Uhlemann Laboratory, Columbia University Irving Medical Center | Medini K. Annavajhala, Anne Kelley, Maya Tagliavia, Anne-Catrin Uhlemann |
| EPI_ISL_1334565 | hCoV-19/USA/NC-UNC-LCCC0397/2020 | North America/USA/North Carolina | 2020-08-14 | Clinical Molecular Microbiology Laboratory, UNC Hospital | Dirk Dittmer | Justin T. Landis, Razia Moorad, Brent A. Eason, Melissa B. Miller, Linda Pluta, Dirk Dittmer, Angelica Juarez, Cameroon Grant, Evelyn Hoffman, Patricio Cano, Jason Wong, Carolina Caro-Vegas, Ryan McNamara, Danielle Chappell, Cecilia Thompson, Shawn Hawken, Jason Smedberg, Andrea McDaniel-Harper, Billy Fisher, Ashwini Roy-Chaudhury, Blossom Damania. |
| EPI_ISL_1192355 | hCoV-19/USA/NC-UNC-0098/2021 | North America/USA/North Carolina | 2021-01-30 | Clinical Molecular Microbiology Laboratory, UNC Hospitals | Jeremy Wang | Jeremy Wang, Alexander Rubinsteyn, Colleen Rice, Jason Smedberg, Shawn Hawken, Melissa Miller, Corbin Jones, Robert Hagan |
| EPI_ISL_2380825 | hCoV-19/USA/NC-UNC-1374/2021 | North America/USA/North Carolina | 2021-05-10 | Clinical Molecular Microbiology Laboratory, UNC Hospitals | Jeremy Wang | Jeremy Wang, Alexander Rubinsteyn, Colleen Rice, Jason Smedberg, Shawn Hawken, Melissa Miller, Corbin Jones, Robert Hagan |
| EPI_ISL_914003 | hCoV-19/USA/TX-CDC-3966078-001/2021 | North America/USA/Texas | 2021-01-18 | Clinical Pathology Lab | Pathogen Discovery, Respiratory Viruses Branch, Division of Viral Diseases, Centers for Disease Control and Prevention | Ying Tao, Yan Li, Jing Zhang, Krista Queen, Anna Uehara, Peter Cook, Clinton R. Paden, Haibin Wang, Suxiang Tong |
| EPI_ISL_778842 | hCoV-19/USA/TX-CDC-3948809-001/2020 | North America/USA/Texas | 2020-12-28 | Clinical Pathology Labs | Centers for Disease Control and Prevention Division of Viral Diseases, Pathogen Discovery | Ying Tao |
| EPI_ISL_2427372 | hCoV-19/Belgium/MBLG750923/2021 | Europe/Belgium/Brussels | 2021-05-22 | Cliniques universitaires Saint-Luc | UCLouvain/IREC/MBLG | Jean Ruelle, OphÃ©lie Simon, Bertrand Bearzatto, Benoit Kabamba Mukadi |
| EPI_ISL_4084986 | hCoV-19/FrenchGuiana/IPG202101176/2021 | South America/French Guiana/Cayenne | 2021-09-07 | CNR Institut Pasteur de la Guyane | Institut Pasteur de la Guyane | Anne Lavergne, Dominique Rousset, Antoine Enfissi, Arielle Salmier |
| EPI_ISL_4325923 | hCoV-19/France/ARA-HCL021159063801/2021 | Europe/France/Auvergne-Rhone-Alpes | 2021-09-06 | CNR Virus des Infections Respiratoires - France SUD | CNR Virus des Infections Respiratoires - France SUD | Antonin Bal, Gregory Destras, Gwendolyne Burfin, Hadrien Regue, Quentin Semanas, Martine Valette, Bruno Lina, Laurence Josset |
| EPI_ISL_751800 | hCoV-19/USA/CO-CDPHE-2100156850/2020 | North America/USA/Colorado | 2020-12-24 | Colorado Department of Public Health and Environment | Colorado Department of Public Health & Environment | Laura Bankers, Molly C. Hetherington-Rauth, Diana Ir, Shannon Ely, Shannon R. Matzinger, Sarah Elizabeth Totten, Emily A. Travanty |
| EPI_ISL_954996 | hCoV-19/USA/CO-CDPHE-2100235277/2021 | North America/USA/Colorado | 2021-01-17 | Colorado Department of Public Health and Environment | Colorado Department of Puplic Health and Environment | Laura Bankers, Molly C. Hetherington-Rauth, Diana Ir, Shannon Ely, Shannon R. Matzinger, Sarah Elizabeth Totten, Emily A. Travanty |
| EPI_ISL_955057 | hCoV-19/USA/CO-CDPHE-2100270506/2021 | North America/USA/Colorado | 2021-01-24 | Colorado Department of Public Health and Environment | Colorado Department of Puplic Health and Environment | Laura Bankers, Molly C. Hetherington-Rauth, Diana Ir, Shannon Ely, Shannon R. Matzinger, Sarah Elizabeth Totten, Emily A. Travanty |
| EPI_ISL_1097522 | hCoV-19/USA/CO-CDPHE-2100348950/2021 | North America/USA/Colorado | 2021-01-29 | Colorado Department of Public Health and Environment | Colorado Department of Puplic Health and Environment | Laura Bankers, Molly C. Hetherington-Rauth, Diana Ir, Shannon Ely, Shannon R. Matzinger, Sarah Elizabeth Totten, Emily A. Travanty |
| EPI_ISL_1097532 | hCoV-19/USA/CO-CDPHE-2100349308/2020 | North America/USA/Colorado | 2020-12-30 | Colorado Department of Public Health and Environment | Colorado Department of Puplic Health and Environment | Laura Bankers, Molly C. Hetherington-Rauth, Diana Ir, Shannon Ely, Shannon R. Matzinger, Sarah Elizabeth Totten, Emily A. Travanty |
| EPI_ISL_1097629 | hCoV-19/USA/CO-CDPHE-2100406691/2021 | North America/USA/Colorado | 2021-02-10 | Colorado Department of Public Health and Environment | Colorado Department of Puplic Health and Environment | Laura Bankers, Molly C. Hetherington-Rauth, Diana Ir, Shannon Ely, Shannon R. Matzinger, Sarah Elizabeth Totten, Emily A. Travanty |
| EPI_ISL_1169645 | hCoV-19/USA/CO-CDPHE-2100438214/2021 | North America/USA/Colorado | 2021-02-16 | Colorado Department of Public Health and Environment | Colorado Department of Public Health and Environment | Laura Bankers, Molly C. Hetherington-Rauth, Diana Ir, Shannon Ely, Shannon R. Matzinger, Sarah Elizabeth Totten, Emily A. Travanty |
| EPI_ISL_1169652 | hCoV-19/USA/CO-CDPHE-2100468241/2021 | North America/USA/Colorado | 2021-02-21 | Colorado Department of Public Health and Environment | Colorado Department of Public Health and Environment | Laura Bankers, Molly C. Hetherington-Rauth, Diana Ir, Shannon Ely, Shannon R. Matzinger, Sarah Elizabeth Totten, Emily A. Travanty |
| EPI_ISL_1169659 | hCoV-19/USA/CO-CDPHE-2100478870/2021 | North America/USA/Colorado | 2021-02-23 | Colorado Department of Public Health and Environment | Colorado Department of Public Health and Environment | Laura Bankers, Molly C. Hetherington-Rauth, Diana Ir, Shannon Ely, Shannon R. Matzinger, Sarah Elizabeth Totten, Emily A. Travanty |
| EPI_ISL_1234108 | hCoV-19/USA/CO-CDPHE-2100498838/2021 | North America/USA/Colorado | 2021-02-24 | Colorado Department of Public Health and Environment | Colorado Department of Public Health and Environment | Laura Bankers, Molly C. Hetherington-Rauth, Diana Ir, Shannon Ely, Shannon R. Matzinger, Sarah Elizabeth Totten, Emily A. Travanty |
| EPI_ISL_1234189 | hCoV-19/USA/CO-CDPHE-2100510232/2021 | North America/USA/Colorado | 2021-02-26 | Colorado Department of Public Health and Environment | Colorado Department of Public Health and Environment | Laura Bankers, Molly C. Hetherington-Rauth, Diana Ir, Shannon Ely, Shannon R. Matzinger, Sarah Elizabeth Totten, Emily A. Travanty |
| EPI_ISL_1234193 | hCoV-19/USA/CO-CDPHE-2100510236/2021 | North America/USA/Colorado | 2021-02-25 | Colorado Department of Public Health and Environment | Colorado Department of Public Health and Environment | Laura Bankers, Molly C. Hetherington-Rauth, Diana Ir, Shannon Ely, Shannon R. Matzinger, Sarah Elizabeth Totten, Emily A. Travanty |
| EPI_ISL_1234282 | hCoV-19/USA/CO-CDPHE-2100529465/2021 | North America/USA/Colorado | 2021-02-25 | Colorado Department of Public Health and Environment | Colorado Department of Public Health and Environment | Laura Bankers, Molly C. Hetherington-Rauth, Diana Ir, Shannon Ely, Shannon R. Matzinger, Sarah Elizabeth Totten, Emily A. Travanty |
| EPI_ISL_2310461 | hCoV-19/USA/CO-CDPHE-2101044866/2021 | North America/USA/Colorado | 2021-05-09 | Colorado Department of Public Health and Environment | Colorado Department of Public Health and Environment | Laura Bankers, Molly C. Hetherington-Rauth, Diana Ir, Alexandria Rossheim, Shannon R. Matzinger, Sarah Elizabeth Totten, Emily A. Travanty |
| EPI_ISL_2550118 | hCoV-19/USA/CO-CDPHE-2101125319/2021 | North America/USA/Colorado | 2021-05-05 | Colorado Department of Public Health and Environment | Colorado Department of Public Health and Environment | Laura Bankers, Molly C. Hetherington-Rauth, Diana Ir, Alexandria Rossheim, Shannon R. Matzinger, Sarah Elizabeth Totten, Emily A. Travanty |
| EPI_ISL_2790668 | hCoV-19/USA/CO-CDPHE-2101246726/2021 | North America/USA/Colorado | 2021-05-27 | Colorado Department of Public Health and Environment | Colorado Department of Public Health and Environment | Laura Bankers, Molly C. Hetherington-Rauth, Diana Ir, Alexandria Rossheim, Michael Martin, Mandy Waters, Shannon R. Matzinger, Sarah Elizabeth Totten, Emily A. Travanty |
| EPI_ISL_794284 | hCoV-19/USA/NY-Wadsworth-21001695-01/2020 | North America/USA/New York | 2020-12-18 | Columbia University Irving Medical Center | Wadsworth Center, New York State Department.of Health | Kirsten St. George, Daryl M. Lamson, Alexis Russel, Matthew Shudt, Melissa A Leisner, Jonathan Plitnick, Navjot Singh, John Kelly, Sara Griesemer, Erasmus Schneider, Erica Lasek-Nesselquist |
| EPI_ISL_794286 | hCoV-19/USA/NY-Wadsworth-21001697-01/2020 | North America/USA/New York | 2020-12-18 | Columbia University Irving Medical Center | Wadsworth Center, New York State Department.of Health | Kirsten St. George, Daryl M. Lamson, Alexis Russel, Matthew Shudt, Melissa A Leisner, Jonathan Plitnick, Navjot Singh, John Kelly, Sara Griesemer, Erasmus Schneider, Erica Lasek-Nesselquist |
| EPI_ISL_804980 | hCoV-19/USA/NY-Wadsworth-21001916-01/2020 | North America/USA/New York | 2020-12-24 | Columbia University Irving Medical Center | Wadsworth Center, New York State Department of Health | Kirsten St. George, Daryl M. Lamson, Alexis Russel, Matthew Shudt, Melissa A Leisner, Jonathan Plitnick, Navjot Singh, John Kelly, Erasmus Schneider, Erica Lasek-Nesselquist |
| EPI_ISL_936043 | hCoV-19/USA/NY-Wadsworth-21007582-01/2020 | North America/USA/New York | 2020-12-28 | Columbia University Irving Medical Center | Wadsworth Center, New York State Department of Health | Kirsten St. George, Daryl M. Lamson, Alexis Russel, Matthew Shudt, Melissa A Leisner, Jonathan Plitnick, Navjot Singh, John Kelly, Erasmus Schneider, Erica Lasek-Nesselquist |
| EPI_ISL_1227930 | hCoV-19/USA/NY-Wadsworth-21015477-01/2021 | North America/USA/New York | 2021-01-21 | Columbia University Irving Medical Center | Wadsworth Center, New York State Department of Health | Kirsten St. George, Daryl M. Lamson, Alexis Russel, Matthew Shudt, Melissa A Leisner, Jonathan Plitnick, Navjot Singh, John Kelly, Erasmus Schneider, Erica Lasek-Nesselquist |
| EPI_ISL_3353677 | hCoV-19/NorthernMarianaIslands/MP-CDC-2-3831009-/2020 | Oceania/Northern Mariana Islands/ | 2020-12-17 | Commonwealth Healthcare Center | Centers for Disease Control and Prevention Division of Viral Diseases, Pathogen Discovery | Mili Sheth,Sarah Nobles,Jasmine Padilla,Alex Burgin,Meghan Bentz,Mark Burroughs,Peter Cook,Clinton Paden,Dhwani Batra,Krista Queen,Kristen Knipe,Dakota Howard,Yvette Unoarumhi,Matthew Schmerer,Ben Rambo-Martin,Kristine Lacek,Sam Shepard,Dave Wentworth,Vivien Dugan,Suxiang Tong,Justin Lee |
| EPI_ISL_738252 | hCoV-19/USA/MA-Yale-446/2020 | North America/USA/Massachusetts | 2020-04-15 | Connecticut Veterans' Affairs Hospital | Grubaugh Lab - Yale School of Public Health | Joseph Fauver, Tara Alpert, Chantal Vogels, Mary Petrone, Isabel Ott, Ellen Foxman, Shaili Gupta, Danielle Plank, Nathan Grubaugh |
| EPI_ISL_1527014 | hCoV-19/CostaRica/HNN-0394/2021 | North America/Costa Rica/Heredia | 2021-02-17 | COOPESIBA BARVA | Inciensa, Instituto Costarricense de InvestigaciÃ³n y EnseÃ±anza en NutriciÃ³n y Salud | PÃ©rez-Corrales C, Barboza-Arguedas E & Kellerman-Montero K |
| EPI_ISL_2658635 | hCoV-19/USA/CA-CZB-33576/2020 | North America/USA/California | 2020-11-20 | County of San Luis Obispo Public Health Laboratory | Chan-Zuckerberg Biohub | CZB Cliahub Consortium |
| EPI_ISL_436672 | hCoV-19/USA/CA-CZB-1031/2020 | North America/USA/California | 2020-03-29 | County of Santa Clara Public Health Department | Chan-Zuckerberg Biohub | CZB Cliahub Consortium |
| EPI_ISL_2227272 | hCoV-19/Iran/GRC-S1213/2021 | Asia/Iran/Yazd | 2021-04-28 | COVID-19 National Reference Laboratory, Pasteur Institute of Iran | Genetics Research Center, University of Social Welfare and Rehabilitation Sciences | Zohreh Fattahi, Marzieh Mohseni, Kimia Kahrizi, Mahsa Tavakoli,Tahmineh Jalali, Mohammad Hassan Pouriayevali, Mostafa Salehi-Vaziri, Hossein Najmabadi. |
| EPI_ISL_4298223 | hCoV-19/Brazil/SP-IB_130671/2021 | South America/Brazil/Sao Paulo | 2021-09-01 | CS III DE PATROCINIO PAULISTA | Instituto Butantan | Dimas Tadeu Covas, Antonio Jorge Martins, Claudia Renata dos Santos Barros, David Schlesinger, Debora Botequio Moretti, Elaine Cristina Marqueze, Elaine Vieira Santos, Evandra Strazza Rodrigues, Heidge Fukumasu, Jayme Augusto de Souza-Neto, JosÃ© Salvatore Leister PatanÃ©, Luiz Alcantara, Luiz Lehmann Coutinho, Maria Carolina Elias, MaurÃ­cio Lacerda Nogueira, Rafael dos Santos Bezerra, Raul Machado Neto, Rejane Maria Tommasini Grotto, Ricardo Haddad, Sandra Coccuzzo Sampaio Vessoni, Simone Kashima, Svetoslav Nanev Slavov, Vincent Louis Viala |
| EPI_ISL_578180 | hCoV-19/India/WB-IICB-012/2020 | Asia/India/West Bengal | 2020-09-10 | CSIR-Indian Institute of Chemical Biology, MEDICA Supercpecialty Hospital Kolkata | CSIR-Indian Institute of Chemical Biology, MEDICA Supercpecialty Hospital Kolkata | Sujay Krishna Maity, Priyanka Mallick, Debaleena Bhowmik, Abhishake Lahiri, Dr. Aviral |
| EPI_ISL_4413370 | hCoV-19/Brazil/DF-NVBS4145GENOV827983657850/2021 | South America/Brazil/Federal District | 2021-08-14 | DASA | DASA | Rodrigo Guarischi, Paulo Pierry, Adriano Bonaldi, Cristina Oliveira, Bianca Cota, Angelica Hristov, Lidia Yamamoto, Rodrigo Salazar, Annelise Lopes, Jose Levi |
| EPI_ISL_2134808 | hCoV-19/USA/DC-DFS-PHL-0517/2021 | North America/USA/District of Columbia | 2021-01-01 | DC Public Health Lab/ Dept. of Forensic Sciences | DC Public Health Lab/ Dept. of Forensic Sciences | Janis Doss, Scott Nguyen, Elizabeth Zelaya, Sarah Scott, Connie Maza, Monica Mann, Brittany Hamilton, David Payne, Jocelyn Hauser |
| EPI_ISL_2176240 | hCoV-19/USA/DC-DFS-PHL-0533/2020 | North America/USA/District of Columbia | 2020-12-01 | DC Public Health Lab/ Dept. of Forensic Sciences | DC Public Health Lab/ Dept. of Forensic Sciences | Janis Doss, Scott Nguyen, Elizabeth Zelaya, Sarah Scott, Connie Maza, Monica Mann, Brittany Hamilton, David Payne, Jocelyn Hauser |
| EPI_ISL_828071 | hCoV-19/Iceland/4379/2020 | Europe/Iceland/Reykjavik | 2020-12-14 | deCODE genetics | deCODE genetics | Daniel F Gudbjartsson; Agnar Helgason; Hakon Jonsson; Olafur T Magnusson; Pall Melsted; Gudmundur L Norddahl; Jona Saemundsdottir; Asgeir Sigurdsson; Patrick Sulem; Arna B Agustsdottir; Hannes Eggertsson; Berglind Eiriksdottir; Run Fridriksdottir; Elisabet E Gardarsdottir; Gudmundur Georgsson; Olafia S Gretarsdottir; Kjartan R Gudmundsson; Thora R Gunnarsdottir; Arnaldur Gylfason; Hilma Holm; Brynjar O Jensson; Aslaug Jonasdottir; Kamilla S Josefsdottir; Thordur Kristjansson; Droplaug N Magnusdottir; Solvi Rognvaldsson; Louise le Roux; Gudrun Sigmundsdottir; Gardar Sveinbjornsson; Kristin E Sveinsdottir; Maney Sveinsdottir; Emil A Thorarensen; Bjarni Thorbjornsson; Gisli Masson; Ingileif Jonsdottir; Alma Moller; Thorolfur Gudnason; Karl G Kristinsson; Unnur Thorsteinsdottir; Kari Stefansson |
| EPI_ISL_828269 | hCoV-19/Iceland/4577/2020 | Europe/Iceland/Reykjavik | 2020-09-21 | deCODE genetics | deCODE genetics | Daniel F Gudbjartsson; Agnar Helgason; Hakon Jonsson; Olafur T Magnusson; Pall Melsted; Gudmundur L Norddahl; Jona Saemundsdottir; Asgeir Sigurdsson; Patrick Sulem; Arna B Agustsdottir; Hannes Eggertsson; Berglind Eiriksdottir; Run Fridriksdottir; Elisabet E Gardarsdottir; Gudmundur Georgsson; Olafia S Gretarsdottir; Kjartan R Gudmundsson; Thora R Gunnarsdottir; Arnaldur Gylfason; Hilma Holm; Brynjar O Jensson; Aslaug Jonasdottir; Kamilla S Josefsdottir; Thordur Kristjansson; Droplaug N Magnusdottir; Solvi Rognvaldsson; Louise le Roux; Gudrun Sigmundsdottir; Gardar Sveinbjornsson; Kristin E Sveinsdottir; Maney Sveinsdottir; Emil A Thorarensen; Bjarni Thorbjornsson; Gisli Masson; Ingileif Jonsdottir; Alma Moller; Thorolfur Gudnason; Karl G Kristinsson; Unnur Thorsteinsdottir; Kari Stefansson |
| EPI_ISL_575335 | hCoV-19/USA/DE-DHSS-F918609/2020 | North America/USA/Delaware | 2020-03-20 | Delaware Public Health Lab | Delaware Public Health Lab | Gregory Hovan |
| EPI_ISL_1340751 | hCoV-19/Paraguay/33610/2020 | South America/Paraguay/ | 2020-11-06 | Departamento de Virologia, Laboratorio Central de Salud PÃºblica, Avenida Venezuela y Teniente Escurra, AsunciÃ³n, Paraguay | Laboratory of Respiratory Viruses and Measles, Oswaldo Cruz Institute, FIOCRUZ | Paola Resende, Cynthia Vazquez, Luciana Appolinario, Fernando Motta, Anna Carolina Paixao, Ana Carolina Mendonca, Alice Sampaio Rocha, Renata Serrano Lopes, Marilda Siqueira on behalf of the Fiocruz COVID-19 Genomic Surveillance Network |
| EPI_ISL_1340750 | hCoV-19/Paraguay/33612-R2/2020 | South America/Paraguay/ | 2020-11-13 | Departamento de Virologia, Laboratorio Central de Salud PÃºblica, Avenida Venezuela y Teniente Escurra, AsunciÃ³n, Paraguay | Laboratory of Respiratory Viruses and Measles, Oswaldo Cruz Institute, FIOCRUZ | Paola Resende, Cynthia Vazquez, Luciana Appolinario, Fernando Motta, Anna Carolina Paixao, Ana Carolina Mendonca, Alice Sampaio Rocha, Renata Serrano Lopes, Marilda Siqueira on behalf of the Fiocruz COVID-19 Genomic Surveillance Network |
| EPI_ISL_1340764 | hCoV-19/Paraguay/33615/2020 | South America/Paraguay/ | 2020-05-05 | Departamento de Virologia, Laboratorio Central de Salud PÃºblica, Avenida Venezuela y Teniente Escurra, AsunciÃ³n, Paraguay | Laboratory of Respiratory Viruses and Measles, Oswaldo Cruz Institute, FIOCRUZ | Paola Resende, Cynthia Vazquez, Luciana Appolinario, Fernando Motta, Anna Carolina Paixao, Ana Carolina Mendonca, Alice Sampaio Rocha, Renata Serrano Lopes, Marilda Siqueira on behalf of the Fiocruz COVID-19 Genomic Surveillance Network |
| EPI_ISL_1340761 | hCoV-19/Paraguay/33618/2020 | South America/Paraguay/ | 2020-06-24 | Departamento de Virologia, Laboratorio Central de Salud PÃºblica, Avenida Venezuela y Teniente Escurra, AsunciÃ³n, Paraguay | Laboratory of Respiratory Viruses and Measles, Oswaldo Cruz Institute, FIOCRUZ | Paola Resende, Cynthia Vazquez, Luciana Appolinario, Fernando Motta, Anna Carolina Paixao, Ana Carolina Mendonca, Alice Sampaio Rocha, Renata Serrano Lopes, Marilda Siqueira on behalf of the Fiocruz COVID-19 Genomic Surveillance Network |
| EPI_ISL_1340756 | hCoV-19/Paraguay/33622/2020 | South America/Paraguay/ | 2020-09-08 | Departamento de Virologia, Laboratorio Central de Salud PÃºblica, Avenida Venezuela y Teniente Escurra, AsunciÃ³n, Paraguay | Laboratory of Respiratory Viruses and Measles, Oswaldo Cruz Institute, FIOCRUZ | Paola Resende, Cynthia Vazquez, Luciana Appolinario, Fernando Motta, Anna Carolina Paixao, Ana Carolina Mendonca, Alice Sampaio Rocha, Renata Serrano Lopes, Marilda Siqueira on behalf of the Fiocruz COVID-19 Genomic Surveillance Network |
| EPI_ISL_1340754 | hCoV-19/Paraguay/33625/2020 | South America/Paraguay/ | 2020-10-06 | Departamento de Virologia, Laboratorio Central de Salud PÃºblica, Avenida Venezuela y Teniente Escurra, AsunciÃ³n, Paraguay | Laboratory of Respiratory Viruses and Measles, Oswaldo Cruz Institute, FIOCRUZ | Paola Resende, Cynthia Vazquez, Luciana Appolinario, Fernando Motta, Anna Carolina Paixao, Ana Carolina Mendonca, Alice Sampaio Rocha, Renata Serrano Lopes, Marilda Siqueira on behalf of the Fiocruz COVID-19 Genomic Surveillance Network |
| EPI_ISL_1914599 | hCoV-19/Georgia/Tb-SNGS052/2021 | Asia/Georgia/Tbilisi | 2021-03-10 | Department for Virology, Molecular Biology and Genome Research, R. G. Lugar Center for Public Health Research, National Center for Disease Control and Public Health (NCDC) of Georgia. | Department for Virology, Molecular Biology and Genome Research, R. G. Lugar Center for Public Health Research, National Center for Disease Control and Public Health (NCDC) of Georgia. | Meri Pantsulaia, Giorgi Tomashvili, Gvantsa Brachveli, Giorgi Gogoladze, Nino Berishvili, Tata Imnadze, Ana Papkiauri, Gvantsa Chanturia, Ann Machablishvili, Nato Kotaria, Nino Chikhovani, Marine Murtskhvaladze, Lela Sabadze, Mari Gavashelidze, Tamar Jashiashvili, Tea Tevdoradze, Ketevan Sidamonidze, Ekaterine Khmaladze, Ekaterine Zhghenti, Roena Sukhiashvili, Mariam Zakalashvili, Lela Urushadze, Magda Dgebuadze, Davit Tsaguria, Ekaterine Zangaladze, Adam Kotorashvili, Maia Alkhazashvili, Irma Burjanadze, Anna Kasradze, Khatuna Zakhashvili, Paata Imnadze, Amiran Gamkrelidze. |
| EPI_ISL_408430 | hCoV-19/France/IDF-0515/2020 | Europe/France/ÃŽle-de-France | 2020-01-29 | Department of Infectious and Tropical Diseases, Bichat Claude Bernard Hospital, Paris | National Reference Center for Viruses of Respiratory Infections, Institut Pasteur, Paris | MÃ©lanie Albert, Marion Barbet, Sylvie Behillil, MÃ©line Bizard, Angela Brisebarre, Flora Donati, Vincent Enouf, Maud Vanpeene, Sylvie van der Werf, Yazdan Yazdanpanah, Xavier Lescure |
| EPI_ISL_1678824 | hCoV-19/Curacao/CW-AUMC-000717/2021 | South America/Curacao/Willemstad | 2021-03-26 | Department of Medical Microbiology & Infection prevention, Amsterdam University Medical Centers location AMC | Department of Medical Microbiology & Infection prevention, Amsterdam University Medical Centers location AMC | Matthijs Welkers, Robin van Houdt, Marcel Jonges, Sebastien Matamoros, Sjoerd Rebers, Fokla Zorgdrager, Janke Schinkel, Menno de Jong |
| EPI_ISL_2932447 | hCoV-19/Canada/un-RIM-1/2020 | North America/Canada/ | 2020-03-26 | Department of Microbiology and immunology, McGill University Health Centre | Department of Microbiology and immunology, McGill University Health Centre | Lupien,A., McIntosh,F., Reiling,S.J., Galvez,J.H., Ragoussis,I. and Behr,M.A. |
| EPI_ISL_419223 | hCoV-19/HongKong/HKPU49-3001/2020 | Asia/Hong Kong/ | 2020-02-11 | Department of Pathology, Princess Margaret Hospital | Department of Health Technology and Informatics, Faculty of Health and Social Science, The Hong Kong Polytechnic University | Kenneth Siu-Sing LEUNG, Timothy Ting-Leung NG, Alan Ka-Lun WU, Miranda Chong-Yee YAU, Hiu-Yin LAO, Ming-Pan CHOI, Kingsley King-Gee TAM, Lam-Kwong LEE, Barry Kin-Chung WONG, Alex Yat-Man HO, Kam-Tong YIP, Kwok-Cheung LUNG, Raymond Wai-To LIU, Eugene Yuk-Keung TSO, Wai-Shing LEUNG, Man-Chun CHAN, Yuk-Yung NG, Kit-Man SIN, Kitty Sau-Chun FUNG, Sandy Ka-Yee CHAU, Wing-Kin TO, Tak-Lun QUE, David Ho-Keung SHUM, Shea Ping YIP, Wing Cheong YAM, Gilman Kit-Hang SIU |
| EPI_ISL_2692908 | hCoV-19/Iran/GRC-9842/2020 | Asia/Iran/Tehran | 2020-07-13 | Department of Pathology, School of Medicine, Imam Hospital Complex, Tehran University of Medical Sciences, Tehran, Iran | Genetics Research Center, University of Social Welfare and Rehabilitation Sciences | Zohreh Fattahi, Marzieh Mohseni, Khadijeh Jalalvand, Azam Ghaziasadi, Seyedeh elham Mortazavi, Ali Jafarpour, Azar Hadadi, Alireza Abdollahi, Saber Soltani, Reza Najafipour, Kimia Kahrizi, Seyed Mohammad Jazayeri, Hossein Najmabadi. |
| EPI_ISL_703451 | hCoV-19/England/CAMC-BC1A7B/2020 | Europe/United Kingdom/England | 2020-11-22 | Department of Pathology, University of Cambridge | COVID-19 Genomics UK (COG-UK) Consortium | Aminu S. Jahun, Yasmin Chaudhry, Grant Hall, Iliana Georgana, Myra Hosmillo, Martin D. Curran, Malte Pinckert, Surendra Parmar, Ian Goodfellow |
| EPI_ISL_654818 | hCoV-19/Ukraine/Chernivtsi_860/2020 | Europe/Ukraine/Chernivtsi | 2020-08-06 | Department of Respiratory and other Viral Infections of L.V.Gromashevsky Institute of Epidemiology & Infectious Diseases NAMS of UkrainÐµ | Department of Respiratory and other Viral Infections of L.V.Gromashevsky Institute of Epidemiology & Infectious Diseases NAMS of UkrainÐµ, JSC "Farmak" | Alla Mironenko, Andriy Goy, Ihor Kravchuk, Ludmyla Bolotova, Larysa Radchenko, Nataliia Teteriuk |
| EPI_ISL_737210 | hCoV-19/Finland/13ES23H9/2020 | Europe/Finland/Uusimaa | 2020-08-13 | Department of Virology and Immunology, University of Helsinki and Helsinki University Hospital, Huslab Finland | Department of Virology, Faculty of Medicine, University of Helsinki, Helsinki, Finland | Teemu Smura, Ravi Kant, Phuoc Truong, Hussein Alburkat, Hannimari Kallio-Kokko, Jenni Virtanen, Maija Suvanto, Sari Hannula, Harri Kangas, Pekka Ellonen, Olli Vapalahti |
| EPI_ISL_481703 | hCoV-19/Finland/5May49S6/2020 | Europe/Finland/Uusimaa | 2020-05-05 | Department of Virology and Immunology, University of Helsinki and Helsinki University Hospital, Huslab Finland | Department of Virology, Faculty of Medicine, University of Helsinki, Helsinki, Finland | Teemu Smura, Hannimari Kallio-Kokko, Jenni Virtanen, Maija Suvanto, Sari Hannula, Harri Kangas, Pekka Ellonen, Olli Vapalahti |
| EPI_ISL_738135 | hCoV-19/Finland/69HEL/2020 | Europe/Finland/Uusimaa | 2020-12-18 | Department of Virology and Immunology, University of Helsinki and Helsinki University Hospital, Huslab Finland | Department of Virology, Faculty of Medicine, University of Helsinki, Helsinki, Finland | Teemu Smura, Olli Vapalahti, Maija Lappalainen, Satu Kurkela |
| EPI_ISL_928641 | hCoV-19/Denmark/DCGC-37500/2021 | Europe/Denmark/Syddanmark | 2021-01-18 | Department of Virus and Microbiological Special Diagnostics, Statens Serum Institut, Copenhagen, Denmark | Aalborg University | Danish Covid-19 Genome Consortium |
| EPI_ISL_973123 | hCoV-19/Denmark/DCGC-41446/2021 | Europe/Denmark/Hovedstaden | 2021-02-01 | Department of Virus and Microbiological Special Diagnostics, Statens Serum Institut, Copenhagen, Denmark | Aalborg University | Danish Covid-19 Genome Consortium |
| EPI_ISL_1863010 | hCoV-19/Denmark/DCGC-54582/2021 | Europe/Denmark/Hovedstaden | 2021-03-08 | Department of Virus and Microbiological Special Diagnostics, Statens Serum Institut, Copenhagen, Denmark | Aalborg University | Danish Covid-19 Genome Consortium |
| EPI_ISL_2024389 | hCoV-19/Denmark/DCGC-88204/2021 | Europe/Denmark/Hovedstaden | 2021-04-26 | Department of Virus and Microbiological Special Diagnostics, Statens Serum Institut, Copenhagen, Denmark | Aalborg University | Danish Covid-19 Genome Consortium |
| EPI_ISL_416142 | hCoV-19/Denmark/SSI-01/2020 | Europe/Denmark/Hovedstaden | 2020-02-26 | Department of Virus and Microbiological Special diagnostics, Statens Serum Institut, Copenhagen, Denmark. | Statens Serum Institute | Morten Rasmussen, Maiken Worsoe Rosenstierne , Anders Fomsgaard |
| EPI_ISL_618183 | hCoV-19/Denmark/DCGC-1780/2020 | Europe/Denmark/Nordjylland | 2020-07-13 | Department of Virus and Microbiological Special Diagnostics, Statens Serum Institut, Denmark | Albertsen lab, Department of Chemistry and Bioscience, Aalborg University, Denmark | Danish Covid-19 Genome Consortia |
| EPI_ISL_437300 | hCoV-19/Austria/Graz-MUG11/2020 | Europe/Austria/Styria | 2020-04-14 | Diagnostic- and Research Institute of Pathology, Medical University of Graz | Diagnostic- and Research Institute of Pathology, Medical University of Graz | Karl Kashofer, Peter Regitnig, Martin Zacharias, Gregor Gorkiewicz |
| EPI_ISL_747355 | hCoV-19/SouthKorea/KDCA0120/2020 | Asia/South Korea/ | 2020-10-01 | Division of Emerging Infectious Diseases, Bureau of Infectious Diseases Diagnosis Control, Korea Disease Control and Prevention Agency | Division of Emerging Infectious Diseases, Bureau of Infectious Diseases Diagnosis Control, Korea Disease Control and Prevention Agency | Ae Kyung Park, Il-Hwan Kim, Heui Man Kim, Jeong-Min Kim, Namjoo Lee, Chaeyoung Lee, Sang Hee Woo, Eun-Jin Kim |
| EPI_ISL_850336 | hCoV-19/SouthKorea/KDCA0654/2020 | Asia/South Korea/ | 2020-08-17 | Division of Emerging Infectious Diseases, Bureau of Infectious Diseases Diagnosis Control, Korea Disease Control and Prevention Agency | Division of Emerging Infectious Diseases, Bureau of Infectious Diseases Diagnosis Control, Korea Disease Control and Prevention Agency | Ae Kyung Park, Il-Hwan Kim, Heui Man Kim, Jeong-Min Kim, Namjoo Lee, Chaeyoung Lee, Sang Hee Woo, Eun-Jin Kim |
| EPI_ISL_4204352 | hCoV-19/SouthKorea/KDCA11192/2021 | Asia/South Korea/ | 2021-09-01 | Division of Emerging Infectious Diseases, Bureau of Infectious Diseases Diagnosis Control, Korea Disease Control and Prevention Agency | Division of Emerging Infectious Diseases, Bureau of Infectious Diseases Diagnosis Control, Korea Disease Control and Prevention Agency | Ae Kyung Park, Il-Hwan Kim, Heui Man Kim, Jeong-Ah Kim, Chae Young Lee, Eun-Jin Kim |
| EPI_ISL_633066 | hCoV-19/USA/NY-NYCPHL-001059/2020 | North America/USA/New York | 2020-10-09 | DOHMH Chelsea | New York City Public Health Laboratory | Jade Wang, et al. |
| EPI_ISL_745421 | hCoV-19/USA/NY-NYCPHL-001493/2020 | North America/USA/New York | 2020-11-30 | DOHMH Chelsea | New York City Public Health Laboratory | Jade Wang, et al. |
| EPI_ISL_632993 | hCoV-19/USA/NY-NYCPHL-000957/2020 | North America/USA/New York | 2020-10-05 | DOHMH Corona | New York City Public Health Laboratory | Jade Wang, et al. |
| EPI_ISL_683953 | hCoV-19/USA/NY-NYCPHL-001272/2020 | North America/USA/New York | 2020-11-09 | DOHMH Corona | New York City Public Health Laboratory | Jade Wang, et al. |
| EPI_ISL_857101 | hCoV-19/USA/NY-NYCPHL-002243/2021 | North America/USA/New York | 2021-01-06 | DOHMH Corona | New York City Public Health Laboratory | Jade Wang, et al. |
| EPI_ISL_1064784 | hCoV-19/USA/NY-NYCPHL-003318/2021 | North America/USA/New York | 2021-02-16 | DOHMH Corona | New York City Public Health Laboratory | Jade Wang, et al. |
| EPI_ISL_3693013 | hCoV-19/USA/NY-NYCPHL-005513/2021 | North America/USA/New York | 2021-08-11 | DOHMH Corona | New York City Public Health Laboratory | Jade Wang, et al. |
| EPI_ISL_683939 | hCoV-19/USA/NY-NYCPHL-001231/2020 | North America/USA/New York | 2020-11-05 | DOHMH Jamaica | New York City Public Health Laboratory | Jade Wang, et al. |
| EPI_ISL_857242 | hCoV-19/USA/NY-NYCPHL-002025/2020 | North America/USA/New York | 2020-12-30 | DOHMH Jamaica | New York City Public Health Laboratory | Jade Wang, et al. |
| EPI_ISL_633080 | hCoV-19/USA/NY-NYCPHL-001079/2020 | North America/USA/New York | 2020-10-16 | DOHMH Morrisania | New York City Public Health Laboratory | Jade Wang, et al. |
| EPI_ISL_745443 | hCoV-19/USA/NY-NYCPHL-001207/2020 | North America/USA/New York | 2020-11-02 | DOHMH Morrisania | New York City Public Health Laboratory | Jade Wang, et al. |
| EPI_ISL_857122 | hCoV-19/USA/NY-NYCPHL-002286/2021 | North America/USA/New York | 2021-01-07 | DOHMH Morrisania | New York City Public Health Laboratory | Jade Wang, et al. |
| EPI_ISL_633079 | hCoV-19/USA/NY-NYCPHL-001078/2020 | North America/USA/New York | 2020-10-16 | DOHMH PHL | New York City Public Health Laboratory | Jade Wang, et al. |
| EPI_ISL_683995 | hCoV-19/USA/NY-NYCPHL-001395/2020 | North America/USA/New York | 2020-11-17 | DOHMH PHL | New York City Public Health Laboratory | Jade Wang, et al. |
| EPI_ISL_683894 | hCoV-19/USA/NY-NYCPHL-001254/2020 | North America/USA/New York | 2020-11-06 | DOHMH Riverside | New York City Public Health Laboratory | Jade Wang, et al. |
| EPI_ISL_683950 | hCoV-19/USA/NY-NYCPHL-001255/2020 | North America/USA/New York | 2020-11-06 | DOHMH Riverside | New York City Public Health Laboratory | Jade Wang, et al. |
| EPI_ISL_683767 | hCoV-19/USA/NY-NYCPHL-001458/2020 | North America/USA/New York | 2020-11-25 | DOHMH Riverside | New York City Public Health Laboratory | Jade Wang, et al. |
| EPI_ISL_683768 | hCoV-19/USA/NY-NYCPHL-001474/2020 | North America/USA/New York | 2020-11-27 | DOHMH Riverside | New York City Public Health Laboratory | Jade Wang, et al. |
| EPI_ISL_683769 | hCoV-19/USA/NY-NYCPHL-001475/2020 | North America/USA/New York | 2020-11-27 | DOHMH Riverside | New York City Public Health Laboratory | Jade Wang, et al. |
| EPI_ISL_3692600 | hCoV-19/USA/DE-B1099524/2021 | North America/USA/Delaware | 2021-06-10 | DPHL | Delaware Public Health Lab | Rebecca Savage |
| EPI_ISL_1339977 | hCoV-19/USA/DE-DHSS-B1069857/2020 | North America/USA/Delaware | 2020-12-31 | DPHL | Delaware Public Health Lab | Rebecca Savage |
| EPI_ISL_1055402 | hCoV-19/Canada/NL-NML-2412/2020 | North America/Canada/Newfoundland and Labrador | 2020-10-09 | Dr. Leonard A. Miller Centre for Health Services | National Microbiology Laboratory (NML) | Anna Majer, Shari Tyson, Grace Seo, Philip Mabon, Elsie Grudeski, Rhiannon Huzarewich, Russell Mandes, Anneliese Landgraff, Jennifer Tanner, Natalie Knox, Morag Graham, Gary Van Domselaar, Robert Needle, Yang Yu, Adel Malek, Laura Gilbert, George Zahariadis, Nathalie Bastien, Yan Li, Timothy Booth, Darian Hole, Madison Chapel, Kirsten Biggar, Kerri Smith, CanCOGeN's metadata curation team, Public Health Agency of Canada CanCOGeN team |
| EPI_ISL_1055404 | hCoV-19/Canada/NL-NML-2415/2020 | North America/Canada/Newfoundland and Labrador | 2020-09-26 | Dr. Leonard A. Miller Centre for Health Services | National Microbiology Laboratory (NML) | Anna Majer, Shari Tyson, Grace Seo, Philip Mabon, Elsie Grudeski, Rhiannon Huzarewich, Russell Mandes, Anneliese Landgraff, Jennifer Tanner, Natalie Knox, Morag Graham, Gary Van Domselaar, Robert Needle, Yang Yu, Adel Malek, Laura Gilbert, George Zahariadis, Nathalie Bastien, Yan Li, Timothy Booth, Darian Hole, Madison Chapel, Kirsten Biggar, Kerri Smith, CanCOGeN's metadata curation team, Public Health Agency of Canada CanCOGeN team |
| EPI_ISL_3578161 | hCoV-19/Liechtenstein/FL-Risch-2161901046/2021 | Europe/Liechtenstein/ | 2021-06-19 | Dr. Risch Ostschweiz AG | Microbiology, Dr. Risch | Nadia Wohlwend, Faina Wehrli, Dominique Fabien Hilti, Sinem Kas, Martin Risch, Thomas Bodmer, Lorenz Risch |
| EPI_ISL_636518 | hCoV-19/Aruba/AW-RIVM-10392/2020 | South America/Aruba/ | 2020-08-25 | Dutch COVID-19 response team | National Institute for Public Health and the Environment (RIVM) | Adam Meijer, Harry Vennema, Jeroen Cremer, Sharon van den Brink, Bas van der Veer, AnneMarie van den Brandt, Florian Zwagemaker, Dennis Schmitz, Chantal Reusken, on behalf of the national COVID-19 response team |
| EPI_ISL_1059905 | hCoV-19/Aruba/AW-RIVM-10750/2021 | South America/Aruba/Aruba | 2021-01-20 | Dutch COVID-19 response team | National Institute for Public Health and the Environment (RIVM) | Adam Meijer, Harry Vennema, Dirk Eggink, Jeroen Cremer, Sharon van den Brink, Bas van der Veer, AnneMarie van den Brandt, Florian Zwagemaker, Dennis Schmitz, Chantal Reusken, on behalf of the national COVID-19 response team |
| EPI_ISL_1014282 | hCoV-19/Aruba/AW-RIVM-11381/2021 | South America/Aruba/Aruba | 2021-01-28 | Dutch COVID-19 response team | National Institute for Public Health and the Environment (RIVM) | Adam Meijer, Harry Vennema, Dirk Eggink, Jeroen Cremer, Sharon van den Brink, Bas van der Veer, AnneMarie van den Brandt, Florian Zwagemaker, Dennis Schmitz, Chantal Reusken, on behalf of the national COVID-19 response team |
| EPI_ISL_1014281 | hCoV-19/Aruba/AW-RIVM-11382/2021 | South America/Aruba/Aruba | 2021-01-28 | Dutch COVID-19 response team | National Institute for Public Health and the Environment (RIVM) | Adam Meijer, Harry Vennema, Dirk Eggink, Jeroen Cremer, Sharon van den Brink, Bas van der Veer, AnneMarie van den Brandt, Florian Zwagemaker, Dennis Schmitz, Chantal Reusken, on behalf of the national COVID-19 response team |
| EPI_ISL_1014280 | hCoV-19/Aruba/AW-RIVM-11545/2021 | South America/Aruba/Aruba | 2021-01-28 | Dutch COVID-19 response team | National Institute for Public Health and the Environment (RIVM) | Adam Meijer, Harry Vennema, Dirk Eggink, Jeroen Cremer, Sharon van den Brink, Bas van der Veer, AnneMarie van den Brandt, Florian Zwagemaker, Dennis Schmitz, Chantal Reusken, on behalf of the national COVID-19 response team |
| EPI_ISL_1014279 | hCoV-19/Aruba/AW-RIVM-11555/2021 | South America/Aruba/Aruba | 2021-01-28 | Dutch COVID-19 response team | National Institute for Public Health and the Environment (RIVM) | Adam Meijer, Harry Vennema, Dirk Eggink, Jeroen Cremer, Sharon van den Brink, Bas van der Veer, AnneMarie van den Brandt, Florian Zwagemaker, Dennis Schmitz, Chantal Reusken, on behalf of the national COVID-19 response team |
| EPI_ISL_1232698 | hCoV-19/Aruba/AW-RIVM-16023/2021 | South America/Aruba/Aruba | 2021-02-26 | Dutch COVID-19 response team | National Institute for Public Health and the Environment (RIVM) | Adam Meijer, Harry Vennema, Dirk Eggink, Jeroen Cremer, Sharon van den Brink, Bas van der Veer, AnneMarie van den Brandt, Florian Zwagemaker, Dennis Schmitz, Chantal Reusken, on behalf of the national COVID-19 response team |
| EPI_ISL_1232699 | hCoV-19/Aruba/AW-RIVM-16024/2021 | South America/Aruba/Aruba | 2021-02-27 | Dutch COVID-19 response team | National Institute for Public Health and the Environment (RIVM) | Adam Meijer, Harry Vennema, Dirk Eggink, Jeroen Cremer, Sharon van den Brink, Bas van der Veer, AnneMarie van den Brandt, Florian Zwagemaker, Dennis Schmitz, Chantal Reusken, on behalf of the national COVID-19 response team |
| EPI_ISL_1232709 | hCoV-19/Aruba/AW-RIVM-16034/2021 | South America/Aruba/Aruba | 2021-02-27 | Dutch COVID-19 response team | National Institute for Public Health and the Environment (RIVM) | Adam Meijer, Harry Vennema, Dirk Eggink, Jeroen Cremer, Sharon van den Brink, Bas van der Veer, AnneMarie van den Brandt, Florian Zwagemaker, Dennis Schmitz, Chantal Reusken, on behalf of the national COVID-19 response team |
| EPI_ISL_1232717 | hCoV-19/Aruba/AW-RIVM-16042/2021 | South America/Aruba/Aruba | 2021-02-26 | Dutch COVID-19 response team | National Institute for Public Health and the Environment (RIVM) | Adam Meijer, Harry Vennema, Dirk Eggink, Jeroen Cremer, Sharon van den Brink, Bas van der Veer, AnneMarie van den Brandt, Florian Zwagemaker, Dennis Schmitz, Chantal Reusken, on behalf of the national COVID-19 response team |
| EPI_ISL_1232749 | hCoV-19/Aruba/AW-RIVM-16074/2021 | South America/Aruba/Aruba | 2021-02-26 | Dutch COVID-19 response team | National Institute for Public Health and the Environment (RIVM) | Adam Meijer, Harry Vennema, Dirk Eggink, Jeroen Cremer, Sharon van den Brink, Bas van der Veer, AnneMarie van den Brandt, Florian Zwagemaker, Dennis Schmitz, Chantal Reusken, on behalf of the national COVID-19 response team |
| EPI_ISL_1232750 | hCoV-19/Aruba/AW-RIVM-16075/2021 | South America/Aruba/Aruba | 2021-02-27 | Dutch COVID-19 response team | National Institute for Public Health and the Environment (RIVM) | Adam Meijer, Harry Vennema, Dirk Eggink, Jeroen Cremer, Sharon van den Brink, Bas van der Veer, AnneMarie van den Brandt, Florian Zwagemaker, Dennis Schmitz, Chantal Reusken, on behalf of the national COVID-19 response team |
| EPI_ISL_1289180 | hCoV-19/Aruba/AW-RIVM-16758/2021 | South America/Aruba/Aruba | 2021-03-01 | Dutch COVID-19 response team | National Institute for Public Health and the Environment (RIVM) | Adam Meijer, Harry Vennema, Dirk Eggink, Jeroen Cremer, Sharon van den Brink, Bas van der Veer, AnneMarie van den Brandt, Florian Zwagemaker, Dennis Schmitz, Chantal Reusken, on behalf of the national COVID-19 response team |
| EPI_ISL_1596084 | hCoV-19/Aruba/AW-RIVM-23202/2021 | South America/Aruba/Aruba | 2021-03-30 | Dutch COVID-19 response team | National Institute for Public Health and the Environment (RIVM) | Adam Meijer, Harry Vennema, Dirk Eggink, Jeroen Cremer, Sharon van den Brink, Bas van der Veer, AnneMarie van den Brandt, Lisa Wijsman, Kim Freriks, Ryanne Jaarsma, EunÃ­ce Then, Jolienke Hardeman, Lynn Aarts, Sanne Bos, Melissa van Tuil, Robert Kohl, Linda van de Nes, Sjoerd Kuiling, James Groot, Florian Zwagemaker, Dennis Schmitz, Annelies Kroneman, Karim Hajji, Chantal Reusken, on behalf of the national COVID-19 response team |
| EPI_ISL_1705006 | hCoV-19/Aruba/AW-RIVM-25533/2021 | South America/Aruba/Aruba | 2021-04-05 | Dutch COVID-19 response team | National Institute for Public Health and the Environment (RIVM) | Adam Meijer, Harry Vennema, Dirk Eggink, Jeroen Cremer, Sharon van den Brink, Bas van der Veer, AnneMarie van den Brandt, Lisa Wijsman, Kim Freriks, Ryanne Jaarsma, EunÃ­ce Then, Jolienke Hardeman, Lynn Aarts, Sanne Bos, Melissa van Tuil, Robert Kohl, Linda van de Nes, Sjoerd Kuiling, James Groot, Florian Zwagemaker, Dennis Schmitz, Annelies Kroneman, Karim Hajji, Chantal Reusken, on behalf of the national COVID-19 response team |
| EPI_ISL_2094333 | hCoV-19/Aruba/AW-RIVM-30224/2021 | South America/Aruba/Aruba | 2021-04-25 | Dutch COVID-19 response team | National Institute for Public Health and the Environment (RIVM) | Adam Meijer, Harry Vennema, Dirk Eggink, Jeroen Cremer, Sharon van den Brink, Bas van der Veer, AnneMarie van den Brandt, Lisa Wijsman, Kim Freriks, Ryanne Jaarsma, EunÃ­ce Then, Lynn Aarts, Sanne Bos, Melissa van Tuil, Linda van de Nes, Sjoerd Kuiling, James Groot, Florian Zwagemaker, Dennis Schmitz, Annelies Kroneman, Karim Hajji, Chantal Reusken, on behalf of the national COVID-19 response team |
| EPI_ISL_2405547 | hCoV-19/Aruba/AW-RIVM-35091/2021 | South America/Aruba/Aruba | 2021-05-25 | Dutch COVID-19 response team | National Institute for Public Health and the Environment (RIVM) | Adam Meijer, Harry Vennema, Dirk Eggink, Jeroen Cremer, Sharon van den Brink, Bas van der Veer, AnneMarie van den Brandt, Lisa Wijsman, Kim Freriks, Ryanne Jaarsma, EunÃ­ce Then, Lynn Aarts, Sanne Bos, Melissa van Tuil, Linda van de Nes, Sjoerd Kuiling, James Groot, Florian Zwagemaker, Dennis Schmitz, Annelies Kroneman, Karim Hajji, Chantal Reusken, on behalf of the national COVID-19 response team |
| EPI_ISL_2405557 | hCoV-19/Aruba/AW-RIVM-35101/2021 | South America/Aruba/Aruba | 2021-05-25 | Dutch COVID-19 response team | National Institute for Public Health and the Environment (RIVM) | Adam Meijer, Harry Vennema, Dirk Eggink, Jeroen Cremer, Sharon van den Brink, Bas van der Veer, AnneMarie van den Brandt, Lisa Wijsman, Kim Freriks, Ryanne Jaarsma, EunÃ­ce Then, Lynn Aarts, Sanne Bos, Melissa van Tuil, Linda van de Nes, Sjoerd Kuiling, James Groot, Florian Zwagemaker, Dennis Schmitz, Annelies Kroneman, Karim Hajji, Chantal Reusken, on behalf of the national COVID-19 response team |
| EPI_ISL_2405607 | hCoV-19/Aruba/AW-RIVM-35150/2021 | South America/Aruba/Aruba | 2021-05-25 | Dutch COVID-19 response team | National Institute for Public Health and the Environment (RIVM) | Adam Meijer, Harry Vennema, Dirk Eggink, Jeroen Cremer, Sharon van den Brink, Bas van der Veer, AnneMarie van den Brandt, Lisa Wijsman, Kim Freriks, Ryanne Jaarsma, EunÃ­ce Then, Lynn Aarts, Sanne Bos, Melissa van Tuil, Linda van de Nes, Sjoerd Kuiling, James Groot, Florian Zwagemaker, Dennis Schmitz, Annelies Kroneman, Karim Hajji, Chantal Reusken, on behalf of the national COVID-19 response team |
| EPI_ISL_2610702 | hCoV-19/Aruba/AW-RIVM-39241/2021 | South America/Aruba/Aruba | 2021-06-04 | Dutch COVID-19 response team | National Institute for Public Health and the Environment (RIVM) | Adam Meijer, Harry Vennema, Dirk Eggink, Jeroen Cremer, Sharon van den Brink, Bas van der Veer, AnneMarie van den Brandt, Lisa Wijsman, Kim Freriks, Ryanne Jaarsma, EunÃ­ce Then, Lynn Aarts, Sanne Bos, Melissa van Tuil, Linda van de Nes, Florian Zwagemaker, Dennis Schmitz, Annelies Kroneman, Karim Hajji, Chantal Reusken, on behalf of the national COVID-19 response team |
| EPI_ISL_3731766 | hCoV-19/Aruba/AW-RIVM-50382/2021 | South America/Aruba/Aruba | 2021-07-30 | Dutch COVID-19 response team | National Institute for Public Health and the Environment (RIVM) | Adam Meijer, Harry Vennema, Dirk Eggink, Jeroen Cremer, Sharon van den Brink, Bas van der Veer, AnneMarie van den Brandt, Lisa Wijsman, Kim Freriks, Ryanne Jaarsma, EunÃ­ce Then, Lynn Aarts, Sanne Bos, Stijn van Rossum, Florian Zwagemaker, Dennis Schmitz, Annelies Kroneman, Karim Hajji, Chantal Reusken, on behalf of the national COVID-19 response team |
| EPI_ISL_3732875 | hCoV-19/Aruba/AW-RIVM-51699/2021 | South America/Aruba/Aruba | 2021-08-03 | Dutch COVID-19 response team | National Institute for Public Health and the Environment (RIVM) | Adam Meijer, Harry Vennema, Dirk Eggink, Jeroen Cremer, Sharon van den Brink, Bas van der Veer, AnneMarie van den Brandt, Lisa Wijsman, Kim Freriks, Ryanne Jaarsma, EunÃ­ce Then, Lynn Aarts, Sanne Bos, Stijn van Rossum, Florian Zwagemaker, Dennis Schmitz, Annelies Kroneman, Karim Hajji, Chantal Reusken, on behalf of the national COVID-19 response team |
| EPI_ISL_3733747 | hCoV-19/Aruba/AW-RIVM-52699/2021 | South America/Aruba/Aruba | 2021-08-06 | Dutch COVID-19 response team | National Institute for Public Health and the Environment (RIVM) | Adam Meijer, Harry Vennema, Dirk Eggink, Jeroen Cremer, Sharon van den Brink, Bas van der Veer, AnneMarie van den Brandt, Lisa Wijsman, Kim Freriks, Ryanne Jaarsma, EunÃ­ce Then, Lynn Aarts, Sanne Bos, Stijn van Rossum, Florian Zwagemaker, Dennis Schmitz, Annelies Kroneman, Karim Hajji, Chantal Reusken, on behalf of the national COVID-19 response team |
| EPI_ISL_4076894 | hCoV-19/Aruba/AW-RIVM-55646/2021 | South America/Aruba/Aruba | 2021-08-19 | Dutch COVID-19 response team | National Institute for Public Health and the Environment (RIVM) | Adam Meijer, Harry Vennema, Dirk Eggink, Jeroen Cremer, Sharon van den Brink, Bas van der Veer, AnneMarie van den Brandt, Lisa Wijsman, Kim Freriks, Ryanne Jaarsma, EunÃ­ce Then, Lynn Aarts, Sanne Bos, Stijn van Rossum, Florian Zwagemaker, Dennis Schmitz, Annelies Kroneman, Karim Hajji, Ivo van Walle, Chantal Reusken, on behalf of the national COVID-19 response team |
| EPI_ISL_4401652 | hCoV-19/Aruba/AW-RIVM-56711/2021 | South America/Aruba/Aruba | 2021-08-26 | Dutch COVID-19 response team | National Institute for Public Health and the Environment (RIVM) | Adam Meijer, Harry Vennema, Dirk Eggink, Jeroen Cremer, Sharon van den Brink, Bas van der Veer, AnneMarie van den Brandt, Lisa Wijsman, Kim Freriks, Ryanne Jaarsma, EunÃ­ce Then, Lynn Aarts, Sanne Bos, Stijn van Rossum, Florian Zwagemaker, Dennis Schmitz, Annelies Kroneman, Karim Hajji, Ivo van Walle, Chantal Reusken, on behalf of the national COVID-19 response team |
| EPI_ISL_4401665 | hCoV-19/Aruba/AW-RIVM-56716/2021 | South America/Aruba/Aruba | 2021-08-27 | Dutch COVID-19 response team | National Institute for Public Health and the Environment (RIVM) | Adam Meijer, Harry Vennema, Dirk Eggink, Jeroen Cremer, Sharon van den Brink, Bas van der Veer, AnneMarie van den Brandt, Lisa Wijsman, Kim Freriks, Ryanne Jaarsma, EunÃ­ce Then, Lynn Aarts, Sanne Bos, Stijn van Rossum, Florian Zwagemaker, Dennis Schmitz, Annelies Kroneman, Karim Hajji, Ivo van Walle, Chantal Reusken, on behalf of the national COVID-19 response team |
| EPI_ISL_1521874 | hCoV-19/Bonaire/BQ-RIVM-22621/2021 | South America/Bonaire/Bonaire | 2021-03-19 | Dutch COVID-19 response team | National Institute for Public Health and the Environment (RIVM) | Adam Meijer, Harry Vennema, Dirk Eggink, Jeroen Cremer, Sharon van den Brink, Bas van der Veer, AnneMarie van den Brandt, Lisa Wijsman, Kim Freriks, Ryanne Jaarsma, EunÃ­ce Then, Jolienke Hardeman, Lynn Aarts, Sanne Bos, Melissa van Tuil, Robert Kohl, Linda van de Nes, Sjoerd Kuiling, James Groot, Florian Zwagemaker, Dennis Schmitz, Annelies Kroneman, Karim Hajji, Chantal Reusken, on behalf of the national COVID-19 response team |
| EPI_ISL_1792865 | hCoV-19/Bonaire/BQ-RIVM-27422/2021 | South America/Bonaire/Bonaire | 2021-04-14 | Dutch COVID-19 response team | National Institute for Public Health and the Environment (RIVM) | Adam Meijer, Harry Vennema, Dirk Eggink, Jeroen Cremer, Sharon van den Brink, Bas van der Veer, AnneMarie van den Brandt, Lisa Wijsman, Kim Freriks, Ryanne Jaarsma, EunÃ­ce Then, Lynn Aarts, Sanne Bos, Melissa van Tuil, Robert Kohl, Linda van de Nes, Sjoerd Kuiling, James Groot, Florian Zwagemaker, Dennis Schmitz, Annelies Kroneman, Karim Hajji, Chantal Reusken, on behalf of the national COVID-19 response team |
| EPI_ISL_2405573 | hCoV-19/Bonaire/BQ-RIVM-35117/2021 | South America/Bonaire/Bonaire | 2021-05-15 | Dutch COVID-19 response team | National Institute for Public Health and the Environment (RIVM) | Adam Meijer, Harry Vennema, Dirk Eggink, Jeroen Cremer, Sharon van den Brink, Bas van der Veer, AnneMarie van den Brandt, Lisa Wijsman, Kim Freriks, Ryanne Jaarsma, EunÃ­ce Then, Lynn Aarts, Sanne Bos, Melissa van Tuil, Linda van de Nes, Sjoerd Kuiling, James Groot, Florian Zwagemaker, Dennis Schmitz, Annelies Kroneman, Karim Hajji, Chantal Reusken, on behalf of the national COVID-19 response team |
| EPI_ISL_2405596 | hCoV-19/Bonaire/BQ-RIVM-35139/2021 | South America/Bonaire/Bonaire | 2021-05-07 | Dutch COVID-19 response team | National Institute for Public Health and the Environment (RIVM) | Adam Meijer, Harry Vennema, Dirk Eggink, Jeroen Cremer, Sharon van den Brink, Bas van der Veer, AnneMarie van den Brandt, Lisa Wijsman, Kim Freriks, Ryanne Jaarsma, EunÃ­ce Then, Lynn Aarts, Sanne Bos, Melissa van Tuil, Linda van de Nes, Sjoerd Kuiling, James Groot, Florian Zwagemaker, Dennis Schmitz, Annelies Kroneman, Karim Hajji, Chantal Reusken, on behalf of the national COVID-19 response team |
| EPI_ISL_2610471 | hCoV-19/Bonaire/BQ-RIVM-39010/2021 | South America/Bonaire/Bonaire | 2021-05-27 | Dutch COVID-19 response team | National Institute for Public Health and the Environment (RIVM) | Adam Meijer, Harry Vennema, Dirk Eggink, Jeroen Cremer, Sharon van den Brink, Bas van der Veer, AnneMarie van den Brandt, Lisa Wijsman, Kim Freriks, Ryanne Jaarsma, EunÃ­ce Then, Lynn Aarts, Sanne Bos, Melissa van Tuil, Linda van de Nes, Florian Zwagemaker, Dennis Schmitz, Annelies Kroneman, Karim Hajji, Chantal Reusken, on behalf of the national COVID-19 response team |
| EPI_ISL_2981896 | hCoV-19/Bonaire/BQ-RIVM-42355/2021 | South America/Bonaire/Bonaire | 2021-06-26 | Dutch COVID-19 response team | National Institute for Public Health and the Environment (RIVM) | Adam Meijer, Harry Vennema, Dirk Eggink, Jeroen Cremer, Sharon van den Brink, Bas van der Veer, AnneMarie van den Brandt, Lisa Wijsman, Kim Freriks, Ryanne Jaarsma, EunÃ­ce Then, Lynn Aarts, Sanne Bos, Melissa van Tuil, Florian Zwagemaker, Dennis Schmitz, Annelies Kroneman, Karim Hajji, Chantal Reusken, on behalf of the national COVID-19 response team |
| EPI_ISL_547451 | hCoV-19/Curacao/CW-RIVM-10093/2020 | South America/Curacao/ | 2020-03-10 | Dutch COVID-19 response team | National Institute for Public Health and the Environment (RIVM) | Adam Meijer, Harry Vennema, Jeroen Cremer, Sharon van den Brink, Bas van der Veer, AnneMarie van den Brandt, Florian Zwagemaker, Dennis Schmitz, Chantal Reusken, on behalf of the national COVID-19 response team |
| EPI_ISL_547452 | hCoV-19/Curacao/CW-RIVM-10094/2020 | South America/Curacao/ | 2020-03-10 | Dutch COVID-19 response team | National Institute for Public Health and the Environment (RIVM) | Adam Meijer, Harry Vennema, Jeroen Cremer, Sharon van den Brink, Bas van der Veer, AnneMarie van den Brandt, Florian Zwagemaker, Dennis Schmitz, Chantal Reusken, on behalf of the national COVID-19 response team |
| EPI_ISL_636492 | hCoV-19/Curacao/CW-RIVM-10308/2020 | South America/Curacao/ | 2020-03-10 | Dutch COVID-19 response team | National Institute for Public Health and the Environment (RIVM) | Adam Meijer, Harry Vennema, Jeroen Cremer, Sharon van den Brink, Bas van der Veer, AnneMarie van den Brandt, Florian Zwagemaker, Dennis Schmitz, Chantal Reusken, on behalf of the national COVID-19 response team |
| EPI_ISL_636521 | hCoV-19/Curacao/CW-RIVM-10366/2020 | South America/Curacao/ | 2020-08-11 | Dutch COVID-19 response team | National Institute for Public Health and the Environment (RIVM) | Adam Meijer, Harry Vennema, Jeroen Cremer, Sharon van den Brink, Bas van der Veer, AnneMarie van den Brandt, Florian Zwagemaker, Dennis Schmitz, Chantal Reusken, on behalf of the national COVID-19 response team |
| EPI_ISL_1014269 | hCoV-19/Curacao/CW-RIVM-11648/2021 | South America/Curacao/Curacao | 2021-01-23 | Dutch COVID-19 response team | National Institute for Public Health and the Environment (RIVM) | Adam Meijer, Harry Vennema, Dirk Eggink, Jeroen Cremer, Sharon van den Brink, Bas van der Veer, AnneMarie van den Brandt, Florian Zwagemaker, Dennis Schmitz, Chantal Reusken, on behalf of the national COVID-19 response team |
| EPI_ISL_1035061 | hCoV-19/Curacao/CW-RIVM-13010/2021 | South America/Curacao/Curacao | 2021-01-26 | Dutch COVID-19 response team | National Institute for Public Health and the Environment (RIVM) | Adam Meijer, Harry Vennema, Dirk Eggink, Jeroen Cremer, Sharon van den Brink, Bas van der Veer, AnneMarie van den Brandt, Florian Zwagemaker, Dennis Schmitz, Chantal Reusken, on behalf of the national COVID-19 response team |
| EPI_ISL_1232247 | hCoV-19/Curacao/CW-RIVM-15572/2021 | South America/Curacao/Curacao | 2021-02-19 | Dutch COVID-19 response team | National Institute for Public Health and the Environment (RIVM) | Adam Meijer, Harry Vennema, Dirk Eggink, Jeroen Cremer, Sharon van den Brink, Bas van der Veer, AnneMarie van den Brandt, Florian Zwagemaker, Dennis Schmitz, Chantal Reusken, on behalf of the national COVID-19 response team |
| EPI_ISL_1232258 | hCoV-19/Curacao/CW-RIVM-15583/2021 | South America/Curacao/Curacao | 2021-02-19 | Dutch COVID-19 response team | National Institute for Public Health and the Environment (RIVM) | Adam Meijer, Harry Vennema, Dirk Eggink, Jeroen Cremer, Sharon van den Brink, Bas van der Veer, AnneMarie van den Brandt, Florian Zwagemaker, Dennis Schmitz, Chantal Reusken, on behalf of the national COVID-19 response team |
| EPI_ISL_1232270 | hCoV-19/Curacao/CW-RIVM-15595/2021 | South America/Curacao/Curacao | 2021-02-19 | Dutch COVID-19 response team | National Institute for Public Health and the Environment (RIVM) | Adam Meijer, Harry Vennema, Dirk Eggink, Jeroen Cremer, Sharon van den Brink, Bas van der Veer, AnneMarie van den Brandt, Florian Zwagemaker, Dennis Schmitz, Chantal Reusken, on behalf of the national COVID-19 response team |
| EPI_ISL_1232290 | hCoV-19/Curacao/CW-RIVM-15615/2021 | South America/Curacao/Curacao | 2021-02-16 | Dutch COVID-19 response team | National Institute for Public Health and the Environment (RIVM) | Adam Meijer, Harry Vennema, Dirk Eggink, Jeroen Cremer, Sharon van den Brink, Bas van der Veer, AnneMarie van den Brandt, Florian Zwagemaker, Dennis Schmitz, Chantal Reusken, on behalf of the national COVID-19 response team |
| EPI_ISL_1232303 | hCoV-19/Curacao/CW-RIVM-15628/2021 | South America/Curacao/Curacao | 2021-02-18 | Dutch COVID-19 response team | National Institute for Public Health and the Environment (RIVM) | Adam Meijer, Harry Vennema, Dirk Eggink, Jeroen Cremer, Sharon van den Brink, Bas van der Veer, AnneMarie van den Brandt, Florian Zwagemaker, Dennis Schmitz, Chantal Reusken, on behalf of the national COVID-19 response team |
| EPI_ISL_1232314 | hCoV-19/Curacao/CW-RIVM-15639/2021 | South America/Curacao/Curacao | 2021-02-18 | Dutch COVID-19 response team | National Institute for Public Health and the Environment (RIVM) | Adam Meijer, Harry Vennema, Dirk Eggink, Jeroen Cremer, Sharon van den Brink, Bas van der Veer, AnneMarie van den Brandt, Florian Zwagemaker, Dennis Schmitz, Chantal Reusken, on behalf of the national COVID-19 response team |
| EPI_ISL_1232325 | hCoV-19/Curacao/CW-RIVM-15650/2021 | South America/Curacao/Curacao | 2021-02-17 | Dutch COVID-19 response team | National Institute for Public Health and the Environment (RIVM) | Adam Meijer, Harry Vennema, Dirk Eggink, Jeroen Cremer, Sharon van den Brink, Bas van der Veer, AnneMarie van den Brandt, Florian Zwagemaker, Dennis Schmitz, Chantal Reusken, on behalf of the national COVID-19 response team |
| EPI_ISL_1232326 | hCoV-19/Curacao/CW-RIVM-15651/2021 | South America/Curacao/Curacao | 2021-02-18 | Dutch COVID-19 response team | National Institute for Public Health and the Environment (RIVM) | Adam Meijer, Harry Vennema, Dirk Eggink, Jeroen Cremer, Sharon van den Brink, Bas van der Veer, AnneMarie van den Brandt, Florian Zwagemaker, Dennis Schmitz, Chantal Reusken, on behalf of the national COVID-19 response team |
| EPI_ISL_1232327 | hCoV-19/Curacao/CW-RIVM-15652/2021 | South America/Curacao/Curacao | 2021-02-19 | Dutch COVID-19 response team | National Institute for Public Health and the Environment (RIVM) | Adam Meijer, Harry Vennema, Dirk Eggink, Jeroen Cremer, Sharon van den Brink, Bas van der Veer, AnneMarie van den Brandt, Florian Zwagemaker, Dennis Schmitz, Chantal Reusken, on behalf of the national COVID-19 response team |
| EPI_ISL_1233007 | hCoV-19/Curacao/CW-RIVM-16333/2021 | South America/Curacao/Curacao | 2021-02-24 | Dutch COVID-19 response team | National Institute for Public Health and the Environment (RIVM) | Adam Meijer, Harry Vennema, Dirk Eggink, Jeroen Cremer, Sharon van den Brink, Bas van der Veer, AnneMarie van den Brandt, Florian Zwagemaker, Dennis Schmitz, Chantal Reusken, on behalf of the national COVID-19 response team |
| EPI_ISL_1289189 | hCoV-19/Curacao/CW-RIVM-16506/2021 | South America/Curacao/Curacao | 2021-02-24 | Dutch COVID-19 response team | National Institute for Public Health and the Environment (RIVM) | Adam Meijer, Harry Vennema, Dirk Eggink, Jeroen Cremer, Sharon van den Brink, Bas van der Veer, AnneMarie van den Brandt, Florian Zwagemaker, Dennis Schmitz, Chantal Reusken, on behalf of the national COVID-19 response team |
| EPI_ISL_1288141 | hCoV-19/Curacao/CW-RIVM-16507/2021 | South America/Curacao/Curacao | 2021-02-27 | Dutch COVID-19 response team | National Institute for Public Health and the Environment (RIVM) | Adam Meijer, Harry Vennema, Dirk Eggink, Jeroen Cremer, Sharon van den Brink, Bas van der Veer, AnneMarie van den Brandt, Florian Zwagemaker, Dennis Schmitz, Chantal Reusken, on behalf of the national COVID-19 response team |
| EPI_ISL_1369772 | hCoV-19/Curacao/CW-RIVM-18441/2021 | South America/Curacao/Curacao | 2021-03-08 | Dutch COVID-19 response team | National Institute for Public Health and the Environment (RIVM) | Adam Meijer, Harry Vennema, Dirk Eggink, Jeroen Cremer, Sharon van den Brink, Bas van der Veer, AnneMarie van den Brandt, Florian Zwagemaker, Dennis Schmitz, Chantal Reusken, on behalf of the national COVID-19 response team |
| EPI_ISL_1370658 | hCoV-19/Curacao/CW-RIVM-18449/2021 | South America/Curacao/Curacao | 2021-03-04 | Dutch COVID-19 response team | National Institute for Public Health and the Environment (RIVM) | Adam Meijer, Harry Vennema, Dirk Eggink, Jeroen Cremer, Sharon van den Brink, Bas van der Veer, AnneMarie van den Brandt, Florian Zwagemaker, Dennis Schmitz, Chantal Reusken, on behalf of the national COVID-19 response team |
| EPI_ISL_1792868 | hCoV-19/Curacao/CW-RIVM-26936/2021 | South America/Curacao/Curacao | 2021-04-09 | Dutch COVID-19 response team | National Institute for Public Health and the Environment (RIVM) | Adam Meijer, Harry Vennema, Dirk Eggink, Jeroen Cremer, Sharon van den Brink, Bas van der Veer, AnneMarie van den Brandt, Lisa Wijsman, Kim Freriks, Ryanne Jaarsma, EunÃ­ce Then, Lynn Aarts, Sanne Bos, Melissa van Tuil, Robert Kohl, Linda van de Nes, Sjoerd Kuiling, James Groot, Florian Zwagemaker, Dennis Schmitz, Annelies Kroneman, Karim Hajji, Chantal Reusken, on behalf of the national COVID-19 response team |
| EPI_ISL_2218086 | hCoV-19/Curacao/CW-RIVM-31681/2021 | South America/Curacao/Curacao | 2021-04-28 | Dutch COVID-19 response team | National Institute for Public Health and the Environment (RIVM) | Adam Meijer, Harry Vennema, Dirk Eggink, Jeroen Cremer, Sharon van den Brink, Bas van der Veer, AnneMarie van den Brandt, Lisa Wijsman, Kim Freriks, Ryanne Jaarsma, EunÃ­ce Then, Lynn Aarts, Sanne Bos, Melissa van Tuil, Linda van de Nes, Sjoerd Kuiling, James Groot, Florian Zwagemaker, Dennis Schmitz, Annelies Kroneman, Karim Hajji, Chantal Reusken, on behalf of the national COVID-19 response team |
| EPI_ISL_2219319 | hCoV-19/Curacao/CW-RIVM-31682/2021 | South America/Curacao/Curacao | 2021-05-01 | Dutch COVID-19 response team | National Institute for Public Health and the Environment (RIVM) | Adam Meijer, Harry Vennema, Dirk Eggink, Jeroen Cremer, Sharon van den Brink, Bas van der Veer, AnneMarie van den Brandt, Lisa Wijsman, Kim Freriks, Ryanne Jaarsma, EunÃ­ce Then, Lynn Aarts, Sanne Bos, Melissa van Tuil, Linda van de Nes, Sjoerd Kuiling, James Groot, Florian Zwagemaker, Dennis Schmitz, Annelies Kroneman, Karim Hajji, Chantal Reusken, on behalf of the national COVID-19 response team |
| EPI_ISL_2862955 | hCoV-19/Curacao/CW-RIVM-41646/2021 | South America/Curacao/Curacao | 2021-06-15 | Dutch COVID-19 response team | National Institute for Public Health and the Environment (RIVM) | Adam Meijer, Harry Vennema, Dirk Eggink, Jeroen Cremer, Sharon van den Brink, Bas van der Veer, AnneMarie van den Brandt, Lisa Wijsman, Kim Freriks, Ryanne Jaarsma, EunÃ­ce Then, Lynn Aarts, Sanne Bos, Melissa van Tuil, Florian Zwagemaker, Dennis Schmitz, Annelies Kroneman, Karim Hajji, Chantal Reusken, on behalf of the national COVID-19 response team |
| EPI_ISL_3136570 | hCoV-19/Curacao/CW-RIVM-44498/2021 | South America/Curacao/Curacao | 2021-06-28 | Dutch COVID-19 response team | National Institute for Public Health and the Environment (RIVM) | Adam Meijer, Harry Vennema, Dirk Eggink, Jeroen Cremer, Sharon van den Brink, Bas van der Veer, AnneMarie van den Brandt, Lisa Wijsman, Kim Freriks, Ryanne Jaarsma, EunÃ­ce Then, Lynn Aarts, Sanne Bos, Melissa van Tuil, Florian Zwagemaker, Dennis Schmitz, Annelies Kroneman, Karim Hajji, Chantal Reusken, on behalf of the national COVID-19 response team |
| EPI_ISL_3136614 | hCoV-19/Curacao/CW-RIVM-44542/2021 | South America/Curacao/Curacao | 2021-06-28 | Dutch COVID-19 response team | National Institute for Public Health and the Environment (RIVM) | Adam Meijer, Harry Vennema, Dirk Eggink, Jeroen Cremer, Sharon van den Brink, Bas van der Veer, AnneMarie van den Brandt, Lisa Wijsman, Kim Freriks, Ryanne Jaarsma, EunÃ­ce Then, Lynn Aarts, Sanne Bos, Melissa van Tuil, Florian Zwagemaker, Dennis Schmitz, Annelies Kroneman, Karim Hajji, Chantal Reusken, on behalf of the national COVID-19 response team |
| EPI_ISL_3390712 | hCoV-19/Curacao/CW-RIVM-50223/2021 | South America/Curacao/Curacao | 2021-07-29 | Dutch COVID-19 response team | National Institute for Public Health and the Environment (RIVM) | Adam Meijer, Harry Vennema, Dirk Eggink, Jeroen Cremer, Sharon van den Brink, Bas van der Veer, AnneMarie van den Brandt, Lisa Wijsman, Kim Freriks, Ryanne Jaarsma, EunÃ­ce Then, Lynn Aarts, Sanne Bos, Stijn van Rossum, Florian Zwagemaker, Dennis Schmitz, Annelies Kroneman, Karim Hajji, Chantal Reusken, on behalf of the national COVID-19 response team |
| EPI_ISL_2217376 | hCoV-19/Netherlands/FL-RIVM-32746/2021 | Europe/Netherlands/Flevoland | 2021-04-29 | Dutch COVID-19 response team | National Institute for Public Health and the Environment (RIVM) | Adam Meijer, Harry Vennema, Dirk Eggink, Jeroen Cremer, Sharon van den Brink, Bas van der Veer, AnneMarie van den Brandt, Lisa Wijsman, Kim Freriks, Ryanne Jaarsma, EunÃ­ce Then, Lynn Aarts, Sanne Bos, Melissa van Tuil, Linda van de Nes, Sjoerd Kuiling, James Groot, Florian Zwagemaker, Dennis Schmitz, Annelies Kroneman, Karim Hajji, Chantal Reusken, on behalf of the national COVID-19 response team |
| EPI_ISL_4400481 | hCoV-19/Netherlands/NB-RIVM-58004/2021 | Europe/Netherlands/Noord-Brabant | 2021-09-02 | Dutch COVID-19 response team | National Institute for Public Health and the Environment (RIVM) | Adam Meijer, Harry Vennema, Dirk Eggink, Jeroen Cremer, Sharon van den Brink, Bas van der Veer, AnneMarie van den Brandt, Lisa Wijsman, Kim Freriks, Ryanne Jaarsma, EunÃ­ce Then, Lynn Aarts, Sanne Bos, Stijn van Rossum, Florian Zwagemaker, Dennis Schmitz, Annelies Kroneman, Karim Hajji, Ivo van Walle, Chantal Reusken, on behalf of the national COVID-19 response team |
| EPI_ISL_823990 | hCoV-19/Netherlands/NH-RIVM-21095/2020 | Europe/Netherlands/Noord-Holland | 2020-12-31 | Dutch COVID-19 response team | National Institute for Public Health and the Environment (RIVM) | Adam Meijer, Harry Vennema, Jeroen Cremer, Sharon van den Brink, Bas van der Veer, AnneMarie van den Brandt, Florian Zwagemaker, Dennis Schmitz, Chantal Reusken, on behalf of the national COVID-19 response team |
| EPI_ISL_1595951 | hCoV-19/SintMaarten/SX-RIVM-23069/2021 | North America/Sint Maarten/Sint Maarten | 2021-02-08 | Dutch COVID-19 response team | National Institute for Public Health and the Environment (RIVM) | Adam Meijer, Harry Vennema, Dirk Eggink, Jeroen Cremer, Sharon van den Brink, Bas van der Veer, AnneMarie van den Brandt, Lisa Wijsman, Kim Freriks, Ryanne Jaarsma, EunÃ­ce Then, Jolienke Hardeman, Lynn Aarts, Sanne Bos, Melissa van Tuil, Robert Kohl, Linda van de Nes, Sjoerd Kuiling, James Groot, Florian Zwagemaker, Dennis Schmitz, Annelies Kroneman, Karim Hajji, Chantal Reusken, on behalf of the national COVID-19 response team |
| EPI_ISL_1595971 | hCoV-19/SintMaarten/SX-RIVM-23089/2021 | North America/Sint Maarten/Sint Maarten | 2021-03-19 | Dutch COVID-19 response team | National Institute for Public Health and the Environment (RIVM) | Adam Meijer, Harry Vennema, Dirk Eggink, Jeroen Cremer, Sharon van den Brink, Bas van der Veer, AnneMarie van den Brandt, Lisa Wijsman, Kim Freriks, Ryanne Jaarsma, EunÃ­ce Then, Jolienke Hardeman, Lynn Aarts, Sanne Bos, Melissa van Tuil, Robert Kohl, Linda van de Nes, Sjoerd Kuiling, James Groot, Florian Zwagemaker, Dennis Schmitz, Annelies Kroneman, Karim Hajji, Chantal Reusken, on behalf of the national COVID-19 response team |
| EPI_ISL_1596034 | hCoV-19/SintMaarten/SX-RIVM-23152/2021 | North America/Sint Maarten/Sint Maarten | 2021-03-26 | Dutch COVID-19 response team | National Institute for Public Health and the Environment (RIVM) | Adam Meijer, Harry Vennema, Dirk Eggink, Jeroen Cremer, Sharon van den Brink, Bas van der Veer, AnneMarie van den Brandt, Lisa Wijsman, Kim Freriks, Ryanne Jaarsma, EunÃ­ce Then, Jolienke Hardeman, Lynn Aarts, Sanne Bos, Melissa van Tuil, Robert Kohl, Linda van de Nes, Sjoerd Kuiling, James Groot, Florian Zwagemaker, Dennis Schmitz, Annelies Kroneman, Karim Hajji, Chantal Reusken, on behalf of the national COVID-19 response team |
| EPI_ISL_1705178 | hCoV-19/SintMaarten/SX-RIVM-25705/2021 | North America/Sint Maarten/Sint Maarten | 2021-04-03 | Dutch COVID-19 response team | National Institute for Public Health and the Environment (RIVM) | Adam Meijer, Harry Vennema, Dirk Eggink, Jeroen Cremer, Sharon van den Brink, Bas van der Veer, AnneMarie van den Brandt, Lisa Wijsman, Kim Freriks, Ryanne Jaarsma, EunÃ­ce Then, Jolienke Hardeman, Lynn Aarts, Sanne Bos, Melissa van Tuil, Robert Kohl, Linda van de Nes, Sjoerd Kuiling, James Groot, Florian Zwagemaker, Dennis Schmitz, Annelies Kroneman, Karim Hajji, Chantal Reusken, on behalf of the national COVID-19 response team |
| EPI_ISL_1705945 | hCoV-19/SintMaarten/SX-RIVM-26472/2021 | North America/Sint Maarten/Sint Maarten | 2021-04-09 | Dutch COVID-19 response team | National Institute for Public Health and the Environment (RIVM) | Adam Meijer, Harry Vennema, Dirk Eggink, Jeroen Cremer, Sharon van den Brink, Bas van der Veer, AnneMarie van den Brandt, Lisa Wijsman, Kim Freriks, Ryanne Jaarsma, EunÃ­ce Then, Jolienke Hardeman, Lynn Aarts, Sanne Bos, Melissa van Tuil, Robert Kohl, Linda van de Nes, Sjoerd Kuiling, James Groot, Florian Zwagemaker, Dennis Schmitz, Annelies Kroneman, Karim Hajji, Chantal Reusken, on behalf of the national COVID-19 response team |
| EPI_ISL_2093176 | hCoV-19/SintMaarten/SX-RIVM-29892/2021 | North America/Sint Maarten/Sint Maarten | 2021-04-23 | Dutch COVID-19 response team | National Institute for Public Health and the Environment (RIVM) | Adam Meijer, Harry Vennema, Dirk Eggink, Jeroen Cremer, Sharon van den Brink, Bas van der Veer, AnneMarie van den Brandt, Lisa Wijsman, Kim Freriks, Ryanne Jaarsma, EunÃ­ce Then, Lynn Aarts, Sanne Bos, Melissa van Tuil, Linda van de Nes, Sjoerd Kuiling, James Groot, Florian Zwagemaker, Dennis Schmitz, Annelies Kroneman, Karim Hajji, Chantal Reusken, on behalf of the national COVID-19 response team |
| EPI_ISL_2476337 | hCoV-19/SintMaarten/SX-RIVM-36318/2021 | North America/Sint Maarten/Sint Maarten | 2021-05-22 | Dutch COVID-19 response team | National Institute for Public Health and the Environment (RIVM) | Adam Meijer, Harry Vennema, Dirk Eggink, Jeroen Cremer, Sharon van den Brink, Bas van der Veer, AnneMarie van den Brandt, Lisa Wijsman, Kim Freriks, Ryanne Jaarsma, EunÃ­ce Then, Lynn Aarts, Sanne Bos, Melissa van Tuil, Linda van de Nes, Florian Zwagemaker, Dennis Schmitz, Annelies Kroneman, Karim Hajji, Chantal Reusken, on behalf of the national COVID-19 response team |
| EPI_ISL_2474474 | hCoV-19/SintMaarten/SX-RIVM-36347/2021 | North America/Sint Maarten/Sint Maarten | 2021-05-22 | Dutch COVID-19 response team | National Institute for Public Health and the Environment (RIVM) | Adam Meijer, Harry Vennema, Dirk Eggink, Jeroen Cremer, Sharon van den Brink, Bas van der Veer, AnneMarie van den Brandt, Lisa Wijsman, Kim Freriks, Ryanne Jaarsma, EunÃ­ce Then, Lynn Aarts, Sanne Bos, Melissa van Tuil, Linda van de Nes, Florian Zwagemaker, Dennis Schmitz, Annelies Kroneman, Karim Hajji, Chantal Reusken, on behalf of the national COVID-19 response team |
| EPI_ISL_2476341 | hCoV-19/SintMaarten/SX-RIVM-38068/2021 | North America/Sint Maarten/Sint Maarten | 2021-05-26 | Dutch COVID-19 response team | National Institute for Public Health and the Environment (RIVM) | Adam Meijer, Harry Vennema, Dirk Eggink, Jeroen Cremer, Sharon van den Brink, Bas van der Veer, AnneMarie van den Brandt, Lisa Wijsman, Kim Freriks, Ryanne Jaarsma, EunÃ­ce Then, Lynn Aarts, Sanne Bos, Melissa van Tuil, Linda van de Nes, Florian Zwagemaker, Dennis Schmitz, Annelies Kroneman, Karim Hajji, Chantal Reusken, on behalf of the national COVID-19 response team |
| EPI_ISL_2475186 | hCoV-19/SintMaarten/SX-RIVM-38118/2021 | North America/Sint Maarten/Sint Maarten | 2021-05-24 | Dutch COVID-19 response team | National Institute for Public Health and the Environment (RIVM) | Adam Meijer, Harry Vennema, Dirk Eggink, Jeroen Cremer, Sharon van den Brink, Bas van der Veer, AnneMarie van den Brandt, Lisa Wijsman, Kim Freriks, Ryanne Jaarsma, EunÃ­ce Then, Lynn Aarts, Sanne Bos, Melissa van Tuil, Linda van de Nes, Florian Zwagemaker, Dennis Schmitz, Annelies Kroneman, Karim Hajji, Chantal Reusken, on behalf of the national COVID-19 response team |
| EPI_ISL_2609623 | hCoV-19/SintMaarten/SX-RIVM-38157/2021 | North America/Sint Maarten/Sint Maarten | 2021-05-27 | Dutch COVID-19 response team | National Institute for Public Health and the Environment (RIVM) | Adam Meijer, Harry Vennema, Dirk Eggink, Jeroen Cremer, Sharon van den Brink, Bas van der Veer, AnneMarie van den Brandt, Lisa Wijsman, Kim Freriks, Ryanne Jaarsma, EunÃ­ce Then, Lynn Aarts, Sanne Bos, Melissa van Tuil, Linda van de Nes, Florian Zwagemaker, Dennis Schmitz, Annelies Kroneman, Karim Hajji, Chantal Reusken, on behalf of the national COVID-19 response team |
| EPI_ISL_2673217 | hCoV-19/SintMaarten/SX-RIVM-40492/2021 | North America/Sint Maarten/Sint Maarten | 2021-06-16 | Dutch COVID-19 response team | National Institute for Public Health and the Environment (RIVM) | Adam Meijer, Harry Vennema, Dirk Eggink, Jeroen Cremer, Sharon van den Brink, Bas van der Veer, AnneMarie van den Brandt, Lisa Wijsman, Kim Freriks, Ryanne Jaarsma, EunÃ­ce Then, Lynn Aarts, Sanne Bos, Melissa van Tuil, Linda van de Nes, Florian Zwagemaker, Dennis Schmitz, Annelies Kroneman, Karim Hajji, Chantal Reusken, on behalf of the national COVID-19 response team |
| EPI_ISL_2673228 | hCoV-19/SintMaarten/SX-RIVM-40503/2021 | North America/Sint Maarten/Sint Maarten | 2021-06-16 | Dutch COVID-19 response team | National Institute for Public Health and the Environment (RIVM) | Adam Meijer, Harry Vennema, Dirk Eggink, Jeroen Cremer, Sharon van den Brink, Bas van der Veer, AnneMarie van den Brandt, Lisa Wijsman, Kim Freriks, Ryanne Jaarsma, EunÃ­ce Then, Lynn Aarts, Sanne Bos, Melissa van Tuil, Linda van de Nes, Florian Zwagemaker, Dennis Schmitz, Annelies Kroneman, Karim Hajji, Chantal Reusken, on behalf of the national COVID-19 response team |
| EPI_ISL_2673250 | hCoV-19/SintMaarten/SX-RIVM-40525/2021 | North America/Sint Maarten/Sint Maarten | 2021-06-16 | Dutch COVID-19 response team | National Institute for Public Health and the Environment (RIVM) | Adam Meijer, Harry Vennema, Dirk Eggink, Jeroen Cremer, Sharon van den Brink, Bas van der Veer, AnneMarie van den Brandt, Lisa Wijsman, Kim Freriks, Ryanne Jaarsma, EunÃ­ce Then, Lynn Aarts, Sanne Bos, Melissa van Tuil, Linda van de Nes, Florian Zwagemaker, Dennis Schmitz, Annelies Kroneman, Karim Hajji, Chantal Reusken, on behalf of the national COVID-19 response team |
| EPI_ISL_2673295 | hCoV-19/SintMaarten/SX-RIVM-40569/2021 | North America/Sint Maarten/Sint Maarten | 2021-06-16 | Dutch COVID-19 response team | National Institute for Public Health and the Environment (RIVM) | Adam Meijer, Harry Vennema, Dirk Eggink, Jeroen Cremer, Sharon van den Brink, Bas van der Veer, AnneMarie van den Brandt, Lisa Wijsman, Kim Freriks, Ryanne Jaarsma, EunÃ­ce Then, Lynn Aarts, Sanne Bos, Melissa van Tuil, Linda van de Nes, Florian Zwagemaker, Dennis Schmitz, Annelies Kroneman, Karim Hajji, Chantal Reusken, on behalf of the national COVID-19 response team |
| EPI_ISL_2787577 | hCoV-19/SintMaarten/SX-RIVM-41602/2021 | North America/Sint Maarten/Sint Maarten | 2021-06-24 | Dutch COVID-19 response team | National Institute for Public Health and the Environment (RIVM) | Adam Meijer, Harry Vennema, Dirk Eggink, Jeroen Cremer, Sharon van den Brink, Bas van der Veer, AnneMarie van den Brandt, Lisa Wijsman, Kim Freriks, Ryanne Jaarsma, EunÃ­ce Then, Lynn Aarts, Sanne Bos, Melissa van Tuil, Florian Zwagemaker, Dennis Schmitz, Annelies Kroneman, Karim Hajji, Chantal Reusken, on behalf of the national COVID-19 response team |
| EPI_ISL_3138301 | hCoV-19/SintMaarten/SX-RIVM-46226/2021 | North America/Sint Maarten/Sint Maarten | 2021-07-22 | Dutch COVID-19 response team | National Institute for Public Health and the Environment (RIVM) | Adam Meijer, Harry Vennema, Dirk Eggink, Jeroen Cremer, Sharon van den Brink, Bas van der Veer, AnneMarie van den Brandt, Lisa Wijsman, Kim Freriks, Ryanne Jaarsma, EunÃ­ce Then, Lynn Aarts, Sanne Bos, Melissa van Tuil, Florian Zwagemaker, Dennis Schmitz, Annelies Kroneman, Karim Hajji, Chantal Reusken, on behalf of the national COVID-19 response team |
| EPI_ISL_3390595 | hCoV-19/SintMaarten/SX-RIVM-50082/2021 | North America/Sint Maarten/Sint Maarten | 2021-07-30 | Dutch COVID-19 response team | National Institute for Public Health and the Environment (RIVM) | Adam Meijer, Harry Vennema, Dirk Eggink, Jeroen Cremer, Sharon van den Brink, Bas van der Veer, AnneMarie van den Brandt, Lisa Wijsman, Kim Freriks, Ryanne Jaarsma, EunÃ­ce Then, Lynn Aarts, Sanne Bos, Stijn van Rossum, Florian Zwagemaker, Dennis Schmitz, Annelies Kroneman, Karim Hajji, Chantal Reusken, on behalf of the national COVID-19 response team |
| EPI_ISL_3390615 | hCoV-19/SintMaarten/SX-RIVM-50106/2021 | North America/Sint Maarten/Sint Maarten | 2021-07-23 | Dutch COVID-19 response team | National Institute for Public Health and the Environment (RIVM) | Adam Meijer, Harry Vennema, Dirk Eggink, Jeroen Cremer, Sharon van den Brink, Bas van der Veer, AnneMarie van den Brandt, Lisa Wijsman, Kim Freriks, Ryanne Jaarsma, EunÃ­ce Then, Lynn Aarts, Sanne Bos, Stijn van Rossum, Florian Zwagemaker, Dennis Schmitz, Annelies Kroneman, Karim Hajji, Chantal Reusken, on behalf of the national COVID-19 response team |
| EPI_ISL_3732393 | hCoV-19/SintMaarten/SX-RIVM-51107/2021 | North America/Sint Maarten/Sint Maarten | 2021-07-31 | Dutch COVID-19 response team | National Institute for Public Health and the Environment (RIVM) | Adam Meijer, Harry Vennema, Dirk Eggink, Jeroen Cremer, Sharon van den Brink, Bas van der Veer, AnneMarie van den Brandt, Lisa Wijsman, Kim Freriks, Ryanne Jaarsma, EunÃ­ce Then, Lynn Aarts, Sanne Bos, Stijn van Rossum, Florian Zwagemaker, Dennis Schmitz, Annelies Kroneman, Karim Hajji, Chantal Reusken, on behalf of the national COVID-19 response team |
| EPI_ISL_3732426 | hCoV-19/SintMaarten/SX-RIVM-51144/2021 | North America/Sint Maarten/Sint Maarten | 2021-08-02 | Dutch COVID-19 response team | National Institute for Public Health and the Environment (RIVM) | Adam Meijer, Harry Vennema, Dirk Eggink, Jeroen Cremer, Sharon van den Brink, Bas van der Veer, AnneMarie van den Brandt, Lisa Wijsman, Kim Freriks, Ryanne Jaarsma, EunÃ­ce Then, Lynn Aarts, Sanne Bos, Stijn van Rossum, Florian Zwagemaker, Dennis Schmitz, Annelies Kroneman, Karim Hajji, Chantal Reusken, on behalf of the national COVID-19 response team |
| EPI_ISL_3733845 | hCoV-19/SintMaarten/SX-RIVM-52807/2021 | North America/Sint Maarten/Sint Maarten | 2021-08-18 | Dutch COVID-19 response team | National Institute for Public Health and the Environment (RIVM) | Adam Meijer, Harry Vennema, Dirk Eggink, Jeroen Cremer, Sharon van den Brink, Bas van der Veer, AnneMarie van den Brandt, Lisa Wijsman, Kim Freriks, Ryanne Jaarsma, EunÃ­ce Then, Lynn Aarts, Sanne Bos, Stijn van Rossum, Florian Zwagemaker, Dennis Schmitz, Annelies Kroneman, Karim Hajji, Chantal Reusken, on behalf of the national COVID-19 response team |
| EPI_ISL_3733917 | hCoV-19/SintMaarten/SX-RIVM-52896/2021 | North America/Sint Maarten/Sint Maarten | 2021-08-18 | Dutch COVID-19 response team | National Institute for Public Health and the Environment (RIVM) | Adam Meijer, Harry Vennema, Dirk Eggink, Jeroen Cremer, Sharon van den Brink, Bas van der Veer, AnneMarie van den Brandt, Lisa Wijsman, Kim Freriks, Ryanne Jaarsma, EunÃ­ce Then, Lynn Aarts, Sanne Bos, Stijn van Rossum, Florian Zwagemaker, Dennis Schmitz, Annelies Kroneman, Karim Hajji, Chantal Reusken, on behalf of the national COVID-19 response team |
| EPI_ISL_4076619 | hCoV-19/SintMaarten/SX-RIVM-55398/2021 | North America/Sint Maarten/Sint Maarten | 2021-08-21 | Dutch COVID-19 response team | National Institute for Public Health and the Environment (RIVM) | Adam Meijer, Harry Vennema, Dirk Eggink, Jeroen Cremer, Sharon van den Brink, Bas van der Veer, AnneMarie van den Brandt, Lisa Wijsman, Kim Freriks, Ryanne Jaarsma, EunÃ­ce Then, Lynn Aarts, Sanne Bos, Stijn van Rossum, Florian Zwagemaker, Dennis Schmitz, Annelies Kroneman, Karim Hajji, Ivo van Walle, Chantal Reusken, on behalf of the national COVID-19 response team |
| EPI_ISL_4075679 | hCoV-19/SintMaarten/SX-RIVM-56456/2021 | North America/Sint Maarten/Sint Maarten | 2021-08-30 | Dutch COVID-19 response team | National Institute for Public Health and the Environment (RIVM) | Adam Meijer, Harry Vennema, Dirk Eggink, Jeroen Cremer, Sharon van den Brink, Bas van der Veer, AnneMarie van den Brandt, Lisa Wijsman, Kim Freriks, Ryanne Jaarsma, EunÃ­ce Then, Lynn Aarts, Sanne Bos, Stijn van Rossum, Florian Zwagemaker, Dennis Schmitz, Annelies Kroneman, Karim Hajji, Ivo van Walle, Chantal Reusken, on behalf of the national COVID-19 response team |
| EPI_ISL_1312683 | hCoV-19/Latvia/1172/2020 | Europe/Latvia/ | 2020-11-19 | E. Gulbja laboratorija | Latvian Biomedical Research and Study Centre | Janis Pjalkovskis, Nikita Zrelovs, Monta Ustinova, Ivars Silamikelis, Liga Birzniece, Kaspars Megnis, Una Krumina, Guntars Zarins, Vita Rovite, Lauma Freimane, Laila Silamikele, Laura Ansone, Davids Fridmanis, Mikus Gavars, Dmitrijs Perminovs, Jurijs Perevoscikovs, Uga Dumpis, Janis Klovins |
| EPI_ISL_593517 | hCoV-19/Canada/ON-E39/2020 | North America/Canada/Ontario | 2020-05-26 | Eastern Ontario Regional Laboratory Association | McMaster University | Leanne Mortimer, Hooman Derakhshani, Emily Panousis, Ahmed Draia, Jalees Nasir, Robert Slinger, Andrew G. McArthur |
| EPI_ISL_1238792 | hCoV-19/Mexico/GUA-31_LA_FB_05_0401/2020 | North America/Mexico/Guanajuato | 2020-09-02 | Ecological and Evolutionary Genomics. UGA-CINVESTAV | Unidad Universitaria de SecuenciaciÃ³n Masiva y BioinformÃ¡tica (UUSMB). IBT-UNAM | Angelica Cibrian |
| EPI_ISL_2940844 | hCoV-19/Canada/AB-ABPHL-18644/2020 | North America/Canada/Alberta | 2020-07-20 | Edmonton Provincial Lab | Public Health Agency of Canada (PHAC) National Microbiology Laboratory | Buss, E, Croxen M, Deo A, Dieu P, Gill K, Ferrato C, Khan F, Koleva P, Li V, Lloyd C, Lynch T, Ma R, Murphy S, Pabbaraju K, Shokoples S, Tipples G, Thayer J, Whitehouse M, Wong A, Yu C, Zelyas N |
| EPI_ISL_2940614 | hCoV-19/Canada/AB-ABPHL-19061/2021 | North America/Canada/Alberta | 2021-05-24 | Edmonton Provincial Lab | Public Health Agency of Canada (PHAC) National Microbiology Laboratory | Buss, E, Croxen M, Deo A, Dieu P, Gill K, Ferrato C, Khan F, Koleva P, Li V, Lloyd C, Lynch T, Ma R, Murphy S, Pabbaraju K, Shokoples S, Tipples G, Thayer J, Whitehouse M, Wong A, Yu C, Zelyas N |
| EPI_ISL_3910969 | hCoV-19/Canada/AB-ABPHL-25470/2021 | North America/Canada/Alberta | 2021-07-22 | Edmonton Provincial Lab | Public Health Agency of Canada (PHAC) National Microbiology Laboratory | Buss, E, Croxen M, Deo A, Dieu P, Gill K, Ferrato C, Khan F, Koleva P, Li V, Lloyd C, Lynch T, Ma R, Murphy S, Pabbaraju K, Shokoples S, Tipples G, Thayer J, Whitehouse M, Wong A, Yu C, Zelyas N |
| EPI_ISL_3524446 | hCoV-19/Canada/AB-ABPHL-25943/2021 | North America/Canada/Alberta | 2021-07-27 | Edmonton Provincial Lab | Public Health Agency of Canada (PHAC) National Microbiology Laboratory | Buss, E, Croxen M, Deo A, Dieu P, Gill K, Ferrato C, Khan F, Koleva P, Li V, Lloyd C, Lynch T, Ma R, Murphy S, Pabbaraju K, Shokoples S, Tipples G, Thayer J, Whitehouse M, Wong A, Yu C, Zelyas N |
| EPI_ISL_4054842 | hCoV-19/Canada/AB-ABPHL-29596/2021 | North America/Canada/Alberta | 2021-08-17 | Edmonton Provincial Lab | Public Health Agency of Canada (PHAC) National Microbiology Laboratory | Buss, E, Croxen M, Deo A, Dieu P, Gill K, Ferrato C, Khan F, Koleva P, Li V, Lloyd C, Lynch T, Ma R, Murphy S, Pabbaraju K, Shokoples S, Tipples G, Thayer J, Whitehouse M, Wong A, Yu C, Zelyas N |
| EPI_ISL_3153269 | hCoV-19/Switzerland/VD-CHUV-GEN5723/2021 | Europe/Switzerland/Vaud | 2021-07-20 | EHNV | Laboratory of genomics and metagenomics | Trestan Pillonel, Damien Jacot, SÃ©bastien Aeby, Gilbert Greub, Claire Bertelli |
| EPI_ISL_845772 | hCoV-19/USA/GA-EHC-425K/2021 | North America/USA/Georgia | 2021-01-06 | Emory Molecular Diagnostics Laboratory, Emory Healthcare | Piantadosi Lab, Emory Department of Pathology | Ahmed Babiker, Anne Piantadosi |
| EPI_ISL_1278086 | hCoV-19/USA/GA-EHC-583M/2020 | North America/USA/Georgia | 2020-12-30 | Emory Molecular Diagnostics Laboratory, Emory Healthcare | Piantadosi Lab, Emory Department of Pathology | Ahmed Babiker, Anne Piantadosi |
| EPI_ISL_2321399 | hCoV-19/USA/GA-EHC-628F/2021 | North America/USA/Georgia | 2021-01-18 | Emory University | Centers for Disease Control and Prevention Division of Viral Diseases, Pathogen Discovery | Mili Sheth, Sarah Nobles, Jasmine Padilla, Mark Burroughs, Shoshona Le, Katie Dillon, Peter Cook, Clinton R. Paden, Dhwani Batra, Krista Queen, Kristen Knipe, Dakota Howard, Yvette Unoarumhi, Darlene Wagner, Matthew Schmerer, Ben L. Rambo-Martin, Kristine Lacek, Sam Shepard, Alison Laufer Halpin, Dave Wentworth, Vivien Dugan, Suxiang Tong, Justin Lee |
| EPI_ISL_681322 | hCoV-19/USA/FL-UF-37/2020 | North America/USA/Florida | 2020-06-30 | Environmental and Global Health, University of Florida | Environmental and Global Health, University of Florida | Loeb,J.C., Stephenson,C.J., Merck,L., Morris,J.G. and Lednicky,J.A. |
| EPI_ISL_2836011 | hCoV-19/USA/un-Yale-5643/2020 | North America/USA/ | 2020-01-19 | Epidemiology of Microbial Diseases, Yale School of Public Health | Epidemiology of Microbial Diseases, Yale School of Public Health | Vogels,C.B., Lucas,C., Fauver,J.R., Breban,M.I., Ott,I.M., Alpert,T., Petrone,M.E., Brito,A.F., Watkins,A.E., Kalinich,C.C., Rothman,J.E., Iwasaki,A., Landry,M.L., Grubaugh,N.D. |
| EPI_ISL_1009291 | hCoV-19/USA/NY-UB-00018/2020 | North America/USA/New York | 2020-04-01 | Erie County Public Health (ECPHL) | University at Buffalo Genomics and Bioinformatics Core | Jonathan Bard, Natalie Lamb, Alyssa Pohlman, Brandon Marzullo, Amanda Boccolucci, Norma Nowak, Donald Yergeau, Jennifer Surtees |
| EPI_ISL_1401029 | hCoV-19/USA/NY-UB-00627/2021 | North America/USA/New York | 2021-01-14 | Erie County Public Health (ECPHL) | University at Buffalo Genomics and Bioinformatics Core | Jonathan Bard, Natalie Lamb, Alyssa Pohlman, Brandon Marzullo, Amanda Boccolucci, Norma Nowak, Donald Yergeau, Jennifer Surtees |
| EPI_ISL_1401048 | hCoV-19/USA/NY-UB-00652/2021 | North America/USA/New York | 2021-01-16 | Erie County Public Health (ECPHL) | University at Buffalo Genomics and Bioinformatics Core | Jonathan Bard, Natalie Lamb, Alyssa Pohlman, Brandon Marzullo, Amanda Boccolucci, Norma Nowak, Donald Yergeau, Jennifer Surtees |
| EPI_ISL_1401053 | hCoV-19/USA/NY-UB-00660/2021 | North America/USA/New York | 2021-01-20 | Erie County Public Health (ECPHL) | University at Buffalo Genomics and Bioinformatics Core | Jonathan Bard, Natalie Lamb, Alyssa Pohlman, Brandon Marzullo, Amanda Boccolucci, Norma Nowak, Donald Yergeau, Jennifer Surtees |
| EPI_ISL_1401060 | hCoV-19/USA/NY-UB-00673/2021 | North America/USA/New York | 2021-01-21 | Erie County Public Health (ECPHL) | University at Buffalo Genomics and Bioinformatics Core | Jonathan Bard, Natalie Lamb, Alyssa Pohlman, Brandon Marzullo, Amanda Boccolucci, Norma Nowak, Donald Yergeau, Jennifer Surtees |
| EPI_ISL_1445016 | hCoV-19/USA/NY-UB-00766/2021 | North America/USA/New York | 2021-02-22 | Erie County Public Health (ECPHL) | University at Buffalo Genomics and Bioinformatics Core | Jonathan Bard, Natalie Lamb, Alyssa Pohlman, Brandon Marzullo, Amanda Boccolucci, Norma Nowak, Donald Yergeau, Jennifer Surtees |
| EPI_ISL_4173948 | hCoV-19/USA/NY-UB-ECPHL-01229/2020 | North America/USA/New York | 2020-08-20 | Erie County Public Health (ECPHL) | University at Buffalo Genomics and Bioinformatics Core | Jonathan Bard, Natalie Lamb, Alyssa Pohlman, Brandon Marzullo, Amanda Boccolucci, Norma Nowak, Donald Yergeau, Jennifer Surtees |
| EPI_ISL_883808 | hCoV-19/USA/AL-HGSC-JGBS/2020 | North America/USA/Alabama | 2020-12-04 | Eurofins Diatherix | Hudsonalpha Genome Sequencing Center | Jane Grimwood, Melissa Williams, Lori H. Handley, Joshua Stough, Leslie Malone, Stefan Brzezinski, Ada Stewart, Teresa Jones, Jenell Webber, John Lovell, Jennifer Cart, and Jeremy Schmutz |
| EPI_ISL_3836418 | hCoV-19/USA/AL-HGSC-JMGI/2021 | North America/USA/Alabama | 2021-08-13 | Eurofins Diatherix | Hudsonalpha Genome Sequencing Center | Jane Grimwood, Melissa Williams, Lori H. Handley, Joshua Stough, Leslie Malone, Stefan Brzezinski, Ada Stewart, Teresa Jones, Jenell Webber, John Lovell, Jennifer Cart, and Jeremy Schmutz |
| EPI_ISL_3215800 | hCoV-19/USA/CA-SEARCH-41165/2021 | North America/USA/California | 2021-07-06 | EXCITE Lab | Andersen lab at Scripps Research | Chip Schooley, Natasha Martin, Cheryl Anderson, Angela Scioscia, Smruthi Karthikeyan, Greg Humphrey, Sawyer Farmer, Abigail Schnapper, Helena Tubb, Tommy Valles + SEARCH |
| EPI_ISL_3692449 | hCoV-19/USA/CA-SEARCH-46044/2021 | North America/USA/California | 2021-08-05 | EXCITE Lab | Andersen lab at Scripps Research | Chip Schooley, Natasha Martin, Cheryl Anderson, Angela Scioscia, Smruthi Karthikeyan, Greg Humphrey, Sawyer Farmer, Abigail Schnapper, Helena Tubb, Tommy Valles + SEARCH |
| EPI_ISL_3692199 | hCoV-19/USA/CA-SEARCH-46502/2021 | North America/USA/California | 2021-08-03 | EXCITE Lab | Andersen lab at Scripps Research | David Pride, Sharon Reed, Chip Schooley, Angela Scioscia, Natasha Martin Cheryl Anderson, Sawyer Farmer, Abigail Schnapper, Helena Tubb, Tommy Valles + SEARCH |
| EPI_ISL_3937004 | hCoV-19/USA/CA-SEARCH-47310/2021 | North America/USA/California | 2021-08-11 | EXCITE Lab | Andersen lab at Scripps Research | David Pride, Sharon Reed, Chip Schooley, Angela Scioscia, Natasha Martin Cheryl Anderson, Sawyer Farmer, Abigail Schnapper, Helena Tubb, Tommy Valles + SEARCH |
| EPI_ISL_1030117 | hCoV-19/USA/FL-BPHL-0246/2021 | North America/USA/Florida | 2021-01-11 | Florida Bureau of Public Health Laboratories | Florida Bureau of Public Health Laboratories | Sarah Schmedes, Jason Blanton |
| EPI_ISL_1049928 | hCoV-19/USA/FL-BPHL-0441/2021 | North America/USA/Florida | 2021-01-13 | Florida Bureau of Public Health Laboratories | Florida Bureau of Public Health Laboratories | Sarah Schmedes, Jason Blanton |
| EPI_ISL_508733 | hCoV-19/USA/FL-BPHL-0530/2020 | North America/USA/Florida | 2020-06-19 | Florida Bureau of Public Health Laboratories | Florida Bureau of Public Health Laboratories | Sarah Schmedes, Jason Blanton |
| EPI_ISL_509755 | hCoV-19/USA/FL-BPHL-0697/2020 | North America/USA/Florida | 2020-03-28 | Florida Bureau of Public Health Laboratories | Florida Bureau of Public Health Laboratories | Sarah Schmedes, Jason Blanton |
| EPI_ISL_1191476 | hCoV-19/USA/FL-BPHL-0740/2021 | North America/USA/Florida | 2021-02-05 | Florida Bureau of Public Health Laboratories | Florida Bureau of Public Health Laboratories | Sarah Schmedes, Jason Blanton |
| EPI_ISL_512555 | hCoV-19/USA/FL-BPHL-0749/2020 | North America/USA/Florida | 2020-06-22 | Florida Bureau of Public Health Laboratories | Florida Bureau of Public Health Laboratories | Sarah Schmedes, Jason Blanton |
| EPI_ISL_512560 | hCoV-19/USA/FL-BPHL-0754/2020 | North America/USA/Florida | 2020-06-23 | Florida Bureau of Public Health Laboratories | Florida Bureau of Public Health Laboratories | Sarah Schmedes, Jason Blanton |
| EPI_ISL_512561 | hCoV-19/USA/FL-BPHL-0755/2020 | North America/USA/Florida | 2020-06-23 | Florida Bureau of Public Health Laboratories | Florida Bureau of Public Health Laboratories | Sarah Schmedes, Jason Blanton |
| EPI_ISL_512565 | hCoV-19/USA/FL-BPHL-0759/2020 | North America/USA/Florida | 2020-06-27 | Florida Bureau of Public Health Laboratories | Florida Bureau of Public Health Laboratories | Sarah Schmedes, Jason Blanton |
| EPI_ISL_512566 | hCoV-19/USA/FL-BPHL-0761/2020 | North America/USA/Florida | 2020-06-28 | Florida Bureau of Public Health Laboratories | Florida Bureau of Public Health Laboratories | Sarah Schmedes, Jason Blanton |
| EPI_ISL_512567 | hCoV-19/USA/FL-BPHL-0762/2020 | North America/USA/Florida | 2020-06-28 | Florida Bureau of Public Health Laboratories | Florida Bureau of Public Health Laboratories | Sarah Schmedes, Jason Blanton |
| EPI_ISL_512570 | hCoV-19/USA/FL-BPHL-0765/2020 | North America/USA/Florida | 2020-06-28 | Florida Bureau of Public Health Laboratories | Florida Bureau of Public Health Laboratories | Sarah Schmedes, Jason Blanton |
| EPI_ISL_512571 | hCoV-19/USA/FL-BPHL-0766/2020 | North America/USA/Florida | 2020-06-28 | Florida Bureau of Public Health Laboratories | Florida Bureau of Public Health Laboratories | Sarah Schmedes, Jason Blanton |
| EPI_ISL_512572 | hCoV-19/USA/FL-BPHL-0767/2020 | North America/USA/Florida | 2020-06-29 | Florida Bureau of Public Health Laboratories | Florida Bureau of Public Health Laboratories | Sarah Schmedes, Jason Blanton |
| EPI_ISL_1218654 | hCoV-19/USA/FL-BPHL-0852/2021 | North America/USA/Florida | 2021-02-12 | Florida Bureau of Public Health Laboratories | Florida Bureau of Public Health Laboratories | Sarah Schmedes, Jason Blanton |
| EPI_ISL_514200 | hCoV-19/USA/FL-BPHL-0854/2020 | North America/USA/Florida | 2020-06-29 | Florida Bureau of Public Health Laboratories | Florida Bureau of Public Health Laboratories | Sarah Schmedes, Jason Blanton |
| EPI_ISL_514202 | hCoV-19/USA/FL-BPHL-0856/2020 | North America/USA/Florida | 2020-06-28 | Florida Bureau of Public Health Laboratories | Florida Bureau of Public Health Laboratories | Sarah Schmedes, Jason Blanton |
| EPI_ISL_514203 | hCoV-19/USA/FL-BPHL-0857/2020 | North America/USA/Florida | 2020-06-28 | Florida Bureau of Public Health Laboratories | Florida Bureau of Public Health Laboratories | Sarah Schmedes, Jason Blanton |
| EPI_ISL_514212 | hCoV-19/USA/FL-BPHL-0866/2020 | North America/USA/Florida | 2020-07-02 | Florida Bureau of Public Health Laboratories | Florida Bureau of Public Health Laboratories | Sarah Schmedes, Jason Blanton |
| EPI_ISL_514216 | hCoV-19/USA/FL-BPHL-0870/2020 | North America/USA/Florida | 2020-07-07 | Florida Bureau of Public Health Laboratories | Florida Bureau of Public Health Laboratories | Sarah Schmedes, Jason Blanton |
| EPI_ISL_514219 | hCoV-19/USA/FL-BPHL-0873/2020 | North America/USA/Florida | 2020-07-09 | Florida Bureau of Public Health Laboratories | Florida Bureau of Public Health Laboratories | Sarah Schmedes, Jason Blanton |
| EPI_ISL_514220 | hCoV-19/USA/FL-BPHL-0874/2020 | North America/USA/Florida | 2020-07-08 | Florida Bureau of Public Health Laboratories | Florida Bureau of Public Health Laboratories | Sarah Schmedes, Jason Blanton |
| EPI_ISL_517808 | hCoV-19/USA/FL-BPHL-0918/2020 | North America/USA/Florida | 2020-07-02 | Florida Bureau of Public Health Laboratories | Florida Bureau of Public Health Laboratories | Sarah Schmedes, Jason Blanton |
| EPI_ISL_517840 | hCoV-19/USA/FL-BPHL-0950/2020 | North America/USA/Florida | 2020-07-07 | Florida Bureau of Public Health Laboratories | Florida Bureau of Public Health Laboratories | Sarah Schmedes, Jason Blanton |
| EPI_ISL_517893 | hCoV-19/USA/FL-BPHL-1003/2020 | North America/USA/Florida | 2020-07-13 | Florida Bureau of Public Health Laboratories | Florida Bureau of Public Health Laboratories | Sarah Schmedes, Jason Blanton |
| EPI_ISL_517895 | hCoV-19/USA/FL-BPHL-1005/2020 | North America/USA/Florida | 2020-07-10 | Florida Bureau of Public Health Laboratories | Florida Bureau of Public Health Laboratories | Sarah Schmedes, Jason Blanton |
| EPI_ISL_526587 | hCoV-19/USA/FL-BPHL-1101/2020 | North America/USA/Florida | 2020-08-03 | Florida Bureau of Public Health Laboratories | Florida Bureau of Public Health Laboratories | Sarah Schmedes, Jason Blanton |
| EPI_ISL_526589 | hCoV-19/USA/FL-BPHL-1103/2020 | North America/USA/Florida | 2020-08-03 | Florida Bureau of Public Health Laboratories | Florida Bureau of Public Health Laboratories | Sarah Schmedes, Jason Blanton |
| EPI_ISL_1218928 | hCoV-19/USA/FL-BPHL-1129/2021 | North America/USA/Florida | 2021-02-23 | Florida Bureau of Public Health Laboratories | Florida Bureau of Public Health Laboratories | Sarah Schmedes, Jason Blanton |
| EPI_ISL_1218990 | hCoV-19/USA/FL-BPHL-1192/2021 | North America/USA/Florida | 2021-02-24 | Florida Bureau of Public Health Laboratories | Florida Bureau of Public Health Laboratories | Sarah Schmedes, Jason Blanton |
| EPI_ISL_1240031 | hCoV-19/USA/FL-BPHL-1252/2021 | North America/USA/Florida | 2021-01-28 | Florida Bureau of Public Health Laboratories | Florida Bureau of Public Health Laboratories | Sarah Schmedes, Jason Blanton |
| EPI_ISL_1239944 | hCoV-19/USA/FL-BPHL-1381/2021 | North America/USA/Florida | 2021-02-26 | Florida Bureau of Public Health Laboratories | Florida Bureau of Public Health Laboratories | Sarah Schmedes, Jason Blanton |
| EPI_ISL_549192 | hCoV-19/USA/FL-BPHL-1410/2020 | North America/USA/Florida | 2020-08-05 | Florida Bureau of Public Health Laboratories | Florida Bureau of Public Health Laboratories | Sarah Schmedes, Jason Blanton |
| EPI_ISL_549234 | hCoV-19/USA/FL-BPHL-1452/2020 | North America/USA/Florida | 2020-04-07 | Florida Bureau of Public Health Laboratories | Florida Bureau of Public Health Laboratories | Sarah Schmedes, Jason Blanton |
| EPI_ISL_1674894 | hCoV-19/USA/FL-BPHL-1579/2021 | North America/USA/Florida | 2021-03-09 | Florida Bureau of Public Health Laboratories | Florida Bureau of Public Health Laboratories | Sarah Schmedes, Jason Blanton |
| EPI_ISL_594332 | hCoV-19/USA/FL-BPHL-1643/2020 | North America/USA/Florida | 2020-07-02 | Florida Bureau of Public Health Laboratories | Florida Bureau of Public Health Laboratories | Sarah Schmedes, Jason Blanton |
| EPI_ISL_594350 | hCoV-19/USA/FL-BPHL-1661/2020 | North America/USA/Florida | 2020-07-05 | Florida Bureau of Public Health Laboratories | Florida Bureau of Public Health Laboratories | Sarah Schmedes, Jason Blanton |
| EPI_ISL_594357 | hCoV-19/USA/FL-BPHL-1668/2020 | North America/USA/Florida | 2020-07-06 | Florida Bureau of Public Health Laboratories | Florida Bureau of Public Health Laboratories | Sarah Schmedes, Jason Blanton |
| EPI_ISL_594385 | hCoV-19/USA/FL-BPHL-1696/2020 | North America/USA/Florida | 2020-08-20 | Florida Bureau of Public Health Laboratories | Florida Bureau of Public Health Laboratories | Sarah Schmedes, Jason Blanton |
| EPI_ISL_613879 | hCoV-19/USA/FL-BPHL-1774/2020 | North America/USA/Florida | 2020-07-29 | Florida Bureau of Public Health Laboratories | Florida Bureau of Public Health Laboratories | Sarah Schmedes, Jason Blanton |
| EPI_ISL_613829 | hCoV-19/USA/FL-BPHL-1897/2020 | North America/USA/Florida | 2020-07-14 | Florida Bureau of Public Health Laboratories | Florida Bureau of Public Health Laboratories | Sarah Schmedes, Jason Blanton |
| EPI_ISL_653288 | hCoV-19/USA/FL-BPHL-2135/2020 | North America/USA/Florida | 2020-05-08 | Florida Bureau of Public Health Laboratories | Florida Bureau of Public Health Laboratories | Sarah Schmedes, Jason Blanton |
| EPI_ISL_1651474 | hCoV-19/USA/FL-BPHL-2295/2021 | North America/USA/Florida | 2021-04-04 | Florida Bureau of Public Health Laboratories | Florida Bureau of Public Health Laboratories | Sarah Schmedes, Jason Blanton |
| EPI_ISL_849011 | hCoV-19/USA/FL-BPHL-2298/2020 | North America/USA/Florida | 2020-10-01 | Florida Bureau of Public Health Laboratories | Florida Bureau of Public Health Laboratories | Sarah Schmedes, Jason Blanton |
| EPI_ISL_848772 | hCoV-19/USA/FL-BPHL-2435/2020 | North America/USA/Florida | 2020-11-19 | Florida Bureau of Public Health Laboratories | Florida Bureau of Public Health Laboratories | Sarah Schmedes, Jason Blanton |
| EPI_ISL_849141 | hCoV-19/USA/FL-BPHL-2482/2020 | North America/USA/Florida | 2020-07-07 | Florida Bureau of Public Health Laboratories | Florida Bureau of Public Health Laboratories | Sarah Schmedes, Jason Blanton |
| EPI_ISL_849142 | hCoV-19/USA/FL-BPHL-2484/2020 | North America/USA/Florida | 2020-07-07 | Florida Bureau of Public Health Laboratories | Florida Bureau of Public Health Laboratories | Sarah Schmedes, Jason Blanton |
| EPI_ISL_848830 | hCoV-19/USA/FL-BPHL-2550/2020 | North America/USA/Florida | 2020-11-12 | Florida Bureau of Public Health Laboratories | Florida Bureau of Public Health Laboratories | Sarah Schmedes, Jason Blanton |
| EPI_ISL_848826 | hCoV-19/USA/FL-BPHL-2551/2020 | North America/USA/Florida | 2020-11-12 | Florida Bureau of Public Health Laboratories | Florida Bureau of Public Health Laboratories | Sarah Schmedes, Jason Blanton |
| EPI_ISL_935387 | hCoV-19/USA/FL-BPHL-2866/2020 | North America/USA/Florida | 2020-09-17 | Florida Bureau of Public Health Laboratories | Florida Bureau of Public Health Laboratories | Sarah Schmedes, Jason Blanton |
| EPI_ISL_935453 | hCoV-19/USA/FL-BPHL-2932/2020 | North America/USA/Florida | 2020-11-18 | Florida Bureau of Public Health Laboratories | Florida Bureau of Public Health Laboratories | Sarah Schmedes, Jason Blanton |
| EPI_ISL_2836020 | hCoV-19/USA/FL-BPHL-4182/2021 | North America/USA/Florida | 2021-05-05 | Florida Bureau of Public Health Laboratories | Florida Bureau of Public Health Laboratories | Sarah Schmedes, Jason Blanton |
| EPI_ISL_2836405 | hCoV-19/USA/FL-BPHL-4573/2021 | North America/USA/Florida | 2021-05-25 | Florida Bureau of Public Health Laboratories | Florida Bureau of Public Health Laboratories | Sarah Schmedes, Jason Blanton |
| EPI_ISL_3902942 | hCoV-19/USA/FL-BPHL-5940/2021 | North America/USA/Florida | 2021-08-02 | Florida Bureau of Public Health Laboratories | Florida Bureau of Public Health Laboratories | Sarah Schmedes, Jiaqi Li, Namratha Tarigopula, Jason Blanton |
| EPI_ISL_476139 | hCoV-19/Sweden/20-02114/2020 | Europe/Sweden/Jonkoping | 2020-01-31 | Folkhalsomyndigheten | The Public Health Agency of Sweden | Oskar Karlsson Lindsjo, Maria Lind Karlberg, Mattias Haukland, Reza Advani, Olov Svartstrom, Anna-Malin Linde, Sandra Broddesson, Petra Edquist, Shamam Muradrasoli, Anna Risberg, Karin Tegmark-Wisell |
| EPI_ISL_581492 | hCoV-19/Congo/UKT-014/2020 | Africa/Republic of the Congo/Brazzaville | 2020-07-03 | Fondation Congolaise pour la recherche medicale (FCRM) | NGS Competence Center TÃ¼bingen, Institut fÃ¼r Medizinische Mikrobiologie und Hygiene, UniversitÃ¤tsklinikum TÃ¼bingen | Angel Angelov |
| EPI_ISL_2134207 | hCoV-19/USA/CA-CDC-FG-032262/2021 | North America/USA/California | 2021-05-06 | Fulgent Genetics | Centers for Disease Control and Prevention Division of Viral Diseases, Pathogen Discovery | Dakota Howard, Dhwani Batra, Peter W. Cook, Kara Moser, Adrian Paskey, Jason Caravas, Benjamin Rambo-Martin, Shatavia Morrison, Christopher Gulvick, Scott Sammons, Yvette Unoarumhi, Darlene Wagner, Matthew Schmerer, Harry Gao, Mickey Li, John Gao, Joseph Fierro, Benafsh Sapra, Becky Tsai, Yan Meng, Doreen Ng, James Xie, Clinton R. Paden, Duncan MacCannell |
| EPI_ISL_3658191 | hCoV-19/USA/CA-CDC-FG-074309/2021 | North America/USA/California | 2021-08-10 | Fulgent Genetics | Centers for Disease Control and Prevention Division of Viral Diseases, Pathogen Discovery | Dakota Howard,Dhwani Batra,Peter Cook,Kara Moser,Adrian Paskey,Jason Caravas,Benjamin Rambo-Martin,Shatavia Morrison,Christopher Gulvick,Scott Sammons,Yvette Unoarumhi,Darlene Wagner,Matthew Schmerer,Harry Gao,Mickey Li,John Gao,Joseph Fierro,Benafsh Sapra,Becky Tsai,Yan Meng,Doreen Ng,James Xie,Clinton Paden,Duncan MacCannell |
| EPI_ISL_3658626 | hCoV-19/USA/CA-CDC-FG-075283/2021 | North America/USA/California | 2021-08-11 | Fulgent Genetics | Centers for Disease Control and Prevention Division of Viral Diseases, Pathogen Discovery | Dakota Howard,Dhwani Batra,Peter Cook,Kara Moser,Adrian Paskey,Jason Caravas,Benjamin Rambo-Martin,Shatavia Morrison,Christopher Gulvick,Scott Sammons,Yvette Unoarumhi,Darlene Wagner,Matthew Schmerer,Harry Gao,Mickey Li,John Gao,Joseph Fierro,Benafsh Sapra,Becky Tsai,Yan Meng,Doreen Ng,James Xie,Clinton Paden,Duncan MacCannell |
| EPI_ISL_3754166 | hCoV-19/USA/CA-CDC-FG-085046/2021 | North America/USA/California | 2021-08-17 | Fulgent Genetics | Centers for Disease Control and Prevention Division of Viral Diseases, Pathogen Discovery | Dakota Howard,Dhwani Batra,Peter Cook,Kara Moser,Adrian Paskey,Jason Caravas,Benjamin Rambo-Martin,Shatavia Morrison,Christopher Gulvick,Scott Sammons,Yvette Unoarumhi,Darlene Wagner,Matthew Schmerer,Harry Gao,Mickey Li,John Gao,Joseph Fierro,Benafsh Sapra,Becky Tsai,Yan Meng,Doreen Ng,James Xie,Clinton Paden,Duncan MacCannell |
| EPI_ISL_4243696 | hCoV-19/USA/CA-CDC-FG-112880/2021 | North America/USA/California | 2021-09-03 | Fulgent Genetics | Centers for Disease Control and Prevention Division of Viral Diseases, Pathogen Discovery | Dakota Howard,Dhwani Batra,Peter Cook,Jason Caravas,Benjamin Rambo-Martin,Scott Sammons,Yvette Unoarumhi,Matthew Schmerer,Kristine Lacek,Tymeckia Kendall,Victoria Caban Figueroa,Shatavia Morrison,Christopher Gulvick,Erisa Sula,Harry Gao,Mickey Li,John Gao,Joseph Fierro,Benafsh Sapra,Becky Tsai,Yan Meng,Doreen Ng,James Xie,Clinton Paden,Duncan MacCannell |
| EPI_ISL_3067424 | hCoV-19/USA/CO-FG-028804/2020 | North America/USA/Colorado | 2020-11-25 | Fulgent Genetics | Fulgent Genetics | Harry Gao, Mickey Li, John Gao, Joseph Fierro, Benafsh Sapra, Becky Tsai, Yan Meng, Doreen Ng, James Xie |
| EPI_ISL_1556494 | hCoV-19/USA/FL-CDC-FG-015805/2021 | North America/USA/Florida | 2021-03-22 | Fulgent Genetics | Centers for Disease Control and Prevention Division of Viral Diseases, Pathogen Discovery | Dakota Howard, Dhwani Batra, Peter W. Cook, Kara Moser, Adrian Paskey, Jason Caravas, Benjamin Rambo-Martin, Shatavia Morrison, Christopher Gulvick, Scott Sammons, Yvette Unoarumhi, Darlene Wagner, Matthew Schmerer, Harry Gao, Mickey Li, John Gao, Joseph Fierro, Benafsh Sapra, Becky Tsai, Yan Meng, Doreen Ng, James Xie, Clinton R. Paden, Duncan MacCannell |
| EPI_ISL_1556495 | hCoV-19/USA/FL-CDC-FG-015806/2021 | North America/USA/Florida | 2021-03-22 | Fulgent Genetics | Centers for Disease Control and Prevention Division of Viral Diseases, Pathogen Discovery | Dakota Howard, Dhwani Batra, Peter W. Cook, Kara Moser, Adrian Paskey, Jason Caravas, Benjamin Rambo-Martin, Shatavia Morrison, Christopher Gulvick, Scott Sammons, Yvette Unoarumhi, Darlene Wagner, Matthew Schmerer, Harry Gao, Mickey Li, John Gao, Joseph Fierro, Benafsh Sapra, Becky Tsai, Yan Meng, Doreen Ng, James Xie, Clinton R. Paden, Duncan MacCannell |
| EPI_ISL_1556711 | hCoV-19/USA/FL-CDC-FG-016330/2021 | North America/USA/Florida | 2021-03-25 | Fulgent Genetics | Centers for Disease Control and Prevention Division of Viral Diseases, Pathogen Discovery | Dakota Howard, Dhwani Batra, Peter W. Cook, Kara Moser, Adrian Paskey, Jason Caravas, Benjamin Rambo-Martin, Shatavia Morrison, Christopher Gulvick, Scott Sammons, Yvette Unoarumhi, Darlene Wagner, Matthew Schmerer, Harry Gao, Mickey Li, John Gao, Joseph Fierro, Benafsh Sapra, Becky Tsai, Yan Meng, Doreen Ng, James Xie, Clinton R. Paden, Duncan MacCannell |
| EPI_ISL_1557102 | hCoV-19/USA/FL-CDC-FG-016835/2021 | North America/USA/Florida | 2021-03-20 | Fulgent Genetics | Centers for Disease Control and Prevention Division of Viral Diseases, Pathogen Discovery | Dakota Howard, Dhwani Batra, Peter W. Cook, Kara Moser, Adrian Paskey, Jason Caravas, Benjamin Rambo-Martin, Shatavia Morrison, Christopher Gulvick, Scott Sammons, Yvette Unoarumhi, Darlene Wagner, Matthew Schmerer, Harry Gao, Mickey Li, John Gao, Joseph Fierro, Benafsh Sapra, Becky Tsai, Yan Meng, Doreen Ng, James Xie, Clinton R. Paden, Duncan MacCannell |
| EPI_ISL_1557130 | hCoV-19/USA/FL-CDC-FG-016905/2021 | North America/USA/Florida | 2021-03-23 | Fulgent Genetics | Centers for Disease Control and Prevention Division of Viral Diseases, Pathogen Discovery | Dakota Howard, Dhwani Batra, Peter W. Cook, Kara Moser, Adrian Paskey, Jason Caravas, Benjamin Rambo-Martin, Shatavia Morrison, Christopher Gulvick, Scott Sammons, Yvette Unoarumhi, Darlene Wagner, Matthew Schmerer, Harry Gao, Mickey Li, John Gao, Joseph Fierro, Benafsh Sapra, Becky Tsai, Yan Meng, Doreen Ng, James Xie, Clinton R. Paden, Duncan MacCannell |
| EPI_ISL_1557197 | hCoV-19/USA/FL-CDC-FG-017098/2021 | North America/USA/Florida | 2021-03-25 | Fulgent Genetics | Centers for Disease Control and Prevention Division of Viral Diseases, Pathogen Discovery | Dakota Howard, Dhwani Batra, Peter W. Cook, Kara Moser, Adrian Paskey, Jason Caravas, Benjamin Rambo-Martin, Shatavia Morrison, Christopher Gulvick, Scott Sammons, Yvette Unoarumhi, Darlene Wagner, Matthew Schmerer, Harry Gao, Mickey Li, John Gao, Joseph Fierro, Benafsh Sapra, Becky Tsai, Yan Meng, Doreen Ng, James Xie, Clinton R. Paden, Duncan MacCannell |
| EPI_ISL_1612910 | hCoV-19/USA/FL-CDC-FG-018515/2021 | North America/USA/Florida | 2021-03-26 | Fulgent Genetics | Centers for Disease Control and Prevention Division of Viral Diseases, Pathogen Discovery | Dakota Howard, Dhwani Batra, Peter W. Cook, Kara Moser, Adrian Paskey, Jason Caravas, Benjamin Rambo-Martin, Shatavia Morrison, Christopher Gulvick, Scott Sammons, Yvette Unoarumhi, Darlene Wagner, Matthew Schmerer, Harry Gao, Mickey Li, John Gao, Joseph Fierro, Benafsh Sapra, Becky Tsai, Yan Meng, Doreen Ng, James Xie, Clinton R. Paden, Duncan MacCannell |
| EPI_ISL_4358784 | hCoV-19/USA/NV-CDC-FG-118837/2021 | North America/USA/Nevada | 2021-09-04 | Fulgent Genetics | Centers for Disease Control and Prevention Division of Viral Diseases, Pathogen Discovery | Dakota Howard,Dhwani Batra,Peter Cook,Jason Caravas,Benjamin Rambo-Martin,Scott Sammons,Yvette Unoarumhi,Matthew Schmerer,Kristine Lacek,Tymeckia Kendall,Victoria Caban Figueroa,Shatavia Morrison,Christopher Gulvick,Erisa Sula,Harry Gao,Mickey Li,John Gao,Joseph Fierro,Benafsh Sapra,Becky Tsai,Yan Meng,Doreen Ng,James Xie,Clinton Paden,Duncan MacCannell |
| EPI_ISL_2598806 | hCoV-19/USA/NY-CDC-FG-037180/2021 | North America/USA/New York | 2021-06-02 | Fulgent Genetics | Centers for Disease Control and Prevention Division of Viral Diseases, Pathogen Discovery | Dakota Howard, Dhwani Batra, Peter W. Cook, Kara Moser, Adrian Paskey, Jason Caravas, Benjamin Rambo-Martin, Shatavia Morrison, Christopher Gulvick, Scott Sammons, Yvette Unoarumhi, Darlene Wagner, Matthew Schmerer, Harry Gao, Mickey Li, John Gao, Joseph Fierro, Benafsh Sapra, Becky Tsai, Yan Meng, Doreen Ng, James Xie, Clinton R. Paden, Duncan MacCannell |
| EPI_ISL_2158349 | hCoV-19/Colombia/NAR-INS-VG-2346/2021 | South America/Colombia/Narino | 2021-04-13 | FUNDACION HOSPITAL SAN PEDRO | Instituto Nacional de Salud- DirecciÃ³n de InvestigaciÃ³n en Salud PÃºblica | Katherine Laiton-Donato, Diego A. Ãlvarez-DÃ­az, Carlos Franco-MuÃ±oz, Hector Alejandro Ruiz-Moreno, Paola Rojas, Maria T. Herrera-SepÃºlveda, Diego AndrÃ©s Prada, Jhonnatan Reales-GonzÃ¡lez, Sheryll Corchuelo, Julian Naizaque, Jorge Rivera, Gerardo SantamarÃ­a, Sergio Gomez, Lisseth Pardo, Juan Camilo Martinez, Marta Lopez Blanco, Ãngela Alarcon Cruz, Diana Malo, Carmen Osorio, Magdalena Wiesner, Martha Lucia Ospina Martinez, Marcela Mercado-Reyes |
| EPI_ISL_1251945 | hCoV-19/Norway/2584/2021 | Europe/Norway/Oslo | 2021-01-31 | Furst Medical Laboratory | Norwegian Institute of Public Health, Department of Virology | Kathrine Stene-Johansen, Kamilla Heddeland Instefjord, Hilde Elshaug, Garcia Llorente Ignacio, Jon BrÃ¥te, Engebretsen Serina Beate,Pedersen Benedikte Nevjen, Debech Nadia, Atiya R Ali,Marie Paulsen Madsen, Rasmus Riis Kopperud, Hilde Vollan, Karoline Bragstad, Olav Hungnes |
| EPI_ISL_493356 | hCoV-19/Norway/2789/2020 | Europe/Norway/ | 2020-06-26 | Furst Medical Laboratory | Norwegian Institute of Public Health, Department of Virology | Kathrine Stene-Johansen, Kamilla Heddeland Instefjord, Hilde Elshaug, Rasmus Riis Kopperud, Karoline Bragstad, Olav Hungnes |
| EPI_ISL_1443816 | hCoV-19/USA/GA-GPHL-0161/2020 | North America/USA/Georgia | 2020-12-10 | GA Department of Public Health | GA Department of Public Health | Stacy Reeves, Jonathan Edwards, Cynthia Dixey, Tonia Parrott, Aliyah Fields, Taylor Smith |
| EPI_ISL_1620650 | hCoV-19/USA/GA-GPHL-0211/2021 | North America/USA/Georgia | 2021-01-28 | GA Department of Public Health | GA Department of Public Health | Stacy Reeves, Jonathan Edwards, Cynthia Dixey, Tonia Parrott, Aliyah Fields, Taylor Smith |
| EPI_ISL_4253950 | hCoV-19/USA/GA-GPHL-1775/2021 | North America/USA/Georgia | 2021-09-03 | GA Department of Public Health | GA Department of Public Health | Stacy Reeves, Sharmila Talekar, Jonathan Edwards, Cynthia Dixey, Tonia Parrott, Aliyah Fields, Taylor Smith |
| EPI_ISL_1008915 | hCoV-19/USA/GA-CDC-3978463-001/2021 | North America/USA/Georgia | 2021-01-28 | GA Department of Public Health Laboratory | Pathogen Discovery, Respiratory Viruses Branch, Division of Viral Diseases, Centers for Disease Control and Prevention | Ying Tao, Jing Zhang, Yan Li, Krista Queen, Anna Uehara, Peter Cook, Clinton R. Paden, Haibin Wang, Suxiang Tong |
| EPI_ISL_984739 | hCoV-19/Turkey/37-Ankara-GUMV-50768/2020 | Europe/Turkey/Ankara | 2020-11-20 | Gazi University Faculty of Medicine, Medical Virology Laboratory | Gazi University Faculty of Medicine, Medical Virology Laboratory | Erdem Åžahin, GÃ¼lendam BozdayÄ±, Hager Muftah, Selin YiÄŸit, Shaknoza Sarzhanova, Ã–zlem GÃ¼zel TunÃ§can, Murat Dizbay, IÅŸÄ±l Fidan, Kayhan Ã‡aÄŸlar |
| EPI_ISL_730570 | hCoV-19/Turkey/Ankara_GUMV_37230/2020 | Europe/Turkey/Ankara | 2020-10-04 | Gazi University Faculty of Medicine, Medical Virology Laboratory | Gazi University Faculty of Medicine, Medical Virology Laboratory | Erdem Åžahin, Hager Muftah, Selin YiÄŸit, Shaknoza Sarzhanova, Ã–zlem GÃ¼zel TunÃ§can, Murat Dizbay, IÅŸÄ±l Fidan, Kayhan Ã‡aÄŸlar, GÃ¼lendam BozdayÄ± |
| EPI_ISL_3673678 | hCoV-19/Colombia/SAN-GO27062/2021 | South America/Colombia/Santander | 2021-07-07 | Gencore - Universidad de los Andes | Gencore - Universidad de los Andes | Luisa Sacristan, Cristian Barrera, Gabriela Ariza, Marcela Guevara, Silvia Restrepo, Marcela Mercado |
| EPI_ISL_406798 | hCoV-19/Wuhan/WH01/2019 | Asia/China/Hubei | 2019-12-26 | General Hospital of Central Theater Command of People's Liberation Army of China | BGI & Institute of Microbiology, Chinese Academy of Sciences & Shandong First Medical University & Shandong Academy of Medical Sciences & General Hospital of Central Theater Command of People's Liberation Army of China | Weijun Chen, Yuhai Bi, Weifeng Shi and Zhenhong Hu |
| EPI_ISL_1167888 | hCoV-19/Chile/AN-19048/2021 | South America/Chile/Antofagasta | 2021-01-13 | Genetica Molecular and Subdepartamento de Virologia ISP Chile | Instituto de Salud Publica de Chile | Javier Tognarelli, Karen Orostica, Barbara Parra, Loredana Arata, Jaime Lagos, Gisselle Barra, Patricia Bustos, Rodrigo Fasce, Andres Castillo, Jorge Fernandez |
| EPI_ISL_746492 | hCoV-19/Chile/AN-195419/2020 | South America/Chile/Antofagasta | 2020-10-01 | Genetica Molecular and Subdepartamento de Virologia ISP Chile | Instituto de Salud Publica de Chile | Javier Tognarelli, Barbara Parra, Loredana Arata, Jaime Lagos, Gisselle Barra, Patricia Bustos, Rodrigo Fasce, Andres Castillo, Jorge Fernandez |
| EPI_ISL_1167881 | hCoV-19/Chile/AN-267385/2020 | South America/Chile/Antofagasta | 2020-12-27 | Genetica Molecular and Subdepartamento de Virologia ISP Chile | Instituto de Salud Publica de Chile | Javier Tognarelli, Karen Orostica, Barbara Parra, Loredana Arata, Jaime Lagos, Gisselle Barra, Patricia Bustos, Rodrigo Fasce, Andres Castillo, Jorge Fernandez |
| EPI_ISL_1300469 | hCoV-19/Chile/AR-208478/2020 | South America/Chile/La Araucania | 2020-09-29 | Genetica Molecular and Subdepartamento de Virologia ISP Chile | Instituto de Salud Publica de Chile | Javier Tognarelli, Karen Orostica, Barbara Parra, Loredana Arata, Jaime Lagos, Gisselle Barra, Patricia Bustos, Rodrigo Fasce, Andres Castillo, Jorge Fernandez |
| EPI_ISL_1167827 | hCoV-19/Chile/AR-253614/2020 | South America/Chile/La Araucania | 2020-11-21 | Genetica Molecular and Subdepartamento de Virologia ISP Chile | Instituto de Salud Publica de Chile | Javier Tognarelli, Karen Orostica, Barbara Parra, Loredana Arata, Jaime Lagos, Gisselle Barra, Patricia Bustos, Rodrigo Fasce, Andres Castillo, Jorge Fernandez |
| EPI_ISL_3369331 | hCoV-19/Chile/AT-130311/2021 | South America/Chile/Atacama | 2021-07-25 | Genetica Molecular and Subdepartamento de Virologia ISP Chile | Instituto de Salud Publica de Chile | Karen Orostica, Constanza Campano, Barbara Parra, Loredana Arata, Gisselle Barra, Patricia Bustos, Rodrigo Fasce, Javier Tognarelli, Andres Castillo, Soledad Ulloa, Jorge Fernandez |
| EPI_ISL_3987629 | hCoV-19/Chile/LI-136753/2021 | South America/Chile/O'Higgins | 2021-08-09 | Genetica Molecular and Subdepartamento de Virologia ISP Chile | Instituto de Salud Publica de Chile | Karen Orostica, Constanza Campano, Barbara Parra, Loredana Arata, Gisselle Barra, Patricia Bustos, Rodrigo Fasce, Javier Tognarelli, Andres Castillo, Soledad Ulloa, Jorge Fernandez |
| EPI_ISL_2009225 | hCoV-19/Chile/LI-79417/2021 | South America/Chile/O'Higgins | 2021-02-17 | Genetica Molecular and Subdepartamento de Virologia ISP Chile | Instituto de Salud Publica de Chile | Javier Tognarelli, Karen Orostica, Barbara Parra, Loredana Arata, Gisselle Barra, Patricia Bustos, Rodrigo Fasce, Andres Castillo, Soledad Ulloa, Jorge Fernandez |
| EPI_ISL_1167796 | hCoV-19/Chile/ML-248656/2020 | South America/Chile/Maule | 2020-12-07 | Genetica Molecular and Subdepartamento de Virologia ISP Chile | Instituto de Salud Publica de Chile | Javier Tognarelli, Karen Orostica, Barbara Parra, Loredana Arata, Jaime Lagos, Gisselle Barra, Patricia Bustos, Rodrigo Fasce, Andres Castillo, Jorge Fernandez |
| EPI_ISL_1300452 | hCoV-19/Chile/NB-175520/2020 | South America/Chile/Ã‘uble | 2020-09-07 | Genetica Molecular and Subdepartamento de Virologia ISP Chile | Instituto de Salud Publica de Chile | Javier Tognarelli, Karen Orostica, Barbara Parra, Loredana Arata, Jaime Lagos, Gisselle Barra, Patricia Bustos, Rodrigo Fasce, Andres Castillo, Jorge Fernandez |
| EPI_ISL_746537 | hCoV-19/Chile/NB-175522/2020 | South America/Chile/Ã‘uble | 2020-09-11 | Genetica Molecular and Subdepartamento de Virologia ISP Chile | Instituto de Salud Publica de Chile | Javier Tognarelli, Barbara Parra, Loredana Arata, Jaime Lagos, Gisselle Barra, Patricia Bustos, Rodrigo Fasce, Andres Castillo, Jorge Fernandez |
| EPI_ISL_3536121 | hCoV-19/Chile/RM-133104/2021 | South America/Chile/Region Metropolitana de Santiago | 2021-08-01 | Genetica Molecular and Subdepartamento de Virologia ISP Chile | Instituto de Salud Publica de Chile | Karen Orostica, Constanza Campano, Barbara Parra, Loredana Arata, Gisselle Barra, Patricia Bustos, Rodrigo Fasce, Javier Tognarelli, Andres Castillo, Soledad Ulloa, Jorge Fernandez |
| EPI_ISL_746737 | hCoV-19/Chile/RM-187968/2020 | South America/Chile/Region Metropolitana de Santiago | 2020-09-23 | Genetica Molecular and Subdepartamento de Virologia ISP Chile | Instituto de Salud Publica de Chile | Javier Tognarelli, Barbara Parra, Loredana Arata, Jaime Lagos, Gisselle Barra, Patricia Bustos, Rodrigo Fasce, Andres Castillo, Jorge Fernandez |
| EPI_ISL_1321447 | hCoV-19/Chile/RM-43288/2021 | South America/Chile/Region Metropolitana de Santiago | 2021-03-03 | Genetica Molecular and Subdepartamento de Virologia ISP Chile | Instituto de Salud Publica de Chile | Javier Tognarelli, Karen Orostica, Barbara Parra, Loredana Arata, Jaime Lagos, Gisselle Barra, Patricia Bustos, Rodrigo Fasce, Andres Castillo, Jorge Fernandez |
| EPI_ISL_3988165 | hCoV-19/Chile/VS-139762/2021 | South America/Chile/Valparaiso | 2021-08-15 | Genetica Molecular and Subdepartamento de Virologia ISP Chile | Instituto de Salud Publica de Chile | Karen Orostica, Constanza Campano, Barbara Parra, Loredana Arata, Gisselle Barra, Patricia Bustos, Rodrigo Fasce, Javier Tognarelli, Andres Castillo, Soledad Ulloa, Jorge Fernandez |
| EPI_ISL_1300461 | hCoV-19/Chile/VS-201677/2020 | South America/Chile/Valparaiso | 2020-10-05 | Genetica Molecular and Subdepartamento de Virologia ISP Chile | Instituto de Salud Publica de Chile | Javier Tognarelli, Karen Orostica, Barbara Parra, Loredana Arata, Jaime Lagos, Gisselle Barra, Patricia Bustos, Rodrigo Fasce, Andres Castillo, Jorge Fernandez |
| EPI_ISL_1167817 | hCoV-19/Chile/VS-39494/2020 | South America/Chile/Valparaiso | 2020-04-07 | Genetica Molecular and Subdepartamento de Virologia ISP Chile | Instituto de Salud Publica de Chile | Javier Tognarelli, Karen Orostica, Barbara Parra, Loredana Arata, Jaime Lagos, Gisselle Barra, Patricia Bustos, Rodrigo Fasce, Andres Castillo, Jorge Fernandez |
| EPI_ISL_730227 | hCoV-19/Mexico/BCN-ALSR-4952/2020 | North America/Mexico/Baja California | 2020-08-02 | Genomica Lab Molecular, MâˆšÂ©xico | Andersen lab at Scripps Research | SEARCH Alliance San Diego with Jonathan Gonzalez Garcia, Jose Roman Chavez Mendez, Jose Horacio Reyna Verdugo, Martin Gonzalez Ibarra, Luis Alberto Rangel Gonzalez |
| EPI_ISL_1081440 | hCoV-19/Mexico/BCN-ALSR-6601/2020 | North America/Mexico/Baja California | 2020-12-21 | Genomica Lab Molecular, MâˆšÂ©xico | Andersen lab at Scripps Research | SEARCH Alliance San Diego with Jonathan Gonzalez Garcia, Jose Roman Chavez Mendez, Jose Horacio Reyna Verdugo, Martin Gonzalez Ibarra, Luis Alberto Rangel Gonzalez |
| EPI_ISL_2340640 | hCoV-19/USA/TX-GHRC-HD00174558/2021 | North America/USA/Texas | 2021-01-20 | GHRC | TxGen | Benjamin W. Neuman, Melissa M. Kahl-Mcdonagh, Kurt A. Zuelke, Charlie D. Johnson, Allison Ficht, Sankar P. Chaki, Sierra Guidry, Joshua Hill, Richard Metz, Marcel Brun |
| EPI_ISL_745888 | hCoV-19/USA/UT-UPHL-2012085638/2020 | North America/USA/Utah | 2020-08-13 | Ginkgo Bioworks Clinical Laboratory | Utah Public Health Laboratory | Erin L. Young, Kelly Oakeson, Tara Gallagher, Michael T. Pyne, E. Susan Slechta, Melanie A. Mallory, Jeffrey B. Stevenson, Salika M. Shakir, David R. Hillyard, Malaika McKenzie-Bennett, James McGann, Jim Griffin, Keith Robison, Alex Plocik, Becky Schilling, Martha Pierson, Rebecca Littlefield, Michelle Spencer, Birgitte Simen |
| EPI_ISL_746165 | hCoV-19/USA/UT-UPHL-2012091811/2020 | North America/USA/Utah | 2020-08-14 | Ginkgo Bioworks Clinical Laboratory | Utah Public Health Laboratory | Erin L. Young, Kelly Oakeson, Tara Gallagher, Michael T. Pyne, E. Susan Slechta, Melanie A. Mallory, Jeffrey B. Stevenson, Salika M. Shakir, David R. Hillyard, Malaika McKenzie-Bennett, James McGann, Jim Griffin, Keith Robison, Alex Plocik, Becky Schilling, Martha Pierson, Rebecca Littlefield, Michelle Spencer, Birgitte Simen |
| EPI_ISL_746122 | hCoV-19/USA/UT-UPHL-2012524544/2020 | North America/USA/Utah | 2020-08-15 | Ginkgo Bioworks Clinical Laboratory | Utah Public Health Laboratory | Erin L. Young, Kelly Oakeson, Tara Gallagher, Michael T. Pyne, E. Susan Slechta, Melanie A. Mallory, Jeffrey B. Stevenson, Salika M. Shakir, David R. Hillyard, Malaika McKenzie-Bennett, James McGann, Jim Griffin, Keith Robison, Alex Plocik, Becky Schilling, Martha Pierson, Rebecca Littlefield, Michelle Spencer, Birgitte Simen |
| EPI_ISL_745909 | hCoV-19/USA/UT-UPHL-2012841191/2020 | North America/USA/Utah | 2020-08-13 | Ginkgo Bioworks Clinical Laboratory | Utah Public Health Laboratory | Erin L. Young, Kelly Oakeson, Tara Gallagher, Michael T. Pyne, E. Susan Slechta, Melanie A. Mallory, Jeffrey B. Stevenson, Salika M. Shakir, David R. Hillyard, Malaika McKenzie-Bennett, James McGann, Jim Griffin, Keith Robison, Alex Plocik, Becky Schilling, Martha Pierson, Rebecca Littlefield, Michelle Spencer, Birgitte Simen |
| EPI_ISL_745851 | hCoV-19/USA/UT-UPHL-2012842916/2020 | North America/USA/Utah | 2020-08-10 | Ginkgo Bioworks Clinical Laboratory | Utah Public Health Laboratory | Erin L. Young, Kelly Oakeson, Tara Gallagher, Michael T. Pyne, E. Susan Slechta, Melanie A. Mallory, Jeffrey B. Stevenson, Salika M. Shakir, David R. Hillyard, Malaika McKenzie-Bennett, James McGann, Jim Griffin, Keith Robison, Alex Plocik, Becky Schilling, Martha Pierson, Rebecca Littlefield, Michelle Spencer, Birgitte Simen |
| EPI_ISL_745895 | hCoV-19/USA/UT-UPHL-2012896384/2020 | North America/USA/Utah | 2020-08-12 | Ginkgo Bioworks Clinical Laboratory | Utah Public Health Laboratory | Erin L. Young, Kelly Oakeson, Tara Gallagher, Michael T. Pyne, E. Susan Slechta, Melanie A. Mallory, Jeffrey B. Stevenson, Salika M. Shakir, David R. Hillyard, Malaika McKenzie-Bennett, James McGann, Jim Griffin, Keith Robison, Alex Plocik, Becky Schilling, Martha Pierson, Rebecca Littlefield, Michelle Spencer, Birgitte Simen |
| EPI_ISL_745591 | hCoV-19/USA/UT-UPHL-2012902224/2020 | North America/USA/Utah | 2020-07-26 | Ginkgo Bioworks Clinical Laboratory | Utah Public Health Laboratory | Erin L. Young, Kelly Oakeson, Tara Gallagher, Michael T. Pyne, E. Susan Slechta, Melanie A. Mallory, Jeffrey B. Stevenson, Salika M. Shakir, David R. Hillyard, Malaika McKenzie-Bennett, James McGann, Jim Griffin, Keith Robison, Alex Plocik, Becky Schilling, Martha Pierson, Rebecca Littlefield, Michelle Spencer, Birgitte Simen |
| EPI_ISL_745879 | hCoV-19/USA/UT-UPHL-2012972043/2020 | North America/USA/Utah | 2020-08-13 | Ginkgo Bioworks Clinical Laboratory | Utah Public Health Laboratory | Erin L. Young, Kelly Oakeson, Tara Gallagher, Michael T. Pyne, E. Susan Slechta, Melanie A. Mallory, Jeffrey B. Stevenson, Salika M. Shakir, David R. Hillyard, Malaika McKenzie-Bennett, James McGann, Jim Griffin, Keith Robison, Alex Plocik, Becky Schilling, Martha Pierson, Rebecca Littlefield, Michelle Spencer, Birgitte Simen |
| EPI_ISL_1626962 | hCoV-19/USA/TX-GHRC-EQ04531232/2021 | North America/USA/Texas | 2021-01-28 | Global Health Research Complex | TxGen | Benjamin W. Neuman, Melissa M. Kahl-Mcdonagh, Kurt A. Zuelke, Charlie D. Johnson, Allison Ficht, Sankar P. Chaki, Sierra Guidry, Joshua Hill, Richard Metz, Marcel Brun |
| EPI_ISL_1626965 | hCoV-19/USA/TX-GHRC-EQ04531413/2021 | North America/USA/Texas | 2021-01-28 | Global Health Research Complex | TxGen | Benjamin W. Neuman, Melissa M. Kahl-Mcdonagh, Kurt A. Zuelke, Charlie D. Johnson, Allison Ficht, Sankar P. Chaki, Sierra Guidry, Joshua Hill, Richard Metz, Marcel Brun |
| EPI_ISL_1626968 | hCoV-19/USA/TX-GHRC-EQ04532000/2021 | North America/USA/Texas | 2021-01-28 | Global Health Research Complex | TxGen | Benjamin W. Neuman, Melissa M. Kahl-Mcdonagh, Kurt A. Zuelke, Charlie D. Johnson, Allison Ficht, Sankar P. Chaki, Sierra Guidry, Joshua Hill, Richard Metz, Marcel Brun |
| EPI_ISL_1626975 | hCoV-19/USA/TX-GHRC-EQ04533897/2021 | North America/USA/Texas | 2021-01-28 | Global Health Research Complex | TxGen | Benjamin W. Neuman, Melissa M. Kahl-Mcdonagh, Kurt A. Zuelke, Charlie D. Johnson, Allison Ficht, Sankar P. Chaki, Sierra Guidry, Joshua Hill, Richard Metz, Marcel Brun |
| EPI_ISL_1626981 | hCoV-19/USA/TX-GHRC-EQ04535097/2021 | North America/USA/Texas | 2021-01-28 | Global Health Research Complex | TxGen | Benjamin W. Neuman, Melissa M. Kahl-Mcdonagh, Kurt A. Zuelke, Charlie D. Johnson, Allison Ficht, Sankar P. Chaki, Sierra Guidry, Joshua Hill, Richard Metz, Marcel Brun |
| EPI_ISL_1626987 | hCoV-19/USA/TX-GHRC-EQ04535553/2021 | North America/USA/Texas | 2021-01-28 | Global Health Research Complex | TxGen | Benjamin W. Neuman, Melissa M. Kahl-Mcdonagh, Kurt A. Zuelke, Charlie D. Johnson, Allison Ficht, Sankar P. Chaki, Sierra Guidry, Joshua Hill, Richard Metz, Marcel Brun |
| EPI_ISL_1001457 | hCoV-19/Panama/GMI-PA376271/2020 | North America/Panama/Panama City | 2020-06-07 | Gorgas memorial Institute For Health Studies | Gorgas memorial Institute For Health Studies | DÃ­az Y, Castillo D, Moreno B, Castillo M,GonzÃ¡lez C, Gondola J, Moreno A, Pitti Y, Chavarria O, Franco D, Saenz L, Gaitan M, Arauz D, Martinez AA, Lopez-Verges S. |
| EPI_ISL_496705 | hCoV-19/Panama/332238/2020 | North America/Panama/Cocle | 2020-03-14 | Gorgas Memorial Laboratory of Health Studies | Gorgas Memorial Laboratory of Health Studies | Danilo Franco, Claudia Gonzalez Sandra Lopez-Verges, Alexander A Martinez |
| EPI_ISL_496748 | hCoV-19/Panama/333568/2020 | North America/Panama/Cocle | 2020-03-16 | Gorgas Memorial Laboratory of Health Studies | Gorgas Memorial Laboratory of Health Studies | Danilo Franco, Claudia Gonzalez Sandra Lopez-Verges, Alexander A Martinez |
| EPI_ISL_496802 | hCoV-19/Panama/335067/2020 | North America/Panama/Panama Center | 2020-04-01 | Gorgas Memorial Laboratory of Health Studies | Gorgas Memorial Laboratory of Health Studies | Danilo Franco, Claudia Gonzalez Sandra Lopez-Verges, Alexander A Martinez |
| EPI_ISL_496862 | hCoV-19/Panama/337640/2020 | North America/Panama/Chiriqui | 2020-07-04 | Gorgas Memorial Laboratory of Health Studies | Gorgas Memorial Laboratory of Health Studies | Danilo Franco, Claudia Gonzalez Sandra Lopez-Verges, Alexander A Martinez |
| EPI_ISL_496863 | hCoV-19/Panama/337660/2020 | North America/Panama/Panama West | 2020-07-04 | Gorgas Memorial Laboratory of Health Studies | Gorgas Memorial Laboratory of Health Studies | Danilo Franco, Claudia Gonzalez Sandra Lopez-Verges, Alexander A Martinez |
| EPI_ISL_496878 | hCoV-19/Panama/338677/2020 | North America/Panama/Colon | 2020-04-09 | Gorgas Memorial Laboratory of Health Studies | Gorgas Memorial Laboratory of Health Studies | Danilo Franco, Claudia Gonzalez Sandra Lopez-Verges, Alexander A Martinez |
| EPI_ISL_1502866 | hCoV-19/Panama/GMI-PA1058/2020 | North America/Panama/Los Santos | 2020-07-02 | Gorgas Memorial Laboratory of Health Studies | Gorgas Memorial Laboratory of Health Studies | Gonzalez Claudia, Leyda Abrego, Moreno Ambar, Oris Chavarria, Jessica Gondola, Marlenne Castillo, Ortiz Alma, Castillo Jorge, Moreno Brechla, Franco Danilo, Lopez-Verges Sandra, Martinez Alexander |
| EPI_ISL_1502852 | hCoV-19/Panama/GMI-PA233/2020 | North America/Panama/Los Santos | 2020-05-19 | Gorgas Memorial Laboratory of Health Studies | Gorgas Memorial Laboratory of Health Studies | Gonzalez Claudia, Leyda Abrego, Moreno Ambar, Oris Chavarria, Jessica Gondola, Marlenne Castillo, Ortiz Alma, Castillo Jorge, Moreno Brechla, Franco Danilo, Lopez-Verges Sandra, Martinez Alexander |
| EPI_ISL_1225337 | hCoV-19/Panama/GMI-PA342491/2020 | North America/Panama/Bocas del Toro | 2020-04-16 | Gorgas Memorial Laboratory of Health Studies | Gorgas Memorial Laboratory of Health Studies | Yamilka Diaz, Anyuri Ortiz, Adriana Weeden, Daniel Castillo, Claudia Gonzalez, Brechla Moreno, Mabel Martinez-Montero, Marlene Castillo, Gretel Vasquez, Lisseth Saenz, Danilo Franco, Yaneth Pitti, Oris Chavarria, Jessica Gondola, Ambar Moreno, Layda Abrego, Davis Beltran, Ilka Guerra, Jim Chang, Zumara Chaverra, Isela Guerrero, Alejandra Valoy, Melissa Gaitan, Dimelza Arauz, Maria Chen-German, Elimelec Valdespino, Rita Rodriguez, Rita Corrales, Juan Miguel Pascale, Alexander Martinez, Sandra Lopez-Verges |
| EPI_ISL_1225352 | hCoV-19/Panama/GMI-PA345048/2020 | North America/Panama/Cocle | 2020-04-20 | Gorgas Memorial Laboratory of Health Studies | Gorgas Memorial Laboratory of Health Studies | Yamilka Diaz, Anyuri Ortiz, Adriana Weeden, Daniel Castillo, Claudia Gonzalez, Brechla Moreno, Mabel Martinez-Montero, Marlene Castillo, Gretel Vasquez, Lisseth Saenz, Danilo Franco, Yaneth Pitti, Oris Chavarria, Jessica Gondola, Ambar Moreno, Layda Abrego, Davis Beltran, Ilka Guerra, Jim Chang, Zumara Chaverra, Isela Guerrero, Alejandra Valoy, Melissa Gaitan, Dimelza Arauz, Maria Chen-German, Elimelec Valdespino, Rita Rodriguez, Rita Corrales, Juan Miguel Pascale, Alexander Martinez, Sandra Lopez-Verges |
| EPI_ISL_1225358 | hCoV-19/Panama/GMI-PA346059/2020 | North America/Panama/Santiago de Veraguas | 2020-04-22 | Gorgas Memorial Laboratory of Health Studies | Gorgas Memorial Laboratory of Health Studies | Yamilka Diaz, Anyuri Ortiz, Adriana Weeden, Daniel Castillo, Claudia Gonzalez, Brechla Moreno, Mabel Martinez-Montero, Marlene Castillo, Gretel Vasquez, Lisseth Saenz, Danilo Franco, Yaneth Pitti, Oris Chavarria, Jessica Gondola, Ambar Moreno, Layda Abrego, Davis Beltran, Ilka Guerra, Jim Chang, Zumara Chaverra, Isela Guerrero, Alejandra Valoy, Melissa Gaitan, Dimelza Arauz, Maria Chen-German, Elimelec Valdespino, Rita Rodriguez, Rita Corrales, Juan Miguel Pascale, Alexander Martinez, Sandra Lopez-Verges |
| EPI_ISL_1225450 | hCoV-19/Panama/GMI-PA369162/2020 | North America/Panama/Guna Yala | 2020-05-28 | Gorgas Memorial Laboratory of Health Studies | Gorgas Memorial Laboratory of Health Studies | Yamilka Diaz, Anyuri Ortiz, Adriana Weeden, Daniel Castillo, Claudia Gonzalez, Brechla Moreno, Mabel Martinez-Montero, Marlene Castillo, Gretel Vasquez, Lisseth Saenz, Danilo Franco, Yaneth Pitti, Oris Chavarria, Jessica Gondola, Ambar Moreno, Layda Abrego, Davis Beltran, Ilka Guerra, Jim Chang, Zumara Chaverra, Isela Guerrero, Alejandra Valoy, Melissa Gaitan, Dimelza Arauz, Maria Chen-German, Elimelec Valdespino, Rita Rodriguez, Rita Corrales, Juan Miguel Pascale, Alexander Martinez, Sandra Lopez-Verges |
| EPI_ISL_1225452 | hCoV-19/Panama/GMI-PA369165/2020 | North America/Panama/Guna Yala | 2020-05-28 | Gorgas Memorial Laboratory of Health Studies | Gorgas Memorial Laboratory of Health Studies | Yamilka Diaz, Anyuri Ortiz, Adriana Weeden, Daniel Castillo, Claudia Gonzalez, Brechla Moreno, Mabel Martinez-Montero, Marlene Castillo, Gretel Vasquez, Lisseth Saenz, Danilo Franco, Yaneth Pitti, Oris Chavarria, Jessica Gondola, Ambar Moreno, Layda Abrego, Davis Beltran, Ilka Guerra, Jim Chang, Zumara Chaverra, Isela Guerrero, Alejandra Valoy, Melissa Gaitan, Dimelza Arauz, Maria Chen-German, Elimelec Valdespino, Rita Rodriguez, Rita Corrales, Juan Miguel Pascale, Alexander Martinez, Sandra Lopez-Verges |
| EPI_ISL_1225455 | hCoV-19/Panama/GMI-PA369170/2020 | North America/Panama/Guna Yala | 2020-05-28 | Gorgas Memorial Laboratory of Health Studies | Gorgas Memorial Laboratory of Health Studies | Yamilka Diaz, Anyuri Ortiz, Adriana Weeden, Daniel Castillo, Claudia Gonzalez, Brechla Moreno, Mabel Martinez-Montero, Marlene Castillo, Gretel Vasquez, Lisseth Saenz, Danilo Franco, Yaneth Pitti, Oris Chavarria, Jessica Gondola, Ambar Moreno, Layda Abrego, Davis Beltran, Ilka Guerra, Jim Chang, Zumara Chaverra, Isela Guerrero, Alejandra Valoy, Melissa Gaitan, Dimelza Arauz, Maria Chen-German, Elimelec Valdespino, Rita Rodriguez, Rita Corrales, Juan Miguel Pascale, Alexander Martinez, Sandra Lopez-Verges |
| EPI_ISL_1225462 | hCoV-19/Panama/GMI-PA370376/2020 | North America/Panama/Colon | 2020-05-30 | Gorgas Memorial Laboratory of Health Studies | Gorgas Memorial Laboratory of Health Studies | Yamilka Diaz, Anyuri Ortiz, Adriana Weeden, Daniel Castillo, Claudia Gonzalez, Brechla Moreno, Mabel Martinez-Montero, Marlene Castillo, Gretel Vasquez, Lisseth Saenz, Danilo Franco, Yaneth Pitti, Oris Chavarria, Jessica Gondola, Ambar Moreno, Layda Abrego, Davis Beltran, Ilka Guerra, Jim Chang, Zumara Chaverra, Isela Guerrero, Alejandra Valoy, Melissa Gaitan, Dimelza Arauz, Maria Chen-German, Elimelec Valdespino, Rita Rodriguez, Rita Corrales, Juan Miguel Pascale, Alexander Martinez, Sandra Lopez-Verges |
| EPI_ISL_1225466 | hCoV-19/Panama/GMI-PA380508/2020 | North America/Panama/Darien | 2020-06-11 | Gorgas Memorial Laboratory of Health Studies | Gorgas Memorial Laboratory of Health Studies | Yamilka Diaz, Anyuri Ortiz, Adriana Weeden, Daniel Castillo, Claudia Gonzalez, Brechla Moreno, Mabel Martinez-Montero, Marlene Castillo, Gretel Vasquez, Lisseth Saenz, Danilo Franco, Yaneth Pitti, Oris Chavarria, Jessica Gondola, Ambar Moreno, Layda Abrego, Davis Beltran, Ilka Guerra, Jim Chang, Zumara Chaverra, Isela Guerrero, Alejandra Valoy, Melissa Gaitan, Dimelza Arauz, Maria Chen-German, Elimelec Valdespino, Rita Rodriguez, Rita Corrales, Juan Miguel Pascale, Alexander Martinez, Sandra Lopez-Verges |
| EPI_ISL_1225467 | hCoV-19/Panama/GMI-PA380510/2020 | North America/Panama/Bocas del Toro | 2020-06-11 | Gorgas Memorial Laboratory of Health Studies | Gorgas Memorial Laboratory of Health Studies | Yamilka Diaz, Anyuri Ortiz, Adriana Weeden, Daniel Castillo, Claudia Gonzalez, Brechla Moreno, Mabel Martinez-Montero, Marlene Castillo, Gretel Vasquez, Lisseth Saenz, Danilo Franco, Yaneth Pitti, Oris Chavarria, Jessica Gondola, Ambar Moreno, Layda Abrego, Davis Beltran, Ilka Guerra, Jim Chang, Zumara Chaverra, Isela Guerrero, Alejandra Valoy, Melissa Gaitan, Dimelza Arauz, Maria Chen-German, Elimelec Valdespino, Rita Rodriguez, Rita Corrales, Juan Miguel Pascale, Alexander Martinez, Sandra Lopez-Verges |
| EPI_ISL_1225469 | hCoV-19/Panama/GMI-PA382224/2020 | North America/Panama/Panama North | 2020-06-12 | Gorgas Memorial Laboratory of Health Studies | Gorgas Memorial Laboratory of Health Studies | Yamilka Diaz, Anyuri Ortiz, Adriana Weeden, Daniel Castillo, Claudia Gonzalez, Brechla Moreno, Mabel Martinez-Montero, Marlene Castillo, Gretel Vasquez, Lisseth Saenz, Danilo Franco, Yaneth Pitti, Oris Chavarria, Jessica Gondola, Ambar Moreno, Layda Abrego, Davis Beltran, Ilka Guerra, Jim Chang, Zumara Chaverra, Isela Guerrero, Alejandra Valoy, Melissa Gaitan, Dimelza Arauz, Maria Chen-German, Elimelec Valdespino, Rita Rodriguez, Rita Corrales, Juan Miguel Pascale, Alexander Martinez, Sandra Lopez-Verges |
| EPI_ISL_1225481 | hCoV-19/Panama/GMI-PA391365/2020 | North America/Panama/Panama Center | 2020-06-21 | Gorgas Memorial Laboratory of Health Studies | Gorgas Memorial Laboratory of Health Studies | Yamilka Diaz, Anyuri Ortiz, Adriana Weeden, Daniel Castillo, Claudia Gonzalez, Brechla Moreno, Mabel Martinez-Montero, Marlene Castillo, Gretel Vasquez, Lisseth Saenz, Danilo Franco, Yaneth Pitti, Oris Chavarria, Jessica Gondola, Ambar Moreno, Layda Abrego, Davis Beltran, Ilka Guerra, Jim Chang, Zumara Chaverra, Isela Guerrero, Alejandra Valoy, Melissa Gaitan, Dimelza Arauz, Maria Chen-German, Elimelec Valdespino, Rita Rodriguez, Rita Corrales, Juan Miguel Pascale, Alexander Martinez, Sandra Lopez-Verges |
| EPI_ISL_1225489 | hCoV-19/Panama/GMI-PA398110/2020 | North America/Panama/Guna Yala | 2020-06-26 | Gorgas Memorial Laboratory of Health Studies | Gorgas Memorial Laboratory of Health Studies | Yamilka Diaz, Anyuri Ortiz, Adriana Weeden, Daniel Castillo, Claudia Gonzalez, Brechla Moreno, Mabel Martinez-Montero, Marlene Castillo, Gretel Vasquez, Lisseth Saenz, Danilo Franco, Yaneth Pitti, Oris Chavarria, Jessica Gondola, Ambar Moreno, Layda Abrego, Davis Beltran, Ilka Guerra, Jim Chang, Zumara Chaverra, Isela Guerrero, Alejandra Valoy, Melissa Gaitan, Dimelza Arauz, Maria Chen-German, Elimelec Valdespino, Rita Rodriguez, Rita Corrales, Juan Miguel Pascale, Alexander Martinez, Sandra Lopez-Verges |
| EPI_ISL_1225490 | hCoV-19/Panama/GMI-PA398254/2020 | North America/Panama/Chiriqui | 2020-06-26 | Gorgas Memorial Laboratory of Health Studies | Gorgas Memorial Laboratory of Health Studies | Yamilka Diaz, Anyuri Ortiz, Adriana Weeden, Daniel Castillo, Claudia Gonzalez, Brechla Moreno, Mabel Martinez-Montero, Marlene Castillo, Gretel Vasquez, Lisseth Saenz, Danilo Franco, Yaneth Pitti, Oris Chavarria, Jessica Gondola, Ambar Moreno, Layda Abrego, Davis Beltran, Ilka Guerra, Jim Chang, Zumara Chaverra, Isela Guerrero, Alejandra Valoy, Melissa Gaitan, Dimelza Arauz, Maria Chen-German, Elimelec Valdespino, Rita Rodriguez, Rita Corrales, Juan Miguel Pascale, Alexander Martinez, Sandra Lopez-Verges |
| EPI_ISL_1225499 | hCoV-19/Panama/GMI-PA403167/2020 | North America/Panama/Panama Center | 2020-07-01 | Gorgas Memorial Laboratory of Health Studies | Gorgas Memorial Laboratory of Health Studies | Yamilka Diaz, Anyuri Ortiz, Adriana Weeden, Daniel Castillo, Claudia Gonzalez, Brechla Moreno, Mabel Martinez-Montero, Marlene Castillo, Gretel Vasquez, Lisseth Saenz, Danilo Franco, Yaneth Pitti, Oris Chavarria, Jessica Gondola, Ambar Moreno, Layda Abrego, Davis Beltran, Ilka Guerra, Jim Chang, Zumara Chaverra, Isela Guerrero, Alejandra Valoy, Melissa Gaitan, Dimelza Arauz, Maria Chen-German, Elimelec Valdespino, Rita Rodriguez, Rita Corrales, Juan Miguel Pascale, Alexander Martinez, Sandra Lopez-Verges |
| EPI_ISL_1225510 | hCoV-19/Panama/GMI-PA419159/2020 | North America/Panama/Panama Center | 2020-07-14 | Gorgas Memorial Laboratory of Health Studies | Gorgas Memorial Laboratory of Health Studies | Yamilka Diaz, Anyuri Ortiz, Adriana Weeden, Daniel Castillo, Claudia Gonzalez, Brechla Moreno, Mabel Martinez-Montero, Marlene Castillo, Gretel Vasquez, Lisseth Saenz, Danilo Franco, Yaneth Pitti, Oris Chavarria, Jessica Gondola, Ambar Moreno, Layda Abrego, Davis Beltran, Ilka Guerra, Jim Chang, Zumara Chaverra, Isela Guerrero, Alejandra Valoy, Melissa Gaitan, Dimelza Arauz, Maria Chen-German, Elimelec Valdespino, Rita Rodriguez, Rita Corrales, Juan Miguel Pascale, Alexander Martinez, Sandra Lopez-Verges |
| EPI_ISL_1225513 | hCoV-19/Panama/GMI-PA425903/2020 | North America/Panama/Bocas del Toro | 2020-07-17 | Gorgas Memorial Laboratory of Health Studies | Gorgas Memorial Laboratory of Health Studies | Yamilka Diaz, Anyuri Ortiz, Adriana Weeden, Daniel Castillo, Claudia Gonzalez, Brechla Moreno, Mabel Martinez-Montero, Marlene Castillo, Gretel Vasquez, Lisseth Saenz, Danilo Franco, Yaneth Pitti, Oris Chavarria, Jessica Gondola, Ambar Moreno, Layda Abrego, Davis Beltran, Ilka Guerra, Jim Chang, Zumara Chaverra, Isela Guerrero, Alejandra Valoy, Melissa Gaitan, Dimelza Arauz, Maria Chen-German, Elimelec Valdespino, Rita Rodriguez, Rita Corrales, Juan Miguel Pascale, Alexander Martinez, Sandra Lopez-Verges |
| EPI_ISL_1225515 | hCoV-19/Panama/GMI-PA426757/2020 | North America/Panama/Cocle | 2020-07-20 | Gorgas Memorial Laboratory of Health Studies | Gorgas Memorial Laboratory of Health Studies | Yamilka Diaz, Anyuri Ortiz, Adriana Weeden, Daniel Castillo, Claudia Gonzalez, Brechla Moreno, Mabel Martinez-Montero, Marlene Castillo, Gretel Vasquez, Lisseth Saenz, Danilo Franco, Yaneth Pitti, Oris Chavarria, Jessica Gondola, Ambar Moreno, Layda Abrego, Davis Beltran, Ilka Guerra, Jim Chang, Zumara Chaverra, Isela Guerrero, Alejandra Valoy, Melissa Gaitan, Dimelza Arauz, Maria Chen-German, Elimelec Valdespino, Rita Rodriguez, Rita Corrales, Juan Miguel Pascale, Alexander Martinez, Sandra Lopez-Verges |
| EPI_ISL_1225530 | hCoV-19/Panama/GMI-PA437909/2020 | North America/Panama/Cocle | 2020-07-29 | Gorgas Memorial Laboratory of Health Studies | Gorgas Memorial Laboratory of Health Studies | Yamilka Diaz, Anyuri Ortiz, Adriana Weeden, Daniel Castillo, Claudia Gonzalez, Brechla Moreno, Mabel Martinez-Montero, Marlene Castillo, Gretel Vasquez, Lisseth Saenz, Danilo Franco, Yaneth Pitti, Oris Chavarria, Jessica Gondola, Ambar Moreno, Layda Abrego, Davis Beltran, Ilka Guerra, Jim Chang, Zumara Chaverra, Isela Guerrero, Alejandra Valoy, Melissa Gaitan, Dimelza Arauz, Maria Chen-German, Elimelec Valdespino, Rita Rodriguez, Rita Corrales, Juan Miguel Pascale, Alexander Martinez, Sandra Lopez-Verges |
| EPI_ISL_1225540 | hCoV-19/Panama/GMI-PA449952/2020 | North America/Panama/Los Santos | 2020-08-05 | Gorgas Memorial Laboratory of Health Studies | Gorgas Memorial Laboratory of Health Studies | Yamilka Diaz, Anyuri Ortiz, Adriana Weeden, Daniel Castillo, Claudia Gonzalez, Brechla Moreno, Mabel Martinez-Montero, Marlene Castillo, Gretel Vasquez, Lisseth Saenz, Danilo Franco, Yaneth Pitti, Oris Chavarria, Jessica Gondola, Ambar Moreno, Layda Abrego, Davis Beltran, Ilka Guerra, Jim Chang, Zumara Chaverra, Isela Guerrero, Alejandra Valoy, Melissa Gaitan, Dimelza Arauz, Maria Chen-German, Elimelec Valdespino, Rita Rodriguez, Rita Corrales, Juan Miguel Pascale, Alexander Martinez, Sandra Lopez-Verges |
| EPI_ISL_1225541 | hCoV-19/Panama/GMI-PA449966/2020 | North America/Panama/Los Santos | 2020-08-05 | Gorgas Memorial Laboratory of Health Studies | Gorgas Memorial Laboratory of Health Studies | Yamilka Diaz, Anyuri Ortiz, Adriana Weeden, Daniel Castillo, Claudia Gonzalez, Brechla Moreno, Mabel Martinez-Montero, Marlene Castillo, Gretel Vasquez, Lisseth Saenz, Danilo Franco, Yaneth Pitti, Oris Chavarria, Jessica Gondola, Ambar Moreno, Layda Abrego, Davis Beltran, Ilka Guerra, Jim Chang, Zumara Chaverra, Isela Guerrero, Alejandra Valoy, Melissa Gaitan, Dimelza Arauz, Maria Chen-German, Elimelec Valdespino, Rita Rodriguez, Rita Corrales, Juan Miguel Pascale, Alexander Martinez, Sandra Lopez-Verges |
| EPI_ISL_1225545 | hCoV-19/Panama/GMI-PA451643/2020 | North America/Panama/Darien | 2020-08-08 | Gorgas Memorial Laboratory of Health Studies | Gorgas Memorial Laboratory of Health Studies | Yamilka Diaz, Anyuri Ortiz, Adriana Weeden, Daniel Castillo, Claudia Gonzalez, Brechla Moreno, Mabel Martinez-Montero, Marlene Castillo, Gretel Vasquez, Lisseth Saenz, Danilo Franco, Yaneth Pitti, Oris Chavarria, Jessica Gondola, Ambar Moreno, Layda Abrego, Davis Beltran, Ilka Guerra, Jim Chang, Zumara Chaverra, Isela Guerrero, Alejandra Valoy, Melissa Gaitan, Dimelza Arauz, Maria Chen-German, Elimelec Valdespino, Rita Rodriguez, Rita Corrales, Juan Miguel Pascale, Alexander Martinez, Sandra Lopez-Verges |
| EPI_ISL_1225548 | hCoV-19/Panama/GMI-PA461366/2020 | North America/Panama/Colon | 2020-08-16 | Gorgas Memorial Laboratory of Health Studies | Gorgas Memorial Laboratory of Health Studies | Yamilka Diaz, Anyuri Ortiz, Adriana Weeden, Daniel Castillo, Claudia Gonzalez, Brechla Moreno, Mabel Martinez-Montero, Marlene Castillo, Gretel Vasquez, Lisseth Saenz, Danilo Franco, Yaneth Pitti, Oris Chavarria, Jessica Gondola, Ambar Moreno, Layda Abrego, Davis Beltran, Ilka Guerra, Jim Chang, Zumara Chaverra, Isela Guerrero, Alejandra Valoy, Melissa Gaitan, Dimelza Arauz, Maria Chen-German, Elimelec Valdespino, Rita Rodriguez, Rita Corrales, Juan Miguel Pascale, Alexander Martinez, Sandra Lopez-Verges |
| EPI_ISL_1225574 | hCoV-19/Panama/GMI-PA571783/2020 | North America/Panama/Panama Center | 2020-11-29 | Gorgas Memorial Laboratory of Health Studies | Gorgas Memorial Laboratory of Health Studies | Yamilka Diaz, Anyuri Ortiz, Adriana Weeden, Daniel Castillo, Claudia Gonzalez, Brechla Moreno, Mabel Martinez-Montero, Marlene Castillo, Gretel Vasquez, Lisseth Saenz, Danilo Franco, Yaneth Pitti, Oris Chavarria, Jessica Gondola, Ambar Moreno, Layda Abrego, Davis Beltran, Ilka Guerra, Jim Chang, Zumara Chaverra, Isela Guerrero, Alejandra Valoy, Melissa Gaitan, Dimelza Arauz, Maria Chen-German, Elimelec Valdespino, Rita Rodriguez, Rita Corrales, Juan Miguel Pascale, Alexander Martinez, Sandra Lopez-Verges |
| EPI_ISL_1225577 | hCoV-19/Panama/GMI-PA572717/2020 | North America/Panama/San Miguelito | 2020-11-30 | Gorgas Memorial Laboratory of Health Studies | Gorgas Memorial Laboratory of Health Studies | Yamilka Diaz, Anyuri Ortiz, Adriana Weeden, Daniel Castillo, Claudia Gonzalez, Brechla Moreno, Mabel Martinez-Montero, Marlene Castillo, Gretel Vasquez, Lisseth Saenz, Danilo Franco, Yaneth Pitti, Oris Chavarria, Jessica Gondola, Ambar Moreno, Layda Abrego, Davis Beltran, Ilka Guerra, Jim Chang, Zumara Chaverra, Isela Guerrero, Alejandra Valoy, Melissa Gaitan, Dimelza Arauz, Maria Chen-German, Elimelec Valdespino, Rita Rodriguez, Rita Corrales, Juan Miguel Pascale, Alexander Martinez, Sandra Lopez-Verges |
| EPI_ISL_1502891 | hCoV-19/Panama/GMI-PA610371/2020 | North America/Panama/San Miguelito | 2020-12-20 | Gorgas Memorial Laboratory of Health Studies | Gorgas Memorial Laboratory of Health Studies | Gonzalez Claudia, Leyda Abrego, Moreno Ambar, Oris Chavarria, Jessica Gondola, Marlenne Castillo, Ortiz Alma, Castillo Jorge, Moreno Brechla, Franco Danilo, Lopez-Verges Sandra, Martinez Alexander |
| EPI_ISL_1502919 | hCoV-19/Panama/GMI-PA611139/2020 | North America/Panama/Panama Oeste | 2020-12-20 | Gorgas Memorial Laboratory of Health Studies | Gorgas Memorial Laboratory of Health Studies | Gonzalez Claudia, Leyda Abrego, Moreno Ambar, Oris Chavarria, Jessica Gondola, Marlenne Castillo, Ortiz Alma, Castillo Jorge, Moreno Brechla, Franco Danilo, Lopez-Verges Sandra, Martinez Alexander |
| EPI_ISL_1502973 | hCoV-19/Panama/GMI-PA646008/2021 | North America/Panama/Cocle | 2021-01-06 | Gorgas Memorial Laboratory of Health Studies | Gorgas Memorial Laboratory of Health Studies | Gonzalez Claudia, Leyda Abrego, Moreno Ambar, Oris Chavarria, Jessica Gondola, Marlenne Castillo, Ortiz Alma, Castillo Jorge, Moreno Brechla, Franco Danilo, Lopez-Verges Sandra, Martinez Alexander |
| EPI_ISL_1502987 | hCoV-19/Panama/GMI-PA655071/2021 | North America/Panama/Colon | 2021-01-10 | Gorgas Memorial Laboratory of Health Studies | Gorgas Memorial Laboratory of Health Studies | Gonzalez Claudia, Leyda Abrego, Moreno Ambar, Oris Chavarria, Jessica Gondola, Marlenne Castillo, Ortiz Alma, Castillo Jorge, Moreno Brechla, Franco Danilo, Lopez-Verges Sandra, Martinez Alexander |
| EPI_ISL_1503002 | hCoV-19/Panama/GMI-PA661264/2021 | North America/Panama/Metropolitana | 2021-01-13 | Gorgas Memorial Laboratory of Health Studies | Gorgas Memorial Laboratory of Health Studies | Gonzalez Claudia, Leyda Abrego, Moreno Ambar, Oris Chavarria, Jessica Gondola, Marlenne Castillo, Ortiz Alma, Castillo Jorge, Moreno Brechla, Franco Danilo, Lopez-Verges Sandra, Martinez Alexander |
| EPI_ISL_1503133 | hCoV-19/Panama/GMI-PA677161/2021 | North America/Panama/ | 2021-01-20 | Gorgas Memorial Laboratory of Health Studies | Gorgas Memorial Laboratory of Health Studies | Gonzalez Claudia, Leyda Abrego, Moreno Ambar, Oris Chavarria, Jessica Gondola, Marlenne Castillo, Ortiz Alma, Castillo Jorge, Moreno Brechla, Franco Danilo, Lopez-Verges Sandra, Martinez Alexander |
| EPI_ISL_1503140 | hCoV-19/Panama/GMI-PA679786/2021 | North America/Panama/ | 2021-01-22 | Gorgas Memorial Laboratory of Health Studies | Gorgas Memorial Laboratory of Health Studies | Gonzalez Claudia, Leyda Abrego, Moreno Ambar, Oris Chavarria, Jessica Gondola, Marlenne Castillo, Ortiz Alma, Castillo Jorge, Moreno Brechla, Franco Danilo, Lopez-Verges Sandra, Martinez Alexander |
| EPI_ISL_1503021 | hCoV-19/Panama/GMI-PA687696/2021 | North America/Panama/San Miguelito | 2021-01-27 | Gorgas Memorial Laboratory of Health Studies | Gorgas Memorial Laboratory of Health Studies | Gonzalez Claudia, Leyda Abrego, Moreno Ambar, Oris Chavarria, Jessica Gondola, Marlenne Castillo, Ortiz Alma, Castillo Jorge, Moreno Brechla, Franco Danilo, Lopez-Verges Sandra, Martinez Alexander |
| EPI_ISL_1503042 | hCoV-19/Panama/GMI-PA693809/2021 | North America/Panama/Cocle | 2021-02-01 | Gorgas Memorial Laboratory of Health Studies | Gorgas Memorial Laboratory of Health Studies | Gonzalez Claudia, Leyda Abrego, Moreno Ambar, Oris Chavarria, Jessica Gondola, Marlenne Castillo, Ortiz Alma, Castillo Jorge, Moreno Brechla, Franco Danilo, Lopez-Verges Sandra, Martinez Alexander |
| EPI_ISL_1503044 | hCoV-19/Panama/GMI-PA694051/2021 | North America/Panama/Panama Oeste | 2021-02-02 | Gorgas Memorial Laboratory of Health Studies | Gorgas Memorial Laboratory of Health Studies | Gonzalez Claudia, Leyda Abrego, Moreno Ambar, Oris Chavarria, Jessica Gondola, Marlenne Castillo, Ortiz Alma, Castillo Jorge, Moreno Brechla, Franco Danilo, Lopez-Verges Sandra, Martinez Alexander |
| EPI_ISL_1503073 | hCoV-19/Panama/GMI-PA699167/2021 | North America/Panama/Colon | 2021-02-07 | Gorgas Memorial Laboratory of Health Studies | Gorgas Memorial Laboratory of Health Studies | Gonzalez Claudia, Leyda Abrego, Moreno Ambar, Oris Chavarria, Jessica Gondola, Marlenne Castillo, Ortiz Alma, Castillo Jorge, Moreno Brechla, Franco Danilo, Lopez-Verges Sandra, Martinez Alexander |
| EPI_ISL_1503092 | hCoV-19/Panama/GMI-PA700792/2021 | North America/Panama/Cocle | 2021-02-13 | Gorgas Memorial Laboratory of Health Studies | Gorgas Memorial Laboratory of Health Studies | Gonzalez Claudia, Leyda Abrego, Moreno Ambar, Oris Chavarria, Jessica Gondola, Marlenne Castillo, Ortiz Alma, Castillo Jorge, Moreno Brechla, Franco Danilo, Lopez-Verges Sandra, Martinez Alexander |
| EPI_ISL_1503085 | hCoV-19/Panama/GMI-PA701950/2021 | North America/Panama/Metropolitana | 2021-02-11 | Gorgas Memorial Laboratory of Health Studies | Gorgas Memorial Laboratory of Health Studies | Gonzalez Claudia, Leyda Abrego, Moreno Ambar, Oris Chavarria, Jessica Gondola, Marlenne Castillo, Ortiz Alma, Castillo Jorge, Moreno Brechla, Franco Danilo, Lopez-Verges Sandra, Martinez Alexander |
| EPI_ISL_1503109 | hCoV-19/Panama/GMI-PA706928/2021 | North America/Panama/San Miguelito | 2021-02-19 | Gorgas Memorial Laboratory of Health Studies | Gorgas Memorial Laboratory of Health Studies | Gonzalez Claudia, Leyda Abrego, Moreno Ambar, Oris Chavarria, Jessica Gondola, Marlenne Castillo, Ortiz Alma, Castillo Jorge, Moreno Brechla, Franco Danilo, Lopez-Verges Sandra, Martinez Alexander |
| EPI_ISL_1503118 | hCoV-19/Panama/GMI-PA712608/2021 | North America/Panama/Herrera | 2021-02-28 | Gorgas Memorial Laboratory of Health Studies | Gorgas Memorial Laboratory of Health Studies | Gonzalez Claudia, Leyda Abrego, Moreno Ambar, Oris Chavarria, Jessica Gondola, Marlenne Castillo, Ortiz Alma, Castillo Jorge, Moreno Brechla, Franco Danilo, Lopez-Verges Sandra, Martinez Alexander |
| EPI_ISL_2105523 | hCoV-19/Philippines/PH-PGC-05778/2020 | Asia/Philippines/Central Visayas | 2020-10-30 | Governor Celestino Gallares Memorial Hospital | Philippine Genome Center | Francis A. Tablizo, Kenneth M. Kim, Carlo M. Lapid, Marc Jerrone R. Castro, Maria Sofia L. Yangzon, Benedict A. Maralit, Marc Edsel C. Ayes, Eva Maria Cutiongco-de la Paz, Alethea R. de Guzman, Jan Michael C. Yap, Jo-Hannah S. Llames, Sheila Mae M. Araiza, Kris P. Punayan, Irish Coleen A. Asin, Candice Francheska B. Tambaoan, Asia Louisa U. Chong, Karol Sophia Agape R. Padilla, Rianna Patricia S. Cruz, El King D. Morado, Joshua Gregor A. Dizon, Razel Nikka M. Hao, Arianne A. Zamora, Devon Ray Pacial, Juan Antonio R. Magalang, Marissa Alejandria, Celia Carlos, Anna Ong-Lim, Edsel Maurice SalvaÃ±a, John Q. Wong, Jaime C. Montoya, Maria Rosario Singh-Vergeire and Cynthia P. Saloma |
| EPI_ISL_2107355 | hCoV-19/USA/GA-EHC-1102L/2021 | North America/USA/Georgia | 2021-01-18 | Grady Memorial Hospital, Clinical Microbiology Laboratory | Piantadosi Lab, Emory Department of Pathology | Yun F (Wayne) Wang, Anne Piantadosi |
| EPI_ISL_2537249 | hCoV-19/USA/CO-GD-SID-21060102621/2021 | North America/USA/Colorado | 2021-06-01 | Gravity Diagnostics, LLC | Gravity Diagnostics, LLC | Gravity Diagnostics |
| EPI_ISL_2537193 | hCoV-19/USA/KY-GD-SID-21052548893/2021 | North America/USA/Kentucky | 2021-05-25 | Gravity Diagnostics, LLC | Gravity Diagnostics, LLC | Gravity Diagnostics |
[truncated: 1,030,735 more chars]
